# Multiple Introductions of Reassorted Highly Pathogenic Avian Influenza H5Nx Viruses Clade 2.3.4.4b Causing Outbreaks in Wild Birds and Poultry in The Netherlands, 2020-2021

**DOI:** 10.1128/spectrum.02499-21

**Published:** 2022-03-14

**Authors:** Marc Engelsma, Rene Heutink, Frank Harders, Evelien A. Germeraad, Nancy Beerens

**Affiliations:** a Wageningen Bioveterinary Research, Lelystad, the Netherlands; University of Prince Edward Island

**Keywords:** H5N8, HPAI, avian influenza virus, introduction routes, outbreak, reassortment, virus evolution

## Abstract

Highly pathogenic avian influenza (HPAI) viruses of subtype H5Nx caused outbreaks in poultry, captive birds, and wild birds in the Netherlands between October 2020 and June 2021. The full genome sequences of 143 viruses were analyzed. HPAI viruses were mainly of subtype H5N8, followed by H5N1, but also viruses of subtypes H5N3, H5N4, and H5N5 were detected. At least seven distinct genotypes were found, carrying closely related H5 segments belonging to clade 2.3.4.4b. Molecular clock analysis suggests that the reassortments of the NA gene segments likely occurred before the introduction of these viruses into the Netherlands. Genetic analysis suggested that multiple independent introductions of HPAI H5N8 viruses occurred in the Netherlands, likely followed by local spread resulting in at least two clusters of related viruses. The analysis provided evidence for independent introductions from wild birds at 10 poultry farms, whereas for two outbreaks transmission between farms could not be excluded. HPAI H5Nx viruses were detected in dead wild birds of 33 species, but mostly infected geese and swans were found. The pathogenicity of the H5N8 virus was determined for chickens and Pekin ducks, showing reduced mortality for ducks. This study provides more insight into the genetic diversity of HPAI H5Nx viruses generated by reassortment and evolution, and the spread of these viruses between wild birds and poultry. The fast and continuing evolution of H5 clade 2.3.4.4b may provide opportunities for these viruses to adapt to specific bird species, or possibly mammalian species and humans.

**IMPORTANCE** Highly pathogenic avian influenza (HPAI) viruses are spread by migratory wild birds. Viruses causing outbreaks in wild birds and poultry in the Netherlands in 2020–2021 were genetically analyzed, which suggested that multiple virus incursions occurred. The outbreaks in poultry were likely caused by independent introductions from wild birds; only in one case virus spread between farms could not be excluded. Viruses of subtype H5N8 were mainly observed, but also other subtypes were detected that likely evolved by exchange of genetic information before these viruses were introduced into the Netherlands. Viruses were detected in many species of dead wild birds, but mostly in geese and swans. We showed that the H5N8 virus causes a higher mortality in chickens compared to ducks. This is consistent with the fact that not many wild ducks were found dead. This study provides more insight in the evolution and spread of HPAI viruses in wild birds and poultry.

## INTRODUCTION

Since its first emergence in 1996 in Guangdong, China (1), highly pathogenic avian influenza (HPAI) virus of the H5N1 subtype has disseminated widely across Asia, Europe, and Africa, infecting a range of domestic and wild bird species (2, 3) and sporadically spilling over into humans (4, 5) and other mammals (6, 7). Over time, the HPAI H5 lineage evolved into multiple phylogenetically distinct sublineages, and reassortment with low pathogenic avian influenza viruses (LPAI) resulted in multiple subtypes and genotypes. H5 clade 2.3.4.4 has become dominant, and was recently divided into subclades a–h according to the World Health Organization (8).

Dissemination of HPAI H5 viruses has been linked to wild bird migration (9). Incursions of HPAI H5 clade 2.3.4.4 viruses have caused outbreaks in Europe in several years between 2014 and 2021, with significant differences in the number of outbreaks detected in poultry and wild birds (10). The H5N8 viruses detected in 2014 belong to clade 2.3.4.4A according to the nomenclature by Lee (11), but were recently classified as 2.3.4.4c in the updated nomenclature proposed by the WHO (8). These H5N8 viruses caused several outbreaks in poultry, but no wild bird mortality was observed. The epidemic in 2016 was especially severe and caused substantial mortality among wild birds, and poultry became infected across Europe; these outbreaks were caused by the introduction of H5N8 clade 2.3.4.4b viruses. The H5N6 viruses introduced in Europe in 2017 belonged to clade 2.3.4.4b and caused outbreaks in poultry but limited wild bird mortality. Then in late 2019 until June 2020, HPAI H5N8 clade 2.3.4.4b viruses caused outbreaks in Central and Eastern European countries. These viruses were generated by reassortment between HPAI H5N8 from sub-Saharan Africa and LPAI viruses from Eurasia (12, 13). In October 2020, a novel H5N8 clade 2.3.4.4b virus was first detected in the Netherlands in wild mute swans (14), and subsequently spread all over Europe, causing massive outbreaks in wild birds and poultry (15–20). Genetic analysis showed the novel virus was not related to viruses found earlier in 2020 in eastern Europe, but shares a common ancestor with HPAI H5 clade 2.3.4.4b viruses causing outbreaks in Eurasia between 2016 and 2018 (14). Prior to the introduction of the virus into Europe, genetically similar viruses have been detected in Russia, Kazakhstan, and Iraq in the summer of 2020 (16). The H5N8 virus may have been directly disseminated from these regions, or alternatively from breeding grounds in Siberia, by wild birds migrating to Europe for wintering. The routes by which HPAI H5 viruses are carried over long distances, as well as the specific migratory species involved, are still only partially known.

The HPAI H5 epidemic in the Netherlands in 2020–2021 caused massive wild bird mortality, affecting mostly species of swans and geese. During the previous epidemic in 2016–2017 a related H5N8 virus clade 2.3.4.4b also caused high mortality among wild birds (10), and in particular affected tufted ducks and Eurasian wigeons in the Netherlands (21). During the current epidemic only limited numbers of ducks were found dead, suggesting differences in the pathogenicity of these two HPAI clade 2.3.4.4b viruses for specific bird species. HPAI H5N8 and H5N1 viruses were detected in healthy Eurasian wigeons caught in the Netherlands (16). HPAI H5 viruses caused outbreaks on 12 commercial poultry farms and several captive bird facilities in the Netherlands in 2020–2021. In addition, sporadic spillover of the virus to mammals was observed. HPAI H5 viruses were detected in red foxes in the Netherlands (22) and in foxes and seals in the United Kingdom, (23) and human infections have been observed in Russia (24). During the 2020–2021 epidemic, several reassortant viruses were detected in the Netherlands. The genetic diversity generated through reassortment plays an important role in the evolution of avian influenza viruses and may provide the opportunity for the adaptation of HPAI H5-viruses to specific wild bird species, or possibly mammalian species. Despite this, little is known about the origin and genesis of these reassortants. In this study, we attempt to determine the source of all eight influenza virus gene segments, and to estimate when and where these reassortments occurred. We determined the full genome sequences of 143 wild bird, captive bird, and poultry viruses that were detected during the HPAI H5 epidemic in the Netherlands, between October 2020 and June 2021. The genetic relationship between these viruses was studied to elucidate possible spreading and introduction routes.

## RESULTS

### Outbreaks in wild birds.

**FIG 1:**
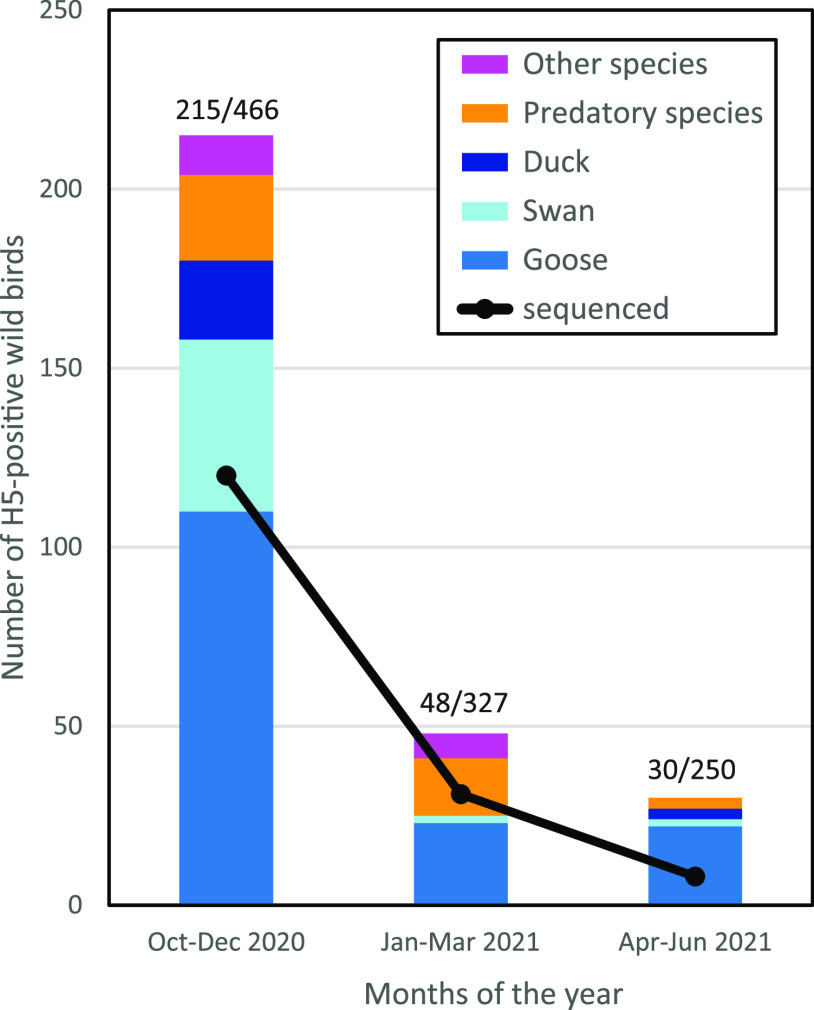
The number of dead wild birds of different species testing positive in the H5-PCR between October 2020 and June 2021. Shown is the number of viruses that were sequenced in this period (black line). The number of H5-positive birds and the total number of dead wild birds tested in this period are listed above the columns (H5+/total number of birds tested).

After the initial detection of HPAI H5N8 virus in two mute swans found dead on October 17, 2020, high wild bird mortality was observed in coastal and other water-rich areas in the Netherland. Dead wild birds were tested for the presence of H5-virus in the passive wild bird surveillance program. The number of dead wild birds testing positive for H5-virus was highest during the first 3 months of the epidemic (October–December), when a prevalence of 45% was measured for the birds tested (Fig. 1). This declined in later months; the prevalence of H5-virus among the dead wild birds tested was 15% in the first quarter and 12% in the second quarter of 2021. HPAI H5-viruses were mostly detected in species of geese, such as the barnacle goose (*Branta leucopsis*) and graylag goose (*Anser anser*), followed by swans (mute swan, *Cygnus olor*). However, many other species of wild birds tested positive for H5-virus, including predatory birds such as the common buzzard (*Buteo buteo*) and peregrine falcon (*Falco peregrinus*), and duck species such as the Eurasian wigeon (*Mareca penelope*) and mallard (Anas platyrhynchos). Besides HPAI H5N8 viruses, viruses of subtypes H5N1, H5N3, H5N4, and H5N5 were detected in dead wild birds. The wild bird species testing positive within the passive wild bird surveillance program and the subtypes of viruses detected between October 2020 and June 2021 are listed in Table S1 in the supplemental material.

### Outbreaks in poultry and pathogenicity of the H5N8 virus.

**FIG 2:**
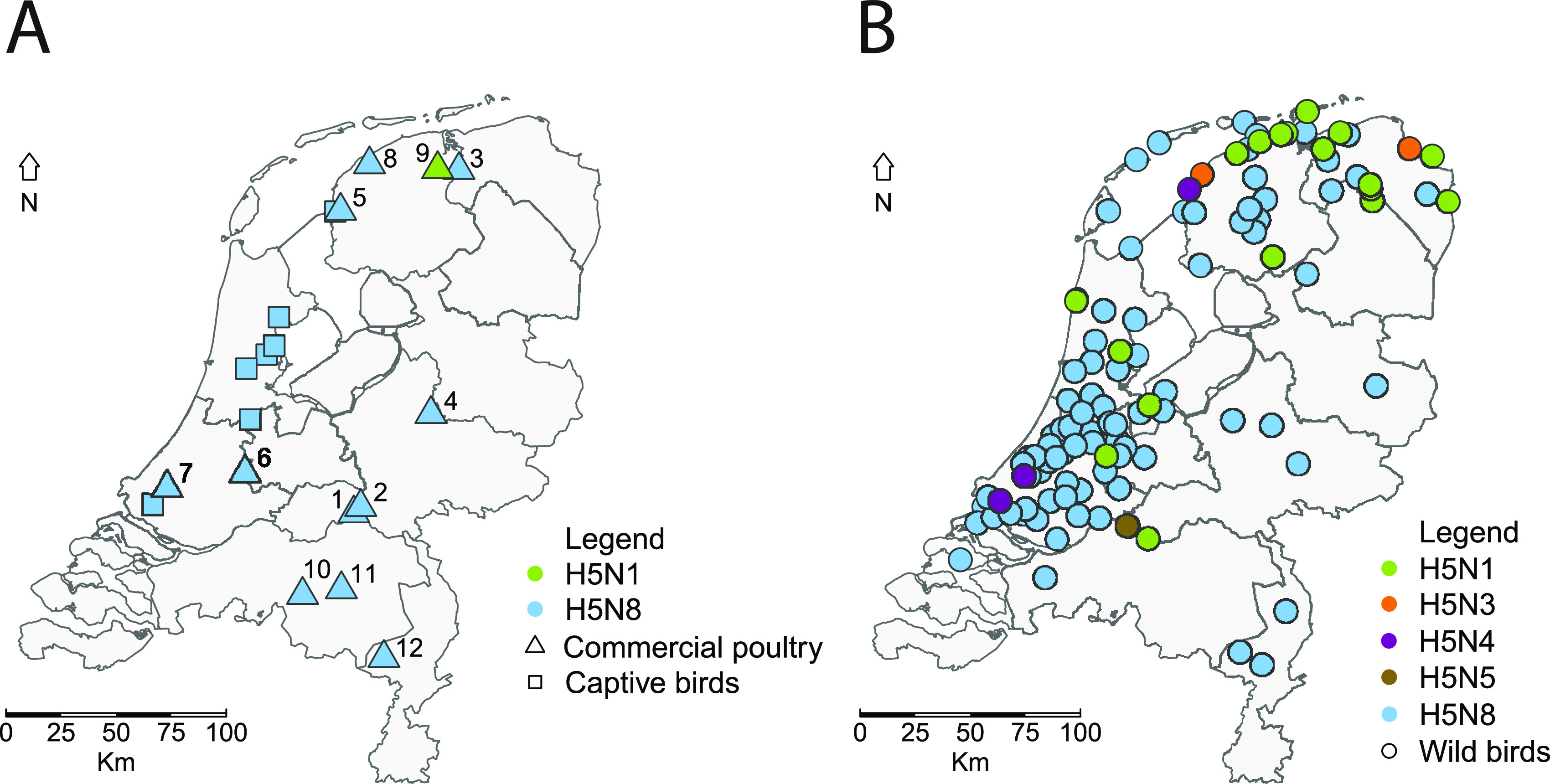
Geographical locations of HPAI H5-viruses detected and sequenced in the Netherlands (A) at commercial poultry farms (squares) and captive bird facilities (triangles) and (B) in dead wild birds (circles). The subtypes of viruses detected are marked: H5N8 (blue), H5N1 (green), H5N3 (orange), H5N4 (purple), and H5N5 (brown).

**FIG 3:**
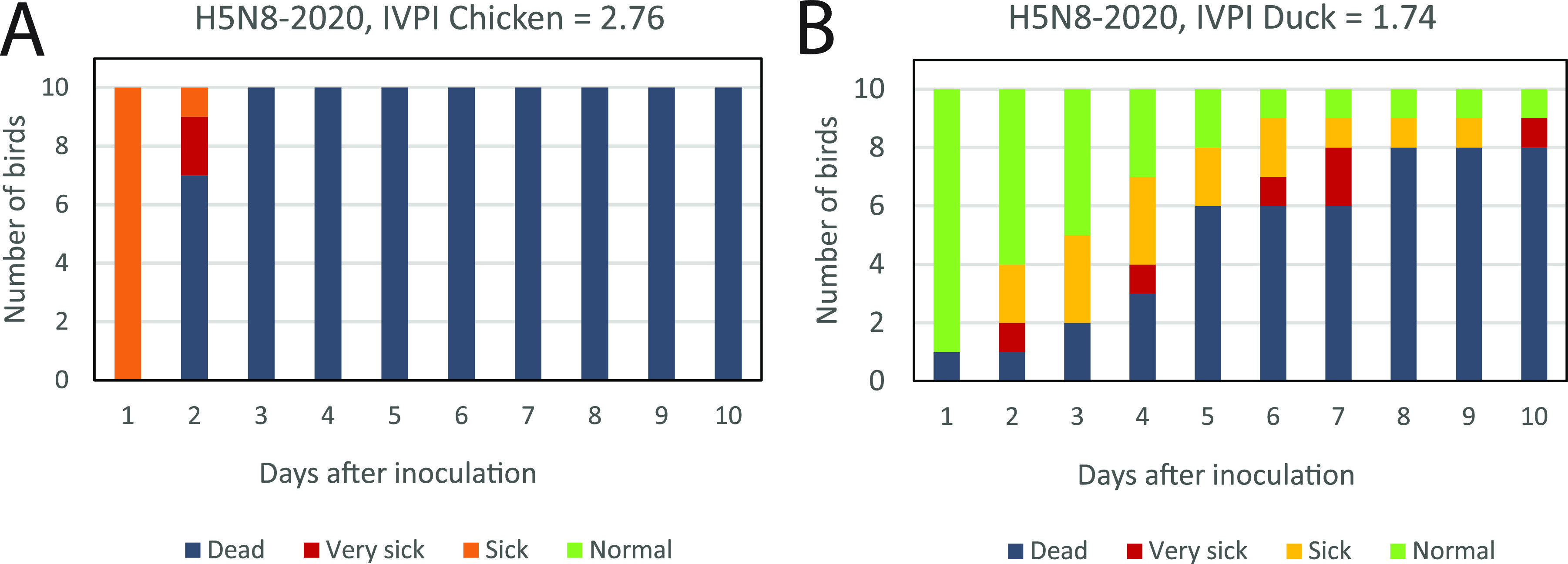
The intravenous pathogenicity index (IVPI) scores in (A) chickens and (B) Pekin ducks. Ten 6-weeks old birds were infected with the HPAI H5N8-2020 virus isolated from the index farm, and were monitored for clinical signs and death for 10 days. The number of normal (green), sick (orange), very sick (red), and dead (dark gray) birds is shown for 10 days after infection.

In the Netherlands, 12 commercial poultry farms (Fig. 2A, Table 1) and 8 captive bird facilities became infected during the 2020–2021 epidemic. The captive birds at the facilities were diverse, and included peacock, guineafowl, swan, goose, duck, and chicken species. The first introduction of HPAI H5N8 virus into a commercial poultry holding was detected on October 29 2020 and the last introduction was detected on May 21 2021. Poultry species affected included laying hens and broilers, turkeys, and Pekin ducks. One commercial chicken farm (farm 9) became infected with a HPAI subtype H5N1 virus; on the other farms, subtype H5N8 viruses were detected. The intravenous pathogenicity index (IVPI) was determined for the HPAI H5N8-2020 virus detected at the index farm (farm 1). The measured index of 2.76 confirmed the high pathogenicity of the H5N8 virus with cleavage site PLREKRRKR/GLF for chickens (Fig. 3A). A similar IVPI experiment was performed using Pekin ducks, for which a score of 1.74 was measured (Fig. 3B). Both disease onset and mortality were observed earlier in chickens compared to ducks in the IVPI experiments. All 10 chickens died within 3 days, whereas two ducks survived the 10-day experiment. A significant difference in the daily probability of mortality was determined between ducks and chickens using a logistic regression model (interaction day:species, *P* = 2.925e-07). These results suggest large differences in pathogenicity of the HPAI H5N8 virus for different poultry species.

**TABLE 1:** Outbreaks on commercial farms during the 2020–2021 HPAI H5 epidemic

Farm	Location	Poultry type	No. of birds	Virus subtype	Detection date	GISAID accession
1	Altforst	Broilers	35.700	HPAI H5N8	2020-10-29	EPI_ISL_603132
2	Puiflijk	Layers	100.000	HPAI H5N8	2020-11-05	EPI_ISL_623075
3	Lutjegast	Layers	48.000	HPAI H5N8	2020-11-10	EPI_ISL_632319
4	Terwolde	Pekin ducks	20.000	HPAI H5N8	2020-11-13	EPI_ISL_641518
5	Witmarsum	Broilers	90.000	HPAI H5N8	2020-11-21	EPI_ISL_653920
6	Hekendorp	Layers	100.000	HPAI H5N8	2020-11-22	EPI_ISL_653921
7	Maasland	Chickens	500	HPAI H5N8	2020-12-05	EPI_ISL_693515
8	St Annaparochie	Broilers	21.000	HPAI H5N8	2020-12-07	EPI_ISL_710539
9	Buitenpost	Layers	28.000	HPAI H5N1	2020-12-15	EPI_ISL_711055
10	Moergestel	Turkeys	18.000	HPAI H5N8	2021-01-05	EPI_ISL_775267
11	St Oedenrode	Layers	35.000	HPAI H5N8	2021-02-20	EPI_ISL_1046889
12	Weert	Turkeys	13.000	HPAI H5N8	2021-05-21	EPI_ISL_2227275

### Reassortants identified in the Netherlands.

**FIG 4:**
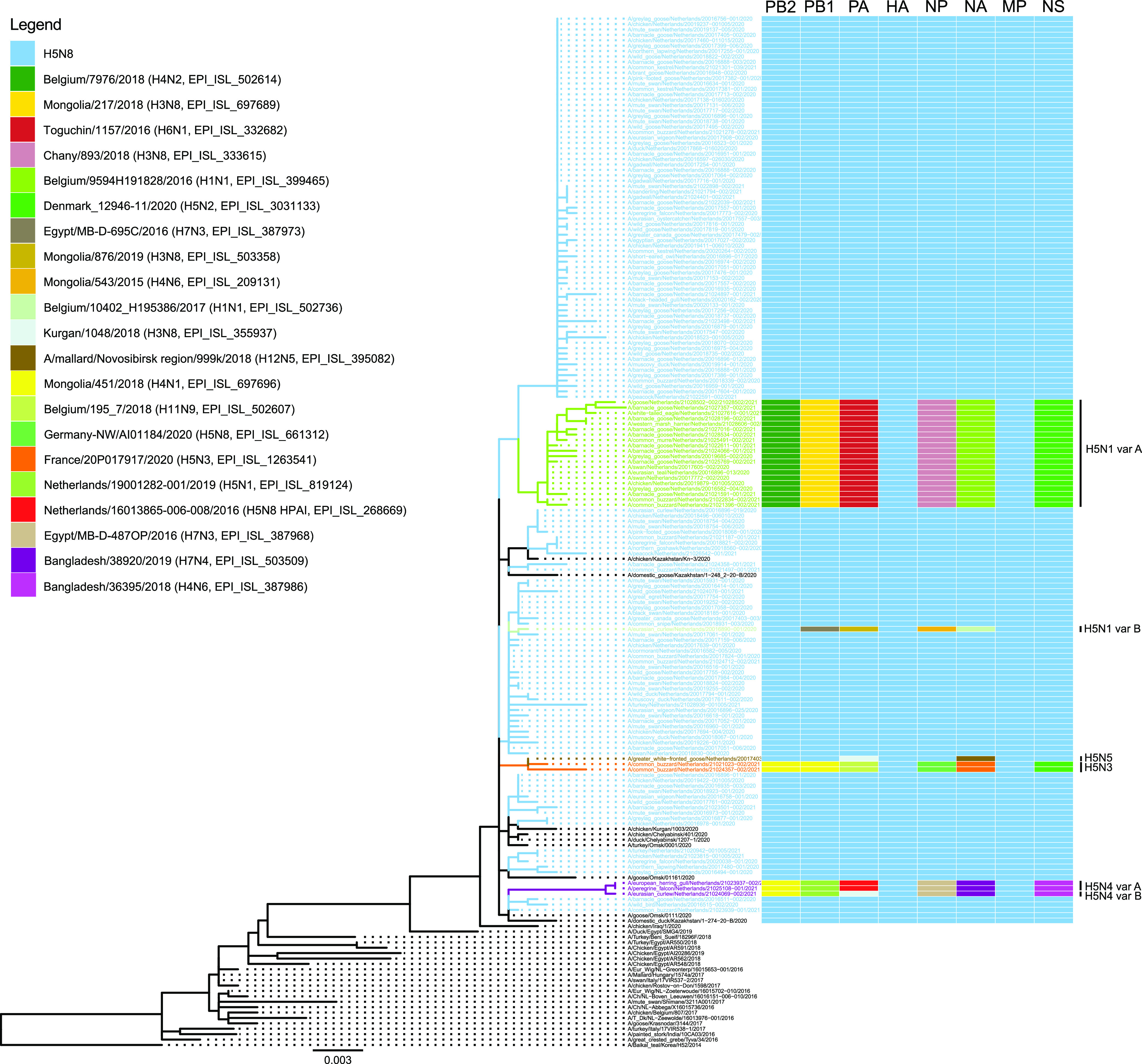
Phylogenetic tree of the HA segment of the poultry, captive, and wild bird viruses sequenced. HPAI H5N8 viruses sequenced in this study are shown in blue, H5N1 in green, H5N3 in orange, H5N4 in purple, and H5N5 in brown. Variants are marked by A or B. Reference viruses from other countries are shown in black. The reassortments for all gene segments are schematically shown. The closest related viruses for each gene segment are listed and are marked by color.

The full genome sequences of 143 viruses were determined to study the genetic diversity of the H5N8 viruses and reassortments leading to the subtypes H5N1, H5N3, H5N4, and H5N5 viruses. Deep sequencing was performed for all poultry and captive bird viruses detected in the Netherlands between October 2020 and June 2021 (Fig. 2A, Table S2), and for a representative set of viruses of wild birds found dead at different locations (Fig. 2B). Phylogenetic analysis was performed for the H5 and other gene segments (Fig. 4), revealing the introduction of 7 different genotypes. All H5N8 viruses detected in wild birds and poultry had a similar genetic constitution, but for the subtype H5N1 viruses two different genotypes were observed. H5N1 variant-A was detected in 20 wild birds sequenced at poultry farm 9, and has HA and MP segments similar to the H5N8 virus. Closest relatives for the other gene segments were detected in LPAI viruses circulating in wild birds in Eurasia between 2016 and 2020. The H5N1 variant-B virus was observed in one Eurasian curlew (*Numenius arquata*), and shares PB2, HA, MP, and NS segments with the H5N8 virus. The N1 segment of H5N1 variant-B is similar to that detected in variant-A, but different PB1, PA, and NP gene segments were found. The closest relatives for the PB1, PA, and NP segments were detected in LPAI viruses circulating in wild birds between 2015 and 2019 in Eurasia and Egypt. Subtype H5N3 was detected in two common buzzards, and contained HA and MP segments similar to the H5N8 virus. Closest relatives for the other gene segments were detected in LPAI viruses circulating in wild birds between 2018 and 2020 in wild birds in Eurasia. Two genotypes were detected for subtype H5N4 viruses; variant-A was detected in a European herring gull (*Larus argentatus*) and a peregrine falcon, and variant-B in one Eurasian curlew. Both variants had HA and MP segments similar to the H5N8 virus. Closest relatives for the other gene segments were detected in LPAI viruses circulating in wild birds in Eurasia between 2016 and 2019, but the origin of the PA segment differs between variant-A and -B. The PA segment in H5N4 variant-A was found previously in HPAI H5N8 viruses circulating in the Netherlands during the 2016–2017 epidemic, whereas that of variant-B was related to LPAI viruses detected in Russia in 2018. The H5N5 virus was detected in one greater white-fronted goose (*Anser albifrons*) and shared all gene segments with the H5N8 virus, except for NA and PA. The closest relatives for both the PA and NA segments were detected in 2018 in the Russian Federation.

### Evolutionary origin of the HA and NA gene segments.

The time to the most recent common ancestor (tMRCA) was calculated for the HA and NA segments of the HPAI H5-viruses to study the emergence of the HPAI H5-viruses detected during the epidemic in further detail. Taking the intersection of the 95% highest posterior density (HPD) intervals of the tMRCA, the ancestral virus carrying the H5 segment of the HPAI H5-viruses in 2020–2021 was estimated to have circulated between March 2019 and February 2020 (Table 2; Fig H5, node 1 in the supplemental material). The ancestral virus carrying the N8 segment of the HPAI H5N8 viruses detected in 2020–2021 likely circulated between April 2019 and March 2020 (Table 2; Fig N8, node 1 in the supplemental material).

**TABLE 2:** tMRCA of HA and NA gene segments of HPAI outbreak viruses in the Netherlands, 2020–2021

Segment	Node^*a*^	tMRCA^*b*^	Date	ht 95% HPD^*c*^				Posterior
				Lower	Date	Upper	Date	
HA_H5	1	2019.70	Sep 2019	2019.23	Mar 2019	2020.13	Feb 2020	0.97
NA_N1	1	2020.17	Mar 2020	2019.67	Sep 2019	2020.51	Jul 2020	1.00
	2	2015.51	Jul 2015	2014.83	Oct 2014	2016.23	Mar 2016	0.99
	3	2017.51	Jul 2017	2017.07	Jan 2017	2017.85	Nov 2017	0.44
NA_N3	1	2020.41	May 2020	2019.82	Oct 2019	2020.81	Oct 2020	0.82
	2	2020.08	Jan 2020	2019.25	Apr 2019	2020.66	Aug 2020	1.00
NA_N4	1	2020.62	Aug 2020	2020.18	Mar 2020	2020.93	Dec 2020	1.00
	2	2018.56	Jul 2018	2018.22	Mar 2018	2018.91	Nov 2018	0.62
NA_N5	1	2019.84	Nov 2019	2019.13	Feb 2019	2020.39	May 2020	0.99
	2	2018.50	Jun 2018	2018.10	Feb 2018	2018.73	Sep 2018	0.90
NA_N8	1	2019.82	Oct 2019	2019.30	Apr 2019	2020.20	Mar 2020	0.99

a Nodes of the time-scaled phylogenetic tree.

b tMRCA, time of the most recent common ancestor (median).

c HPD, highest posterior density interval.

The N1 gene segments of HPAI H5N1 variant-A viruses detected in 2020–2021 had a common ancestor that was estimated to have circulated around March 2020 (Table 2; Fig. S1, N1, node 1), and had a common ancestor with the closest related LPAI virus, A/Anas platyrhynchos/Belgium/9594H191828/2016, which circulated around July 2015 (node 2). Only one virus belonging to H5N1 genotype variant-B was reported during the 2020–2021 epidemic; the common ancestor with the closest related LPAI virus, A/Anas platyrhynchos/Belgium/1837_H101620/2018, likely circulated around July 2017, with a relatively low posterior value (Table 2; Fig. S1, N1, node 3). The common ancestor of the N3 gene segment of the HPAI H5N3 viruses detected in 2020–2021 was estimated around May 2020 (Table 2; Fig. S1, N3, node 1), and the common ancestor with the closest related LPAI virus, A/mallard/France/20P017917/2020 (H5N3), circulated around January 2020. The N4 gene segments of the HPAI H5N4 variant-A viruses detected in 2020–2021 had a common ancestor that was estimated around August 2020 (Table 2; Fig. S1, N4, node 1), and both H5N4 variant-A and -B viruses had a common ancestor with the closest related LPAI virus, A/garganey/Bangladesh/38920/2019 (H7N4), which circulated around July 2018 (node 2). The common ancestor of the N5 gene segment of the HPAI H5N5 viruses detected in 2020–2021 was estimated around November 2019 (Table 2; Fig. S1, N5, node 1), and the common ancestor with the closest related LPAI virus, A/mallard/Novosibirsk region/999k/2018 (H12N5), was estimated to have circulated around June 2018.

### Relationship between HPAI H5N8 wild bird and poultry viruses.

**FIG 5:**
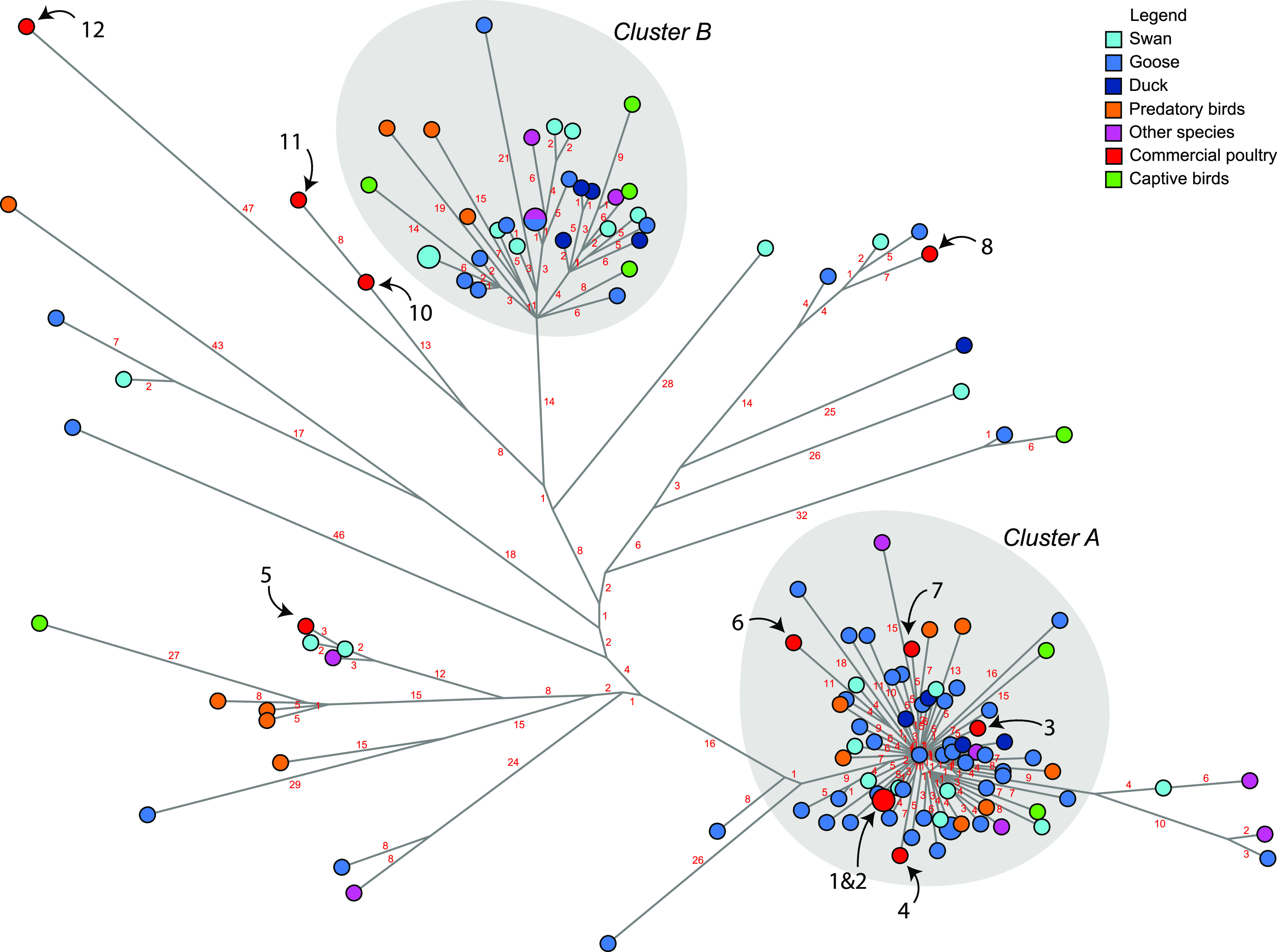
Median-joining network showing the genetic relationship between the HPAI H5N8 viruses isolated from commercial poultry farms (red), captive bird facilities (green), and dead wild birds submitted for testing. Wild birds were classified according to species: swan (light blue), goose (cobalt blue), duck (dark blue), predatory species (orange), and other species (magenta). The infected farms are marked by black numbers, and the number of nucleotide differences between viruses is shown in red numbers. Two virus clusters are present, marked A and B.

Median-joining network analysis of the eight concatenated gene segments was performed to study the relationship between HPAI H5N8 viruses isolated from wild birds and poultry (Fig. 5). This analysis showed that the viruses divide into two major clusters (cluster A and B), suggesting that at least two separate introductions of HPAI H5N8 viruses occurred in the Netherlands, after which the viruses may have been locally amplified. The common ancestor of the viruses in cluster A was detected in a barnacle goose (EPI_ISL_1139080, sample date 2020-10-29) early in the epidemic; the viruses in cluster B evolved from a predicted common ancestor that was not detected. In addition to the two major clusters, multiple separate viruses and small clusters were detected. This suggests that more independent introductions of H5N8 viruses have occurred in the Netherlands during the epidemic. The network analysis showed no correlation between virus cluster and bird species. In addition, no correlation with geographical location or month of isolation was detected (results not shown).

The HPAI H5N8 viruses detected at six commercial poultry farms belong to cluster A, whereas cluster B does not contain viruses from commercial poultry farms. The viruses detected at the other five farms are found at different locations in the network. The sequences of the viruses detected at farms 1 and 2 were identical, 8 nucleotide differences were found between farms 10 and 11, and numerous nucleotide differences were found between the viruses detected at the other poultry farms (Table S3). The genetic results indicate that the virus may have been transmitted between farms 1 and 2, although an infection from the same outside source cannot be excluded. The viruses detected at the other farms were likely the result of separate introductions from wild birds. The viruses isolated from the poultry farms were most closely related to viruses detected in goose or swan species; however, these species were also mostly tested within the passive wild bird surveillance program. For farms 10, 11, and 12, no closely related viruses were found in wild birds. The number of nucleotide differences between the poultry viruses detected at farms 1 to 8 and the closest related wild bird virus varied between 4 and 13 nucleotides (Table S4).

## DISCUSSION

The Netherlands was first to report HPAI H5N8 virus in Europe, when the virus was detected in two mute swans found dead on 17 October 2020 (14). Subsequently, H5N8 viruses were detected in other species of wild birds and poultry in the Netherlands, and in many other European countries. In the Netherlands, 11 commercial poultry farms and 8 captive bird facilities were affected by H5N8 viruses, whereas at one commercial poultry farm HPAI H5N1 virus was detected (farm 9). Phylogenetic analysis showed that the H5N8 viruses introduced in Europe in 2020–2021 are related to viruses detected in Russia, Kazakhstan, and Iraq in the summer of 2020 (16), and have a similar genetic constitution as the HPAI H5N8 clade 2.3.4.4b viruses that caused the epidemic in 2016–2017 in Europe. In this study, we showed that the H5N8-2020 virus is less pathogenic for Pekin ducks (IVPI 1.74) compared to chickens (IVPI 2.76). We previously performed IVPI experiments for the related HPAI H5N8-2016 virus and measured scores of 3.0 for both chickens and Pekin ducks (25). Natural infection predominantly occurs via the respiratory route, whereas the virus was inoculated intravenously in the IVPI experiments. Potential differences in virus attachment and entry via the respiratory tract therefore cannot be assessed in these experiments. Sequence analysis shows that the HA proteins of the H5N8 clade 2.3.4.4b viruses isolated from the index cases in poultry in 2016 and 2020 differ at 7 positions (results not shown). For these positions, no functional role has been described, which suggests a similar entry efficiency for both viruses. The novel H5N8-2020 virus caused severe wild bird mortality, affecting mostly species of swans and goose, whereas the related HPAI H5N8-2016 virus caused severe mortality among duck species during the 2016–2017 epidemic in the Netherlands (21). These results may suggest that HPAI H5N8 virus evolved toward lower pathogenicity for duck species, but increased pathogenicity for species of swans and geese.

HPAI H5-viruses were detected in at least 33 different species of wild birds in the passive surveillance program. The majority of the viruses detected during the 2020–2021 epidemic in the Netherlands was of subtype H5N8, but four other subtypes (H5N1, H5N3, H5N4 and H5N5) were detected. Subtype H5N1 was the second most detected subtype, and both H5N8 and H5N1 viruses were mostly detected in birds of the Anatidae family, specifically in geese and swans. However, the H5N3 and H5N4 subtypes of viruses were detected in bird species not belonging to the Anatidae family, such as the Eurasian curlew, common buzzard, European herring gull, and peregrine falcon. The complete genome sequences of 143 wild bird, captive bird, and poultry viruses were determined to study their genetic relationship. The analysis revealed that viruses of 7 different genotypes were introduced in the Netherlands. All viruses had similar HA and MP segments, but other segments were derived from LPAI viruses circulating in wild birds between 2015 and 2020 in Eurasia and Egypt. Molecular clock analysis suggests that the H5 and N8 segments of the HPAI H5N8 viruses detected in the Netherlands share an ancestor with viruses circulating in Eurasia around September–October 2019. The common ancestors of the HPAI H5 viruses with N1, N3, N4, and N5 segments were estimated to have circulated between November 2019 and August 2020. This suggests that although the HPAI H5N3 (first detection 2021-01-04) and HPAI H5N4 (first detection 2021-02-15) viruses were detected relatively late in the epidemic, these reassortments likely did not occur within the Netherlands. The common ancestors with LPAI viruses carrying the NA gene segments were likely circulating around January 2020 for N3, and between 2015 and 2018 for N1, N4, and N5 in Eurasia and Egypt. No related LPAI viruses were detected more recently, suggesting under-surveillance of the genetic diversity of avian influenza viruses in both wild birds and poultry worldwide. Surveillance for avian influenza viruses, and the subsequent sequencing and publication of these sequences, is important for insight into the location and timing of reassortment events. Extensive surveillance may provide an early warning for the emergence of novel reassortants and sequence mutations. Analysis of the sequences in this study did not identify known zoonotic mutations (e.g., PB2 D701N or E672K) in wild bird or poultry viruses (results not shown). The large variety of genotypes detected in the Netherlands provides evidence for rapid and continuing reassortment of the H5 clade 2.3.4.4b viruses, which allows the virus to change its genetic architecture very quickly and may increase the ability of the virus to infect poultry or humans in the future.

Median-joining network analysis of wild bird and poultry HPAI H5N8 viruses suggests at least two virus incursions occurred that were followed by local amplification of the virus, resulting in clusters A and B. The viruses found in the index case in wild birds (mute swans) and the index case in poultry (chickens) are located in cluster A (14). The viruses in cluster A may have evolved from a common ancestor virus that was detected in a barnacle goose early in the epidemic (EPI_ISL_1139080, detection date 2020-10-29), which was found in the center of cluster A. The HPAI H5N8 viruses in cluster B evolved from a predicted common ancestor that was not isolated during the epidemic. HPAI H5N8 viruses were mostly detected in barnacle geese, in particular early in the epidemic, suggesting that this migratory bird species may have played a role in virus introduction and amplification. However, barnacle geese appeared highly affected by virus infection resulting in high mortality. Therefore, the HPAI H5N8 virus may have been introduced by a carrier species not showing (severe) disease symptoms. These bird species, for which infection does not result in mortality, will be underrepresented in passive wild bird surveillance programs. Both clusters A and B also contain viruses isolated from bird species not belonging to the Anatidae family. At this moment the role of other bird species and residential wild birds in the amplification of virus circulation, and the persistence of circulation in the spring and summer months, is unknown. In addition to the two major clusters, multiple separate viruses and small clusters were detected. This suggests that more independent introductions of HPAI H5N8 viruses have occurred in the Netherlands in 2020–2021.

Poultry viruses were found in cluster A and in other locations of the network. The outbreaks on farms 11 and 12 occurred later in the epidemic, and limited numbers of wild birds were found dead geographically close to these farms. Several nucleotide differences were identified between the viruses detected at most farms (farms 3–12). These farms were likely infected by separate introductions from wild birds. This is further supported by the epidemiological investigation, which revealed no links between the poultry farms. For farms 1 and 2, identical viruses were detected, which could indicate either transmission between farms, or an introduction from the same source (wild birds). The two farms were located within 3 km distance, and the virus was detected within a period of 7 days, but no potential hazardous contacts were identified. With the exception of this outbreak, the analysis demonstrated that no virus transmission occurred between the farms. Fast diagnostics for HPAI viruses, rapid culling of the farms, and establishment of restriction and protection zones showed to be effective in preventing secondary spread. The network analysis identified viruses isolated from geese and swans as the most closely related to viruses detected at commercial poultry farms. These bird species can often be found grazing in agricultural areas surrounding poultry farms in the Netherlands. However, these species were also most affected by the infection with HPAI H5N8 virus and most tested in the dead wild bird surveillance program. The results emphasize the need for poultry farmers to prevent direct or indirect contact between poultry and wild birds by strictly following hygienic measures.

## MATERIALS AND METHODS

### Virus detection and subtyping.

Viral RNA was extracted from tracheal or cloacal swabs from dead wild birds using MagNa Pure 96 (Roche). For commercial poultry farms, pools of five tracheal or five cloacal swabs from clinically affected animals were analyzed. Samples were tested in a matrix-gene real-time PCR, which detects all avian influenza virus subtypes, as described previously (13). Positive samples were subtyped using an H5-specific real-time PCR as recommended by the European Union reference laboratory (14). The sequence of the HA cleavage site and the N-subtype was determined by Sanger sequencing (13).

### IVPI in chickens and ducks.

The Intravenous Pathogenicity Index (IVPI) was determined for the H5N8-2020 virus in chickens according to the standard procedure (26). A similar experiment following the same procedure was performed in Pekin ducks. The virus used in this study was derived from the index case in commercial poultry (EPI_ISL_603132_A/chicken/Netherlands/20016597-026030/2020_H5N8), and isolated by two passages in 9- to 11-day-old specific pathogen free (SPF) embryonated chicken eggs. The full genome sequence of the isolated virus was also determined (EPI_ISL_8650948_A/chicken/Netherlands/20016597-026030/2020_H5N8_E2), and no nucleotide differences were observed, excluding potential adaptations during egg passaging. Ten 6-week-old specific-pathogen-free (SPF) White Leghorn chickens were obtained from Royal GD (Deventer, the Netherlands), and 10 6-week-old Pekin ducks were obtained from a commercial breeding farm. All animals used in this study were from both sexes. Swimming ponds were provided to the ducks. The ducks tested negative for AI virus and antibodies on the day of arrival and after an acclimatization period of 7 days. The animals were tested for the absence of antibodies against AI using in-house ELISA as described previously (27), and for virus excretion by PCR as described above. After intravenous inoculation, the ducks were monitored for clinical signs and mortality for 10 days. The experiment was performed in biosafety level 3 (BSL3) facilities at Wageningen Bioveterinary Research (WBVR, Lelystad, the Netherlands), under the approval of the Central Animal Experiments Committee (license number 2017.D-0054.001) in the Netherlands. The probability (proportion) of daily mortality was compared between ducks and chickens in the IVPI experiments by fitting a logistic regression model where the proportion of dead animals was modeled as a function of the day postchallenge, bird species, and the interaction between day and species. Variable significance was assessed by the likelihood ratio test.

### Complete genome sequencing and analysis.

All viral genome sequences were determined directly on the swab samples. Viral RNA was purified using the High Pure Viral RNA kit (Roche), and amplified using universal eight-segment primers and directly sequenced, as described previously (15). Purified amplicons were sequenced at high coverage (average > 1000 per nt position) using the Illumina DNA Prep method and Illumina MiSeq 150PE sequencing. The reads were mapped using the ViralProfiler-Workflow, an extension of the CLC Genomics Workbench (Qiagen, Germany). Consensus sequences were generated by a reference-based method. Reads were first mapped to a reference set of genomes, and subsequently remapped to the closest reference sequence. Finally, the consensus sequence of the complete virus genome was extracted. The Workflow assigns the HA cleavage site and coding region of the proteins, which were used as secondary controls for the integrity of consensus sequences generated. Sequences generated in this study were submitted to the GISAID database; the accession numbers are listed in Table S1.

### Phylogenetic analysis.

In addition to the 143 viral genome sequences obtained in this study, the available H5N8 genome sequences from 2014 onward (HA and NA segment) or May 2020 (other segments) were downloaded from the GISAID database (accession date September 9th, 2021). For the H5N1, H5N3, H5N4, and H5N5 sequences, GISAID blast analyses were carried out for the reassorted gene segments using representative strains (H5N1, EPI_ISL_632314 A/greylag_goose/Netherlands/20016582-004/2020 and EPI_ISL_632316 A/eurasian_curlew/Netherlands/20016890-001/2020; H5N3, EPI_ISL_1048239 A/common_buzzard/Netherlands/21021023-002/2021; H5N4, EPI_ISL_2173364 A/peregrine_falcon/Netherlands/21025108-001/2021, and EPI_ISL_1225079 A/eurasian_curlew/Netherlands/21024069-002/2021; H5N5, EPI_ISL_641378 A/greater_white-fronted_goose/Netherlands/20017403-004/2020), and the top 100 BLAST results were used for the analysis. Phylogenetic analysis of the complete genome sequences was performed for each genome segment separately, aligning the viral sequences using MAFFT v7.475 (28), reconstructing the phylogeny using maximum likelihood (ML) analysis with IQ-TREE v2.0.3 and 1,000 ultrafast bootstrap replicates (29), and visualizing the ML tree using FigTree1.4.4 (21). The ML analysis results of the gene segments were subsequently summarized in a phylogenetic HA tree with heatmap of the associated segments. The phylogenetic heatmap tree was created using the function gheatmap from the R package ggtree (30).

### Network analysis.

For network analysis, the eight gene segment alignments of the H5N8 viral genome sequences from the current outbreak in the Netherlands were concatenated to generate a single alignment. This was used to construct phylogenetic networks using the median-joining method implemented in the program NETWORK (Fluxus Technology Ltd.; 31), as described previously (17). This model-free method uses a parsimony approach, based on pairwise differences, to connect each sequence to its closest neighbor, and allows creation of internal nodes (“median vectors”), which could be interpreted as unsampled or extinct ancestral genotypes to link the existing genotypes in the most parsimonious way.

### Molecular clock analysis.

For molecular clock analyses. the HA and NA data sets described above were analyzed for clock-likeness in TempEst (32) and outliers removed from the data set. The HA H5 and NA N8 data sets were down-sampled by random sampling to ≤ 50 sequences per year from years 2020 and 2021 and 50 sequences from the predecessors. For each data set, maximum clade credibility trees were inferred using BEAST v1.10.4 (33). In all analysis, an uncorrelated lognormal relaxed molecular clock, SRD codon position model with the HKY model with gamma distribution rates and the Gaussian Markov random Field (GMRF) skyride coalescent model, was used. Three independent analyses of 100 million generations, sampling every 10,000 generations, were performed. Convergence of the runs was checked in Tracer v1.7.1 for adequate mixing and sufficient effective samples size of all parameters. The runs were combined after removing the initial 10% burnin. The maximum clade credibility trees were visualized in FigTree v1.4.4. For the origin and acknowledgments of the used sequences downloaded from GISAID for the final analysis, see Table S5.
