# Supplementary material for: Multiple Introductions of Reassorted Highly Pathogenic Avian Influenza H5Nx Viruses Clade 2.3.4.4b Causing Outbreaks in Wild Birds and Poultry in The Netherlands, 2020-2021
Source: Microbiol Spectr. 2022 Mar 14;10(2):e02499-21. doi: 10.1128/spectrum.02499-21 (PMC9045216; doi:10.1128/spectrum.02499-21)
Supplement: SUPPLEMENTAL FILE 1 — Supplemental material. Download SPECTRUM02499-21_Supp_1_seq8.pdf, PDF file, 1.8 MB [file spectrum02499-21_supp_1_seq8.pdf]

HA H5

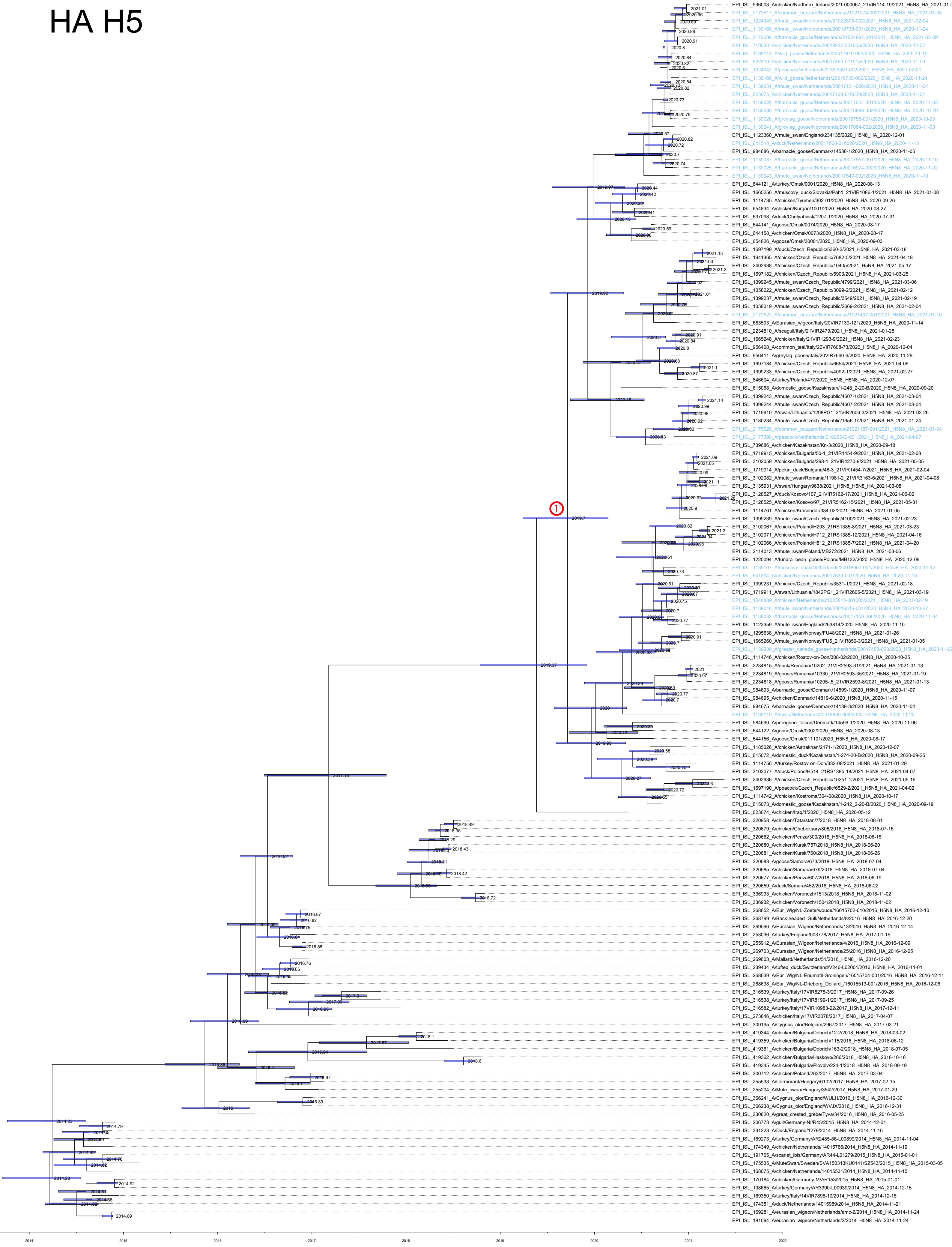

EPI_ISL_996003_A/chicken/Northern_Ireland/2021-000067_21VIR114-19/2021_H5N8_HA_2021-01-0
EPI_ISL_2172517_A/common_buzzard/Netherlands/21021278-002/2021_H5N8_HA_2021-01-05
EPI_ISL_1224949_A/mute_swan/Netherlands/21022898-002/2021_H5N8_HA_2021-02-04
EPI_ISL_1139168_A/mute_swan/Netherlands/20018738-001/2020_H5N8_HA_2020-11-24
EPI_ISL_2172608_A/barnacle_goose/Netherlands/21024897-001/2021_H5N8_HA_2021-03-09
EPI_ISL_710533_A/chicken/Netherlands/20019237-001005/2020_H5N8_HA_2020-12-02
EPI_ISL_1139113_A/wild_goose/Netherlands/20017819-001/2020_H5N8_HA_2020-11-10
EPI_ISL_632319_A/chicken/Netherlands/20017460-011015/2020_H5N8_HA_2020-11-09
EPI_ISL_1224942_A/peacock/Netherlands/21022591-002/2021_H5N8_HA_2021-02-01
EPI_ISL_1139166_A/wild_goose/Netherlands/20018735-002/2020_H5N8_HA_2020-11-24
EPI_ISL_1139031_A/mute_swan/Netherlands/20017131-006/2020_H5N8_HA_2020-11-04
EPI_ISL_623075_A/chicken/Netherlands/20017138-016020/2020_H5N8_HA_2020-11-04
EPI_ISL_1139028_A/barnacle_goose/Netherlands/20017051-001/2020_H5N8_HA_2020-11-03
EPI_ISL_1139080_A/barnacle_goose/Netherlands/20016888-003/2020_H5N8_HA_2020-10-29
EPI_ISL_1139020_A/greylag_goose/Netherlands/20016756-001/2020_H5N8_HA_2020-10-29
EPI_ISL_1139041_A/greylag_goose/Netherlands/20017064-002/2020_H5N8_HA_2020-11-03
EPI_ISL_1123360_A/mute_swan/England/234135/2020_H5N8_HA_2020-12-01
EPI_ISL_641518_A/duck/Netherlands/20017868-016020/2020_H5N8_HA_2020-11-13
EPI_ISL_984686_A/barnacle_goose/Denmark/14536-1/2020_H5N8_HA_2020-11-05
EPI_ISL_1139087_A/barnacle_goose/Netherlands/20017557-001/2020_H5N8_HA_2020-11-10
EPI_ISL_1139025_A/barnacle_goose/Netherlands/20016974-002/2020_H5N8_HA_2020-11-02
EPI_ISL_1139043_A/mute_swan/Netherlands/20017547-002/2020_H5N8_HA_2020-11-10
EPI_ISL_644121_A/turkey/Omsk/0001/2020_H5N8_HA_2020-08-13
EPI_ISL_1665256_A/muscovy_duck/Slovakia/Pah1_21VIR1086-1/2021_H5N8_HA_2021-01-08
EPI_ISL_1114735_A/chicken/Tyumen/302-01/2020_H5N8_HA_2020-09-26
EPI_ISL_654834_A/chicken/Kurgan/1001/2020_H5N8_HA_2020-08-27
EPI_ISL_637098_A/duck/Chelyabinsk/1207-1/2020_H5N8_HA_2020-07-31
EPI_ISL_644141_A/goose/Omsk/0074/2020_H5N8_HA_2020-08-17
EPI_ISL_644158_A/chicken/Omsk/0073/2020_H5N8_HA_2020-08-17
EPI_ISL_654826_A/goose/Omsk/30001/2020_H5N8_HA_2020-09-03
EPI_ISL_1697199_A/duck/Czech_Republic/5360-2/2021_H5N8_HA_2021-03-18
EPI_ISL_1941365_A/chicken/Czech_Republic/7682-5/2021_H5N8_HA_2021-04-18
EPI_ISL_2402938_A/chicken/Czech_Republic/10405/2021_H5N8_HA_2021-05-17
EPI_ISL_1697182_A/chicken/Czech_Republic/5903/2021_H5N8_HA_2021-03-25
EPI_ISL_1399245_A/mute_swan/Czech_Republic/4799/2021_H5N8_HA_2021-03-06
EPI_ISL_1058022_A/chicken/Czech_Republic/3099-2/2021_H5N8_HA_2021-02-12
EPI_ISL_1399237_A/mute_swan/Czech_Republic/3549/2021_H5N8_HA_2021-02-19
EPI_ISL_1058019_A/mute_swan/Czech_Republic/2669-2/2021_H5N8_HA_2021-02-04
EPI_ISL_2172522_A/common_buzzard/Netherlands/21021497-001/2021_H5N8_HA_2021-01-10
EPI_ISL_683593_A/Eurasian_wigeon/Italy/20VIR7139-121/2020_H5N8_HA_2020-11-14
EPI_ISL_2234810_A/seagull/Italy/21VIR2479/2021_H5N8_HA_2021-01-28
EPI_ISL_1665248_A/chicken/Italy/21VIR1293-9/2021_H5N8_HA_2021-02-23
EPI_ISL_956408_A/common_teal/Italy/20VIR7608-73/2020_H5N8_HA_2020-12-04
EPI_ISL_956411_A/greylag_goose/Italy/20VIR7660-6/2020_H5N8_HA_2020-11-29
EPI_ISL_1697184_A/chicken/Czech_Republic/6654/2021_H5N8_HA_2021-04-06
EPI_ISL_1399233_A/chicken/Czech_Republic/4092-1/2021_H5N8_HA_2021-02-27
EPI_ISL_846604_A/turkey/Poland/477/2020_H5N8_HA_2020-12-07
EPI_ISL_615068_A/domestic_goose/Kazakhstan/1-248_2-20-B/2020_H5N8_HA_2020-09-20
EPI_ISL_1399243_A/mute_swan/Czech_Republic/4607-1/2021_H5N8_HA_2021-03-04
EPI_ISL_1399244_A/mute_swan/Czech_Republic/4607-2/2021_H5N8_HA_2021-03-04
EPI_ISL_1719910_A/swan/Lithuania/1298PG1_21VIR2606-3/2021_H5N8_HA_2021-02-26
EPI_ISL_1180234_A/mute_swan/Czech_Republic/1656-1/2021_H5N8_HA_2021-01-24
EPI_ISL_2175628_A/common_buzzard/Netherlands/21021187-001/2021_H5N8_HA_2021-01-04
EPI_ISL_2177398_A/peacock/Netherlands/21026542-001/2021_H5N8_HA_2021-04-07
EPI_ISL_739686_A/chicken/Kazakhstan/Kn-3/2020_H5N8_HA_2020-09-18
EPI_ISL_1719915_A/chicken/Bulgaria/50-1_21VIR1454-9/2021_H5N8_HA_2021-02-08
EPI_ISL_3102059_A/chicken/Bulgaria/298-1_21VIR4270-9/2021_H5N8_HA_2021-05-05
EPI_ISL_1719914_A/pekin_duck/Bulgaria/48-3_21VIR1454-7/2021_H5N8_HA_2021-02-04
EPI_ISL_3102082_A/mute_swan/Romania/11981-2_21VIR3163-6/2021_H5N8_HA_2021-04-08
EPI_ISL_3135931_A/swan/Hungary/9638/2021_H5N8_H5N8_HA_2021-03-08
EPI_ISL_3128527_A/duck/Kosovo/107_21VIR5162-17/2021_H5N8_HA_2021-06-02
EPI_ISL_3128525_A/chicken/Kosovo/97_21VIR5162-15/2021_H5N8_HA_2021-05-31
EPI_ISL_1114761_A/chicken/Krasnodar/334-02/2021_H5N8_HA_2021-01-05
EPI_ISL_1399239_A/mute_swan/Czech_Republic/4100/2021_H5N8_HA_2021-02-23
EPI_ISL_3102067_A/chicken/Poland/H293_21RS1385-8/2021_H5N8_HA_2021-03-23
EPI_ISL_3102071_A/chicken/Poland/H712_21RS1385-12/2021_H5N8_HA_2021-04-16
EPI_ISL_3102066_A/chicken/Poland/H812_21RS1385-7/2021_H5N8_HA_2021-04-20
EPI_ISL_2114013_A/mute_swan/Poland/MB272/2021_H5N8_HA_2021-03-06
EPI_ISL_1220094_A/tundra_bean_goose/Poland/MB132/2020_H5N8_HA_2020-12-09
EPI_ISL_1139107_A/muscovy_duck/Netherlands/20018067-001/2020_H5N8_HA_2020-11-12
EPI_ISL_641394_A/chicken/Netherlands/20017639-001/2020_H5N8_HA_2020-11-10
EPI_ISL_1399231_A/chicken/Czech_Republic/3531-1/2021_H5N8_HA_2021-02-18
EPI_ISL_1719911_A/swan/Lithuania/1842PG1_21VIR2606-5/2021_H5N8_HA_2021-03-19
EPI_ISL_1046889_A/chicken/Netherlands/21023815-001005/2021_H5N8_HA_2021-02-19
EPI_ISL_1139016_A/mute_swan/Netherlands/20016516-001/2020_H5N8_HA_2020-10-27
EPI_ISL_1139032_A/barnacle_goose/Netherlands/20017159-006/2020_H5N8_HA_2020-11-04
EPI_ISL_1123359_A/mute_swan/England/263814/2020_H5N8_HA_2020-11-10
EPI_ISL_1295638_A/mute_swan/Norway/FU48/2021_H5N8_HA_2021-01-26
EPI_ISL_1665260_A/mute_swan/Norway/FU5_21VIR850-3/2021_H5N8_HA_2021-01-05
EPI_ISL_1139086_A/greater_canada_goose/Netherlands/20017403-003/2020_H5N8_HA_2020-11-0
EPI_ISL_1114746_A/chicken/Rostov-on-Don/308-02/2020_H5N8_HA_2020-10-25
EPI_ISL_2234815_A/duck/Romania/10202_21VIR2593-31/2021_H5N8_HA_2021-01-13
EPI_ISL_2234819_A/goose/Romania/10330_21VIR2593-35/2021_H5N8_HA_2021-01-19
EPI_ISL_2234818_A/goose/Romania/10205-t5_21VIR2593-8/2021_H5N8_HA_2021-01-13
EPI_ISL_984693_A/barnacle_goose/Denmark/14599-1/2020_H5N8_HA_2020-11-07
EPI_ISL_984695_A/chicken/Denmark/14819-6/2020_H5N8_HA_2020-11-15
EPI_ISL_984675_A/barnacle_goose/Denmark/14139-3/2020_H5N8_HA_2020-11-04
EPI_ISL_1139110_A/swan/Netherlands/20018830-004/2020_H5N8_HA_2020-11-25
EPI_ISL_984690_A/peregrine_falcon/Denmark/14596-1/2020_H5N8_HA_2020-11-06
EPI_ISL_644122_A/goose/Omsk/0002/2020_H5N8_HA_2020-08-13
EPI_ISL_644156_A/goose/Omsk/011101/2020_H5N8_HA_2020-08-17
EPI_ISL_1185026_A/chicken/Astrakhan/2171-1/2020_H5N8_HA_2020-12-07
EPI_ISL_615072_A/domestic_duck/Kazakhstan/1-274-20-B/2020_H5N8_HA_2020-09-25
EPI_ISL_1114756_A/turkey/Rostov-on-Don/332-08/2021_H5N8_HA_2021-01-29
EPI_ISL_3102077_A/duck/Poland/H514_21RS1385-18/2021_H5N8_HA_2021-04-07
EPI_ISL_2402936_A/chicken/Czech_Republic/10251-1/2021_H5N8_HA_2021-05-16
EPI_ISL_1697190_A/peacock/Czech_Republic/6529-2/2021_H5N8_HA_2021-04-02
EPI_ISL_1114742_A/chicken/Kostroma/304-08/2020_H5N8_HA_2020-10-17
EPI_ISL_615073_A/domestic_goose/Kazakhstan/1-242_2-20-B/2020_H5N8_HA_2020-09-19
EPI_ISL_623074_A/chicken/Iraq/1/2020_H5N8_HA_2020-05-12
EPI_ISL_320958_A/chicken/Tatarstan/7/2018_H5N8_HA_2018-08-01
EPI_ISL_320679_A/chicken/Cheboksary/806/2018_H5N8_HA_2018-07-16
EPI_ISL_320662_A/chicken/Penza/300/2018_H5N8_HA_2018-06-15
EPI_ISL_320680_A/chicken/Kursk/757/2018_H5N8_HA_2018-06-20
EPI_ISL_320681_A/chicken/Kursk/760/2018_H5N8_HA_2018-06-26
EPI_ISL_320683_A/goose/Samara/673/2018_H5N8_HA_2018-07-04
EPI_ISL_320685_A/chicken/Samara/679/2018_H5N8_HA_2018-07-04
EPI_ISL_320677_A/chicken/Penza/607/2018_H5N8_HA_2018-06-19
EPI_ISL_320659_A/duck/Samara/452/2018_H5N8_HA_2018-06-22
EPI_ISL_336933_A/chicken/Voronezh/1513/2018_H5N8_HA_2018-11-02
EPI_ISL_336932_A/chicken/Voronezh/1504/2018_H5N8_HA_2018-11-02
EPI_ISL_268652_A/Eur_Wig/NL-Zoeterwoude/16015702-010/2016_H5N8_HA_2016-12-10
EPI_ISL_268799_A/Back-headed_Gull/Netherlands/8/2016_H5N8_HA_2016-12-20
EPI_ISL_269596_A/Eurasian_Wigeon/Netherlands/13/2016_H5N8_HA_2016-12-14
EPI_ISL_253036_A/turkey/England/003778/2017_H5N8_HA_2017-01-15
EPI_ISL_255912_A/Eurasian_Wigeon/Netherlands/4/2016_H5N8_HA_2016-12-09
EPI_ISL_269703_A/Eurasian_Wigeon/Netherlands/25/2016_H5N8_HA_2016-12-05
EPI_ISL_269603_A/Mallard/Netherlands/51/2016_H5N8_HA_2016-12-20
EPI_ISL_239434_A/tufted_duck/Switzerland/V246-L02001/2016_H5N8_HA_2016-11-01
EPI_ISL_268639_A/Eur_Wig/NL-Enumatil-Groningen/16015704-001/2016_H5N8_HA_2016-12-11
EPI_ISL_268638_A/Eur_Wig/NL-Drieborg_Dollard_/16015513-001/2016_H5N8_HA_2016-12-06
EPI_ISL_316539_A/turkey/Italy/17VIR8275-3/2017_H5N8_HA_2017-09-26
EPI_ISL_316538_A/turkey/Italy/17VIR8199-1/2017_H5N8_HA_2017-09-25
EPI_ISL_316582_A/turkey/Italy/17VIR10983-22/2017_H5N8_HA_2017-12-11
EPI_ISL_273846_A/chicken/Italy/17VIR3078/2017_H5N8_HA_2017-04-07
EPI_ISL_309195_A/Cygnus_olor/Belgium/2967/2017_H5N8_HA_2017-03-21
EPI_ISL_419344_A/chicken/Bulgaria/Dobrich/12-2/2018_H5N8_HA_2018-03-02
EPI_ISL_419359_A/chicken/Bulgaria/Dobrich/115/2018_H5N8_HA_2018-06-12
EPI_ISL_419361_A/chicken/Bulgaria/Dobrich/163-2/2018_H5N8_HA_2018-07-05
EPI_ISL_419362_A/chicken/Bulgaria/Haskovo/286/2018_H5N8_HA_2018-10-16
EPI_ISL_419345_A/chicken/Bulgaria/Plovdiv/224-1/2018_H5N8_HA_2018-09-19
EPI_ISL_300712_A/chicken/Poland/263/2017_H5N8_HA_2017-03-04
EPI_ISL_255933_A/Cormorant/Hungary/6102/2017_H5N8_HA_2017-02-15
EPI_ISL_255204_A/Mute_swan/Hungary/3542/2017_H5N8_HA_2017-01-29
EPI_ISL_366241_A/Cygnus_olor/England/WULH/2016_H5N8_HA_2016-12-30
EPI_ISL_366238_A/Cygnus_olor/England/WVJX/2016_H5N8_HA_2016-12-31
EPI_ISL_230820_A/great_crested_grebe/Tyva/34/2016_H5N8_HA_2016-05-25
EPI_ISL_206773_A/gull/Germany-NI/R45/2015_H5N8_HA_2014-12-01
EPI_ISL_331223_A/Duck/England/1279/2014_H5N8_HA_2014-11-16
EPI_ISL_169273_A/turkey/Germany/AR2485-86-L00899/2014_H5N8_HA_2014-11-04
EPI_ISL_174349_A/chicken/Netherlands/14015766/2014_H5N8_HA_2014-11-19
EPI_ISL_191765_A/scarlet_ibis/Germany/AR44-L01279/2015_H5N8_HA_2015-01-01
EPI_ISL_175535_A/MuteSwan/Sweden/SVA150313KU0141/SZ543/2015_H5N8_HA_2015-03-05
EPI_ISL_168075_A/chicken/Netherlands/14015531/2014_H5N8_HA_2014-11-15
EPI_ISL_170184_A/chicken/Germany-MV/R153/2015_H5N8_HA_2015-01-01
EPI_ISL_198665_A/turkey/Germany/AR3390-L00939/2014_H5N8_HA_2014-12-15
EPI_ISL_169350_A/turkey/Italy/14VIR7898-10/2014_H5N8_HA_2014-12-15
EPI_ISL_174351_A/duck/Netherlands/14015989/2014_H5N8_HA_2014-11-21
EPI_ISL_169281_A/eurasian_wigeon/Netherlands/emc-2/2014_H5N8_HA_2014-11-24
EPI_ISL_181094_A/eurasian_wigeon/Netherlands/2/2014_H5N8_HA_2014-11-24
1
2014
2015
2016
2017
2018
2019
2020
2021
2022

NA N8

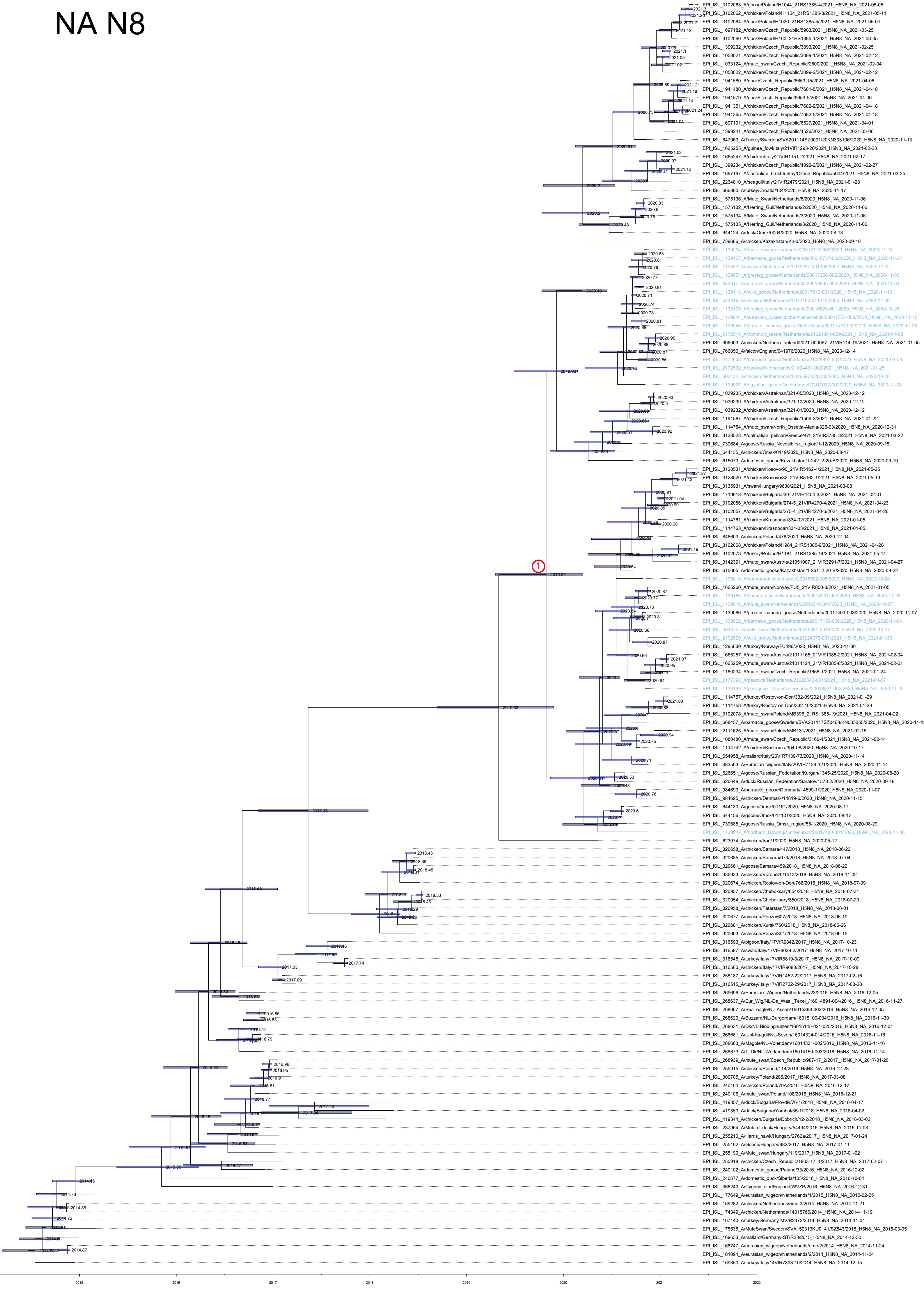

NA N1

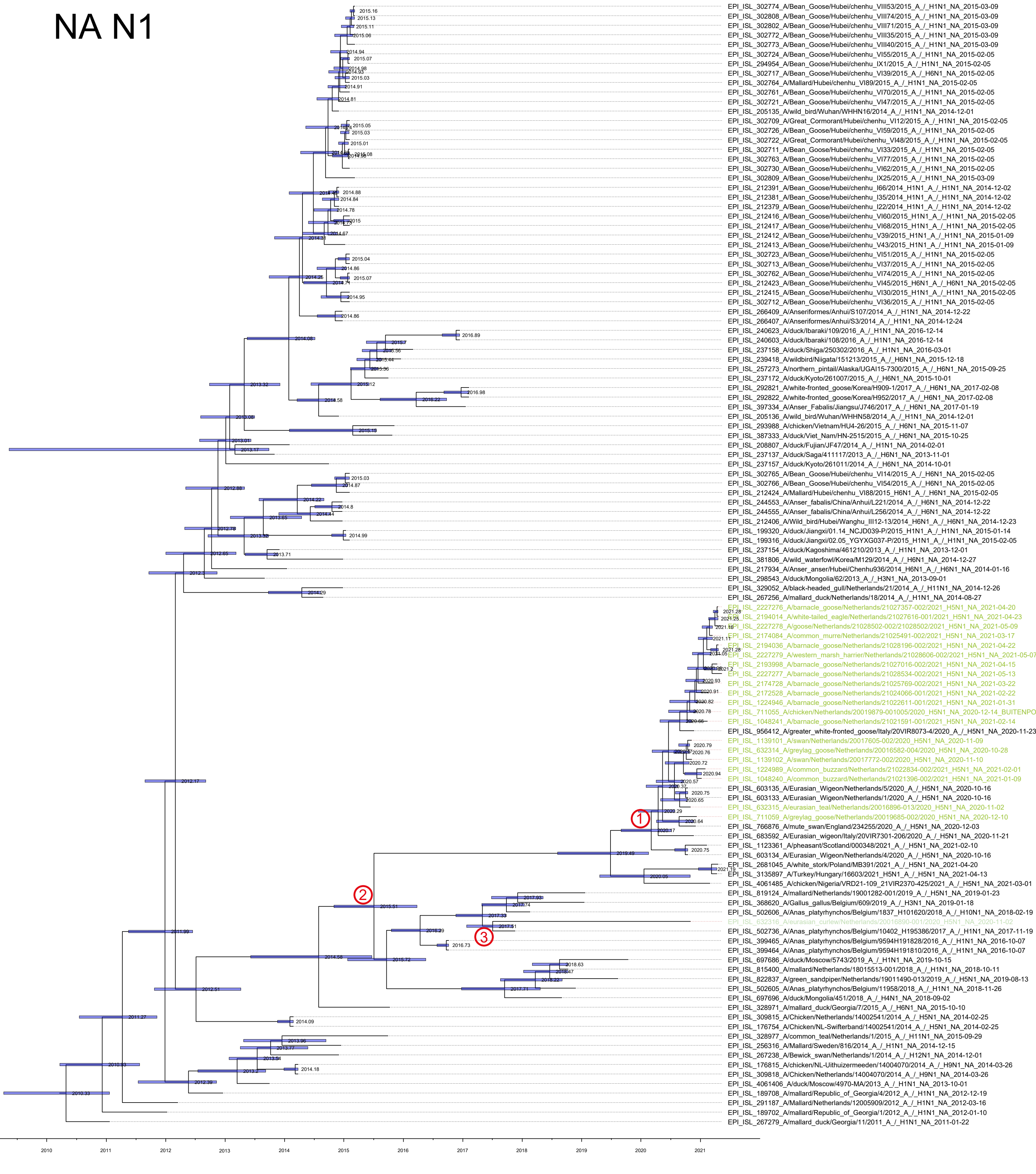
EPI_ISL_302774_A/Bean_Goose/Hubei/chenhu_VIII53/2015_A_/_H1N1_NA_2015-03-09
EPI_ISL_302808_A/Bean_Goose/Hubei/chenhu_VIII74/2015_A_/_H1N1_NA_2015-03-09
EPI_ISL_302802_A/Bean_Goose/Hubei/chenhu_VIII71/2015_A_/_H1N1_NA_2015-03-09
EPI_ISL_302772_A/Bean_Goose/Hubei/chenhu_VIII35/2015_A_/_H1N1_NA_2015-03-09
EPI_ISL_302773_A/Bean_Goose/Hubei/chenhu_VIII40/2015_A_/_H1N1_NA_2015-03-09
EPI_ISL_302724_A/Bean_Goose/Hubei/chenhu_VI55/2015_A_/_H1N1_NA_2015-02-05
EPI_ISL_294954_A/Bean_Goose/Hubei/chenhu_IX1/2015_A_/_H1N1_NA_2015-02-05
EPI_ISL_302717_A/Bean_Goose/Hubei/chenhu_VI39/2015_A_/_H6N1_NA_2015-02-05
EPI_ISL_302764_A/Mallard/Hubei/chenhu_VI89/2015_A_/_H1N1_NA_2015-02-05
EPI_ISL_302761_A/Bean_Goose/Hubei/chenhu_VI70/2015_A_/_H1N1_NA_2015-02-05
EPI_ISL_302721_A/Bean_Goose/Hubei/chenhu_VI47/2015_A_/_H1N1_NA_2015-02-05
EPI_ISL_205135_A/wild_bird/Wuhan/WHHN16/2014_A_/_H1N1_NA_2014-12-01
EPI_ISL_302709_A/Great_Cormorant/Hubei/chenhu_VI12/2015_A_/_H1N1_NA_2015-02-05
EPI_ISL_302726_A/Bean_Goose/Hubei/chenhu_VI59/2015_A_/_H1N1_NA_2015-02-05
EPI_ISL_302722_A/Great_Cormorant/Hubei/chenhu_VI48/2015_A_/_H1N1_NA_2015-02-05
EPI_ISL_302711_A/Bean_Goose/Hubei/chenhu_VI33/2015_A_/_H1N1_NA_2015-02-05
EPI_ISL_302763_A/Bean_Goose/Hubei/chenhu_VI77/2015_A_/_H1N1_NA_2015-02-05
EPI_ISL_302730_A/Bean_Goose/Hubei/chenhu_VI62/2015_A_/_H1N1_NA_2015-02-05
EPI_ISL_302809_A/Bean_Goose/Hubei/chenhu_IX25/2015_A_/_H1N1_NA_2015-03-09
EPI_ISL_212391_A/Bean_Goose/Hubei/chenhu_I66/2014_H1N1_A_/_H1N1_NA_2014-12-02
EPI_ISL_212381_A/Bean_Goose/Hubei/chenhu_I35/2014_H1N1_A_/_H1N1_NA_2014-12-02
EPI_ISL_212379_A/Bean_Goose/Hubei/chenhu_I22/2014_H1N1_A_/_H1N1_NA_2014-12-02
EPI_ISL_212416_A/Bean_Goose/Hubei/chenhu_VI60/2015_H1N1_A_/_H1N1_NA_2015-02-05
EPI_ISL_212417_A/Bean_Goose/Hubei/chenhu_VI68/2015_H1N1_A_/_H1N1_NA_2015-02-05
EPI_ISL_212412_A/Bean_Goose/Hubei/chenhu_V39/2015_H1N1_A_/_H1N1_NA_2015-01-09
EPI_ISL_212413_A/Bean_Goose/Hubei/chenhu_V43/2015_H1N1_A_/_H1N1_NA_2015-01-09
EPI_ISL_302723_A/Bean_Goose/Hubei/chenhu_VI51/2015_A_/_H1N1_NA_2015-02-05
EPI_ISL_302713_A/Bean_Goose/Hubei/chenhu_VI37/2015_A_/_H1N1_NA_2015-02-05
EPI_ISL_302762_A/Bean_Goose/Hubei/chenhu_VI74/2015_A_/_H1N1_NA_2015-02-05
EPI_ISL_212423_A/Bean_Goose/Hubei/chenhu_VI45/2015_H6N1_A_/_H6N1_NA_2015-02-05
EPI_ISL_212415_A/Bean_Goose/Hubei/chenhu_VI30/2015_H1N1_A_/_H1N1_NA_2015-02-05
EPI_ISL_302712_A/Bean_Goose/Hubei/chenhu_VI36/2015_A_/_H1N1_NA_2015-02-05
EPI_ISL_266409_A/Anseriformes/Anhui/S107/2014_A_/_H1N1_NA_2014-12-22
EPI_ISL_266407_A/Anseriformes/Anhui/S3/2014_A_/_H1N1_NA_2014-12-24
EPI_ISL_240623_A/duck/Ibaraki/109/2016_A_/_H1N1_NA_2016-12-14
EPI_ISL_240603_A/duck/Ibaraki/108/2016_A_/_H1N1_NA_2016-12-14
EPI_ISL_237158_A/duck/Shiga/250302/2016_A_/_H1N1_NA_2016-03-01
EPI_ISL_239418_A/wildbird/Niigata/151213/2015_A_/_H6N1_NA_2015-12-18
EPI_ISL_257273_A/northern_pintail/Alaska/UGAI15-7300/2015_A_/_H6N1_NA_2015-09-25
EPI_ISL_237172_A/duck/Kyoto/261007/2015_A_/_H6N1_NA_2015-10-01
EPI_ISL_292821_A/white-fronted_goose/Korea/H909-1/2017_A_/_H6N1_NA_2017-02-08
EPI_ISL_292822_A/white-fronted_goose/Korea/H952/2017_A_/_H6N1_NA_2017-02-08
EPI_ISL_397334_A/Anser_Fabalis/Jiangsu/J746/2017_A_/_H6N1_NA_2017-01-19
EPI_ISL_205136_A/wild_bird/Wuhan/WHHN58/2014_A_/_H1N1_NA_2014-12-01
EPI_ISL_293988_A/chicken/Vietnam/HU4-26/2015_A_/_H6N1_NA_2015-11-07
EPI_ISL_387333_A/duck/Viet_Nam/HN-2515/2015_A_/_H6N1_NA_2015-10-25
EPI_ISL_208807_A/duck/Fujian/JF47/2014_A_/_H1N1_NA_2014-02-01
EPI_ISL_237137_A/duck/Saga/411117/2013_A_/_H6N1_NA_2013-11-01
EPI_ISL_237157_A/duck/Kyoto/261011/2014_A_/_H6N1_NA_2014-10-01
EPI_ISL_302765_A/Bean_Goose/Hubei/chenhu_VI14/2015_A_/_H6N1_NA_2015-02-05
EPI_ISL_302766_A/Bean_Goose/Hubei/chenhu_VI54/2015_A_/_H6N1_NA_2015-02-05
EPI_ISL_212424_A/Mallard/Hubei/chenhu_VI88/2015_H6N1_A_/_H6N1_NA_2015-02-05
EPI_ISL_244553_A/Anser_fabalis/China/Anhui/L221/2014_A_/_H6N1_NA_2014-12-22
EPI_ISL_244555_A/Anser_fabalis/China/Anhui/L256/2014_A_/_H6N1_NA_2014-12-22
EPI_ISL_212406_A/Wild_bird/Hubei/Wanghu_III12-13/2014_H6N1_A_/_H6N1_NA_2014-12-23
EPI_ISL_199320_A/duck/Jiangxi/01.14_NCJD039-P/2015_H1N1_A_/_H1N1_NA_2015-01-14
EPI_ISL_199316_A/duck/Jiangxi/02.05_YGYXG037-P/2015_H1N1_A_/_H1N1_NA_2015-02-05
EPI_ISL_237154_A/duck/Kagoshima/461210/2013_A_/_H1N1_NA_2013-12-01
EPI_ISL_381806_A/wild_waterfowl/Korea/M129/2014_A_/_H6N1_NA_2014-12-27
EPI_ISL_217934_A/Anser_anser/Hubei/Chenhu936/2014_H6N1_A_/_H6N1_NA_2014-01-16
EPI_ISL_298543_A/duck/Mongolia/62/2013_A_/_H3N1_NA_2013-09-01
EPI_ISL_329052_A/black-headed_gull/Netherlands/21/2014_A_/_H11N1_NA_2014-12-26
EPI_ISL_267256_A/mallard_duck/Netherlands/18/2014_A_/_H1N1_NA_2014-08-27
EPI_ISL_2227276_A/barnacle_goose/Netherlands/21027357-002/2021_H5N1_NA_2021-04-20
EPI_ISL_2194014_A/white-tailed_eagle/Netherlands/21027616-001/2021_H5N1_NA_2021-04-23
EPI_ISL_2227278_A/goose/Netherlands/21028502-002/21028502/2021_H5N1_NA_2021-05-09
EPI_ISL_2174084_A/common_murre/Netherlands/21025491-002/2021_H5N1_NA_2021-03-17
EPI_ISL_2194036_A/barnacle_goose/Netherlands/21028196-002/2021_H5N1_NA_2021-04-22
EPI_ISL_2227279_A/western_marsh_harrier/Netherlands/21028606-002/2021_H5N1_NA_2021-05-07
EPI_ISL_2193998_A/barnacle_goose/Netherlands/21027016-002/2021_H5N1_NA_2021-04-15
EPI_ISL_2227277_A/barnacle_goose/Netherlands/21028534-002/2021_H5N1_NA_2021-05-13
EPI_ISL_2174728_A/barnacle_goose/Netherlands/21025769-002/2021_H5N1_NA_2021-03-22
EPI_ISL_2172528_A/barnacle_goose/Netherlands/21024066-001/2021_H5N1_NA_2021-02-22
EPI_ISL_1224946_A/barnacle_goose/Netherlands/21022611-001/2021_H5N1_NA_2021-01-31
EPI_ISL_711055_A/chicken/Netherlands/20019879-001005/2020_H5N1_NA_2020-12-14_BUITENPO
EPI_ISL_1048241_A/barnacle_goose/Netherlands/21021591-001/2021_H5N1_NA_2021-02-14
EPI_ISL_956412_A/greater_white-fronted_goose/Italy/20VIR8073-4/2020_A_/_H5N1_NA_2020-11-23
EPI_ISL_1139101_A/swan/Netherlands/20017605-002/2020_H5N1_NA_2020-11-09
EPI_ISL_632314_A/greylag_goose/Netherlands/20016582-004/2020_H5N1_NA_2020-10-28
EPI_ISL_1139102_A/swan/Netherlands/20017772-002/2020_H5N1_NA_2020-11-10
EPI_ISL_1224989_A/common_buzzard/Netherlands/21022834-002/2021_H5N1_NA_2021-02-01
EPI_ISL_1048240_A/common_buzzard/Netherlands/21021396-002/2021_H5N1_NA_2021-01-09
EPI_ISL_603135_A/Eurasian_Wigeon/Netherlands/5/2020_A_/_H5N1_NA_2020-10-16
EPI_ISL_603133_A/Eurasian_Wigeon/Netherlands/1/2020_A_/_H5N1_NA_2020-10-16
EPI_ISL_632315_A/eurasian_teal/Netherlands/20016896-013/2020_H5N1_NA_2020-11-02
EPI_ISL_711059_A/greylag_goose/Netherlands/20019685-002/2020_H5N1_NA_2020-12-10
EPI_ISL_766876_A/mute_swan/England/234255/2020_A_/_H5N1_NA_2020-12-03
EPI_ISL_683592_A/Eurasian_wigeon/Italy/20VIR7301-206/2020_A_/_H5N1_NA_2020-11-21
EPI_ISL_1123361_A/pheasant/Scotland/000348/2021_A_/_H5N1_NA_2021-02-10
EPI_ISL_603134_A/Eurasian_Wigeon/Netherlands/4/2020_A_/_H5N1_NA_2020-10-16
EPI_ISL_2681045_A/white_stork/Poland/MB391/2021_A_/_H5N1_NA_2021-04-20
EPI_ISL_3135897_A/Turkey/Hungary/16603/2021_H5N1_A_/_H5N1_NA_2021-04-13
EPI_ISL_4061485_A/chicken/Nigeria/VRD21-109_21VIR2370-425/2021_A_/_H5N1_NA_2021-03-01
EPI_ISL_819124_A/mallard/Netherlands/19001282-001/2019_A_/_H5N1_NA_2019-01-23
EPI_ISL_368620_A/Gallus_gallus/Belgium/609/2019_A_/_H3N1_NA_2019-01-18
EPI_ISL_502606_A/Anas_platyrhynchos/Belgium/1837_H101620/2018_A_/_H10N1_NA_2018-02-19
EPI_ISL_632316_A/eurasian_curlew/Netherlands/20016890-001/2020_H5N1_NA_2020-11-02
EPI_ISL_502736_A/Anas_platyrhynchos/Belgium/10402_H195386/2017_A_/_H1N1_NA_2017-11-19
EPI_ISL_399465_A/Anas_platyrhynchos/Belgium/9594H191828/2016_A_/_H1N1_NA_2016-10-07
EPI_ISL_399464_A/Anas_platyrhynchos/Belgium/9594H191810/2016_A_/_H1N1_NA_2016-10-07
EPI_ISL_697686_A/duck/Moscow/5743/2019_A_/_H1N1_NA_2019-10-15
EPI_ISL_815400_A/mallard/Netherlands/18015513-001/2018_A_/_H1N1_NA_2018-10-11
EPI_ISL_822837_A/green_sandpiper/Netherlands/19011490-013/2019_A_/_H5N1_NA_2019-08-13
EPI_ISL_502605_A/Anas_platyrhynchos/Belgium/11958/2018_A_/_H1N1_NA_2018-11-26
EPI_ISL_697696_A/duck/Mongolia/451/2018_A_/_H4N1_NA_2018-09-02
EPI_ISL_328971_A/mallard_duck/Georgia/7/2015_A_/_H6N1_NA_2015-10-10
EPI_ISL_309815_A/Chicken/Netherlands/14002541/2014_A_/_H5N1_NA_2014-02-25
EPI_ISL_176754_A/Chicken/NL-Swifterband/14002541/2014_A_/_H5N1_NA_2014-02-25
EPI_ISL_328977_A/common_teal/Netherlands/1/2015_A_/_H11N1_NA_2015-09-29
EPI_ISL_256316_A/Mallard/Sweden/816/2014_A_/_H1N1_NA_2014-12-15
EPI_ISL_267238_A/Bewick_swan/Netherlands/1/2014_A_/_H12N1_NA_2014-12-01
EPI_ISL_176815_A/chicken/NL-Uithuizermeeden/14004070/2014_A_/_H9N1_NA_2014-03-26
EPI_ISL_309818_A/Chicken/Netherlands/14004070/2014_A_/_H9N1_NA_2014-03-26
EPI_ISL_4061406_A/duck/Moscow/4970-MA/2013_A_/_H1N1_NA_2013-10-01
EPI_ISL_189708_A/Mallard/Republic_of_Georgia/4/2012_A_/_H1N1_NA_2012-12-19
EPI_ISL_291187_A/Mallard/Netherlands/12005909/2012_A_/_H1N1_NA_2012-03-16
EPI_ISL_189702_A/Mallard/Republic_of_Georgia/1/2012_A_/_H1N1_NA_2012-01-10
EPI_ISL_267279_A/mallard_duck/Georgia/11/2011_A_/_H1N1_NA_2011-01-22
1
2
3
2010
2011
2012
2013
2014
2015
2016
2017
2018
2019
2020
2021

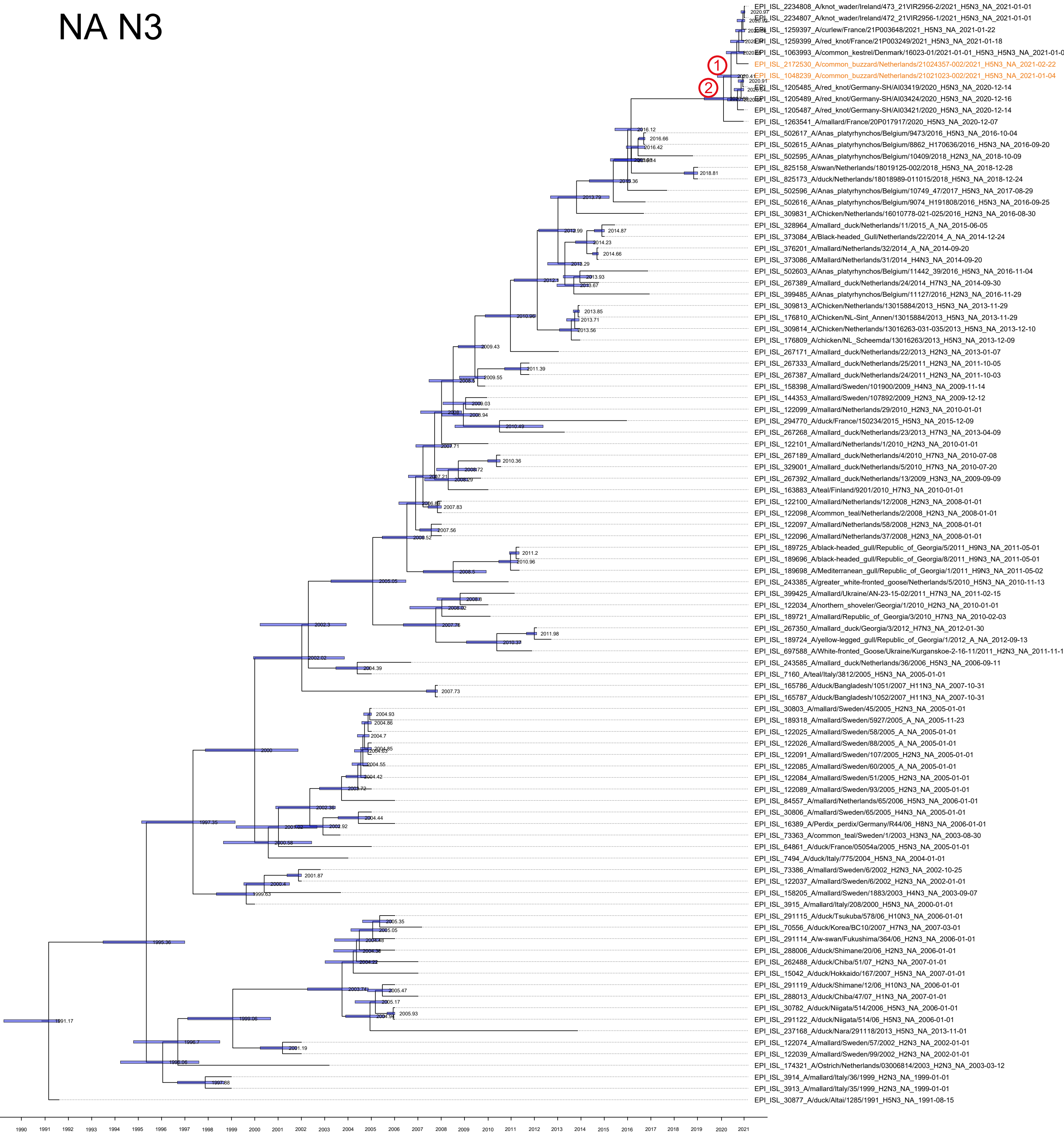
NA N3
1
2
EPI_ISL_2234808_A/knot_wader/Ireland/473_21VIR2956-2/2021_H5N3_NA_2021-01-01
EPI_ISL_2234807_A/knot_wader/Ireland/472_21VIR2956-1/2021_H5N3_NA_2021-01-01
EPI_ISL_1259397_A/curlew/France/21P003648/2021_H5N3_NA_2021-01-22
EPI_ISL_1259399_A/red_knot/France/21P003249/2021_H5N3_NA_2021-01-18
EPI_ISL_1063993_A/common_kestrel/Denmark/16023-01/2021-01-01_H5N3_H5N3_NA_2021-01-0
EPI_ISL_2172530_A/common_buzzard/Netherlands/21024357-002/2021_H5N3_NA_2021-02-22
EPI_ISL_1048239_A/common_buzzard/Netherlands/21021023-002/2021_H5N3_NA_2021-01-04
EPI_ISL_1205485_A/red_knot/Germany-SH/AI03419/2020_H5N3_NA_2020-12-14
EPI_ISL_1205489_A/red_knot/Germany-SH/AI03424/2020_H5N3_NA_2020-12-16
EPI_ISL_1205487_A/red_knot/Germany-SH/AI03421/2020_H5N3_NA_2020-12-14
EPI_ISL_1263541_A/mallard/France/20P017917/2020_H5N3_NA_2020-12-07
EPI_ISL_502617_A/Anas_platyrhynchos/Belgium/9473/2016_H5N3_NA_2016-10-04
EPI_ISL_502615_A/Anas_platyrhynchos/Belgium/8862_H170636/2016_H5N3_NA_2016-09-20
EPI_ISL_502595_A/Anas_platyrhynchos/Belgium/10409/2018_H2N3_NA_2018-10-09
EPI_ISL_825158_A/swan/Netherlands/18019125-002/2018_H5N3_NA_2018-12-28
EPI_ISL_825173_A/duck/Netherlands/18018989-011015/2018_H5N3_NA_2018-12-24
EPI_ISL_502596_A/Anas_platyrhynchos/Belgium/10749_47/2017_H5N3_NA_2017-08-29
EPI_ISL_502616_A/Anas_platyrhynchos/Belgium/9074_H191808/2016_H5N3_NA_2016-09-25
EPI_ISL_309831_A/Chicken/Netherlands/16010778-021-025/2016_H2N3_NA_2016-08-30
EPI_ISL_328964_A/mallard_duck/Netherlands/11/2015_A_NA_2015-06-05
EPI_ISL_373084_A/Black-headed_Gull/Netherlands/22/2014_A_NA_2014-12-24
EPI_ISL_376201_A/mallard/Netherlands/32/2014_A_NA_2014-09-20
EPI_ISL_373086_A/Mallard/Netherlands/31/2014_H4N3_NA_2014-09-20
EPI_ISL_502603_A/Anas_platyrhynchos/Belgium/11442_39/2016_H5N3_NA_2016-11-04
EPI_ISL_267389_A/mallard_duck/Netherlands/24/2014_H7N3_NA_2014-09-30
EPI_ISL_399485_A/Anas_platyrhynchos/Belgium/11127/2016_H2N3_NA_2016-11-29
EPI_ISL_309813_A/Chicken/Netherlands/13015884/2013_H5N3_NA_2013-11-29
EPI_ISL_176810_A/Chicken/NL-Sint_Annen/13015884/2013_H5N3_NA_2013-11-29
EPI_ISL_309814_A/Chicken/Netherlands/13016263-031-035/2013_H5N3_NA_2013-12-10
EPI_ISL_176809_A/chicken/NL_Scheemda/13016263/2013_H5N3_NA_2013-12-09
EPI_ISL_267171_A/mallard_duck/Netherlands/22/2013_H2N3_NA_2013-01-07
EPI_ISL_267333_A/mallard_duck/Netherlands/25/2011_H2N3_NA_2011-10-05
EPI_ISL_267387_A/mallard_duck/Netherlands/24/2011_H2N3_NA_2011-10-03
EPI_ISL_158398_A/mallard/Sweden/101900/2009_H4N3_NA_2009-11-14
EPI_ISL_144353_A/mallard/Sweden/107892/2009_H2N3_NA_2009-12-12
EPI_ISL_122099_A/mallard/Netherlands/29/2010_H2N3_NA_2010-01-01
EPI_ISL_294770_A/duck/France/150234/2015_H5N3_NA_2015-12-09
EPI_ISL_267268_A/mallard_duck/Netherlands/23/2013_H7N3_NA_2013-04-09
EPI_ISL_122101_A/mallard/Netherlands/1/2010_H2N3_NA_2010-01-01
EPI_ISL_267189_A/mallard_duck/Netherlands/4/2010_H7N3_NA_2010-07-08
EPI_ISL_329001_A/mallard_duck/Netherlands/5/2010_H7N3_NA_2010-07-20
EPI_ISL_267392_A/mallard_duck/Netherlands/13/2009_H3N3_NA_2009-09-09
EPI_ISL_163883_A/teal/Finland/9201/2010_H7N3_NA_2010-01-01
EPI_ISL_122100_A/mallard/Netherlands/12/2008_H2N3_NA_2008-01-01
EPI_ISL_122098_A/common_teal/Netherlands/2/2008_H2N3_NA_2008-01-01
EPI_ISL_122097_A/mallard/Netherlands/58/2008_H2N3_NA_2008-01-01
EPI_ISL_122096_A/mallard/Netherlands/37/2008_H2N3_NA_2008-01-01
EPI_ISL_189725_A/black-headed_gull/Republic_of_Georgia/5/2011_H9N3_NA_2011-05-01
EPI_ISL_189696_A/black-headed_gull/Republic_of_Georgia/8/2011_H9N3_NA_2011-05-01
EPI_ISL_189698_A/Mediterranean_gull/Republic_of_Georgia/1/2011_H9N3_NA_2011-05-02
EPI_ISL_243385_A/greater_white-fronted_goose/Netherlands/5/2010_H5N3_NA_2010-11-13
EPI_ISL_399425_A/mallard/Ukraine/AN-23-15-02/2011_H7N3_NA_2011-02-15
EPI_ISL_122034_A/northern_shoveler/Georgia/1/2010_H2N3_NA_2010-01-01
EPI_ISL_189721_A/mallard/Republic_of_Georgia/3/2010_H7N3_NA_2010-02-03
EPI_ISL_267350_A/mallard_duck/Georgia/3/2012_H7N3_NA_2012-01-30
EPI_ISL_189724_A/yellow-legged_gull/Republic_of_Georgia/1/2012_A_NA_2012-09-13
EPI_ISL_697588_A/White-fronted_Goose/Ukraine/Kurganskoe-2-16-11/2011_H2N3_NA_2011-11-1
EPI_ISL_243585_A/mallard_duck/Netherlands/36/2006_H5N3_NA_2006-09-11
EPI_ISL_7160_A/teal/Italy/3812/2005_H5N3_NA_2005-01-01
EPI_ISL_165786_A/duck/Bangladesh/1051/2007_H11N3_NA_2007-10-31
EPI_ISL_165787_A/duck/Bangladesh/1052/2007_H11N3_NA_2007-10-31
EPI_ISL_30803_A/mallard/Sweden/45/2005_H2N3_NA_2005-01-01
EPI_ISL_189318_A/mallard/Sweden/5927/2005_A_NA_2005-11-23
EPI_ISL_122025_A/mallard/Sweden/58/2005_A_NA_2005-01-01
EPI_ISL_122026_A/mallard/Sweden/88/2005_A_NA_2005-01-01
EPI_ISL_122091_A/mallard/Sweden/107/2005_H2N3_NA_2005-01-01
EPI_ISL_122085_A/mallard/Sweden/60/2005_A_NA_2005-01-01
EPI_ISL_122084_A/mallard/Sweden/51/2005_H2N3_NA_2005-01-01
EPI_ISL_122089_A/mallard/Sweden/93/2005_H2N3_NA_2005-01-01
EPI_ISL_84557_A/mallard/Netherlands/65/2006_H5N3_NA_2006-01-01
EPI_ISL_30806_A/mallard/Sweden/65/2005_H4N3_NA_2005-01-01
EPI_ISL_16389_A/Perdix_perdix/Germany/R44/06_H8N3_NA_2006-01-01
EPI_ISL_73363_A/common_teal/Sweden/1/2003_H3N3_NA_2003-08-30
EPI_ISL_64861_A/duck/France/05054a/2005_H5N3_NA_2005-01-01
EPI_ISL_7494_A/duck/Italy/775/2004_H5N3_NA_2004-01-01
EPI_ISL_73386_A/mallard/Sweden/6/2002_H2N3_NA_2002-10-25
EPI_ISL_122037_A/mallard/Sweden/6/2002_H2N3_NA_2002-01-01
EPI_ISL_158205_A/mallard/Sweden/1883/2003_H4N3_NA_2003-09-07
EPI_ISL_3915_A/mallard/Italy/208/2000_H5N3_NA_2000-01-01
EPI_ISL_291115_A/duck/Tsukuba/578/06_H10N3_NA_2006-01-01
EPI_ISL_70556_A/duck/Korea/BC10/2007_H7N3_NA_2007-03-01
EPI_ISL_291114_A/w-swan/Fukushima/364/06_H2N3_NA_2006-01-01
EPI_ISL_288006_A/duck/Shimane/20/06_H2N3_NA_2006-01-01
EPI_ISL_262488_A/duck/Chiba/51/07_H2N3_NA_2007-01-01
EPI_ISL_15042_A/duck/Hokkaido/167/2007_H5N3_NA_2007-01-01
EPI_ISL_291119_A/duck/Shimane/12/06_H10N3_NA_2006-01-01
EPI_ISL_288013_A/duck/Chiba/47/07_H1N3_NA_2007-01-01
EPI_ISL_30782_A/duck/Niigata/514/2006_H5N3_NA_2006-01-01
EPI_ISL_291122_A/duck/Niigata/514/06_H5N3_NA_2006-01-01
EPI_ISL_237168_A/duck/Nara/291118/2013_H5N3_NA_2013-11-01
EPI_ISL_122074_A/mallard/Sweden/57/2002_H2N3_NA_2002-01-01
EPI_ISL_122039_A/mallard/Sweden/99/2002_H2N3_NA_2002-01-01
EPI_ISL_174321_A/Ostrich/Netherlands/03006814/2003_H2N3_NA_2003-03-12
EPI_ISL_3914_A/mallard/Italy/36/1999_H2N3_NA_1999-01-01
EPI_ISL_3913_A/mallard/Italy/35/1999_H2N3_NA_1999-01-01
EPI_ISL_30877_A/duck/Altai/1285/1991_H5N3_NA_1991-08-15
1990 1991 1992 1993 1994 1995 1996 1997 1998 1999 2000 2001 2002 2003 2004 2005 2006 2007 2008 2009 2010 2011 2012 2013 2014 2015 2016 2017 2018 2019 2020 2021

NA N4

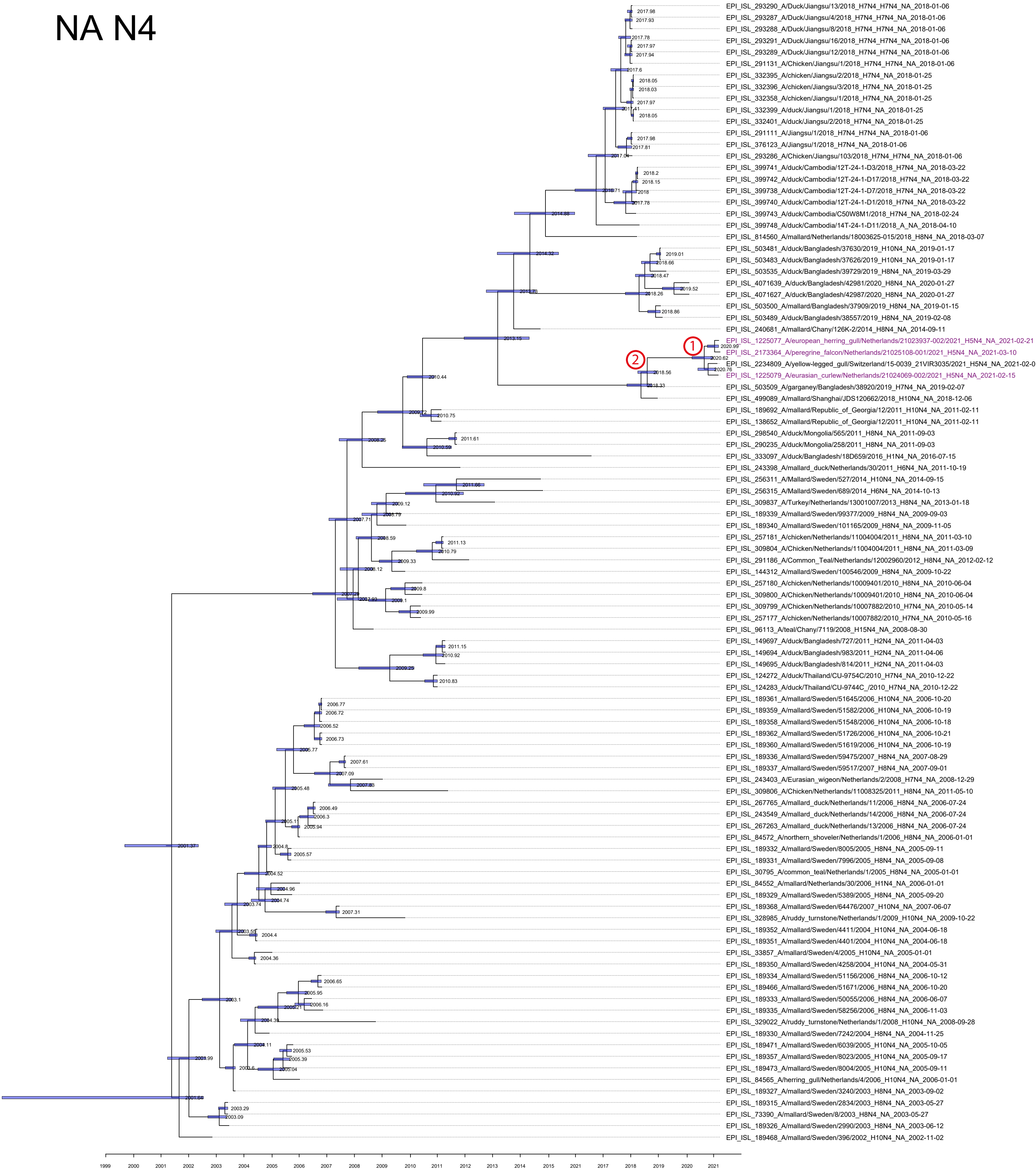

EPI_ISL_293290_A/Duck/Jiangsu/13/2018_H7N4_H7N4_NA_2018-01-06
EPI_ISL_293287_A/Duck/Jiangsu/4/2018_H7N4_NA_2018-01-06
EPI_ISL_293288_A/Duck/Jiangsu/8/2018_H7N4_H7N4_NA_2018-01-06
EPI_ISL_293291_A/Duck/Jiangsu/16/2018_H7N4_H7N4_NA_2018-01-06
EPI_ISL_293289_A/Duck/Jiangsu/12/2018_H7N4_H7N4_NA_2018-01-06
EPI_ISL_291131_A/Chicken/Jiangsu/1/2018_H7N4_H7N4_NA_2018-01-06
EPI_ISL_332395_A/chicken/Jiangsu/2/2018_H7N4_NA_2018-01-25
EPI_ISL_332396_A/chicken/Jiangsu/3/2018_H7N4_NA_2018-01-25
EPI_ISL_332358_A/chicken/Jiangsu/1/2018_H7N4_NA_2018-01-25
EPI_ISL_332399_A/duck/Jiangsu/1/2018_H7N4_NA_2018-01-25
EPI_ISL_332401_A/duck/Jiangsu/2/2018_H7N4_NA_2018-01-25
EPI_ISL_291111_A/Jiangsu/1/2018_H7N4_H7N4_NA_2018-01-06
EPI_ISL_376123_A/Jiangsu/1/2018_H7N4_NA_2018-01-06
EPI_ISL_293286_A/Chicken/Jiangsu/103/2018_H7N4_H7N4_NA_2018-01-06
EPI_ISL_399741_A/duck/Cambodia/12T-24-1-D3/2018_H7N4_NA_2018-03-22
EPI_ISL_399742_A/duck/Cambodia/12T-24-1-D17/2018_H7N4_NA_2018-03-22
EPI_ISL_399738_A/duck/Cambodia/12T-24-1-D7/2018_H7N4_NA_2018-03-22
EPI_ISL_399740_A/duck/Cambodia/12T-24-1-D1/2018_H7N4_NA_2018-03-22
EPI_ISL_399743_A/duck/Cambodia/C50W8M1/2018_H7N4_NA_2018-02-24
EPI_ISL_399748_A/duck/Cambodia/14T-24-1-D11/2018_A_NA_2018-04-10
EPI_ISL_814560_A/mallard/Netherlands/18003625-015/2018_H8N4_NA_2018-03-07
EPI_ISL_503481_A/duck/Bangladesh/37630/2019_H10N4_NA_2019-01-17
EPI_ISL_503483_A/duck/Bangladesh/37626/2019_H10N4_NA_2019-01-17
EPI_ISL_503535_A/duck/Bangladesh/39729/2019_H8N4_NA_2019-03-29
EPI_ISL_4071639_A/duck/Bangladesh/42981/2020_H8N4_NA_2020-01-27
EPI_ISL_4071627_A/duck/Bangladesh/42987/2020_H8N4_NA_2020-01-27
EPI_ISL_503500_A/mallard/Bangladesh/37909/2019_H8N4_NA_2019-01-15
EPI_ISL_503489_A/duck/Bangladesh/38557/2019_H8N4_NA_2019-02-08
EPI_ISL_240681_A/mallard/Chany/126K-2/2014_H8N4_NA_2014-09-11
EPI_ISL_1225077_A/european_herring_gull/Netherlands/21023937-002/2021_H5N4_NA_2021-02-21
EPI_ISL_2173364_A/peregrine_falcon/Netherlands/21025108-001/2021_H5N4_NA_2021-03-10
EPI_ISL_2234809_A/yellow-legged_gull/Switzerland/15-0039_21VIR3035/2021_H5N4_NA_2021-02-0
EPI_ISL_1225079_A/eurasian_curlew/Netherlands/21024069-002/2021_H5N4_NA_2021-02-15
EPI_ISL_503509_A/garganey/Bangladesh/38920/2019_H7N4_NA_2019-02-07
EPI_ISL_499089_A/mallard/Shanghai/JDS120662/2018_H10N4_NA_2018-12-06
EPI_ISL_189692_A/mallard/Republic_of_Georgia/12/2011_H10N4_NA_2011-02-11
EPI_ISL_138652_A/mallard/Republic_of_Georgia/12/2011_H10N4_NA_2011-02-11
EPI_ISL_298540_A/duck/Mongolia/565/2011_H8N4_NA_2011-09-03
EPI_ISL_290235_A/duck/Mongolia/258/2011_H8N4_NA_2011-09-03
EPI_ISL_333097_A/duck/Bangladesh/18D659/2016_H1N4_NA_2016-07-15
EPI_ISL_243398_A/mallard_duck/Netherlands/30/2011_H6N4_NA_2011-10-19
EPI_ISL_256311_A/Mallard/Sweden/527/2014_H10N4_NA_2014-09-15
EPI_ISL_256315_A/Mallard/Sweden/689/2014_H6N4_NA_2014-10-13
EPI_ISL_309837_A/Turkey/Netherlands/13001007/2013_H8N4_NA_2013-01-18
EPI_ISL_189339_A/mallard/Sweden/99377/2009_H8N4_NA_2009-09-03
EPI_ISL_189340_A/mallard/Sweden/101165/2009_H8N4_NA_2009-11-05
EPI_ISL_257181_A/chicken/Netherlands/11004004/2011_H8N4_NA_2011-03-10
EPI_ISL_309804_A/Chicken/Netherlands/11004004/2011_H8N4_NA_2011-03-09
EPI_ISL_291186_A/Common_Teal/Netherlands/12002960/2012_H8N4_NA_2012-02-12
EPI_ISL_144312_A/mallard/Sweden/100546/2009_H8N4_NA_2009-10-22
EPI_ISL_257180_A/chicken/Netherlands/10009401/2010_H8N4_NA_2010-06-04
EPI_ISL_309800_A/Chicken/Netherlands/10009401/2010_H8N4_NA_2010-06-04
EPI_ISL_309799_A/Chicken/Netherlands/10007882/2010_H7N4_NA_2010-05-14
EPI_ISL_257177_A/chicken/Netherlands/10007882/2010_H7N4_NA_2010-05-16
EPI_ISL_96113_A/teal/Chany/7119/2008_H15N4_NA_2008-08-30
EPI_ISL_149697_A/duck/Bangladesh/727/2011_H2N4_NA_2011-04-03
EPI_ISL_149694_A/duck/Bangladesh/983/2011_H2N4_NA_2011-04-06
EPI_ISL_149695_A/duck/Bangladesh/814/2011_H2N4_NA_2011-04-03
EPI_ISL_124272_A/duck/Thailand/CU-9754C/2010_H7N4_NA_2010-12-22
EPI_ISL_124283_A/duck/Thailand/CU-9744C_/2010_H7N4_NA_2010-12-22
EPI_ISL_189361_A/mallard/Sweden/51645/2006_H10N4_NA_2006-10-20
EPI_ISL_189359_A/mallard/Sweden/51582/2006_H10N4_NA_2006-10-19
EPI_ISL_189358_A/mallard/Sweden/51548/2006_H10N4_NA_2006-10-18
EPI_ISL_189362_A/mallard/Sweden/51726/2006_H10N4_NA_2006-10-21
EPI_ISL_189360_A/mallard/Sweden/51619/2006_H10N4_NA_2006-10-19
EPI_ISL_189336_A/mallard/Sweden/59475/2007_H8N4_NA_2007-08-29
EPI_ISL_189337_A/mallard/Sweden/59517/2007_H8N4_NA_2007-09-01
EPI_ISL_243403_A/Eurasian_wigeon/Netherlands/2/2008_H7N4_NA_2008-12-29
EPI_ISL_309806_A/Chicken/Netherlands/11008325/2011_H8N4_NA_2011-05-10
EPI_ISL_267765_A/mallard_duck/Netherlands/11/2006_H8N4_NA_2006-07-24
EPI_ISL_243549_A/mallard_duck/Netherlands/14/2006_H8N4_NA_2006-07-24
EPI_ISL_267263_A/mallard_duck/Netherlands/13/2006_H8N4_NA_2006-07-24
EPI_ISL_84572_A/northern_shoveler/Netherlands/1/2006_H8N4_NA_2006-01-01
EPI_ISL_189332_A/mallard/Sweden/8005/2005_H8N4_NA_2005-09-11
EPI_ISL_189331_A/mallard/Sweden/7996/2005_H8N4_NA_2005-09-08
EPI_ISL_30795_A/common_teal/Netherlands/1/2005_H8N4_NA_2005-01-01
EPI_ISL_84552_A/mallard/Netherlands/30/2006_H1N4_NA_2006-01-01
EPI_ISL_189329_A/mallard/Sweden/5389/2005_H8N4_NA_2005-09-20
EPI_ISL_189368_A/mallard/Sweden/64476/2007_H10N4_NA_2007-06-07
EPI_ISL_328985_A/ruddy_turnstone/Netherlands/1/2009_H10N4_NA_2009-10-22
EPI_ISL_189352_A/mallard/Sweden/4411/2004_H10N4_NA_2004-06-18
EPI_ISL_189351_A/mallard/Sweden/4401/2004_H10N4_NA_2004-06-18
EPI_ISL_33857_A/mallard/Sweden/4/2005_H10N4_NA_2005-01-01
EPI_ISL_189350_A/mallard/Sweden/4258/2004_H10N4_NA_2004-05-31
EPI_ISL_189334_A/mallard/Sweden/51156/2006_H8N4_NA_2006-10-12
EPI_ISL_189466_A/mallard/Sweden/51671/2006_H8N4_NA_2006-10-20
EPI_ISL_189333_A/mallard/Sweden/50055/2006_H8N4_NA_2006-06-07
EPI_ISL_189335_A/mallard/Sweden/58256/2006_H8N4_NA_2006-11-03
EPI_ISL_329022_A/ruddy_turnstone/Netherlands/1/2008_H10N4_NA_2008-09-28
EPI_ISL_189330_A/mallard/Sweden/7242/2004_H8N4_NA_2004-11-25
EPI_ISL_189471_A/mallard/Sweden/6039/2005_H10N4_NA_2005-10-05
EPI_ISL_189357_A/mallard/Sweden/8023/2005_H10N4_NA_2005-09-17
EPI_ISL_189473_A/mallard/Sweden/8004/2005_H10N4_NA_2005-09-11
EPI_ISL_84565_A/herring_gull/Netherlands/4/2006_H10N4_NA_2006-01-01
EPI_ISL_189327_A/mallard/Sweden/3240/2003_H8N4_NA_2003-09-02
EPI_ISL_189315_A/mallard/Sweden/2834/2003_H8N4_NA_2003-05-27
EPI_ISL_73390_A/mallard/Sweden/8/2003_H8N4_NA_2003-05-27
EPI_ISL_189326_A/mallard/Sweden/2990/2003_H8N4_NA_2003-06-12
EPI_ISL_189468_A/mallard/Sweden/396/2002_H10N4_NA_2002-11-02
1999
2000
2001
2002
2003
2004
2005
2006
2007
2008
2009
2010
2011
2012
2013
2014
2015
2021
2017
2018
2019
2020
2021

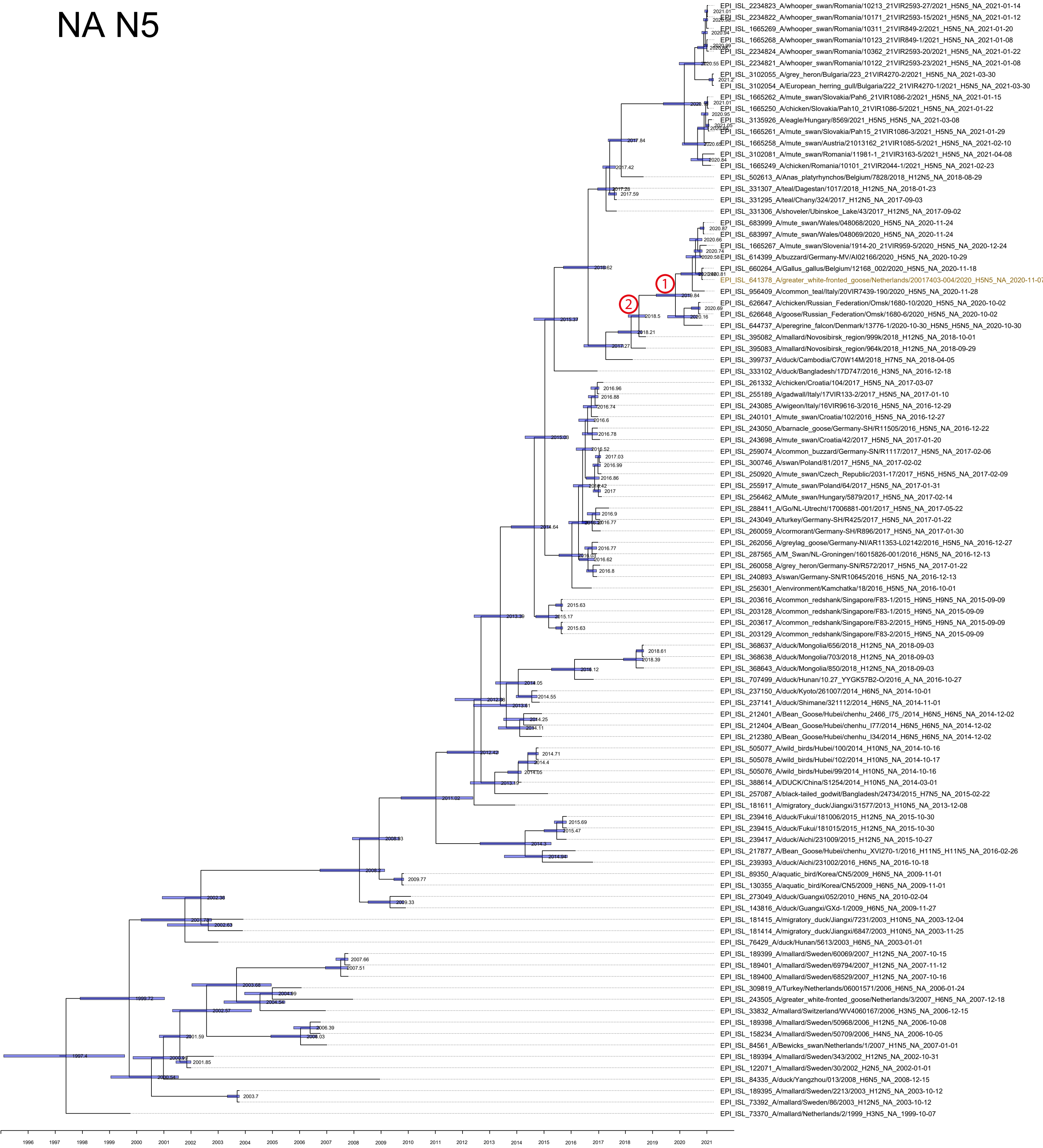
NA N5
EPI_ISL_2234823_A/whooper_swan/Romania/10213_21VIR2593-27/2021_H5N5_NA_2021-01-14
EPI_ISL_2234822_A/whooper_swan/Romania/10171_21VIR2593-15/2021_H5N5_NA_2021-01-12
EPI_ISL_1665269_A/whooper_swan/Romania/10311_21VIR849-2/2021_H5N5_NA_2021-01-20
EPI_ISL_1665268_A/whooper_swan/Romania/10123_21VIR849-1/2021_H5N5_NA_2021-01-08
EPI_ISL_2234824_A/whooper_swan/Romania/10362_21VIR2593-20/2021_H5N5_NA_2021-01-22
EPI_ISL_2234821_A/whooper_swan/Romania/10122_21VIR2593-23/2021_H5N5_NA_2021-01-08
EPI_ISL_3102055_A/grey_heron/Bulgaria/223_21VIR4270-2/2021_H5N5_NA_2021-03-30
EPI_ISL_3102054_A/European_herring_gull/Bulgaria/222_21VIR4270-1/2021_H5N5_NA_2021-03-30
EPI_ISL_1665262_A/mute_swan/Slovakia/Pah6_21VIR1086-2/2021_H5N5_NA_2021-01-15
EPI_ISL_1665250_A/chicken/Slovakia/Pah10_21VIR1086-5/2021_H5N5_NA_2021-01-22
EPI_ISL_3135926_A/eagle/Hungary/8569/2021_H5N5_H5N5_NA_2021-03-08
EPI_ISL_1665261_A/mute_swan/Slovakia/Pah15_21VIR1086-3/2021_H5N5_NA_2021-01-29
EPI_ISL_1665258_A/mute_swan/Austria/21013162_21VIR1085-5/2021_H5N5_NA_2021-02-10
EPI_ISL_3102081_A/mute_swan/Romania/11981-1_21VIR3163-5/2021_H5N5_NA_2021-04-08
EPI_ISL_1665249_A/chicken/Romania/10101_21VIR2044-1/2021_H5N5_NA_2021-02-23
EPI_ISL_502613_A/Anas_platyrhynchos/Belgium/7828/2018_H12N5_NA_2018-08-29
EPI_ISL_331307_A/teal/Dagestan/1017/2018_H12N5_NA_2018-01-23
EPI_ISL_331295_A/teal/Chany/324/2017_H12N5_NA_2017-09-03
EPI_ISL_331306_A/shoveler/Ubinskoe_Lake/43/2017_H12N5_NA_2017-09-02
EPI_ISL_683999_A/mute_swan/Wales/048068/2020_H5N5_NA_2020-11-24
EPI_ISL_683997_A/mute_swan/Wales/048069/2020_H5N5_NA_2020-11-24
EPI_ISL_1665267_A/mute_swan/Slovenia/1914-20_21VIR959-5/2020_H5N5_NA_2020-12-24
EPI_ISL_614399_A/buzzard/Germany-MV/AI02166/2020_H5N5_NA_2020-10-29
EPI_ISL_660264_A/Gallus_gallus/Belgium/12168_002/2020_H5N5_NA_2020-11-18
EPI_ISL_641378_A/greater_white-fronted_goose/Netherlands/20017403-004/2020_H5N5_NA_2020-11-0
1
EPI_ISL_956409_A/common_teal/Italy/20VIR7439-190/2020_H5N5_NA_2020-11-28
2
EPI_ISL_626647_A/chicken/Russian_Federation/Omsk/1680-10/2020_H5N5_NA_2020-10-02
EPI_ISL_626648_A/goose/Russian_Federation/Omsk/1680-6/2020_H5N5_NA_2020-10-02
EPI_ISL_644737_A/peregrine_falcon/Denmark/13776-1/2020-10-30_H5N5_H5N5_NA_2020-10-30
EPI_ISL_395082_A/mallard/Novosibirsk_region/999k/2018_H12N5_NA_2018-10-01
EPI_ISL_395083_A/mallard/Novosibirsk_region/964k/2018_H12N5_NA_2018-09-29
EPI_ISL_399737_A/duck/Cambodia/C70W14M/2018_H7N5_NA_2018-04-05
EPI_ISL_333102_A/duck/Bangladesh/17D747/2016_H3N5_NA_2016-12-18
EPI_ISL_261332_A/chicken/Croatia/104/2017_H5N5_NA_2017-03-07
EPI_ISL_255189_A/gadwall/Italy/17VIR133-2/2017_H5N5_NA_2017-01-10
EPI_ISL_243085_A/wigeon/Italy/16VIR9616-3/2016_H5N5_NA_2016-12-29
EPI_ISL_240101_A/mute_swan/Croatia/102/2016_H5N5_NA_2016-12-27
EPI_ISL_243050_A/barnacle_goose/Germany-SH/R11505/2016_H5N5_NA_2016-12-22
EPI_ISL_243698_A/mute_swan/Croatia/42/2017_H5N5_NA_2017-01-20
EPI_ISL_259074_A/common_buzzard/Germany-SN/R1117/2017_H5N5_NA_2017-02-06
EPI_ISL_300746_A/swan/Poland/81/2017_H5N5_NA_2017-02-02
EPI_ISL_250920_A/mute_swan/Czech_Republic/2031-17/2017_H5N5_H5N5_NA_2017-02-09
EPI_ISL_255917_A/mute_swan/Poland/64/2017_H5N5_NA_2017-01-31
EPI_ISL_256462_A/Mute_swan/Hungary/5879/2017_H5N5_NA_2017-02-14
EPI_ISL_288411_A/Go/NL-Utrecht/17006881-001/2017_H5N5_NA_2017-05-22
EPI_ISL_243049_A/turkey/Germany-SH/R425/2017_H5N5_NA_2017-01-22
EPI_ISL_260059_A/cormorant/Germany-SH/R896/2017_H5N5_NA_2017-01-30
EPI_ISL_262056_A/greylag_goose/Germany-NI/AR11353-L02142/2016_H5N5_NA_2016-12-27
EPI_ISL_287565_A/M_Swan/NL-Groningen/16015826-001/2016_H5N5_NA_2016-12-13
EPI_ISL_260058_A/grey_heron/Germany-SN/R572/2017_H5N5_NA_2017-01-22
EPI_ISL_240893_A/swan/Germany-SH/R10645/2016_H5N5_NA_2016-12-13
EPI_ISL_256301_A/environment/Kamchatka/18/2016_H5N5_NA_2016-10-01
EPI_ISL_203616_A/common_redshank/Singapore/F83-1/2015_H9N5_H9N5_NA_2015-09-09
EPI_ISL_203128_A/common_redshank/Singapore/F83-1/2015_H9N5_NA_2015-09-09
EPI_ISL_203617_A/common_redshank/Singapore/F83-2/2015_H9N5_H9N5_NA_2015-09-09
EPI_ISL_203129_A/common_redshank/Singapore/F83-2/2015_H9N5_NA_2015-09-09
EPI_ISL_368637_A/duck/Mongolia/656/2018_H12N5_NA_2018-09-03
EPI_ISL_368638_A/duck/Mongolia/703/2018_H12N5_NA_2018-09-03
EPI_ISL_368643_A/duck/Mongolia/850/2018_H12N5_NA_2018-09-03
EPI_ISL_707499_A/duck/Hunan/10.27_YYGK57B2-O/2016_A_NA_2016-10-27
EPI_ISL_237150_A/duck/Kyoto/261007/2014_H6N5_NA_2014-10-01
EPI_ISL_237141_A/duck/Shimane/321112/2014_H6N5_NA_2014-11-01
EPI_ISL_212401_A/Bean_Goose/Hubei/chenhu_2466_I75_/2014_H6N5_H6N5_NA_2014-12-02
EPI_ISL_212404_A/Bean_Goose/Hubei/chenhu_I77/2014_H6N5_H6N5_NA_2014-12-02
EPI_ISL_212380_A/Bean_Goose/Hubei/chenhu_I34/2014_H6N5_H6N5_NA_2014-12-02
EPI_ISL_505077_A/wild_birds/Hubei/100/2014_H10N5_NA_2014-10-16
EPI_ISL_505078_A/wild_birds/Hubei/102/2014_H10N5_NA_2014-10-17
EPI_ISL_505076_A/wild_birds/Hubei/99/2014_H10N5_NA_2014-10-16
EPI_ISL_388614_A/DUCK/China/S1254/2014_H10N5_NA_2014-03-01
EPI_ISL_257087_A/black-tailed_godwit/Bangladesh/24734/2015_H7N5_NA_2015-02-22
EPI_ISL_181611_A/migratory_duck/Jiangxi/31577/2013_H10N5_NA_2013-12-08
EPI_ISL_239416_A/duck/Fukui/181006/2015_H12N5_NA_2015-10-30
EPI_ISL_239415_A/duck/Fukui/181015/2015_H12N5_NA_2015-10-30
EPI_ISL_239417_A/duck/Aichi/231009/2015_H12N5_NA_2015-10-27
EPI_ISL_217877_A/Bean_Goose/Hubei/chenhu_XVI270-1/2016_H11N5_H11N5_NA_2016-02-26
EPI_ISL_239393_A/duck/Aichi/231002/2016_H6N5_NA_2016-10-18
EPI_ISL_89350_A/aquatic_bird/Korea/CN5/2009_H6N5_NA_2009-11-01
EPI_ISL_130355_A/aquatic_bird/Korea/CN5/2009_H6N5_NA_2009-11-01
EPI_ISL_273049_A/duck/Guangxi/052/2010_H6N5_NA_2010-02-04
EPI_ISL_143816_A/duck/Guangxi/GXd-1/2009_H6N5_NA_2009-11-27
EPI_ISL_181415_A/migratory_duck/Jiangxi/7231/2003_H10N5_NA_2003-12-04
EPI_ISL_181414_A/migratory_duck/Jiangxi/6847/2003_H10N5_NA_2003-11-25
EPI_ISL_76429_A/duck/Hunan/5613/2003_H6N5_NA_2003-01-01
EPI_ISL_189399_A/mallard/Sweden/60069/2007_H12N5_NA_2007-10-15
EPI_ISL_189401_A/mallard/Sweden/69794/2007_H12N5_NA_2007-11-12
EPI_ISL_189400_A/mallard/Sweden/68529/2007_H12N5_NA_2007-10-16
EPI_ISL_309819_A/Turkey/Netherlands/06001571/2006_H6N5_NA_2006-01-24
EPI_ISL_243505_A/greater_white-fronted_goose/Netherlands/3/2007_H6N5_NA_2007-12-18
EPI_ISL_33832_A/mallard/Switzerland/WV4060167/2006_H3N5_NA_2006-12-15
EPI_ISL_189398_A/mallard/Sweden/50968/2006_H12N5_NA_2006-10-08
EPI_ISL_158234_A/mallard/Sweden/50709/2006_H4N5_NA_2006-10-05
EPI_ISL_84561_A/Bewicks_swan/Netherlands/1/2007_H1N5_NA_2007-01-01
EPI_ISL_189394_A/mallard/Sweden/343/2002_H12N5_NA_2002-10-31
EPI_ISL_122071_A/mallard/Sweden/30/2002_H2N5_NA_2002-01-01
EPI_ISL_84335_A/duck/Yangzhou/013/2008_H6N5_NA_2008-12-15
EPI_ISL_189395_A/mallard/Sweden/2213/2003_H12N5_NA_2003-10-12
EPI_ISL_73392_A/mallard/Sweden/86/2003_H12N5_NA_2003-10-12
EPI_ISL_73370_A/mallard/Netherlands/2/1999_H3N5_NA_1999-10-07
1996
1997
1998
1999
2000
2001
2002
2003
2004
2005
2006
2007
2008
2009
2010
2011
2012
2013
2014
2015
2016
2017
2018
2019
2020
2021

Fig S1: Molecular clock analysis was performed for the H5, N8, N1, N3, N4 and N5 segments of the viruses isolated in the Netherlands in 2020-2021. In the time scaled phylogenetic trees the H5N8 viruses are shown in blue, H5N1 in green, H5N3 in orange, H5N4 in purple and H5N5 in brown. The tMRCA for the marked nodes is listed in Table 2, as is the credible interval and posterior value. The GISAID accession numbers are shown in the trees (for details see Supplementary Table S5).

Table S1: Dead wild bird species testing positive for HPAI H5 subtypes of viruses between October 2020 and June 2021.

	Total	H5N8	H5N1	H5N3	H5N4	H5N5	H5Nx
Goose	155						
Barnacle Goose	79	30	17	0	0	0	32
Greylag Goose	34	20	2	0	0	0	12
Goose (unidentified)	25	19	2	0	0	0	4
Greater Canada Goose	6	4	0	0	0	0	2
Egyptian Goose	4	2	0	0	0	0	2
Bean Goose	2	2	0	0	0	0	0
Brant Goose	1	1	0	0	0	0	0
Greater White-fronted Goose	2	1	0	0	0	1	0
Pink-footed Goose	2	2	0	0	0	0	0
Swan	52						
Mute swan	45	28	2	0	0	0	15
Swan (unidentified)	5	2	1	0	0	0	2
Black swan	1	1	0	0	0	0	0
Whooper swan	1	1	0	0	0	0	0
Duck	25						
Eurasian wigeon	9	5	0	0	0	0	4
Wild duck (unidentified)	8	6	0	0	0	0	2
Mallard	3	2	0	0	0	0	1
Eurasian teal	2	0	1	0	0	0	1
Muscovy duck	3	3	0	0	0	0	0
Predators	43						
Common buzzard	23	17	3	2	0	0	1
Common Krestrel	5	4	0	0	0	0	1
Peregrine Falcon	7	6	0	0	1	0	0
Northern Goshawk	2	2	0	0	0	0	0
Northern Lapwing	3	3	0	0	0	0	0
Short-eared Owl	1	1	0	0	0	0	0
White-tailed Eagle	2	0	2	0	0	0	0
Others	18						
Black-headed Gull	1	1	0	0	0	0	0
Common Murre	1	0	1	0	0	0	0
Common Snipe	1	1	0	0	0	0	0
Dunlin	1	0	0	0	0	0	1
Eurasian Curlew	3	0	1	0	1	0	1
Eurasian Oystercatcher	1	1	0	0	0	0	0
European Herring Gull	4	1	1	0	1	0	1
Gadwall	**2**	2	0	0	0	0	0
Great Cormorant	2	2	0	0	0	0	0
Great Egret	1	1	0	0	0	0	0
Sanderling	1	1	0	0	0	0	0
Total		172	33	2	3	1	82

Table S2: Details of full genome sequences of HPAI viruses detected in the Netherlands between October 2020 and June 2021

GISAID Isolate ID	Name	Collection date	Host species	Subtype	Sample ID
EPI_ISL_591075*	A/mute_swan/Netherlands/20015931-001/2020	2020-10-17	Mute Swan	H5N8	20015931-001
EPI_ISL_603132*	A/chicken/Netherlands/20016597-026030/2020	2020-10-28	Chicken	H5N8	20016597-026-030
EPI_ISL_623075	A/chicken/Netherlands/20017138-016020/2020	2020-11-04	Chicken	H5N8	20017138-016-020
EPI_ISL_632314*	A/greylag_goose/Netherlands/20016582-004/2020	2020-10-28	Greylag goose	H5N1	20016582-004
EPI_ISL_632315*	A/eurasian_teal/Netherlands/20016896-013/2020	2020-11-02	Eurasian teal	H5N1	20016896-013
EPI_ISL_632316	A/eurasian_curlew/Netherlands/20016890-001/2020	2020-11-01	Eurasian curlew	H5N1	20016890-001
EPI_ISL_632317*	A/barnacle_goose/Netherlands/20016935-002/2020	2020-11-01	Barnacle Goose	H5N8	20016935-002
EPI_ISL_632318*	A/greylag_goose/Netherlands/20016879-001/2020	2020-11-01	Greylag goose	H5N8	20016879-001
EPI_ISL_632319	A/chicken/Netherlands/20017460-011015/2020	2020-11-09	Chicken	H5N8	20017460-011-015
EPI_ISL_641377*	A/chicken/Netherlands/20016978-001/2020	2020-11-02	Chicken	H5N8	20016978-001
EPI_ISL_641378	A/greater_white-fronted_goose/Netherlands/20017403-004/202(	2020-11-07	Greater white-fronted goose	H5N5	20017403-004
EPI_ISL_641394*	A/chicken/Netherlands/20017639-001/2020	2020-11-10	Chicken	H5N8	20017639-001
EPI_ISL_641395*	A/chicken/Netherlands/20017694-004/2020	2020-11-11	Chicken	H5N8	20017694-004
EPI_ISL_641518	A/duck/Netherlands/20017868-016020/2020	2020-11-13	Duck	H5N8	20017868-016-020
EPI_ISL_653918	A/greylag_goose/Netherlands/20016414-001/2020	2020-10-26	Greylag goose	H5N8	20016414-001
EPI_ISL_653919	A/greylag_goose/Netherlands/20016494-001/2020	2020-10-27	Greylag goose	H5N8	20016494-001
EPI_ISL_653920	A/chicken/Netherlands/20018496-006010/2020	2020-11-20	Chicken	H5N8	20018496-006-010
EPI_ISL_653921	A/chicken/Netherlands/20018523-001005/2020	2020-11-21	Chicken	H5N8	20018523-001-005
EPI_ISL_693515	A/chicken/Netherlands/20019411-006010/2020	2020-12-04	Chicken	H5N8	20019411-006-010
EPI_ISL_710533	A/chicken/Netherlands/20019237-001005/2020	2020-12-02	Chicken	H5N8	20019237-001-005
EPI_ISL_710538	A/chicken/Netherlands/20019226-001/2020	2020-12-02	Chicken	H5N8	20019226-001
EPI_ISL_710539	A/chicken/Netherlands/20019422-001005/2020	2020-12-06	Chicken	H5N8	20019422-001-005
EPI_ISL_711055*	A/chicken/Netherlands/20019879-001005/2020	2020-12-14	Chicken	H5N1	20019879-001-005
EPI_ISL_711056	A/mute_swan/Netherlands/20019252-002/2020	2020-12-02	Mute swan	H5N8	20019252-002
EPI_ISL_711058	A/mute_swan/Netherlands/20019255-002/2020	2020-12-02	Mute swan	H5N8	20019255-002
EPI_ISL_711059	A/greylag_goose/Netherlands/20019685-002/2020	2020-12-08	Greylag goose	H5N1	20019685-002
EPI_ISL_775248	A/muscovy_duck/Netherlands/20019914-001/2020_A	2020-12-11	Muscovy duck	H5N8	20019914-001
EPI_ISL_775251	A/peregrine_falcon/Netherlands/20020038-001/2020	2020-12-13	Peregrine falcon	H5N8	20020038-001
EPI_ISL_775253	A/mute_swan/Netherlands/20020133-001/2020_A	2020-12-15	Mute swan	H5N8	20020133-001
EPI_ISL_775266	A/black-headed_gull/Netherlands/20020162-002/2020_A	2020-12-17	Black-headed gull	H5N8	20020162-002
EPI_ISL_775267	A/turkey/Netherlands/21020942-001005/2021_A	2021-01-04	Turkey	H5N8	21020942-001-005
EPI_ISL_1046889	A/chicken/Netherlands/21023815-001005/2021	2021-02-19	Chicken	H5N8	21023815-001-005
EPI_ISL_1048238	A/common_kestrel/Netherlands/20020264-002/2020	2020-12-17	Common kestrel	H5N8	20020264-002
EPI_ISL_1048239	A/common_buzzard/Netherlands/21021023-002/2021	2021-01-04	Common_Buzzard	H5N3	21021023-002
EPI_ISL_1048240	A/common_buzzard/Netherlands/21021396-002/2021	2021-01-09	Common_Buzzard	H5N1	21021396-002
EPI_ISL_1048241	A/barnacle_goose/Netherlands/21021591-001/2021	2021-01-14	Barnacle Goose	H5N1	21021591-001
EPI_ISL_1139014	A/barnacle_goose/Netherlands/20016511-002/2020	2020-10-27	Barnacle Goose	H5N8	20016511-002
EPI_ISL_1139015	A/wild_bird/Netherlands/20016515-002/2020	2020-10-26	Mute swan	H5N8	20016515-002
EPI_ISL_1139016	A/mute_swan/Netherlands/20016516-001/2020	2020-10-27	Mute swan	H5N8	20016516-001
EPI_ISL_1139017	A/mute_swan/Netherlands/20016634-001/2020	2020-10-28	Mute swan	H5N8	20016634-001
EPI_ISL_1139018	A/cormorant/Netherlands/20016582-005/2020	2020-10-28	Cormorant	H5N8	20016582-005
EPI_ISL_1139019	A/mute_swan/Netherlands/20016618-001/2020	2020-10-28	Mute swan	H5N8	20016618-001
EPI_ISL_1139020	A/greylag_goose/Netherlands/20016756-001/2020	2020-10-29	Greylag goose	H5N8	20016756-001
EPI_ISL_1139021	A/eurasian_wigeon/Netherlands/20016758-001/2020	2020-10-29	Eurasian wigeon	H5N8	20016758-001
EPI_ISL_1139022	A/barnacle_goose/Netherlands/20016888-001/2020	2020-10-29	Barnacle Goose	H5N8	20016888-001
EPI_ISL_1139023	A/short-eared_owl/Netherlands/20016896-017/2020	2020-11-02	Short-eared Owl	H5N8	20016896-017
EPI_ISL_1139024	A/mute_swan/Netherlands/20016973-001/2020	2020-11-02	Mute swan	H5N8	20016973-001
EPI_ISL_1139025	A/barnacle_goose/Netherlands/20016974-002/2020	2020-11-02	Barnacle Goose	H5N8	20016974-002
EPI_ISL_1139026	A/greylag_goose/Netherlands/20016975-004/2020	2020-11-02	Greylag goose	H5N8	20016975-004
EPI_ISL_1139027	A/egyptian_goose/Netherlands/20017027-002/2020	2020-11-03	Egyptian Goose	H5N8	20017027-002
EPI_ISL_1139028	A/barnacle_goose/Netherlands/20017051-001/2020	2020-11-03	Barnacle Goose	H5N8	20017051-001
EPI_ISL_1139029	A/barnacle_goose/Netherlands/20017051-006/2020	2020-11-03	Barnacle Goose	H5N8	20017051-006
EPI_ISL_1139030	A/barnacle_goose/Netherlands/20017052-001/2020	2020-11-03	Barnacle Goose	H5N8	20017052-001
EPI_ISL_1139031	A/mute_swan/Netherlands/20017131-006/2020	2020-11-04	Mute swan	H5N8	20017131-006
EPI_ISL_1139032	A/barnacle_goose/Netherlands/20017159-006/2020	2020-11-04	Barnacle Goose	H5N8	20017159-006
EPI_ISL_1139033	A/greylag_goose/Netherlands/20017399-006/2020	2020-11-06	Greylag goose	H5N8	20017399-006
EPI_ISL_1139034	A/barnacle_goose/Netherlands/20017405-002/2020	2020-11-07	Barnacle Goose	H5N8	20017405-002
EPI_ISL_1139035	A/greylag_goose/Netherlands/20016877-001/2020	2020-10-30	Greylag goose	H5N8	20016877-001
EPI_ISL_1139036	A/brant_goose/Netherlands/20016948-002/2020	2020-10-31	Brent goose	H5N8	20016948-002
EPI_ISL_1139037	A/barnacle_goose/Netherlands/20016951-001/2020	2020-10-31	Barnacle Goose	H5N8	20016951-001
EPI_ISL_1139038	A/wild_goose/Netherlands/20016959-001/2020	2020-11-02	Goose	H5N8	20016959-001
EPI_ISL_1139039	A/mute_swan/Netherlands/20016960-001/2020	2020-11-02	Mute swan	H5N8	20016960-001
EPI_ISL_1139040	A/mute_swan/Netherlands/20017061-001/2020	2020-11-02	Mute swan	H5N8	20017061-001
EPI_ISL_1139041	A/greylag_goose/Netherlands/20017064-002/2020	2020-11-03	Greylag goose	H5N8	20017064-002
EPI_ISL_1139042	A/mute_swan/Netherlands/20017153-002/2020	2020-11-03	Mute swan	H5N8	20017153-002
EPI_ISL_1139043	A/mute_swan/Netherlands/20017547-002/2020	2020-11-10	Mute swan	H5N8	20017547-002
EPI_ISL_1139044	A/eurasian_oystercatcher/Netherlands/20017557-003/2020	2020-11-10	Oystercatcher	H5N8	20017557-003
EPI_ISL_1139045	A/greylag_goose/Netherlands/20017476-001/2020	2020-11-06	Greylag goose	H5N8	20017476-001
EPI_ISL_1139046	A/greater_canada_goose/Netherlands/20017479-002/2020	2020-11-06	Canada goose	H5N8	20017479-002
EPI_ISL_1139047	A/northern_lapwing/Netherlands/20017480-001/2020	2020-11-06	Lapwing	H5N8	20017480-001
EPI_ISL_1139048	A/wild_goose/Netherlands/20017495-002/2020	2020-11-09	Goose	H5N8	20017495-002

EPI_ISL_1139049	A/gadwall/Netherlands/20017254-001/2020	2020-11-01	Gadwall	H5N8	20017254-001
EPI_ISL_1139050	A/northern_lapwing/Netherlands/20017255-001/2020	2020-11-01	Lapwing	H5N8	20017255-001
EPI_ISL_1139051	A/greylag_goose/Netherlands/20017256-002/2020	2020-11-03	Greylag goose	H5N8	20017256-002
EPI_ISL_1139076	A/common_kestrel/Netherlands/20017381-001/2020	2020-11-05	Common Kestrel	H5N8	20017381-001
EPI_ISL_1139078	A/pink-footed_goose/Netherlands/20017382-001/2020	2020-11-06	Pink-footed goose	H5N8	20017382-001
EPI_ISL_1139079	A/barnacle_goose/Netherlands/20016888-002/2020	2020-10-29	Barnacle Goose	H5N8	20016888-002
EPI_ISL_1139080	A/barnacle_goose/Netherlands/20016888-003/2020	2020-10-29	Barnacle Goose	H5N8	20016888-003
EPI_ISL_1139081	A/greylag_goose/Netherlands/20016896-001/2020	2020-11-02	Greylag goose	H5N8	20016896-001
EPI_ISL_1139082	A/barnacle_goose/Netherlands/20016896-011/2020	2020-11-02	Barnacle Goose	H5N8	20016896-011
EPI_ISL_1139083	A/barnacle_goose/Netherlands/20016896-012/2020	2020-11-02	Barnacle Goose	H5N8	20016896-012
EPI_ISL_1139084	A/eurasian_wigeon/Netherlands/20016896-025/2020	2020-11-02	Eurasian wigeon	H5N8	20016896-025
EPI_ISL_1139085	A/barnacle_goose/Netherlands/20016935-003/2020	2020-11-01	Barnacle Goose	H5N8	20016935-003
EPI_ISL_1139086	A/greater_canada_goose/Netherlands/20017403-003/2020	2020-11-07	Greater Canada Goose	H5N8	20017403-003
EPI_ISL_1139087	A/barnacle_goose/Netherlands/20017557-001/2020	2020-11-10	Barnacle Goose	H5N8	20017557-001
EPI_ISL_1139088	A/barnacle_goose/Netherlands/20017557-002/2020	2020-11-10	Barnacle Goose	H5N8	20017557-002
EPI_ISL_1139089	A/wild_duck/Netherlands/20017794-001/2020	2020-11-12	Duck	H5N8	20017794-001
EPI_ISL_1139090	A/common_buzzard/Netherlands/20017824-001/2020	2020-11-12	Common buzzard	H5N8	20017824-001
EPI_ISL_1139091	A/muscovy_duck/Netherlands/20017611-002/2020	2020-11-10	Muscovy duck	H5N8	20017611-002
EPI_ISL_1139092	A/barnacle_goose/Netherlands/20017713-002/2020	2020-11-08	Barnacle Goose	H5N8	20017713-002
EPI_ISL_1139093	A/gadwall/Netherlands/20017716-001/2020	2020-11-10	Gadwall	H5N8	20017716-001
EPI_ISL_1139094	A/mute_swan/Netherlands/20017717-002/2020	2020-11-10	Mute swan	H5N8	20017717-002
EPI_ISL_1139095	A/great_egret/Netherlands/20017754-002/2020	2020-11-01	Great white egret	H5N8	20017754-002
EPI_ISL_1139096	A/wild_goose/Netherlands/20017755-002/2020	2020-11-01	Goose	H5N8	20017755-002
EPI_ISL_1139097	A/wild_goose/Netherlands/20017761-002/2020	2020-11-04	Goose	H5N8	20017761-002
EPI_ISL_1139098	A/eurasian_wigeon/Netherlands/20017908-002/2020	2020-11-10	Eurasian wigeon	H5N8	20017908-002
EPI_ISL_1139099	A/barnacle_goose/Netherlands/20017984-004/2020	2020-11-13	Barnacle Goose	H5N8	20017984-004
EPI_ISL_1139100	A/barnacle_goose/Netherlands/20017604-001/2020	2020-11-08	Barnacle Goose	H5N8	20017604-001
EPI_ISL_1139101	A/swan/Netherlands/20017605-002/2020	2020-11-09	Swan	H5N1	20017605-002
EPI_ISL_1139102	A/swan/Netherlands/20017772-002/2020	2020-11-10	Swan	H5N1	20017772-002
EPI_ISL_1139103	A/peregrine_falcon/Netherlands/20017773-002/2020	2020-11-01	Peregrine falcon	H5N8	20017773-002
EPI_ISL_1139104	A/wild_goose/Netherlands/20017816-001/2020	2020-11-11	Goose	H5N8	20017816-001
EPI_ISL_1139105	A/greylag_goose/Netherlands/20016523-001/2020	2020-10-26	Greylag goose	H5N8	20016523-001
EPI_ISL_1139106	A/eurasian_curlew/Netherlands/20016896-019/2020	2020-11-02	Eurasian Curlew	H5N8	20016896-019
EPI_ISL_1139107	A/muscovy_duck/Netherlands/20018067-001/2020	2020-11-12	Muscovy duck	H5N8	20018067-001
EPI_ISL_1139108	A/mute_swan/Netherlands/20018754-004/2020	2020-11-24	Mute swan	H5N8	20018754-004
EPI_ISL_1139109	A/mute_swan/Netherlands/20018754-006/2020	2020-11-24	Mute swan	H5N8	20018754-006
EPI_ISL_1139110	A/swan/Netherlands/20018830-004/2020	2020-11-25	Swan	H5N8	20018830-004
EPI_ISL_1139112	A/greylag_goose/Netherlands/20017386-001/2020	2020-11-05	Greylag goose	H5N8	20017386-001
EPI_ISL_1139113	A/wild_goose/Netherlands/20017819-001/2020	2020-11-10	Goose	H5N8	20017819-001
EPI_ISL_1139159	A/greylag_goose/Netherlands/20017058-002/2020	2020-10-25	Greylag goose	H5N8	20017058-002
EPI_ISL_1139160	A/pink-footed_goose/Netherlands/20018068-001/2020	2020-11-14	Bean goose	H5N8	20018068-001
EPI_ISL_1139161	A/greylag_goose/Netherlands/20018070-002/2020	2020-11-15	Greylag goose	H5N8	20018070-002
EPI_ISL_1139162	A/black_swan/Netherlands/20018185-001/2020	2020-11-16	Black Swan	H5N8	20018185-001
EPI_ISL_1139163	A/common_buzzard/Netherlands/20018339-002/2020	2020-11-16	Buzzard	H5N8	20018339-002
EPI_ISL_1139164	A/northern_goshawk/Netherlands/20018560-002/2020	2020-11-22	Northern goshawk	H5N8	20018560-002
EPI_ISL_1139165	A/common_snipe/Netherlands/20018931-003/2020	2020-11-26	Common snipe	H5N8	20018931-003
EPI_ISL_1139166	A/wild_goose/Netherlands/20018735-002/2020	2020-11-24	Goose	H5N8	20018735-002
EPI_ISL_1139167	A/barnacle_goose/Netherlands/20018737-002/2020	2020-11-24	Barnacle Goose	H5N8	20018737-002
EPI_ISL_1139168	A/mute_swan/Netherlands/20018738-001/2020	2020-11-24	Mute swan	H5N8	20018738-001
EPI_ISL_1139169	A/peregrine_falcon/Netherlands/20018821-002/2020	2020-11-25	Peregrine falcon	H5N8	20018821-002
EPI_ISL_1139170	A/wild_goose/Netherlands/20018822-002/2020	2020-11-25	Goose	H5N8	20018822-002
EPI_ISL_1139171	A/mute_swan/Netherlands/20018824-002/2020	2020-11-25	Mute swan	H5N8	20018824-002
EPI_ISL_1139172	A/mute_swan/Netherlands/20018923-001/2020	2020-11-26	Mute swan	H5N8	20018923-001
EPI_ISL_1139173	A/mute_swan/Netherlands/20019137-005/2020	2020-11-30	Mute swan	H5N8	20019137-005
EPI_ISL_1224942	A/peacock/Netherlands/21022591-002/2021	2021-02-01	Peacock	H5N8	21022591-002
EPI_ISL_1224946	A/barnacle_goose/Netherlands/21022611-001/2021	2021-01-31	Barnacle Goose	H5N1	21022611-001
EPI_ISL_1224949	A/mute_swan/Netherlands/21022898-002/2021	2021-02-04	Mute swan	H5N8	21022898-002
EPI_ISL_1224989	A/common_buzzard/Netherlands/21022834-002/2021	2021-02-01	Common buzzard	H5N1	21022834-002
EPI_ISL_1225077	A/european_herring_gull/Netherlands/21023937-002/2021	2021-02-21	European Herring Gull	H5N4	21023937-002
EPI_ISL_1225079	A/eurasian_curlew/Netherlands/21024069-002/2021	2021-02-15	Eurasian Curlew	H5N4	21024069-002
EPI_ISL_2172517	A/common_buzzard/Netherlands/21021278-002/2021	2021-01-05	Common buzzard	H5N8	21021278-002
EPI_ISL_2172518	A/common_kestrel/Netherlands/21021301-039/2021	2021-01-08	Common Kestrel	H5N8	21021301-039
EPI_ISL_2172522	A/common_buzzard/Netherlands/21021497-001/2021	2021-01-10	Common buzzard	H5N8	21021497-001
EPI_ISL_2172523	A/sanderling/Netherlands/21021794-002/2021	2021-01-18	Sanderling	H5N8	21021794-002
EPI_ISL_2172525	A/barnacle_goose/Netherlands/21022039-002/2021	2021-01-19	Barnacle Goose	H5N8	21022039-002
EPI_ISL_2172526	A/common_buzzard/Netherlands/21023939-001/2021	2021-02-21	Common buzzard	H5N8	21023939-001
EPI_ISL_2172528	A/barnacle_goose/Netherlands/21024066-001/2021	2021-02-22	Barnacle Goose	H5N1	21024066-001
EPI_ISL_2172529	A/wild_goose/Netherlands/21024076-001/2021	2021-01-30	Goose	H5N8	21024076-001
EPI_ISL_2172530	A/common_buzzard/Netherlands/21024357-002/2021	2021-02-22	Common buzzard	H5N3	21024357-002
EPI_ISL_2172531	A/barnacle_goose/Netherlands/21024358-001/2021	2021-02-21	Barnacle Goose	H5N8	21024358-001
EPI_ISL_2172532	A/gadwall/Netherlands/21024401-002/2021	2021-01-25	Gadwall	H5N8	21024401-002
EPI_ISL_2172533	A/common_buzzard/Netherlands/21024712-002/2021	2021-03-04	Common buzzard	H5N8	21024712-002
EPI_ISL_2172608	A/barnacle_goose/Netherlands/21024897-001/2021	2021-03-09	Barnacle Goose	H5N8	21024897-001
EPI_ISL_2173364	A/peregrine_falcon/Netherlands/21025108-001/2021	2021-03-10	Peregrine Falcon	H5N4	21025108-001
EPI_ISL_2174084*	A/common_murre/Netherlands/21025491-002/2021	2021-03-17	Common Murre	H5N1	21025491-002

EPI_ISL_2174728	A/barnacle_goose/Netherlands/21025769-002/2021	2021-03-22	Barnacle Goose	H5N1	21025769-002
EPI_ISL_2175628	A/common_buzzard/Netherlands/21021187-001/2021	2021-01-04	Common buzzard	H5N8	21021187-001
EPI_ISL_2176321	A/barnacle_goose/Netherlands/21023498-002/2021	2021-02-14	Barnacle Goose	H5N8	21023498-002
EPI_ISL_2176841	A/barnacle_goose/Netherlands/21023501-002/2021	2021-02-05	Barnacle Goose	H5N8	21023501-002
EPI_ISL_2177398	A/peacock/Netherlands/21026542-001/2021	2021-04-07	Peacock	H5N8	21026542-001
EPI_ISL_2193998*	A/barnacle_goose/Netherlands/21027016-002/2021	2021-04-15	Barnacle Goose	H5N1	21027016-002
EPI_ISL_2194014*	A/white-tailed_eagle/Netherlands/21027616-001/2021	2021-04-23	White-tailed Eagle	H5N1	21027616-001
EPI_ISL_2194036*	A/barnacle_goose/Netherlands/21028196-002/2021	2021-04-22	Barnacle Goose	H5N1	21028196-002
EPI_ISL_2227275	A/turkey/Netherlands/21028936-001005/2021	2021-05-21	Turkey	H5N8	21028936-001005
EPI_ISL_2227276	A/barnacle_goose/Netherlands/21027357-002/2021	2021-04-20	Barnacle Goose	H5N1	21027357-002
EPI_ISL_2227277	A/barnacle_goose/Netherlands/21028534-002/2021	2021-05-13	Barnacle Goose	H5N1	21028534-002
EPI_ISL_2227278	A/goose/Netherlands/21028502-002/21028502/2021	2021-05-09	Goose	H5N1	21028502-002
EPI_ISL_2227279	A/western_marsh_harrier/Netherlands/21028606-002/2021	2021-05-07	Western Marsh Harrier	H5N1	21028606-002

* Sequences were determined and submitted to GISAID in previous studies.

Table S3: closest related virus isolated from a dead wild bird

Farm	Location	Virus subtype	Sample date	Poultry virus	Closest related poultry virus	Farm location	Sample date	Days since detection closest related	Distance between farms (km)	Number of nucleotide differences
1	Altforst	HPAI H5N8	2020-10-28	EPI_ISL_603132	EPI_ISL_623075	Puiflijk	2020-11-04	-7	2	0
2	Puiflijk	HPAI H5N8	2020-11-04	EPI_ISL_623075	EPI_ISL_603132	Altforst	2020-10-28	7	2	0
3	Lutjegast	HPAI H5N8	2020-11-09	EPI_ISL_632319	EPI_ISL_693515	Maasland	2020-12-04	-25	197	12
4	Terwolde	HPAI H5N8	2020-11-13	EPI_ISL_641518	EPI_ISL_623075	Puiflijk*	2020-11-04	9	56	12
5	Witmarsum	HPAI H5N8	2020-11-20	EPI_ISL_653920	EPI_ISL_623075	Puiflijk*	2020-11-04	16	136	56
6	Hekendorp	HPAI H5N8	2020-11-21	EPI_ISL_653921	EPI_ISL_623075	Puiflijk*	2020-11-04	17	54	17
7	Maasland	HPAI H5N8	2020-12-04	EPI_ISL_693515	EPI_ISL_632319	Lutjegast	2020-11-09	25	197	12
8	St Annaparochie	HPAI H5N8	2020-12-06	EPI_ISL_710539	EPI_ISL_623075	Puiflijk*	2020-11-04	32	155	67
10	Moergestel	HPAI H5N8	2021-01-04	EPI_ISL_775267	EPI_ISL_1046889	Sint-Oedenrode	2021-02-19	-46	18	10
11	St Oedenrode	HPAI H5N8	2021-02-19	EPI_ISL_1046889	EPI_ISL_775267	Moergestel	2021-01-04	46	18	10
12	Weert	HPAI H5N8	2021-05-21	EPI_ISL_2227275	EPI_ISL_775267	Moergestel	2021-01-04	137	47	58

* The virus was last detected at Puiflijk, but is identical to the virus in Alforst

Table S4: closest related virus isolated from a commercial poulty farm

Farm	Location	Virus subtype	Sample date	Poultry virus	Closest related wild bird virus	Wild bird species	Sample date	Days since detection closest wild bird virus	Distance between locations (km)	Number of nucleotide differences
1	Altforst	HPAI H5N8	2020-10-28	EPI_ISL_603132	EPI_ISL_1139017	Mute Swan	2020-10-28	0	58	4
					EPI_ISL_1139080	Barnacle Goose	2020-10-29	-1	178	4
2	Puiflijk	HPAI H5N8	2020-11-04	EPI_ISL_623075	EPI_ISL_1139017	Mute Swan	2020-10-28	7	59	4
					EPI_ISL_1139080	Barnacle Goose	2020-10-29	6	177	4
3	Lutjegast	HPAI H5N8	2020-11-09	EPI_ISL_632319	EPI_ISL_1139080	Barnacle Goose	2020-10-29	11	38	5
4	Terwolde	HPAI H5N8	2020-11-13	EPI_ISL_641518	EPI_ISL_1139080	Barnacle Goose	2020-10-29	15	133	8
5	Witmarsum	HPAI H5N8	2020-11-20	EPI_ISL_653920	EPI_ISL_1139109	Mute Swan	2020-11-24	-4	2	3
6	Hekendorp	HPAI H5N8	2020-11-21	EPI_ISL_653921	EPI_ISL_1139080	Barnacle Goose	2020-10-29	23	172	13
7	Maasland	HPAI H5N8	2020-12-04	EPI_ISL_693515	EPI_ISL_1139080	Barnacle Goose	2020-10-29	36	195	7
8	St Annaparochie	HPAI H5N8	2020-12-06	EPI_ISL_710539	EPI_ISL_1139172	Mute Swan	2020-11-26	10	196	10
9	*Buitenpost*	*HPAI H5N1*	*2020-12-14*	*EPI_ISL_711055*	*EPI_ISL_632314*	*Greylag Goose*	*2020-10-28*	*47*	*114*	*8*
10	Moergestel	HPAI H5N8	2021-01-04	EPI_ISL_775267	EPI_ISL_1139096	goose	2020-11-01	64	68	39
11	St Oedenrode	HPAI H5N8	2021-02-19	EPI_ISL_1046889	EPI_ISL_1139096	goose	2020-11-01	110	72	47
12	Weert	HPAI H5N8	2021-05-21	EPI_ISL_2227275	EPI_ISL_1139096	goose	2020-11-01	201	108	73

Table S5: acknowledgment of all contributers to the GISAID's EpiFlu™ Database

Segment ID	Segment	Country	Collection date	Isolate-ID	Isolate name	Originating Lab	Submitting Lab	Authors
EPI1774527	PB2	Belgium	2018-Apr-09	EPI_ISL_502614	A/Anas platyrhynchos/Belgium/7976/2018		Import from public-domain	Lambrecht,B.; Steensels,M.; Fusaro,A.; Milani,A.; Pastori,A.; Schivo,A.; Salviato,A.; Zamperin,G.; Monne,I.; Terregino,C.
EPI1818105	PB2	Mongolia	2018-Sep-02	EPI_ISL_697696	A/duck/Mongolia/451/2018		Import from public-domain	Sakoda,Y.; Okamatsu,M.; Matsuno,K.
EPI1581322	PB1	Egypt	2016-Mar-17	EPI_ISL_387973	A/northern shoveler/Egypt/MB-D-695C/2016		Import from public-domain	Direct Submission
EPI1818050	PB1	Mongolia	2018-Sep-02	EPI_ISL_697689	A/duck/Mongolia/217/2018		Import from public-domain	Sakoda,Y.; Okamatsu,M.; Matsuno,K.
EPI1818106	PB1	Mongolia	2018-Sep-02	EPI_ISL_697696	A/duck/Mongolia/451/2018		Import from public-domain	Sakoda,Y.; Okamatsu,M.; Matsuno,K.
EPI1841791	PB1	Netherlands	2019-Jan-23	EPI_ISL_819124	A/mallard/Netherlands/19001282-001/2019	Wageningen Bioveterinary Research	Wageningen Bioveterinary Research	Beerens, Nancy; Harders, Frank; Pritz-Verschuren, Sylvia; Roose, Marit; Germeraad, Evelien; Engelsma, Marc; Bossers, Alex; Heutink, Rene
EPI1019770	PA	Netherlands	2016-Nov-08	EPI_ISL_268669	A/T_Dk/NL-Monnickendam/16013865-006-008/2016	Wageningen Bioveterinary Research	Wageningen Bioveterinary Research	Beerens, Nancy; Heutink, Rene; Harders, Frank; Verschuren-Pritz, Sylvia; Bossers, Alex; Koch, Guus; Bergervoet, Saskia
EPI1328453	PA	Russian Fede	2016-Sep-03	EPI_ISL_332682	A/teal/Toguchin/1157/2016	State Research Center of Virology and Biotechnology (VECTOR)	State Research Center of Virology and Biotechnology (VECTOR)	Natalia,Goncharova; Ivan,Susloparov; Natalia,Kolosova; Vasiliy,Marchenko; Alexander,Ryzhikov
EPI1440603	PA	Russian Fede	2018-Aug-26	EPI_ISL_355937	A/green sandpiper/Kurgan/1048/2018	State Research Center of Virology and Biotechnology (VECTOR)	State Research Center of Virology and Biotechnology (VECTOR)	Natalia,Goncharova; Ivan,Susloparov; Natalia,Kolosova; Alexey,Danilenko; Juliya,Bulanovich; Vasiliy,Marchenko; Alexander,Ryzhikov
EPI1774421	PA	Belgium	2018-Oct-12	EPI_ISL_502607	A/Anas platyrhynchos/Belgium/195_7/2018		Import from public-domain	Lambrecht,B.; Steensels,M.; Fusaro,A.; Milani,A.; Pastori,A.; Schivo,A.; Salviato,A.; Zamperin,G.; Monne,I.; Terregino,C.
EPI1777588	PA	Mongolia	2019-Sep-20	EPI_ISL_503358	A/duck/Mongolia/876/2019		Import from public-domain	Sakoda,Y.; Okamatsu,M.; Matsuno,K.; Enkhbold,B.
EPI1850204	HA	Poland	2020-Dec-09	EPI_ISL_1220094	A/tundra_bean_goose/Poland/MB132/2020	National Veterinary Research Institut Poland, PIWet-PIB	National Veterinary Research Institut Poland, PIWet-PIB	Swieton E., Smietanka K.
EPI1860127	HA	Romania	2021-Jan-19	EPI_ISL_2234819	A/goose/Romania/10330_21VIR2593-35/2021	Istituto Zooprofilattico Sperimentale delle Venezie, EU/OIE/Reference Laboratory and FAO Reference Centre for AI and ND	Istituto Zooprofilattico Sperimentale Delle Venezie	Onita, I.; Neicut, A.; Raluca, B.; Razvan, M.; Florica, B.; Zecchin, B.; Fusaro, A.; Giussani, E.; Schivo, A.; Salviato, A.; Monne, I.; Terregino, C.
EPI1860119	HA	Romania	2021-Jan-13	EPI_ISL_2234818	A/goose/Romania/10205-t5_21VIR2593-8/2021	Istituto Zooprofilattico Sperimentale delle Venezie, EU/OIE/Reference Laboratory and FAO Reference Centre for AI and ND	Istituto Zooprofilattico Sperimentale Delle Venezie	Onita, I.; Neicut, A.; Raluca, B.; Razvan, M.; Florica, B.; Zecchin, B.; Fusaro, A.; Giussani, E.; Schivo, A.; Salviato, A.; Monne, I.; Terregino, C.
EPI1858468	HA	Czech Republ	2021-Apr-02	EPI_ISL_1697190	A/peacock/Czech Republic/6529-2/2021	State Veterinary Institute Prague	State Veterinary Institute Prague	Nagy,A;Cernikova,L;Stara,M
EPI1858590	HA	Lithuania	2021-Mar-19	EPI_ISL_1719911	A/swan/Lithuania/1842PG1_21VIR2606-5/2021	Istituto Zooprofilattico Sperimentale delle Venezie, EU/OIE/Reference Laboratory and FAO Reference Centre for AI and ND	Istituto Zooprofilattico Sperimentale Delle Venezie	Pridotkas, G.; Jurgelevicius, V.; Pileviciene, S.; Zecchin, B.; Fusaro, A.; Milani, A.; Schivo, A.; Salviato, A.; Giussani, E.; Monne, I.; Terregino, C.
EPI1858582	HA	Lithuania	2021-Feb-26	EPI_ISL_1719910	A/swan/Lithuania/1298PG1_21VIR2606-3/2021	Istituto Zooprofilattico Sperimentale delle Venezie, EU/OIE/Reference Laboratory and FAO Reference Centre for AI and ND	Istituto Zooprofilattico Sperimentale Delle Venezie	Pridotkas, G.; Jurgelevicius, V.; Pileviciene, S.; Zecchin, B.; Fusaro, A.; Milani, A.; Schivo, A.; Salviato, A.; Giussani, E.; Monne, I.; Terregino, C.
EPI1858276	HA	Norway	2021-Jan-05	EPI_ISL_1665260	A/mute_swan/Norway/FU5_21VIR850-3/2021	Istituto Zooprofilattico Sperimentale delle Venezie, EU/OIE/Reference Laboratory and FAO Reference Centre for AI and ND	Istituto Zooprofilattico Sperimentale Delle Venezie	Madslien, K.; Moldal, T.; Gjerset, B.; Gudmundsson, S.; Follestad, A.; Tronerud, OH.; Dean, KR.; Akerstedt, J.; Jorgensen,HJ.; das Neves, CG.; Romo, G.; Zecchin, B.; Fusaro, A.; Pastori, A.; Schivo, A.; Salviato, A.; Monne, I.; Terregino, C.
EPI1859671	HA	Poland	2021-Mar-06	EPI_ISL_2114013	A/mute_swan/Poland/MB272/2021	National Veterinary Research Institut Poland, PIWet-PIB	National Veterinary Research Institute	Dziadek, K.; Swieton, E.; Smietanka, K.
EPI1883992	HA	Hungary	2021-Mar-08	EPI_ISL_3135931	A/swan/Hungary/9638/2021 (H5N8)	National Food Chain Safety Office Veterinary Diagnostic Directorate Laboratory for Molecular Biology	National Food Chain Safety Office, Hungary	Katalin,Szentpáli-Gavallér;Ádám,Bálint;Krisztina,Ursu;Péter,Malik
EPI1854355	HA	Czech Republ	2021-Mar-06	EPI_ISL_1399245	A/mute swan/Czech Republic/4799/2021	State Veterinary Institute Prague	State Veterinary Institute Prague	Nagy,A;Cernikova,L;Stara,M
EPI1854347	HA	Czech Republ	2021-Mar-04	EPI_ISL_1399244	A/mute swan/Czech Republic/4607-2/2021	State Veterinary Institute Prague	State Veterinary Institute Prague	Nagy,A;Cernikova,L;Stara,M
EPI1854339	HA	Czech Republ	2021-Mar-04	EPI_ISL_1399243	A/mute swan/Czech Republic/4607-1/2021	State Veterinary Institute Prague	State Veterinary Institute Prague	Nagy,A;Cernikova,L;Stara,M
EPI1854307	HA	Czech Republ	2021-Feb-23	EPI_ISL_1399239	A/mute swan/Czech Republic/4100/2021	State Veterinary Institute Prague	State Veterinary Institute Prague	Nagy,A;Cernikova,L;Stara,M
EPI1854291	HA	Czech Republ	2021-Feb-19	EPI_ISL_1399237	A/mute swan/Czech Republic/3549/2021	State Veterinary Institute Prague	State Veterinary Institute Prague	Nagy,A;Cernikova,L;Stara,M
EPI1847740	HA	Czech Republ	2021-Feb-04	EPI_ISL_1058019	A/mute swan/Czech Republic/2669-2/2021	State Veterinary Institute Prague	State Veterinary Institute Prague	Nagy,A; Cernikova,L; Stara,M
EPI1850128	HA	Czech Republ	2021-Jan-24	EPI_ISL_1180234	A/mute swan/Czech Republic/1656-1/2021	State Veterinary Institute Prague	State Veterinary Institute Prague	Nagy,A; Cernikova,L; Stara,M
EPI1851824	HA	Norway	2021-Jan-26	EPI_ISL_1295638	A/mute_swan/Norway/FU48/2021	Norwegian Veterinary Institute	Animal and Plant Health Agency (APHA)	Britt Gjerset, Torfinn Moldal
EPI1883070	HA	Denmark	2020-Nov-15	EPI_ISL_984695	A/chicken/Denmark/14819-6/2020	Statens Serumm Institute	Statens Serum Institute	Yuan Liang, Charlotte Hjulsager
EPI1883876	HA	Kosovo	2021-May-31	EPI_ISL_3128525	A/chicken/Kosovo/97_21VIR5162-15/2021	Istituto Zooprofilattico Sperimentale delle Venezie, EU/OIE/Reference Laboratory and FAO Reference Centre for AI and ND	Istituto Zooprofilattico Sperimentale Delle Venezie	Uka, K.; Cana, A.; Merovci, X.; Krstevski, K.; Zecchin, B.; Fusaro, A.; Giussani, E.; Schivo, A.; Salviato, A.; Palumbo, E.; Monne, I.; Terregino, C.
EPI1883541	HA	Poland	2021-Apr-16	EPI_ISL_3102071	A/chicken/Poland/H712_21RS1385-12/2021	Istituto Zooprofilattico Sperimentale delle Venezie, EU/OIE/Reference Laboratory and FAO Reference Centre for AI and ND	Istituto Zooprofilattico Sperimentale Delle Venezie	Smietanka, K.; Swieton, E.; Zecchin, B.; Fusaro, A.; Milani, A.; Schivo, A.; Salviato, A.; Giussani, E.; Monne, I.; Terregino, C.

EPI1883509	HA	Poland	2021-Mar-23	EPI_ISL_3102067	A/chicken/Poland/H293_21RS1385-8/2021	Istituto Zooprofilattico Sperimentale delle Venezie, EU/OIE/Reference Laboratory and FAO Reference Centre for AI and ND	Istituto Zooprofilattico Sperimentale Delle Venezie	Smietanka, K.; Swieton, E.; Zecchin, B.; Fusaro, A.; Milani, A.; Schivo, A.; Salviato, A.; Giussani, E.; Monne, I.; Terregino, C.
EPI1883501	HA	Poland	2021-Apr-20	EPI_ISL_3102066	A/chicken/Poland/H812_21RS1385-7/2021	Istituto Zooprofilattico Sperimentale delle Venezie, EU/OIE/Reference Laboratory and FAO Reference Centre for AI and ND	Istituto Zooprofilattico Sperimentale Delle Venezie	Smietanka, K.; Swieton, E.; Zecchin, B.; Fusaro, A.; Milani, A.; Schivo, A.; Salviato, A.; Giussani, E.; Monne, I.; Terregino, C.
EPI1883445	HA	Bulgaria	2021-May-05	EPI_ISL_3102059	A/chicken/Bulgaria/298-1_21VIR4270-9/2021	Istituto Zooprofilattico Sperimentale delle Venezie, EU/OIE/Reference Laboratory and FAO Reference Centre for AI and ND	Istituto Zooprofilattico Sperimentale Delle Venezie	Goujgoulova, G.; Slavcheva, I.; Zecchin, B.; Fusaro, A.; Milani, A.; Schivo, A.; Salviato, A.; Giussani, E.; Monne, I.; Terregino, C.
EPI1858622	HA	Bulgaria	2021-Feb-08	EPI_ISL_1719915	A/chicken/Bulgaria/50-1_21VIR1454-9/2021	Istituto Zooprofilattico Sperimentale delle Venezie, EU/OIE/Reference Laboratory and FAO Reference Centre for AI and ND	Istituto Zooprofilattico Sperimentale Delle Venezie	Goujgoulova, G.; Slavcheva, I.; Zecchin, B.; Fusaro, A.; Milani, A.; Schivo, A.; Salviato, A.; Giussani, E.; Monne, I.; Terregino, C.
EPI1858188	HA	Italy	2021-Feb-23	EPI_ISL_1665248	A/chicken/Italy/21VIR1293-9/2021	Istituto Zooprofilattico Sperimentale delle Venezie, EU/OIE/Reference Laboratory and FAO Reference Centre for AI and ND	Istituto Zooprofilattico Sperimentale Delle Venezie	Zecchin, B.; Fusaro, A.; Pastori, A.; Schivo, A.; Salviato, A.; Monne, I.; Terregino, C.
EPI1846305	HA	United Kingdo	2021-Jan-05	EPI_ISL_996003	A/chicken/Northern_Ireland/2021-000067_21VIR114-19/2021	AFBI - Agri-Food & Bioscience Institute	Istituto Zooprofilattico Sperimentale Delle Venezie	McMenamy, MJ.; Harkin, V.; Lemon, K.; Zecchin, B.; Fusaro, A.; Schivo, A.; Salviato, A.; Pastori, A.; Monne, I.; Terregino, C.
EPI1868472	HA	Czech Republ	2021-May-17	EPI_ISL_2402938	A/chicken/Czech Republic/10405/2021	State Veterinary Institute Prague	State Veterinary Institute Prague	Nagy,A;Cernikova,L;Stara,M
EPI1868456	HA	Czech Republ	2021-May-16	EPI_ISL_2402936	A/chicken/Czech Republic/10251-1/2021	State Veterinary Institute Prague	State Veterinary Institute Prague	Nagy,A;Cernikova,L;Stara,M
EPI1859513	HA	Czech Republ	2021-Apr-18	EPI_ISL_1941365	A/chicken/Czech Republic/7682-5/2021	State Veterinary Institute Prague	State Veterinary Institute Prague	Nagy,A;Cernikova,L;Stara,M
EPI1858420	HA	Czech Republ	2021-Apr-06	EPI_ISL_1697184	A/chicken/Czech Republic/6654/2021	State Veterinary Institute Prague	State Veterinary Institute Prague	Nagy,A;Cernikova,L;Stara,M
EPI1854259	HA	Czech Republ	2021-Feb-27	EPI_ISL_1399233	A/chicken/Czech Republic/4092-1/2021	State Veterinary Institute Prague	State Veterinary Institute Prague	Nagy,A;Cernikova,L;Stara,M
EPI1854243	HA	Czech Republ	2021-Feb-18	EPI_ISL_1399231	A/chicken/Czech Republic/3531-1/2021	State Veterinary Institute Prague	State Veterinary Institute Prague	Nagy,A;Cernikova,L;Stara,M
EPI1850047	HA	Russian Fede	2020-Dec-07	EPI_ISL_1185026	A/chicken/Astrakhan/2171-1/2020	Federal Centre for Animal Health (ARRIAH) OIE Regional Reference Laboratory	Federal Centre for Animal Health (ARRIAH)	N., Zinyakov; P., Zhestkov; A., Andriyasov; A., Kozlov; E., Ovchinnikova; Z., Nikonova; V., Sosipatorova; L., Scherbakova; D., Andreychuk; I., Chvala
EPI1813077	HA	Netherlands	2020-Nov-10	EPI_ISL_641394	A/chicken/Netherlands/20017639-001/2020	Wageningen Bioveterinary Research	Wageningen Bioveterinary Research	Beerens, Nancy; Harders, Frank; Verschuren-Pritz, Sylvia; Roose, Marit; Germeraad, Evelien; Engelsma, Marc; Bossers, Alex; Heutink, Rene
EPI1883892	HA	Kosovo	2021-Jun-02	EPI_ISL_3128527	A/duck/Kosovo/107_21VIR5162-17/2021	Istituto Zooprofilattico Sperimentale delle Venezie, EU/OIE/Reference Laboratory and FAO Reference Centre for AI and ND	Istituto Zooprofilattico Sperimentale Delle Venezie	Uka, K.; Cana, A.; Merovci, X.; Krstevski, K.; Zecchin, B.; Fusaro, A.; Giussani, E.; Schivo, A.; Salviato, A.; Palumbo, E.; Monne, I.; Terregino, C.
EPI1883589	HA	Poland	2021-Apr-07	EPI_ISL_3102077	A/duck/Poland/H514_21RS1385-18/2021	Istituto Zooprofilattico Sperimentale delle Venezie, EU/OIE/Reference Laboratory and FAO Reference Centre for AI and ND	Istituto Zooprofilattico Sperimentale Delle Venezie	Smietanka, K.; Swieton, E.; Zecchin, B.; Fusaro, A.; Milani, A.; Schivo, A.; Salviato, A.; Giussani, E.; Monne, I.; Terregino, C.
EPI1860095	HA	Romania	2021-Jan-13	EPI_ISL_2234815	A/duck/Romania/10202_21VIR2593-31/2021	Istituto Zooprofilattico Sperimentale delle Venezie, EU/OIE/Reference Laboratory and FAO Reference Centre for AI and ND	Istituto Zooprofilattico Sperimentale Delle Venezie	Onita, I.; Neicut, A.; Raluca, B.; Razvan, M.; Florica, B.; Zecchin, B.; Fusaro, A.; Giussani, E.; Schivo, A.; Salviato, A.; Monne, I.; Terregino, C.
EPI1858614	HA	Bulgaria	2021-Feb-04	EPI_ISL_1719914	A/pekin_duck/Bulgaria/48-3_21VIR1454-7/2021	Istituto Zooprofilattico Sperimentale delle Venezie, EU/OIE/Reference Laboratory and FAO Reference Centre for AI and ND	Istituto Zooprofilattico Sperimentale Delle Venezie	Goujgoulova, G.; Slavcheva, I.; Zecchin, B.; Fusaro, A.; Milani, A.; Schivo, A.; Salviato, A.; Giussani, E.; Monne, I.; Terregino, C.
EPI1858244	HA	Slovakia	2021-Jan-08	EPI_ISL_1665256	A/muscovy_duck/Slovakia/Pah1_21VIR1086-1/2021	Istituto Zooprofilattico Sperimentale delle Venezie, EU/OIE/Reference Laboratory and FAO Reference Centre for AI and ND	Istituto Zooprofilattico Sperimentale Delle Venezie	Dirb?kov?, Z.; Tin?k, M.; Zecchin, B.; Fusaro, A.; Pastori, A.; Schivo, A.; Salviato, A.; Monne, I.; Terregino, C.
EPI1858540	HA	Czech Republ	2021-Mar-18	EPI_ISL_1697199	A/duck/Czech Republic/5360-2/2021	State Veterinary Institute Prague	State Veterinary Institute Prague	Nagy,A;Cernikova,L;Stara,M
EPI1883629	HA	Romania	2021-Apr-08	EPI_ISL_3102082	A/mute_swan/Romania/11981-2_21VIR3163-6/2021	Istituto Zooprofilattico Sperimentale delle Venezie, EU/OIE/Reference Laboratory and FAO Reference Centre for AI and ND	Istituto Zooprofilattico Sperimentale Delle Venezie	Onita, I.; Neicut, A.; Raluca, B.; Razvan, M.; Florica, B.; Zecchin, B.; Fusaro, A.; Giussani, E.; Schivo, A.; Salviato, A.; Monne, I.; Terregino, C.
EPI1860055	HA	Italy	2021-Jan-28	EPI_ISL_2234810	A/seagull/Italy/21VIR2479/2021	Istituto Zooprofilattico Sperimentale delle Venezie, EU/OIE/Reference Laboratory and FAO Reference Centre for AI and ND	Istituto Zooprofilattico Sperimentale Delle Venezie	Zecchin, B.; Fusaro, A.; Schivo, A.; Salviato, A.; Giussani, E.; Monne, I.; Terregino, C.
EPI1858404	HA	Czech Republ	2021-Mar-25	EPI_ISL_1697182	A/chicken/Czech Republic/5903/2021	State Veterinary Institute Prague	State Veterinary Institute Prague	Nagy,A;Cernikova,L;Stara,M
EPI1847764	HA	Czech Republ	2021-Feb-12	EPI_ISL_1058022	A/chicken/Czech Republic/3099-2/2021	State Veterinary Institute Prague	State Veterinary Institute Prague	Nagy,A; Cernikova,L; Stara,M
EPI1848790	HA	Russian Fede	2021-Jan-05	EPI_ISL_1114761	A/chicken/Krasnodar/334-02/2021	State Research Center of Virology and Biotechnology (VECTOR)	State Research Center of Virology and Biotechnology (VECTOR)	Natalia,Goncharova; Ivan,Susloparov; Natalia,Kolosova; Alexey,Danilenko; Juliya,Bulanovich; Vasiliy,Marchenko; Alexander,Ryzhikov

EPI1848670	HA	Russian Fede	2020-Oct-25	EPI_ISL_1114746	A/chicken/Rostov-on-Don/308-02/2020	State Research Center of Virology and Biotechnology (VECTOR)	State Research Center of Virology and Biotechnology (VECTOR)	Natalia,Goncharova; Ivan,Susloparov; Natalia,Kolosova; Alexey,Danilenko; Juliya,Bulanovich; Vasiliy,Marchenko; Alexander,Ryzhikov
EPI1848606	HA	Russian Fede	2020-Sep-26	EPI_ISL_1114735	A/chicken/Tyumen/302-01/2020	State Research Center of Virology and Biotechnology (VECTOR)	State Research Center of Virology and Biotechnology (VECTOR)	Natalia,Goncharova; Ivan,Susloparov; Natalia,Kolosova; Alexey,Danilenko; Juliya,Bulanovich; Vasiliy,Marchenko; Alexander,Ryzhikov
EPI1814345	HA	Russian Fede	2020-Aug-27	EPI_ISL_654834	A/chicken/Kurgan/1001/2020	State Research Center of Virology and Biotechnology (VECTOR)	State Research Center of Virology and Biotechnology (VECTOR)	Natalia,Goncharova; Ivan,Susloparov; Natalia,Kolosova; Alexey,Danilenko; Juliya,Bulanovich; Vasiliy,Marchenko; Alexander,Ryzhikov
EPI1814281	HA	Russian Fede	2020-Sep-03	EPI_ISL_654826	A/goose/Omsk/30001/2020	State Research Center of Virology and Biotechnology (VECTOR)	State Research Center of Virology and Biotechnology (VECTOR)	Natalia,Goncharova; Ivan,Susloparov; Natalia,Kolosova; Alexey,Danilenko; Juliya,Bulanovich; Vasiliy,Marchenko; Alexander,Ryzhikov
EPI1848750	HA	Russian Fede	2021-Jan-29	EPI_ISL_1114756	A/turkey/Rostov-on-Don/332-08/2021	State Research Center of Virology and Biotechnology (VECTOR)	State Research Center of Virology and Biotechnology (VECTOR)	Natalia,Goncharova; Ivan,Susloparov; Natalia,Kolosova; Alexey,Danilenko; Juliya,Bulanovich; Vasiliy,Marchenko; Alexander,Ryzhikov
EPI1883029	HA	Denmark	2020-Nov-06	EPI_ISL_984690	A/peregrine falcon/Denmark/14596-1/2020	Statens Serumm Institute	Statens Serum Institute	Yuan Liang, Charlotte Hjulsager
EPI1811619	HA	Kazakhstan	2020-Sep-19	EPI_ISL_615073	A/domestic_goose/Kazakhstan/1-242_2-20-B/2020	National Veterinary Reference Center	Animal and Plant Health Agency (APHA)	
EPI1811601	HA	Kazakhstan	2020-Sep-20	EPI_ISL_615068	A/domestic_goose/Kazakhstan/1-248_2-20-B/2020	National Veterinary Reference Center	Animal and Plant Health Agency (APHA)	
EPI1271003	HA	Russian Fede	2018-Jul-04	EPI_ISL_320683	A/goose/Samara/673/2018		State Research Center of Virology and Biotechnology (VECTOR)	Alexey,Danilenko; Vasiliy,Marchenko; Ivan,Susloparov; Natalya,Goncharova; Natalya,Kolosova; Juliya, Bulanovich; Alexander,Ryzhikov
EPI1848926	HA	United Kingdc	2020-Dec-01	EPI_ISL_1123360	A/mute_swan/England/234135/2020	Animal and Plant Health Agency (APHA)	Animal and Plant Health Agency (APHA)	
EPI1848918	HA	United Kingdc	2020-Nov-10	EPI_ISL_1123359	A/mute swan/England/263814/2020	Animal and Plant Health Agency (APHA)	Animal and Plant Health Agency (APHA)	
EPI1850226	HA	Poland	2020-Dec-07	EPI_ISL_846604	A/turkey/Poland/477/2020(H5N8)	National Veterinary Research Institut Poland, PIWet-PIB	National Veterinary Research Institut Poland, PIWet-PIB	Swieton, E.; Smietanka, K.
EPI552746	HA	Germany	2014-Nov-04	EPI_ISL_169273	A/turkey/Germany/AR2485-86-L00899/2014		Friedrich-Loeffler-Institut	
EPI553144	HA	Italy	2014-Dec-15	EPI_ISL_169350	A/turkey/Italy/14VIR7898-10/2014	Istituto Zooprofilattico Sperimentale Delle Venezie	Istituto Zooprofilattico Sperimentale Delle Venezie	Luca,Tassoni; Silvia,Ormelli; Alessia,Schivo; Alice,Fusaro; Isabella,Monne; Giovanni,Cattoli
EPI959523	HA	Hungary	2017-Feb-15	EPI_ISL_255933	A/Cormorant/Hungary/6102/2017	National Food Chain Safety Office Veterinary Diagnostic Directorate Laboratory for Molecular Biology	Danam.Vet.Molbiol	Adam, Dan
EPI1721922	HA	Bulgaria	2018-Jul-05	EPI_ISL_419361	A/chicken/Bulgaria/Dobrich/163-2/2018	NDRVMI (National Diagnostic and Research Veterinary Medical Institute)	University of Cambridge	
EPI1721906	HA	Bulgaria	2018-Jun-12	EPI_ISL_419359	A/chicken/Bulgaria/Dobrich/115/2018	NDRVMI (National Diagnostic and Research Veterinary Medical Institute)	University of Cambridge	
EPI1721786	HA	Bulgaria	2018-Mar-02	EPI_ISL_419344	A/chicken/Bulgaria/Dobrich/12-2/2018	NDRVMI (National Diagnostic and Research Veterinary Medical Institute)	University of Cambridge	
EPI1040223	HA	Italy	2017-Apr-07	EPI_ISL_273846	A/chicken/Italy/17VIR3078/2017	Istituto Zooprofilattico Sperimentale Delle Venezie	Istituto Zooprofilattico Sperimentale Delle Venezie	Bianca,Zecchin; Alice,Fusaro; Gianpiero,Zamperin; Alessia,Schivo; Annalisa,Salviato; Sabrina,Marciano; Silvia,Ormelli; Calogero,Terregino; Isabella,Monne
EPI1811628	HA	Iraq	2020-May-12	EPI_ISL_623074	A/chicken/Iraq/1/2020	Central Veterinary Labs	Animal and Plant Health Agency (APHA)	
EPI1839261	HA	Kazakhstan	2020-Sep-18	EPI_ISL_739686	A/chicken/Kazakhstan/Kn-3/2020	Research Institute of Experimental and Clinical Medicine	WHO National Influenza Centre Russian Federation	Sobolev, I.; Sharshov, K.; Dubovitskiy, N.; Alekseev, A.; Leonov, S.; Irza, V.; Fadeev, A.; Danilenko, D.; Komissarov, A.; Shestopalov, A.
EPI573163	HA	Netherlands	2014-Nov-19	EPI_ISL_174349	A/chicken/Netherlands/14015766/2014	Wageningen Bioveterinary Research	Wageningen Bioveterinary Research	Heutink, Rene; Harders, Frank; Verschuren-Pritz, Sylvia; Bossers, Alex; Koch, Guus; Bouwstra, Ruth
EPI548623	HA	Netherlands	2014-Nov-15	EPI_ISL_168075	A/chicken/Netherlands/14015531/2014	Wageningen Bioveterinary Research	Wageningen Bioveterinary Research	Heutink, Rene; Harders, Frank; Verschuren-Pritz, Sylvia; Bossers, Alex; Koch, Guus; Bouwstra, Ruth

EPI1812533	HA	Russian Fede	2020-Jul-31	EPI_ISL_637098	A/duck/Chelyabinsk/1207-1/2020	Federal Centre for Animal Health (ARRIAH) OIE Regional Reference Laboratory	Federal Centre for Animal Health (ARRIAH)	N., Zinyakov; P., Akshalova; P., Zhestkov; A., Kozlov; A., Andriyasov; E., Ovchinnikova; Z., Nikonova; V., Sosipatorova; L., Scherbakova; D., Andreychuk; I., Chvala
EPI1318870	HA	United Kingdc	2014-Nov-16	EPI_ISL_331223	A/Duck/England/1279/2014	Animal and Plant Health Agency (APHA)	Animal and Plant Health Agency (APHA)	Puranik,Anita; Warren,Caroline; Mahmood,Sahar; Thomas,Saumya; Byrne,Alexander; Ramsay,Andrew; Everett,Helen; Skinner,Paul; Núñez,Alejandro; Watson,Samantha; Slomka,Marek; Brown,Ian; Brookes,Sharon
EPI1811611	HA	Kazakhstan	2020-Sep-25	EPI_ISL_615072	A/domestic_duck/Kazakhstan/1-274-20-B/2020	National Veterinary Reference Center	Animal and Plant Health Agency (APHA)	
EPI959413	HA	Netherlands	2016-Dec-09	EPI_ISL_255912	A/Eurasian Wigeon/Netherlands/4/2016	Erasmus Medical Center	Erasmus Medical Center	Poen,M.J.;Van Der Jeugd,H.P.;Vuong,O.;Scheuer,R.D.;Kleyheeg,E.;Lexmond,P.; Eggink,W.D.;Müskens,G.J.D.M;Bestebroer,T.M.;Koopmans,M.P.G.; Kuiken,T.;Fouchier,R.A.M.
EPI1023580	HA	Netherlands	2016-Dec-05	EPI_ISL_269703	A/Eurasian_Wigeon/Netherlands/25/2016		Erasmus Medical Center	Poen,M.J.;Van Der Jeugd,H.P.;Vuong,O.;Scheuer,R.D.;Kleyheeg,E.; Bestebroer,T.M.;Begeman,L;van den Brand,J.M.A.;Kuiken,T.;Fouchier,R.A.M.
EPI1019638	HA	Netherlands	2016-Dec-10	EPI_ISL_268652	A/Eur_Wig/NL-Zoeterwoude/16015702-010/2016	Wageningen Bioveterinary Research	Wageningen Bioveterinary Research	Beerens, Nancy; Heutink, Rene; Harders, Frank; Verschuren-Pritz, Sylvia; Bossers, Alex; Koch, Guus; Bergervoet, Saskia
EPI1019534	HA	Netherlands	2016-Dec-11	EPI_ISL_268639	A/Eur_Wig/NL-Enumatil-Groningen/16015704-001/2016	Wageningen Bioveterinary Research	Wageningen Bioveterinary Research	Beerens, Nancy; Heutink, Rene; Harders, Frank; Verschuren-Pritz, Sylvia; Bossers, Alex; Koch, Guus; Bergervoet, Saskia
EPI1270858	HA	Russian Fede	2018-Jun-22	EPI_ISL_320659	A/duck/Samara/452/2018		State Research Center of Virology and Biotechnology (VECTOR)	Alexey,Danilenko; Vasiliy,Marchenko; Ivan,Susloparov; Natalya,Goncharova; Natalya,Kolosova; Juliya, Bulanovich; Alexander,Ryzhikov
EPI1023572	HA	Netherlands	2016-Dec-20	EPI_ISL_269603	A/Mallard/Netherlands/51/2016	Erasmus Medical Center	Erasmus Medical Center	Poen,M.J.;Van Der Jeugd,H.P.;Vuong,O.;Scheuer,R.D.;Kleyheeg,E.; Bestebroer,T.M.;Begeman,L;van den Brand,J.M.A.;Kuiken,T.;Fouchier,R.A.M.
EPI573179	HA	Netherlands	2014-Nov-21	EPI_ISL_174351	A/duck/Netherlands/14015989/2014	Wageningen Bioveterinary Research	Wageningen Bioveterinary Research	Heutink, Rene; Harders, Frank; Verschuren-Pritz, Sylvia; Bossers, Alex; Koch, Guus; Bouwstra, Ruth
EPI1815150	HA	Italy	2020-Nov-14	EPI_ISL_683593	A/Eurasian_wigeon/Italy/20VIR7139-121/2020	Istituto Zooprofilattico Sperimentale delle Venezie, EU/OIE/Reference Laboratory and FAO Reference Centre for AI and ND	Istituto Zooprofilattico Sperimentale Delle Venezie	Zecchin, B.; Fusaro, A.; Pastori, A.; Milani, A.; Salviato, A.; Schivo, A.; Monne, I.; Terregino, C.
EPI1843634	HA	Italy	2020-Nov-29	EPI_ISL_956411	A/greylag_goose/Italy/20VIR7660-6/2020	Istituto Zooprofilattico Sperimentale delle Venezie, EU/OIE/Reference Laboratory and FAO Reference Centre for AI and ND	Istituto Zooprofilattico Sperimentale Delle Venezie	Zecchin, B.; Fusaro, A.; Milani, A.; Schivo, A.; Salviato, A.; Pastori, A.; Zamperin, G.; Monne, I.; Terregino, C.
EPI1883054	HA	Denmark	2020-Nov-07	EPI_ISL_984693	A/barnacle goose/Denmark/14599-1/2020	Statens Serumm Institute	Statens Serum Institute	Yuan Liang, Charlotte Hjulsager
EPI1883013	HA	Denmark	2020-Nov-05	EPI_ISL_984686	A/barnacle goose/Denmark/14536-1/2020	Statens Serumm Institute	Statens Serum Institute	Yuan Liang, Charlotte Hjulsager
EPI1882979	HA	Denmark	2020-Nov-04	EPI_ISL_984675	A/barnacle goose/Denmark/14139-3/2020	Statens Serumm Institute	Statens Serum Institute	Yuan Liang, Charlotte Hjulsager
EPI1848654	HA	Russian Fede	2020-Oct-17	EPI_ISL_1114742	A/chicken/Kostroma/304-08/2020	State Research Center of Virology and Biotechnology (VECTOR)	State Research Center of Virology and Biotechnology (VECTOR)	Natalia,Goncharova; Ivan,Susloparov; Natalia,Kolosova; Alexey,Danilenko; Juliya,Bulanovich; Vasiliy,Marchenko; Alexander,Ryzhikov
EPI1813409	HA	Russian Fede	2020-Aug-17	EPI_ISL_644158	A/chicken/Omsk/0073/2020	State Research Center of Virology and Biotechnology (VECTOR)	State Research Center of Virology and Biotechnology (VECTOR)	Natalia,Goncharova; Ivan,Susloparov; Natalia,Kolosova; Alexey,Danilenko; Juliya,Bulanovich; Vasiliy,Marchenko; Alexander,Ryzhikov
EPI1351443	HA	Russian Fede	2018-Nov-02	EPI_ISL_336933	A/chicken/Voronezh/1513/2018		State Research Center of Virology and Biotechnology (VECTOR)	Natalya,Goncharova; Alexey,Danilenko; Vasiliy,Marchenko; Ivan,Susloparov; Natalya,Kolosova; Juliya, Bulanovich; Alexander,Ryzhikov
EPI1351435	HA	Russian Fede	2018-Nov-02	EPI_ISL_336932	A/chicken/Voronezh/1504/2018		State Research Center of Virology and Biotechnology (VECTOR)	Natalya,Goncharova; Alexey,Danilenko; Vasiliy,Marchenko; Ivan,Susloparov; Natalya,Kolosova; Juliya, Bulanovich; Alexander,Ryzhikov
EPI1271019	HA	Russian Fede	2018-Jul-04	EPI_ISL_320685	A/chicken/Samara/679/2018		State Research Center of Virology and Biotechnology (VECTOR)	Alexey,Danilenko; Vasiliy,Marchenko; Ivan,Susloparov; Natalya,Goncharova; Natalya,Kolosova; Juliya, Bulanovich; Alexander,Ryzhikov
EPI1270987	HA	Russian Fede	2018-Jun-26	EPI_ISL_320681	A/chicken/Kursk/760/2018		State Research Center of Virology and Biotechnology (VECTOR)	Alexey,Danilenko; Vasiliy,Marchenko; Ivan,Susloparov; Natalya,Goncharova; Natalya,Kolosova; Juliya, Bulanovich; Alexander,Ryzhikov
EPI1270971	HA	Russian Fede	2018-Jul-16	EPI_ISL_320679	A/chicken/Cheboksary/806/2018		State Research Center of Virology and Biotechnology (VECTOR)	Alexey,Danilenko; Vasiliy,Marchenko; Ivan,Susloparov; Natalya,Goncharova; Natalya,Kolosova; Juliya, Bulanovich; Alexander,Ryzhikov

EPI1270882	HA	Russian Fede	2018-Jun-15	EPI_ISL_320662	A/chicken/Penza/300/2018		State Research Center of Virology and Biotechnology (VECTOR)	Alexey,Danilenko; Vasiliy,Marchenko; Ivan,Susloparov; Natalya,Goncharova; Natalya,Kolosova; Juliya, Bulanovich; Alexander,Ryzhikov
EPI1813393	HA	Russian Fede	2020-Aug-17	EPI_ISL_644156	A/goose/Omsk/011101/2020	State Research Center of Virology and Biotechnology (VECTOR)	State Research Center of Virology and Biotechnology (VECTOR)	Natalia,Goncharova; Ivan,Susloparov; Natalia,Kolosova; Alexey,Danilenko; Juliya,Bulanovich; Vasiliy,Marchenko; Alexander,Ryzhikov
EPI1813273	HA	Russian Fede	2020-Aug-17	EPI_ISL_644141	A/goose/Omsk/0074/2020	State Research Center of Virology and Biotechnology (VECTOR)	State Research Center of Virology and Biotechnology (VECTOR)	Natalia,Goncharova; Ivan,Susloparov; Natalia,Kolosova; Alexey,Danilenko; Juliya,Bulanovich; Vasiliy,Marchenko; Alexander,Ryzhikov
EPI1813121	HA	Russian Fede	2020-Aug-13	EPI_ISL_644122	A/goose/Omsk/0002/2020	State Research Center of Virology and Biotechnology (VECTOR)	State Research Center of Virology and Biotechnology (VECTOR)	Natalia,Goncharova; Ivan,Susloparov; Natalia,Kolosova; Alexey,Danilenko; Juliya,Bulanovich; Vasiliy,Marchenko; Alexander,Ryzhikov
EPI1843618	HA	Italy	2020-Dec-04	EPI_ISL_956408	A/common_teal/Italy/20VIR7608-73/2020	Istituto Zooprofilattico Sperimentale delle Venezie, EU/OIE/Reference Laboratory and FAO Reference Centre for AI and ND	Istituto Zooprofilattico Sperimentale Delle Venezie	Zecchin, B.; Fusaro, A.; Milani, A.; Schivo, A.; Salviato, A.; Pastori, A.; Zamperin, G.; Monne, I.; Terregino, C.
EPI1813113	HA	Russian Fede	2020-Aug-13	EPI_ISL_644121	A/turkey/Omsk/0001/2020	State Research Center of Virology and Biotechnology (VECTOR)	State Research Center of Virology and Biotechnology (VECTOR)	Natalia,Goncharova; Ivan,Susloparov; Natalia,Kolosova; Alexey,Danilenko; Juliya,Bulanovich; Vasiliy,Marchenko; Alexander,Ryzhikov
EPI909436	HA	Russian Fede	2017-Jan-06	EPI_ISL_247722	A/goose/Krasnodar/3144/2017		State Research Center of Virology and Biotechnology (VECTOR)	Ivan,Susloparov; Natalya,Goncharova; Natalya,Kolosova; Vasiliy,Marchenko; Alexander,Ryzhikov
EPI691836	HA	Germany	2014-Dec-01	EPI_ISL_206773	A/gull/Germany-NI/R45/2015		Friedrich-Loeffler-Institut	
EPI954559	HA	Italy	2017-Jan-19	EPI_ISL_255181	A/swan/Italy/17VIR537-2/2017	Istituto Zooprofilattico Sperimentale Delle Venezie	Istituto Zooprofilattico Sperimentale Delle Venezie	Zecchin, B.; Fusaro, A.; Milani, A.; Schivo, A.; Salviato, A.; Zamperin, G.; Marciano, S.; Ormelli, S.; Terregino, C.; Monne, I.
EPI1498937	HA	United Kingdc	2016-Dec-30	EPI_ISL_366241	A/Cygnus olor/England/WULH/2016		Import from public-domain	Hill,S.C.; Hansen,R.; Watson,S.; Coward,V.; Russell,C.; Cooper,J.; Essen,S.; Everest,H.; Parag,K.V.; Fiddaman,S.; Reid,S.; Lewis,N.; Brookes,S.M.; Smith,A.L.; Sheldon,B.; Perrins,C.M.; Brown,I.H.; Pybus,O.G.
EPI1498932	HA	United Kingdc	2016-Dec-31	EPI_ISL_366238	A/Cygnus olor/England/WVJX/2016		Import from public-domain	Hill,S.C.; Hansen,R.; Watson,S.; Coward,V.; Russell,C.; Cooper,J.; Essen,S.; Everest,H.; Parag,K.V.; Fiddaman,S.; Reid,S.; Lewis,N.; Brookes,S.M.; Smith,A.L.; Sheldon,B.; Perrins,C.M.; Brown,I.H.; Pybus,O.G.
EPI1226452	HA	Belgium	2017-Mar-21	EPI_ISL_309195	A/Cygnus olor/Belgium/2967/2017		Import from public-domain	Van Borm,S.; Vandenbussche,F.; Mathijs,E.; Lambrecht,B.; Steensels,M.
EPI1127538	HA	Japan	2017-Nov-05	EPI_ISL_289173	A/mute swan/Shimane/3211A001/2017		Import from public-domain	Soda,K.; Ito,H.; Usui,T.; Ozaki,H.; Murase,T.; Yamaguchi,T.; Ito,T.
EPI576391	HA	Sweden	2015-Mar-05	EPI_ISL_175535	A/MuteSwan/Sweden/SVA150313KU0141/SZ54 3/2015	National Veterinary Institute	National Veterinary Institute	'Zohari,Siamak';'Karin,Ullman';Olofsson,Ann-Sophie*
EPI1787898	HA	Egypt	2018-Feb-01	EPI_ISL_505411	A/Turkey/Beni Sueif/18296F/2018		Import from public-domain	Yehia,N.; Elhussieny,M.; Hagag,N.; Arafa,A.; Mohamed,W.
EPI1381395	HA	Egypt	2018-Mar-28	EPI_ISL_344539	A/Turkey/Egypt/AR550/2018	Poultry Diseases Department ,Faculty of Veterinary Medicine, Beni-Suef University	Friedrich-Loeffler-Institut	Hassan ,KE; King,J ; El-Kady, MF; Abohamra ,S; Pohlmann, A ; Harder, TC
EPI687239	HA	Germany	2014-Dec-15	EPI_ISL_198665	A/turkey/Germany/AR3390-L00939/2014		Friedrich-Loeffler-Institut	
EPI1261901	HA	Italy	2017-Dec-11	EPI_ISL_316582	A/turkey/Italy/17VIR10983-22/2017	Istituto Zooprofilattico Sperimentale Delle Venezie	Istituto Zooprofilattico Sperimentale Delle Venezie	Mulatti, P.; Fusaro, A.; Scolamacchia, F.; Zecchin, B.; Azzolini, A.; Zamperin, G.; Milani, A.; Salviato, A.; Schivo, A.; Terregino, C.; Bonfanti, L.; Monne, I.; Marangon, S.
EPI1261559	HA	Italy	2017-Sep-26	EPI_ISL_316539	A/turkey/Italy/17VIR8275-3/2017	Istituto Zooprofilattico Sperimentale Delle Venezie	Istituto Zooprofilattico Sperimentale Delle Venezie	Mulatti, P.; Fusaro, A.; Scolamacchia, F.; Zecchin, B.; Azzolini, A.; Zamperin, G.; Milani, A.; Salviato, A.; Schivo, A.; Terregino, C.; Bonfanti, L.; Monne, I.; Marangon, S.
EPI1261551	HA	Italy	2017-Sep-25	EPI_ISL_316538	A/turkey/Italy/17VIR8199-1/2017	Istituto Zooprofilattico Sperimentale Delle Venezie	Istituto Zooprofilattico Sperimentale Delle Venezie	Mulatti, P.; Fusaro, A.; Scolamacchia, F.; Zecchin, B.; Azzolini, A.; Zamperin, G.; Milani, A.; Salviato, A.; Schivo, A.; Terregino, C.; Bonfanti, L.; Monne, I.; Marangon, S.
EPI954567	HA	Italy	2017-Jan-20	EPI_ISL_255182	A/turkey/Italy/17VIR538-1/2017	Istituto Zooprofilattico Sperimentale Delle Venezie	Istituto Zooprofilattico Sperimentale Delle Venezie	Zecchin, B.; Fusaro, A.; Milani, A.; Schivo, A.; Salviato, A.; Zamperin, G.; Marciano, S.; Ormelli, S.; Terregino, C.; Monne, I.
EPI942935	HA	United Kingdc	2017-Jan-15	EPI_ISL_253036	A/turkey/England/003778/2017	Animal and Plant Health Agency (APHA)	Animal and Plant Health Agency (APHA)	Seekings, James; Ellis, Richard; Brookes, Sharon M; Reid, Scott; Essen, Stephen; Brown, Ian H
EPI858844	HA	India	2016-Oct-20	EPI_ISL_237554	A/painted stork/India/10CA03/2016	ICAR-National Institute of High Security Animal Diseases	ICAR-National Institute of High Security Animal Diseases	'Nagarajan, Shanmugasundaram; Shukla, Shweta; Kumar, Manoj; Murugkar, H. V.; Tosh, Chakradhar; Singh, Vijendra Pal
EPI823460	HA	Russian Fede	2016-May-25	EPI_ISL_230820	A/great crested grebe/Tyva/34/2016	State Research Center of Virology and Biotechnology (VECTOR)	WHO National Influenza Centre Russian Federation	Fadeev,Artem; Komissarov,Andrey; Egorova,Anna; Sintsova,Ksenia; Musaeva,Tamila; Susloparov,Ivan; Marchenko,Vasiliy; Ryzhikov,Aleksandr
EPI954743	HA	Hungary	2017-Jan-29	EPI_ISL_255204	A/Mute swan/Hungary/3542/2017	Danam.Vet.Molbiol	Danam.Vet.Molbiol	Adam, Dan
EPI624535	HA	Germany	2015-Jan-01	EPI_ISL_191765	A/scarlet_ibis/Germany/AR44-L01279/2015		Friedrich-Loeffler-Institut	
EPI1721930	HA	Bulgaria	2018-Oct-16	EPI_ISL_419362	A/chicken/Bulgaria/Haskovo/286/2018	NDRVMI (National Diagnostic and Research Veterinary Medical Institute)	University of Cambridge	

EPI1721794	HA	Bulgaria	2018-Sep-19	EPI_ISL_419345	A/chicken/Bulgaria/Plovdiv/224-1/2018	NDRVMI (National Diagnostic and Research Veterinary Medical Institute)	University of Cambridge	
EPI1185013	HA	Poland	2017-Mar-04	EPI_ISL_300712	A/chicken/Poland/263/2017		National Veterinary Research Institut Poland, PIWet-PIB	Swieton E., Smietanka K.
EPI1636681	HA	Egypt	2019-Apr-23	EPI_ISL_400043	A/Chicken/Egypt/AI20286/2019	Poultry Diseases Department ,Faculty of Veterinary Medicine, Beni-Suef University	Friedrich-Loeffler-Institut	Hassan,KE;El-Kady,MF;Harder,T.
EPI1381404	HA	Egypt	2018-May-14	EPI_ISL_344548	A/Chicken/Egypt/AR591/2018		Friedrich-Loeffler-Institut	Hassan KE, El-Khady MF, Abohamra S, Harder TC
EPI1381403	HA	Egypt	2018-Jun-25	EPI_ISL_344547	A/Chicken/Egypt/AR562/2018		Friedrich-Loeffler-Institut	Hassan KE, El-Khady MF, Abohamra S, Harder TC
EPI1381427	HA	Egypt	2018-Mar-20	EPI_ISL_344537	A/Chicken/Egypt/AR548/2018		Friedrich-Loeffler-Institut	Hassan KE, El-Khady MF, Abohamra S, Harder TC
EPI1007667	HA	Belgium	2017-Feb-01	EPI_ISL_266536	A/chicken/Belgium/807/2017		Import from public-domain	Steensels,M.; Lambrecht,B.; Vandenbussche,F.; Van Borm,S.
EPI556967	HA	Germany	2015-Jan-01	EPI_ISL_170184	A/chicken/Germany-MV/R153/2015		Friedrich-Loeffler-Institut	
EPI1019406	HA	Netherlands	2016-Dec-19	EPI_ISL_268623	A/Ch/NL-Boven Leeuwen/16016151-006-010/2016	Wageningen Bioveterinary Research	Wageningen Bioveterinary Research	Beerens, Nancy; Heutink, Rene; Harders, Frank; Verschuren-Pritz, Sylvia; Bossers, Alex; Koch, Guus; Bergervoet, Saskia
EPI1019398	HA	Netherlands	2016-Dec-12	EPI_ISL_268622	A/Ch/NL-Abbega/X16015736/2016	Wageningen Bioveterinary Research	Wageningen Bioveterinary Research	Beerens, Nancy; Heutink, Rene; Harders, Frank; Verschuren-Pritz, Sylvia; Bossers, Alex; Koch, Guus; Bergervoet, Saskia
EPI1634518	HA	Egypt	2019-Jan-23	EPI_ISL_399644	A/Duck/Egypt/SMG4/2019		Import from public-domain	Tantawy,A.S.; Sultan,H.; Arafa,A.; Hagag,N.
EPI561495	HA	Korea, Repub	2014-Jan-20	EPI_ISL_171695	A/Baikal teal/Korea/H52/2014		Import from public-domain	Jeong,J.; Kang,H.M.; Lee,E.K.; Song,B.M.; Kwon,Y.K.; Kim,H.R.; Choi,K.S.; Kim,J.Y.; Lee,H.J.; Moon,O.K.; Jeong,W.; Choi,J.; Baek,J.H.; Joo,Y.S.; Park,Y.H.; Lee,H.S.; Lee,Y.J.; Lee,Y.-J.; Kang,H.-M.; Lee,E.-K.; Song,B.-M.; Lee,K.-J.; Hong,M.-S.
EPI596295	HA	Netherlands	2014-Nov-24	EPI_ISL_181094	A/eurasian wigeon/Netherlands/2/2014		Import from public-domain	Verhagen,J.H.; Van der Jeugd,H.P.; Nolet,B.A.; Vuong,O.; Majoor,F.; De Vries,P.P.; Kharitonov,S.; Kuiken,T.; Fouchier,R.A.M.
EPI1023565	HA	Netherlands	2016-Dec-14	EPI_ISL_269596	A/Eurasian_Wigeon/Netherlands/13/2016	Erasmus Medical Center	Erasmus Medical Center	Poen,M.J.;Van Der Jeugd,H.P.;Vuong,O.;Scheuer,R.D.;Kleyheeg,E.; Bestebroer,T.M.;Kuiken,T.;Fouchier,R.A.M.
EPI552768	HA	Netherlands	2014-Nov-24	EPI_ISL_169281	A/eurasian wigeon/Netherlands/emc-2/2014	Erasmus Medical Center	Erasmus Medical Center	Fouchier, Ron A.M.; Verhagen, Josanne H.; Vuong, Oanh; Bestebroer, Theo; Van Vliet, Stefan; Van der Jeugd, Henk
EPI1019558	HA	Netherlands	2016-Dec-08	EPI_ISL_268642	A/Eur_Wig/NL-Greonterp/16015653-001/2016	Wageningen Bioveterinary Research	Wageningen Bioveterinary Research	Beerens, Nancy; Heutink, Rene; Harders, Frank; Verschuren-Pritz, Sylvia; Bossers, Alex; Koch, Guus; Bergervoet, Saskia
EPI1019526	HA	Netherlands	2016-Dec-06	EPI_ISL_268638	A/Eur_Wig/NL-Drieborg (Dollard)/16015513-001/2016	Wageningen Bioveterinary Research	Wageningen Bioveterinary Research	Beerens, Nancy; Heutink, Rene; Harders, Frank; Verschuren-Pritz, Sylvia; Bossers, Alex; Koch, Guus; Bergervoet, Saskia
EPI954877	HA	Hungary	2017-Jan-14	EPI_ISL_255220	A/Mallard/Hungary/1574a/2017	Danam.Vet.Molbiol	Danam.Vet.Molbiol	Adam, Dan
EPI867092	HA	Switzerland	2016-Nov-01	EPI_ISL_239434	A/tufted_duck/Switzerland/V246-L02001/2016	Institut für Virologie und Immunologie - Bundesamt für Lebensmittelsicherheit und Veterinärwesen	Friedrich-Loeffler-Institut	
EPI1019814	HA	Netherlands	2016-Nov-09	EPI_ISL_268674	A/T_Dk/NL-Zeewolde/16013976-001/2016	Wageningen Bioveterinary Research	Wageningen Bioveterinary Research	Beerens, Nancy; Heutink, Rene; Harders, Frank; Verschuren-Pritz, Sylvia; Bossers, Alex; Koch, Guus; Bergervoet, Saskia
EPI1023555	HA	Netherlands	2016-Dec-20	EPI_ISL_268799	A/Back-headed_Gull/Netherlands/8/2016	Erasmus Medical Center	Erasmus Medical Center	Poen,M.J.;Van Der Jeugd,H.P.;Vuong,O.;Scheuer,R.D.;Kleyheeg,E.; Bestebroer,T.M.;Begeman,L;van den Brand,J.M.A.;Kuiken,T.;Fouchier,R.A.M.
EPI1814353	HA	Russian Fede	2020-Aug-27	EPI_ISL_654835	A/chicken/Kurgan/1003/2020	State Research Center of Virology and Biotechnology (VECTOR)	State Research Center of Virology and Biotechnology (VECTOR)	Natalia,Goncharova; Ivan,Susloparov; Natalia,Kolosova; Alexey,Danilenko; Juliya,Bulanovich; Vasiliy,Marchenko; Alexander,Ryzhikov
EPI1813305	HA	Russian Fede	2020-Aug-06	EPI_ISL_644145	A/chicken/Chelyabinsk/401/2020	State Research Center of Virology and Biotechnology (VECTOR)	State Research Center of Virology and Biotechnology (VECTOR)	Natalia,Goncharova; Ivan,Susloparov; Natalia,Kolosova; Alexey,Danilenko; Juliya,Bulanovich; Vasiliy,Marchenko; Alexander,Ryzhikov
EPI1272569	HA	Russian Fede	2018-Aug-01	EPI_ISL_320958	A/chicken/Tatarstan/7/2018		State Research Center of Virology and Biotechnology (VECTOR)	Alexey,Danilenko; Vasiliy,Marchenko; Ivan,Susloparov; Natalya,Goncharova; Natalya,Kolosova; Juliya, Bulanovich; Alexander,Ryzhikov
EPI1270979	HA	Russian Fede	2018-Jun-20	EPI_ISL_320680	A/chicken/Kursk/757/2018		State Research Center of Virology and Biotechnology (VECTOR)	Alexey,Danilenko; Vasiliy,Marchenko; Ivan,Susloparov; Natalya,Goncharova; Natalya,Kolosova; Juliya, Bulanovich; Alexander,Ryzhikov
EPI1270955	HA	Russian Fede	2018-Jun-19	EPI_ISL_320677	A/chicken/Penza/607/2018		State Research Center of Virology and Biotechnology (VECTOR)	Alexey,Danilenko; Vasiliy,Marchenko; Ivan,Susloparov; Natalya,Goncharova; Natalya,Kolosova; Juliya, Bulanovich; Alexander,Ryzhikov
EPI1169201	HA	Russian Fede	2017-Nov-17	EPI_ISL_297235	A/chicken/Rostov-on-Don/1598/2017		State Research Center of Virology and Biotechnology (VECTOR)	Ivan,Susloparov; Natalya,Goncharova; Natalya,Kolosova; Vasiliy,Marchenko; Alexander,Ryzhikov
EPI1813185	HA	Russian Fede	2020-Aug-17	EPI_ISL_644130	A/goose/Omsk/01161/2020	State Research Center of Virology and Biotechnology (VECTOR)	State Research Center of Virology and Biotechnology (VECTOR)	Natalia,Goncharova; Ivan,Susloparov; Natalia,Kolosova; Alexey,Danilenko; Juliya,Bulanovich; Vasiliy,Marchenko; Alexander,Ryzhikov

EPI1813145	HA	Russian Fede	2020-Aug-17	EPI_ISL_644125	A/goose/Omsk/0111/2020	State Research Center of Virology and Biotechnology (VECTOR)	State Research Center of Virology and Biotechnology (VECTOR)	Natalia,Goncharova; Ivan,Susloparov; Natalia,Kolosova; Alexey,Danilenko; Juliya,Bulanovich; Vasiliy,Marchenko; Alexander,Ryzhikov
EPI704415	NP	Mongolia	2015-Aug-31	EPI_ISL_209131	A/duck/Mongolia/543/2015		Import from public-domain	Okamatsu,M.; Hatamachi,J.; Kida,H.; Sakoda,Y.
EPI1333764	NP	Russian Fede	2018-Oct-19	EPI_ISL_333615	A/gadwall/Chany/893/2018	State Research Center of Virology and Biotechnology (VECTOR)	State Research Center of Virology and Biotechnology (VECTOR)	Natalia,Goncharova; Ivan,Susloparov; Natalia,Kolosova; Alexey,Danilenko; Juliya,Bulanovich; Vasiliy,Marchenko; Alexander,Ryzhikov
EPI1581280	NP	Egypt	2016-Jan-28	EPI_ISL_387968	A/teal/Egypt/MB-D-487OP/2016		Import from public-domain	Direct Submission
EPI1814667	NP	Germany	2020-Sep-11	EPI_ISL_661312	A/guinea fowl/Germany-NW/AI01184/2020	Chemisches und Veterinäruntersuchungsamt Münsterland-Emscher-Lippe	Friedrich-Loeffler-Institut	
EPI1843641	NA	Italy	2020-Nov-23	EPI_ISL_956412	A/greater_white-fronted_goose/Italy/20VIR8073-4/2020	Istituto Zooprofilattico Sperimentale delle Venezie, EU/OIE/Reference Laboratory and FAO Reference Centre for AI and ND	Istituto Zooprofilattico Sperimentale Delle Venezie	Zecchin, B.; Fusaro, A.; Milani, A.; Schivo, A.; Salviato, A.; Pastori, A.; Zamperin, G.; Monne, I.; Terregino, C.
EPI740632	NA	China	2014-Jan-16	EPI_ISL_217934	A/Anser anser/Hubei/Chenhu936/2014_H6N1	State Key Laboratory of Virology and Wuhan Institute of Virology, Chinese Academy of Sciences	Wuhan Institute of Virology	
EPI1195229	NA	China	2015-Mar-09	EPI_ISL_302802	A/Bean_Goose/Hubei/chenhu_VIII71/2015	Wuhan Institute of Virology, CAS	Wuhan Institute of Virology	
EPI1195154	NA	China	2015-Mar-09	EPI_ISL_302774	A/Bean_Goose/Hubei/chenhu_VIII53/2015	Wuhan Institute of Virology, CAS	Wuhan Institute of Virology	
EPI1195139	NA	China	2015-Mar-09	EPI_ISL_302772	A/Bean_Goose/Hubei/chenhu_VIII35/2015	Wuhan Institute of Virology, CAS	Wuhan Institute of Virology	
EPI1195075	NA	China	2015-Feb-05	EPI_ISL_302762	A/Bean_Goose/Hubei/chenhu_VI74/2015	Wuhan Institute of Virology, CAS	Wuhan Institute of Virology	
EPI1194816	NA	China	2015-Feb-05	EPI_ISL_302723	A/Bean_Goose/Hubei/chenhu_VI51/2015	Wuhan Institute of Virology, CAS	Wuhan Institute of Virology	
EPI1195233	NA	China	2015-Mar-09	EPI_ISL_302808	A/Bean_Goose/Hubei/chenhu_VIII74/2015		Wuhan Institute of Virology	
EPI712786	NA	China	2015-Feb-05	EPI_ISL_212423	A/Bean Goose/Hubei/chenhu VI45/2015_H6N1	State Key Laboratory of Virology and Wuhan Institute of Virology, Chinese Academy of Sciences	Wuhan Institute of Virology	
EPI712738	NA	China	2015-Feb-05	EPI_ISL_212417	A/Bean Goose/Hubei/chenhu VI68/2015_H1N1	State Key Laboratory of Virology and Wuhan Institute of Virology, Chinese Academy of Sciences	Wuhan Institute of Virology	
EPI712730	NA	China	2015-Feb-05	EPI_ISL_212416	A/Bean Goose/Hubei/chenhu VI60/2015_H1N1	State Key Laboratory of Virology and Wuhan Institute of Virology, Chinese Academy of Sciences	Wuhan Institute of Virology	
EPI712722	NA	China	2015-Feb-05	EPI_ISL_212415	A/Bean Goose/Hubei/chenhu VI30/2015_H1N1	State Key Laboratory of Virology and Wuhan Institute of Virology, Chinese Academy of Sciences	Wuhan Institute of Virology	
EPI712707	NA	China	2015-Jan-09	EPI_ISL_212413	A/Bean Goose/Hubei/chenhu V43/2015_H1N1	State Key Laboratory of Virology and Wuhan Institute of Virology, Chinese Academy of Sciences	Wuhan Institute of Virology	
EPI712699	NA	China	2015-Jan-09	EPI_ISL_212412	A/Bean Goose/Hubei/chenhu V39/2015_H1N1	State Key Laboratory of Virology and Wuhan Institute of Virology, Chinese Academy of Sciences	Wuhan Institute of Virology	
EPI712604	NA	China	2014-Dec-02	EPI_ISL_212391	A/Bean Goose/Hubei/chenhu I66/2014_H1N1	State Key Laboratory of Virology and Wuhan Institute of Virology, Chinese Academy of Sciences	Wuhan Institute of Virology	
EPI712558	NA	China	2014-Dec-02	EPI_ISL_212381	A/Bean Goose/Hubei/chenhu I35/2014_H1N1	State Key Laboratory of Virology and Wuhan Institute of Virology, Chinese Academy of Sciences	Wuhan Institute of Virology	
EPI712541	NA	China	2014-Dec-02	EPI_ISL_212379	A/Bean Goose/Hubei/chenhu I22/2014_H1N1	State Key Laboratory of Virology and Wuhan Institute of Virology, Chinese Academy of Sciences	Wuhan Institute of Virology	
EPI1848933	NA	United Kingdc	2021-Feb-10	EPI_ISL_1123361	A/pheasant/Scotland/000348/2021	Animal and Plant Health Agency (APHA)	Animal and Plant Health Agency (APHA)	
EPI1840059	NA	United Kingdc	2020-Dec-03	EPI_ISL_766876	A/mute_swan/England/234255/2020	Animal and Plant Health Agency (APHA)	Animal and Plant Health Agency (APHA)	
EPI1014333	NA	Netherlands	2014-Dec-01	EPI_ISL_267238	A/Bewick swan/Netherlands/1/2014		Import from public-domain	
EPI1807258	NA	Netherlands	2020-Oct-16	EPI_ISL_603135	A/Eurasian Wigeon/Netherlands/5/2020	Erasmus Medical Center	Erasmus Medical Center	
EPI1807250	NA	Netherlands	2020-Oct-16	EPI_ISL_603134	A/Eurasian Wigeon/Netherlands/4/2020	Erasmus Medical Center	Erasmus Medical Center	
EPI1807242	NA	Netherlands	2020-Oct-16	EPI_ISL_603133	A/Eurasian Wigeon/Netherlands/1/2020	Erasmus Medical Center	Erasmus Medical Center	
EPI1877875	NA	Poland	2021-Apr-20	EPI_ISL_2681045	A/white_stork/Poland/MB391/2021	National Veterinary Research Institut Poland, PIWet-PIB	National Veterinary Research Institut Poland, PIWet-PIB	Edyta, Swieton; Kamila, Dziadek; Krzysztof, Smietanka
EPI1896068	NA	Nigeria	2021-Mar-01	EPI_ISL_4061485	A/chicken/Nigeria/VRD21-109_21VIR2370-425/2021		Import from public-domain	Shittu,I.; Meseko,C.; Nwosuh,C.; Muhammad,M.; Alabi,O.; Tassoni,L.; Schivo,A.; Salviato,A.; Edoardo,G.; Zecchin,B.; Fusaro,A.; Monne,I.
EPI1229985	NA	Netherlands	2014-Mar-26	EPI_ISL_309818	A/Chicken/Netherlands/14004070/2014	Wageningen Bioveterinary Research	Wageningen Bioveterinary Research	Bergervoet, Saskia; Heutink, Rene; Harders, Frank; Beerens, Nancy
EPI1229961	NA	Netherlands	2014-Feb-25	EPI_ISL_309815	A/Chicken/Netherlands/14002541/2014	Wageningen Bioveterinary Research	Wageningen Bioveterinary Research	Bergervoet, Saskia; Heutink, Rene; Harders, Frank; Beerens, Nancy

EPI580259	NA	Netherlands	2014-Mar-26	EPI_ISL_176815	A/chicken/NL-Uithuizermeeden/14004070/2014	Wageningen Bioveterinary Research	Wageningen Bioveterinary Research	Heutink,Rene;Harders,Frank;Pritz-Verschuren,Sylvia;Koch,Guus;Bouwstra,Ruth
EPI866967	NA	Japan	2015-Dec-18	EPI_ISL_239418	A/wildbird/Niigata/151213/2015	National Institute of Animal Health	National Institute of Animal Health	Mine,J
EPI855734	NA	Japan	2014-Oct-01	EPI_ISL_237157	A/duck/Kyoto/261011/2014	National Institute of Animal Health	National Institute of Animal Health	
EPI1895939	NA	Russian Fede	2013-Oct-01	EPI_ISL_4061406	A/duck/Moscow/4970-MA/2013		Import from public-domain	Lomakina,N.F.; Sadykova,G.K.; Prilipov,A.G.; Gambaryan,A.S.; Timofeeva,T.A.; Rudneva,I.A.
EPI1818110	NA	Mongolia	2018-Sep-02	EPI_ISL_697696	A/duck/Mongolia/451/2018		Import from public-domain	Sakoda,Y.; Okamatsu,M.; Matsuno,K.
EPI1818030	NA	Russian Fede	2019-Oct-15	EPI_ISL_697686	A/duck/Moscow/5743/2019		Import from public-domain	Lomakina,N.F.; Treshchalina,A.A.; Sadykova,G.K.; Prilipov,A.G.; Gambaryan,A.S.; Postnikova,Y.N.
EPI1175905	NA	Mongolia	2013-Sep-01	EPI_ISL_298543	A/duck/Mongolia/62/2013		Import from public-domain	Sakoda,Y.; Okamatsu,M.; Matsuno,K.; Jizou,M.
EPI712794	NA	China	2015-Feb-05	EPI_ISL_212424	A/Mallard/Hubei/chenhu VI88/2015_H6N1	State Key Laboratory of Virology and Wuhan Institute of Virology, Chinese Academy of Sciences	Wuhan Institute of Virology	
EPI1195090	NA	China	2015-Feb-05	EPI_ISL_302764	A/Mallard/Hubei/chenhu_VI89/2015	Wuhan Institute of Virology, CAS	Wuhan Institute of Virology	
EPI1775500	NA	Belgium	2017-Nov-19	EPI_ISL_502736	A/Anas platyrhynchos/Belgium/10402_H195386/2017		Import from public-domain	Lambrecht,B.; Steensels,M.; Fusaro,A.; Milani,A.; Pastori,A.; Schivo,A.; Salviato,A.; Zamperin,G.; Monne,I.; Terregino,C.
EPI1774410	NA	Belgium	2018-Feb-19	EPI_ISL_502606	A/Anas platyrhynchos/Belgium/1837_H101620/2018		Import from public-domain	Lambrecht,B.; Steensels,M.; Fusaro,A.; Milani,A.; Pastori,A.; Schivo,A.; Salviato,A.; Zamperin,G.; Monne,I.; Terregino,C.
EPI1778777	NA	Belgium	2018-Nov-26	EPI_ISL_502605	A/Anas platyrhynchos/Belgium/11958/2018		Import from public-domain	Lambrecht,B.; Steensels,M.; Fusaro,A.; Milani,A.; Pastori,A.; Schivo,A.; Salviato,A.; Zamperin,G.; Monne,I.; Terregino,C.
EPI1774402	NA	Belgium	2018-Nov-26	EPI_ISL_502605	A/Anas platyrhynchos/Belgium/11958/2018		Import from public-domain	Lambrecht,B.; Steensels,M.; Fusaro,A.; Milani,A.; Pastori,A.; Schivo,A.; Salviato,A.; Zamperin,G.; Monne,I.; Terregino,C.
EPI1634325	NA	Belgium	2016-Oct-07	EPI_ISL_399465	A/Anas platyrhynchos/Belgium/9594H191828/2016		Import from public-domain	Steensels,M.; Lambrecht,B.
EPI1774594	NA	Belgium	2016-Oct-07	EPI_ISL_399465	A/Anas platyrhynchos/Belgium/9594H191828/2016		Import from public-domain	Steensels,M.; Lambrecht,B.
EPI1634324	NA	Belgium	2016-Oct-07	EPI_ISL_399464	A/Anas platyrhynchos/Belgium/9594H191810/2016		Import from public-domain	Steensels,M.; Lambrecht,B.
EPI1774586	NA	Belgium	2016-Oct-07	EPI_ISL_399464	A/Anas platyrhynchos/Belgium/9594H191810/2016		Import from public-domain	Steensels,M.; Lambrecht,B.
EPI1307025	NA	Georgia	2015-Oct-10	EPI_ISL_328971	A/mallard duck/Georgia/7/2015		Import from public-domain	Poen,M.J.; Verhagen,J.H.; Vuong,O.; Scheuer,R.D.; Pas,S.D.; Fouchier,R.A.M.
EPI961548	NA	Sweden	2014-Dec-15	EPI_ISL_256316	A/Mallard/Sweden/816/2014		Import from public-domain	Wille,M.; Lindqvist,K.; Muradrasoli,S.; Olsen,B.; Jarhult,J.; Jarhult,J.D.
EPI617857	NA	Georgia	2012-Dec-19	EPI_ISL_189708	A/mallard/Republic of Georgia/4/2012		Import from public-domain	Wentworth,D.E.; Halpin,R.A.; Lin,X.; Simenauer,A.; Akopov,A.; Mohan,M.; Fedorova,N.; Tsitrin,T.; Puri,V.; Stockwell,T.; Amedeo,P.; Bishop,B.; Gupta,N.; Hoover,J.; Katzel,D.; Schobel,S.; Shrivastava,S.; Thovarai,V.; Bao,Y.; Sanders,R.; Zhdanov,S.; Kiryutin,B.; Lipman,D.J.; Tatusova,T.; Fouchier,R.A.M.
EPI1140144	NA	Netherlands	2012-Mar-16	EPI_ISL_291187	A/Mallard/Netherlands/12005909/2012	Wageningen Bioveterinary Research	Wageningen Bioveterinary Research	Bergervoet, Saskia; Heutink, Rene; Harders, Frank; Beerens, Nancy
EPI1815144	NA	Italy	2020-Nov-21	EPI_ISL_683592	A/Eurasian_wigeon/Italy/20VIR7301-206/2020	Istituto Zooprofilattico Sperimentale delle Venezie, EU/OIE/Reference Laboratory and FAO Reference Centre for AI and ND	Istituto Zooprofilattico Sperimentale Delle Venezie	Zecchin, B.; Fusaro, A.; Pastori, A.; Milani, A.; Salviato, A.; Schivo, A.; Monne, I.; Terregino, C.
EPI1841795	NA	Netherlands	2019-Jan-23	EPI_ISL_819124	A/mallard/Netherlands/19001282-001/2019	Wageningen Bioveterinary Research	Wageningen Bioveterinary Research	Beerens, Nancy; Harders, Frank; Pritz-Verschuren, Sylvia; Roose, Marit; Germeraad, Evelien; Engelsma, Marc; Bossers, Alex; Heutink, Rene
EPI1841787	NA	Netherlands	2018-Oct-11	EPI_ISL_815400	A/mallard/Netherlands/18015513-001/2018	Wageningen Bioveterinary Research	Wageningen Bioveterinary Research	Beerens, Nancy; Harders, Frank; Pritz-Verschuren, Sylvia; Roose, Marit; Germeraad, Evelien; Engelsma, Marc; Bossers, Alex; Heutink, Rene
EPI579929	NA	Netherlands	2014-Feb-25	EPI_ISL_176754	A/Chicken/NL-Swifterband/14002541/2014	Wageningen Bioveterinary Research	Wageningen Bioveterinary Research	Heutink,Rene; Harders,Frank; Verschuren-Pritz,Sylvia; Koch,Guus; Bouwstra,Ruth
EPI1883978	NA	Hungary	2021-Apr-13	EPI_ISL_3135897	A/Turkey/Hungary/16603/2021 (H5N1)	National Food Chain Safety Office Veterinary Diagnostic Directorate Laboratory for Molecular Biology	National Food Chain Safety Office, Hungary	Katalin,Szentpáli-Gavallér;Ádám,Bálint;Krisztina,Ursu;Péter,Malik
EPI1847782	NA	Denmark	2021-Jan-01	EPI_ISL_1063993	A/common kestrel/Denmark/16023-01/2021-01-01(H5N3)	Statens Serumm Institute	Statens Serum Institute	Charlotte Hjulsager, Jesper Schak Krog
EPI1148065	NA	Korea, Repub	2017-Feb-08	EPI_ISL_292822	A/white-fronted goose/Korea/H952/2017	Animal and Plant Quarantine Agency (O-2144)	Animal and Plant Quarantine Agency (S-2145)	
EPI1148057	NA	Korea, Repub	2017-Feb-08	EPI_ISL_292821	A/white-fronted goose/Korea/H909-1/2017	Animal and Plant Quarantine Agency (O-2144)	Animal and Plant Quarantine Agency (S-2145)	
EPI1208377	NA	China	2015-Mar-09	EPI_ISL_302809	A/Bean_Goose/Hubei/chenhu_IX25/2015	Wuhan Institute of Virology, CAS	Wuhan Institute of Virology	
EPI1195144	NA	China	2015-Mar-09	EPI_ISL_302773	A/Bean_Goose/Hubei/chenhu_VIII40/2015	Wuhan Institute of Virology, CAS	Wuhan Institute of Virology	

EPI1195109	NA	China	2015-Feb-05	EPI_ISL_302766	A/Bean_Goose/Hubei/chenhu_VI54/2015	Wuhan Institute of Virology, CAS	Wuhan Institute of Virology	
EPI1195098	NA	China	2015-Feb-05	EPI_ISL_302765	A/Bean_Goose/Hubei/chenhu_VI14/2015	Wuhan Institute of Virology, CAS	Wuhan Institute of Virology	
EPI1195087	NA	China	2015-Feb-05	EPI_ISL_302763	A/Bean_Goose/Hubei/chenhu_VI77/2015	Wuhan Institute of Virology, CAS	Wuhan Institute of Virology	
EPI1195070	NA	China	2015-Feb-05	EPI_ISL_302761	A/Bean_Goose/Hubei/chenhu_VI70/2015	Wuhan Institute of Virology, CAS	Wuhan Institute of Virology	
EPI1195049	NA	China	2015-Feb-05	EPI_ISL_302730	A/Bean_Goose/Hubei/chenhu_VI62/2015	Wuhan Institute of Virology, CAS	Wuhan Institute of Virology	
EPI1194836	NA	China	2015-Feb-05	EPI_ISL_302726	A/Bean_Goose/Hubei/chenhu_VI59/2015	Wuhan Institute of Virology, CAS	Wuhan Institute of Virology	
EPI1194830	NA	China	2015-Feb-05	EPI_ISL_302724	A/Bean_Goose/Hubei/chenhu_VI55/2015	Wuhan Institute of Virology, CAS	Wuhan Institute of Virology	
EPI1194801	NA	China	2015-Feb-05	EPI_ISL_302721	A/Bean_Goose/Hubei/chenhu_VI47/2015	Wuhan Institute of Virology, CAS	Wuhan Institute of Virology	
EPI1194784	NA	China	2015-Feb-05	EPI_ISL_302717	A/Bean_Goose/Hubei/chenhu_VI39/2015	Wuhan Institute of Virology, CAS	Wuhan Institute of Virology	
EPI1194774	NA	China	2015-Feb-05	EPI_ISL_302713	A/Bean_Goose/Hubei/chenhu_VI37/2015	Wuhan Institute of Virology, CAS	Wuhan Institute of Virology	
EPI1194765	NA	China	2015-Feb-05	EPI_ISL_302712	A/Bean_Goose/Hubei/chenhu_VI36/2015	Wuhan Institute of Virology, CAS	Wuhan Institute of Virology	
EPI1194758	NA	China	2015-Feb-05	EPI_ISL_302711	A/Bean_Goose/Hubei/chenhu_VI33/2015	Wuhan Institute of Virology, CAS	Wuhan Institute of Virology	
EPI1193363	NA	China	2015-Feb-05	EPI_ISL_294954	A/Bean_Goose/Hubei/chenhu_IX1/2015	Wuhan Institute of Virology, CAS	Wuhan Institute of Virology	
EPI895348	NA	China	2014-Dec-22	EPI_ISL_244555	A/Anser fabalis/China/Anhui/L256/2014		Import from public-domain	Ye,G.; Liang,C.H.; Ping,H.Y.; Hua,D.G.; Cheng,Y.Q.
EPI895340	NA	China	2014-Dec-22	EPI_ISL_244553	A/Anser fabalis/China/Anhui/L221/2014		Import from public-domain	Ye,G.; Liang,C.H.; Ping,H.Y.; Hua,D.G.; Cheng,Y.Q.
EPI1194808	NA	China	2015-Feb-05	EPI_ISL_302722	A/Great_Cormorant/Hubei/chenhu_VI48/2015	Wuhan Institute of Virology, CAS	Wuhan Institute of Virology	
EPI1194746	NA	China	2015-Feb-05	EPI_ISL_302709	A/Great_Cormorant/Hubei/chenhu_VI12/2015	Wuhan Institute of Virology, CAS	Wuhan Institute of Virology	
EPI712656	NA	China	2014-Dec-23	EPI_ISL_212406	A/Wild bird/Hubei/Wanghu III12-13/2014_H6N1	State Key Laboratory of Virology and Wuhan Institute of Virology, Chinese Academy of Sciences	Wuhan Institute of Virology	
EPI1006620	NA	China	2014-Dec-22	EPI_ISL_266409	A/Anseriformes/Anhui/S107/2014		Import from public-domain	Ye,G.; Liang,C.H.; Ping,H.Y.; Hua,D.G.; Cheng,Y.Q.
EPI1006607	NA	China	2014-Dec-24	EPI_ISL_266407	A/Anseriformes/Anhui/S3/2014		Import from public-domain	Ye,G.; Liang,C.H.; Ping,H.Y.; Hua,D.G.; Cheng,Y.Q.
EPI682990	NA	China	2014-Dec-01	EPI_ISL_205136	A/wild bird/Wuhan/WHHN58/2014		Import from public-domain	Chen,L.-J.; Lin,X.-D.; Guo,W.-P.; Tian,J.-H.; Zhang,Y.-Z.
EPI682989	NA	China	2014-Dec-01	EPI_ISL_205135	A/wild bird/Wuhan/WHHN16/2014		Import from public-domain	Chen,L.-J.; Lin,X.-D.; Guo,W.-P.; Tian,J.-H.; Zhang,Y.-Z.
EPI1510474	NA	Belgium	2019-Jan-18	EPI_ISL_368620	A/Gallus gallus/Belgium/609/2019		Import from public-domain	Steensels,M.; Roupie,V.; Rauw,F.; Lambrecht,B.
EPI660910	NA	China	2015-Jan-14	EPI_ISL_199320	A/duck/Jiangxi/01.14 NCJD039-P/2015(H1N1)		Institute of Microbiology, Chinese Academy of Sciences	
EPI660878	NA	China	2015-Feb-05	EPI_ISL_199316	A/duck/Jiangxi/02.05 YGYXG037-P/2015(H1N1)		Institute of Microbiology, Chinese Academy of Sciences	
EPI873495	NA	Japan	2016-Dec-14	EPI_ISL_240623	A/duck/Ibaraki/109/2016	National Institute of Animal Health	National Institute of Animal Health	Uchida,Y.
EPI873334	NA	Japan	2016-Dec-14	EPI_ISL_240603	A/duck/Ibaraki/108/2016	National Institute of Animal Health	National Institute of Animal Health	Uchida,Y.
EPI855906	NA	Japan	2015-Oct-01	EPI_ISL_237172	A/duck/Kyoto/261007/2015	National Institute of Animal Health	National Institute of Animal Health	
EPI855742	NA	Japan	2016-Mar-01	EPI_ISL_237158	A/duck/Shiga/250302/2016	National Institute of Animal Health	National Institute of Animal Health	
EPI855710	NA	Japan	2013-Dec-01	EPI_ISL_237154	A/duck/Kagoshima/461210/2013	National Institute of Animal Health	National Institute of Animal Health	
EPI855574	NA	Japan	2013-Nov-01	EPI_ISL_237137	A/duck/Saga/411117/2013	National Institute of Animal Health	National Institute of Animal Health	
EPI1576691	NA	Vietnam	2015-Oct-25	EPI_ISL_387333	A/duck/Viet Nam/HN-2515/2015		Import from public-domain	Tan,G.; Pickett,B.; Fedorova,N.; Amedeo,P.; Isom,R.; Hu,L.; Christensen,J.; Miller,J.; Durbin,A.; Williams,T.; Arumemi,F.; Kawaoka,Y.; Zhong,G.; Neumann,G.
EPI703429	NA	China	2014-Feb-01	EPI_ISL_208807	A/duck/Fujian/JF47/2014		Import from public-domain	Liu,X.; Liu,S.; Wang,S.; Zhuang,Q.; Jiang,W.; Hou,G.; Yu,J.; Wang,K.; Li,J.
EPI966716	NA	United States	2015-Sep-25	EPI_ISL_257273	A/northern pintail/Alaska/UGAI15-7300/2015		Import from public-domain	Reeves,A.B.; Ramey,A.M.; Stallknecht,D.E.
EPI1307084	NA	Netherlands	2015-Sep-29	EPI_ISL_328977	A/common teal/Netherlands/1/2015		Import from public-domain	Poen,M.J.; Verhagen,J.H.; Vuong,O.; Scheuer,R.D.; Pas,S.D.; Fouchier,R.A.M.
EPI1014613	NA	Georgia	2011-Jan-22	EPI_ISL_267279	A/mallard duck/Georgia/11/2011		Import from public-domain	Lewis,N.S.; Machablishvili,A.; Chkhaidze,M.; Vuong,O.; Scheuer,R.D.; Poen,M.J.; Fouchier,R.A.M.
EPI1011304	NA	Netherlands	2014-Aug-27	EPI_ISL_267256	A/mallard duck/Netherlands/18/2014		Import from public-domain	
EPI617892	NA	Georgia	2012-Jan-10	EPI_ISL_189702	A/mallard/Republic of Georgia/1/2012		Import from public-domain	Wentworth,D.E.; Halpin,R.A.; Lin,X.; Simenauer,A.; Akopov,A.; Mohan,M.; Fedorova,N.; Tsitrin,T.; Puri,V.; Stockwell,T.; Amedeo,P.; Bishop,B.; Gupta,N.; Hoover,J.; Katzel,D.; Schobel,S.; Shrivastava,S.; Thovarai,V.; Bao,Y.; Sanders,R.; Zhdanov,S.; Kiryutin,B.; Lipman,D.J.; Tatusova,T.; Fouchier,R.A.M.
EPI1841811	NA	Netherlands	2019-Aug-13	EPI_ISL_822837	A/green sandpiper/Netherlands/19011490-013/2019	Wageningen Bioveterinary Research	Wageningen Bioveterinary Research	Beerens, Nancy; Harders, Frank; Pritz-Verschuren, Sylvia; Roose, Marit; Germeraad, Evelien; Engelsma, Marc; Bossers, Alex; Heutink, Rene
EPI1860038	NA	Ireland	2021-Jan-01	EPI_ISL_2234808	A/knot_wader/Ireland/473_21VIR2956-2/2021	Istituto Zooprofilattico Sperimentale delle Venezie, EU/OIE/Reference Laboratory and FAO Reference Centre for AI and ND	Istituto Zooprofilattico Sperimentale Delle Venezie	Garza Cuartero, L.; Flynn, O.; Connaghan, E.; Byrne, C.; O?Neill, R.; Zecchin, B.; Fusaro, A.; Pastori, A.; Salviato, A.; Schivo, A.; Monne, I.; Terregino, C.
EPI1860030	NA	Ireland	2021-Jan-01	EPI_ISL_2234807	A/knot_wader/Ireland/472_21VIR2956-1/2021	Istituto Zooprofilattico Sperimentale delle Venezie, EU/OIE/Reference Laboratory and FAO Reference Centre for AI and ND	Istituto Zooprofilattico Sperimentale Delle Venezie	Garza Cuartero, L.; Flynn, O.; Connaghan, E.; Byrne, C.; O?Neill, R.; Zecchin, B.; Fusaro, A.; Pastori, A.; Salviato, A.; Schivo, A.; Monne, I.; Terregino, C.
EPI1619615	NA	China	2017-Jan-19	EPI_ISL_397334	A/Anser Fabalis/Jiangsu/J746/2017	College of Wildlife and Protected Areas, Northeast Forestry University	Northeast Forestry University	

EPI1307705	NA	Netherlands	2014-Dec-26	EPI_ISL_329052	A/black-headed gull/Netherlands/21/2014		Import from public-domain	Poen,M.J.; Verhagen,J.H.; Vuong,O.; Scheuer,R.D.; Pas,S.D.; Fouchier,R.A.M.
EPI1153484	NA	Vietnam	2015-Nov-07	EPI_ISL_293988	A/chicken/Vietnam/HU4-26/2015		Import from public-domain	Sakoda,Y.; Okamatsu,M.; Matsuno,K.; Jizou,M.
EPI1567221	NA	Korea, Repub	2014-Dec-27	EPI_ISL_381806	A/wild waterfowl/Korea/M129/2014		Import from public-domain	Kang,M.; Jang,H.-K.
EPI891094	NA	Netherlands	2010-Nov-13	EPI_ISL_243385	A/greater white-fronted goose/Netherlands/5/2010		Import from public-domain	Poen,M.J.; Verhagen,J.H.; Vuong,O.; Scheuer,R.D.; Pas,S.D.; Fouchier,R.A.M.
EPI617949	NA	Georgia	2011-May-02	EPI_ISL_189698	A/Mediterranean gull/Republic of Georgia/1/2011		Import from public-domain	Wentworth,D.E.; Halpin,R.A.; Lin,X.; Simenauer,A.; Akopov,A.; Mohan,M.; Fedorova,N.; Tsitrin,T.; Puri,V.; Stockwell,T.; Amedeo,P.; Bishop,B.; Gupta,N.; Hoover,J.; Katzel,D.; Schobel,S.; Shrivastava,S.; Thovarai,V.; Bao,Y.; Sanders,R.; Zhdanov,S.; Kiryutin,B.; Lipman,D.J.; Tatusova,T.; Fouchier,R.A.M.
EPI617787	NA	Georgia	2012-Sep-13	EPI_ISL_189724	A/yellow-legged gull/Republic of Georgia/1/2012		Import from public-domain	Wentworth,D.E.; Halpin,R.A.; Lin,X.; Simenauer,A.; Akopov,A.; Mohan,M.; Fedorova,N.; Tsitrin,T.; Puri,V.; Stockwell,T.; Amedeo,P.; Bishop,B.; Gupta,N.; Hoover,J.; Katzel,D.; Schobel,S.; Shrivastava,S.; Thovarai,V.; Bao,Y.; Sanders,R.; Zhdanov,S.; Kiryutin,B.; Lipman,D.J.; Tatusova,T.; Fouchier,R.A.M.
EPI617788	NA	Georgia	2012-Sep-13	EPI_ISL_189724	A/yellow-legged gull/Republic of Georgia/1/2012		Import from public-domain	Wentworth,D.E.; Halpin,R.A.; Lin,X.; Simenauer,A.; Akopov,A.; Mohan,M.; Fedorova,N.; Tsitrin,T.; Puri,V.; Stockwell,T.; Amedeo,P.; Bishop,B.; Gupta,N.; Hoover,J.; Katzel,D.; Schobel,S.; Shrivastava,S.; Thovarai,V.; Bao,Y.; Sanders,R.; Zhdanov,S.; Kiryutin,B.; Lipman,D.J.; Tatusova,T.; Fouchier,R.A.M.
EPI617916	NA	Georgia	2011-May-01	EPI_ISL_189725	A/black-headed gull/Republic of Georgia/5/2011		Import from public-domain	Wentworth,D.E.; Halpin,R.A.; Lin,X.; Simenauer,A.; Akopov,A.; Mohan,M.; Fedorova,N.; Tsitrin,T.; Puri,V.; Stockwell,T.; Amedeo,P.; Bishop,B.; Gupta,N.; Hoover,J.; Katzel,D.; Schobel,S.; Shrivastava,S.; Thovarai,V.; Bao,Y.; Sanders,R.; Zhdanov,S.; Kiryutin,B.; Lipman,D.J.; Tatusova,T.; Fouchier,R.A.M.
EPI617921	NA	Georgia	2011-May-01	EPI_ISL_189696	A/black-headed gull/Republic of Georgia/8/2011		Import from public-domain	Wentworth,D.E.; Halpin,R.A.; Lin,X.; Simenauer,A.; Akopov,A.; Mohan,M.; Fedorova,N.; Tsitrin,T.; Puri,V.; Stockwell,T.; Amedeo,P.; Bishop,B.; Gupta,N.; Hoover,J.; Katzel,D.; Schobel,S.; Shrivastava,S.; Thovarai,V.; Bao,Y.; Sanders,R.; Zhdanov,S.; Kiryutin,B.; Lipman,D.J.; Tatusova,T.; Fouchier,R.A.M.
EPI1841827	NA	Netherlands	2018-Dec-28	EPI_ISL_825158	A/swan/Netherlands/18019125-002/2018	Wageningen Bioveterinary Research	Wageningen Bioveterinary Research	Beerens, Nancy; Harders, Frank; Pritz-Verschuren, Sylvia; Roose, Marit; Germeraad, Evelien; Engelsma, Marc; Bossers, Alex; Heutink, Rene
EPI1850162	NA	Germany	2020-Dec-16	EPI_ISL_1205489	A/red knot/Germany-SH/AI03424/2020	Landeslabor Schleswig-Holstein	Friedrich-Loeffler-Institut	
EPI1850154	NA	Germany	2020-Dec-14	EPI_ISL_1205487	A/red knot/Germany-SH/AI03421/2020	Landeslabor Schleswig-Holstein	Friedrich-Loeffler-Institut	
EPI1850146	NA	Germany	2020-Dec-14	EPI_ISL_1205485	A/red knot/Germany-SH/AI03419/2020	Landeslabor Schleswig-Holstein	Friedrich-Loeffler-Institut	
EPI1230078	NA	Netherlands	2016-Aug-30	EPI_ISL_309831	A/Chicken/Netherlands/16010778-021-025/2016	Wageningen Bioveterinary Research	Wageningen Bioveterinary Research	Bergervoet, Saskia; Heutink, Rene; Harders, Frank; Beerens, Nancy
EPI1229953	NA	Netherlands	2013-Dec-10	EPI_ISL_309814	A/Chicken/Netherlands/13016263-031-035/2013	Wageningen Bioveterinary Research	Wageningen Bioveterinary Research	Bergervoet, Saskia; Heutink, Rene; Harders, Frank; Beerens, Nancy
EPI1229945	NA	Netherlands	2013-Nov-29	EPI_ISL_309813	A/Chicken/Netherlands/13015884/2013	Wageningen Bioveterinary Research	Wageningen Bioveterinary Research	Bergervoet, Saskia; Heutink, Rene; Harders, Frank; Beerens, Nancy
EPI580218	NA	Netherlands	2013-Nov-29	EPI_ISL_176810	A/Chicken/NL-Sint Annen/13015884/2013	Wageningen Bioveterinary Research	Wageningen Bioveterinary Research	Heutink,Rene;Harders,Frank;Pritz-Verschuren,Sylvia;Koch,Guus;Bouwstra,Ruth
EPI580201	NA	Netherlands	2013-Dec-09	EPI_ISL_176809	A/chicken/NL_Scheemda/13016263/2013	Wageningen Bioveterinary Research	Wageningen Bioveterinary Research	Heutink,Rene;Harders,Frank;Verschuren-Pritz,Sylvia;Koch,Guus;Bouwstra,Ruth
EPI1156488	NA	France	2015-Dec-09	EPI_ISL_294770	A/duck/France/150234/2015	Anses (Ploufragan-Plouzané)	ANSES Agence Nationale De Securite Sanitaire De L'alimentation	
EPI1841835	NA	Netherlands	2018-Dec-24	EPI_ISL_825173	A/duck/Netherlands/18018989-011015/2018	Wageningen Bioveterinary Research	Wageningen Bioveterinary Research	Beerens, Nancy; Harders, Frank; Pritz-Verschuren, Sylvia; Roose, Marit; Germeraad, Evelien; Engelsma, Marc; Bossers, Alex; Heutink, Rene
EPI377996	NA	Netherlands	2008-Jan-01	EPI_ISL_122098	A/common teal/Netherlands/2/2008		Import from public-domain	Wentworth,D.E.; Dugan,V.; Halpin,R.; Lin,X.; Wester,E.; Ghedin,E.; Fedorova,N.; Tsitrin,T.; Stockwell,T.; Amedeo,P.; Bishop,B.; Edworthy,P.; Gupta,N.; Katzel,D.; Li,K.; Schobel,S.; Shrivastava,S.; Thovarai,V.; Wang,S.; Webster,R.; Webby,R.; Krauss,S.; Bao,Y.; Sanders,R.; Dernovoy,D.; Kiryutin,B.; Lipman,D.J.; Tatusova,T.

EPI1774578	NA	Belgium	2016-Oct-04	EPI_ISL_502617	A/Anas platyrhynchos/Belgium/9473/2016	Import from public-domain	Lambrecht,B.; Steensels,M.; Fusaro,A.; Milani,A.; Pastori,A.; Schivo,A.; Salviato,A.; Zamperin,G.; Monne,I.; Terregino,C.
EPI1774562	NA	Belgium	2016-Sep-25	EPI_ISL_502616	A/Anas platyrhynchos/Belgium/9074_H191808/2016	Import from public-domain	Lambrecht,B.; Steensels,M.; Fusaro,A.; Milani,A.; Pastori,A.; Schivo,A.; Salviato,A.; Zamperin,G.; Monne,I.; Terregino,C.
EPI1774546	NA	Belgium	2016-Sep-20	EPI_ISL_502615	A/Anas platyrhynchos/Belgium/8862_H170636/2016	Import from public-domain	Lambrecht,B.; Steensels,M.; Fusaro,A.; Milani,A.; Pastori,A.; Schivo,A.; Salviato,A.; Zamperin,G.; Monne,I.; Terregino,C.
EPI1774386	NA	Belgium	2016-Nov-04	EPI_ISL_502603	A/Anas platyrhynchos/Belgium/11442_39/2016	Import from public-domain	Lambrecht,B.; Steensels,M.; Fusaro,A.; Milani,A.; Pastori,A.; Schivo,A.; Salviato,A.; Zamperin,G.; Monne,I.; Terregino,C.
EPI1778941	NA	Belgium	2017-Aug-29	EPI_ISL_502596	A/Anas platyrhynchos/Belgium/10749_47/2017	Import from public-domain	Lambrecht,B.; Steensels,M.; Fusaro,A.; Milani,A.; Pastori,A.; Schivo,A.; Salviato,A.; Zamperin,G.; Monne,I.; Terregino,C.
EPI1774306	NA	Belgium	2017-Aug-29	EPI_ISL_502596	A/Anas platyrhynchos/Belgium/10749_47/2017	Import from public-domain	Lambrecht,B.; Steensels,M.; Fusaro,A.; Milani,A.; Pastori,A.; Schivo,A.; Salviato,A.; Zamperin,G.; Monne,I.; Terregino,C.
EPI1778769	NA	Belgium	2018-Oct-09	EPI_ISL_502595	A/Anas platyrhynchos/Belgium/10409/2018	Import from public-domain	Lambrecht,B.; Steensels,M.; Fusaro,A.; Milani,A.; Pastori,A.; Schivo,A.; Salviato,A.; Zamperin,G.; Monne,I.; Terregino,C.
EPI1774298	NA	Belgium	2018-Oct-09	EPI_ISL_502595	A/Anas platyrhynchos/Belgium/10409/2018	Import from public-domain	Lambrecht,B.; Steensels,M.; Fusaro,A.; Milani,A.; Pastori,A.; Schivo,A.; Salviato,A.; Zamperin,G.; Monne,I.; Terregino,C.
EPI1634280	NA	Belgium	2016-Nov-29	EPI_ISL_399485	A/Anas platyrhynchos/Belgium/11127/2016	Import from public-domain	Steensels,M.; Lambrecht,B.
EPI1774362	NA	Belgium	2016-Nov-29	EPI_ISL_399485	A/Anas platyrhynchos/Belgium/11127/2016	Import from public-domain	Steensels,M.; Lambrecht,B.
EPI1307270	NA	Netherlands	2010-Jul-20	EPI_ISL_329001	A/mallard duck/Netherlands/5/2010	Import from public-domain	Poen,M.J.; Verhagen,J.H.; Vuong,O.; Scheuer,R.D.; Pas,S.D.; Fouchier,R.A.M.
EPI1306959	NA	Netherlands	2015-Jun-05	EPI_ISL_328964	A/mallard duck/Netherlands/11/2015	Import from public-domain	Poen,M.J.; Verhagen,J.H.; Vuong,O.; Scheuer,R.D.; Pas,S.D.; Fouchier,R.A.M.
EPI1306973	NA	Netherlands	2015-Jun-05	EPI_ISL_328964	A/mallard duck/Netherlands/11/2015	Import from public-domain	Poen,M.J.; Verhagen,J.H.; Vuong,O.; Scheuer,R.D.; Pas,S.D.; Fouchier,R.A.M.
EPI1014627	NA	Netherlands	2009-Sep-09	EPI_ISL_267392	A/mallard duck/Netherlands/13/2009	Import from public-domain	
EPI1014190	NA	Netherlands	2014-Sep-30	EPI_ISL_267389	A/mallard duck/Netherlands/24/2014	Import from public-domain	
EPI1014539	NA	Netherlands	2011-Oct-03	EPI_ISL_267387	A/mallard duck/Netherlands/24/2011	Import from public-domain	
EPI1013975	NA	Georgia	2012-Jan-30	EPI_ISL_267350	A/mallard duck/Georgia/3/2012	Import from public-domain	Lewis,N.S.; Machablishvili,A.; Chkhaidze,M.; Vuong,O.; Scheuer,R.D.; Poen,M.J.; Fouchier,R.A.M.
EPI1013793	NA	Netherlands	2011-Oct-05	EPI_ISL_267333	A/mallard duck/Netherlands/25/2011	Import from public-domain	
EPI1011255	NA	Netherlands	2013-Apr-09	EPI_ISL_267268	A/mallard duck/Netherlands/23/2013	Import from public-domain	
EPI1011213	NA	Netherlands	2010-Jul-08	EPI_ISL_267189	A/mallard duck/Netherlands/4/2010	Import from public-domain	
EPI1013595	NA	Netherlands	2013-Jan-07	EPI_ISL_267171	A/mallard duck/Netherlands/22/2013	Import from public-domain	
EPI891158	NA	Netherlands	2006-Sep-11	EPI_ISL_243585	A/mallard duck/Netherlands/36/2006	Import from public-domain	Poen,M.J.; Verhagen,J.H.; Vuong,O.; Scheuer,R.D.; Pas,S.D.; Fouchier,R.A.M.
EPI1537031	NA	Netherlands	2014-Sep-20	EPI_ISL_376201	A/mallard/Netherlands/32/2014	Import from public-domain	Poen,M.J.; Verhagen,J.H.; Vuong,O.; Scheuer,R.D.; Pas,S.D.; Fouchier,R.A.M.
EPI1537004	NA	Netherlands	2014-Sep-20	EPI_ISL_376201	A/mallard/Netherlands/32/2014	Import from public-domain	Poen,M.J.; Verhagen,J.H.; Vuong,O.; Scheuer,R.D.; Pas,S.D.; Fouchier,R.A.M.
EPI1530563	NA	Netherlands	2014-Sep-20	EPI_ISL_373086	A/Mallard/Netherlands/31/2014	Import from public-domain	Poen,M.J.; Verhagen,J.H.; Vuong,O.; Scheuer,R.D.; Pas,S.D.; Fouchier,R.A.M.
EPI617994	NA	Georgia	2010-Feb-03	EPI_ISL_189721	A/mallard/Republic of Georgia/3/2010	Import from public-domain	Wentworth,D.E.; Halpin,R.A.; Lin,X.; Simenauer,A.; Akopov,A.; Mohan,M.; Fedorova,N.; Tsitrin,T.; Puri,V.; Stockwell,T.; Amedeo,P.; Bishop,B.; Gupta,N.; Hoover,J.; Katzel,D.; Schobel,S.; Shrivastava,S.; Thovarai,V.; Bao,Y.; Sanders,R.; Zhdanov,S.; Kiryutin,B.; Lipman,D.J.; Tatusova,T.; Fouchier,R.A.M.
EPI513365	NA	Sweden	2009-Nov-14	EPI_ISL_158398	A/mallard/Sweden/101900/2009	Import from public-domain	Wentworth,D.E.; Halpin,R.A.; Lin,X.; Bera,J.; Ransier,A.; Fedorova,N.; Tsitrin,T.; McLellan,M.; Stockwell,T.; Amedeo,P.; Appalla,L.; Bishop,B.; Edworthy,P.; Gupta,N.; Hoover,J.; Katzel,D.; Li,K.; Schobel,S.; Shrivastava,S.; Thovarai,V.; Wang,S.; Fouchier,R.; Osterhaus,A.; Olsen,B.; Wille,M.; Latorre-Margalef,N.; Tolf,C.; Bao,Y.; Sanders,R.; Zhdanov,S.; Kiryutin,B.; Lipman,D.J.; Tatusova,T.; Waldenstrom,J.
EPI378020	NA	Netherlands	2010-Jan-01	EPI_ISL_122101	A/mallard/Netherlands/1/2010	Import from public-domain	Wentworth,D.E.; Dugan,V.; Halpin,R.; Lin,X.; Wester,E.; Ghedin,E.; Fedorova,N.; Tsitrin,T.; Stockwell,T.; Amedeo,P.; Bishop,B.; Edworthy,P.; Gupta,N.; Katzel,D.; Li,K.; Schobel,S.; Shrivastava,S.; Thovarai,V.; Wang,S.; Webster,R.; Webby,R.; Krauss,S.; Bao,Y.; Sanders,R.; Dernovoy,D.; Kiryutin,B.; Lipman,D.J.; Tatusova,T.

EPI378012	NA	Netherlands	2008-Jan-01	EPI_ISL_122100	A/mallard/Netherlands/12/2008		Import from public-domain	Wentworth,D.E.; Dugan,V.; Halpin,R.; Lin,X.; Wester,E.; Ghedin,E.; Fedorova,N.; Tsitrin,T.; Stockwell,T.; Amedeo,P.; Bishop,B.; Edworthy,P.; Gupta,N.; Katzel,D.; Li,K.; Schobel,S.; Shrivastava,S.; Thovarai,V.; Wang,S.; Webster,R.; Webby,R.; Krauss,S.; Bao,Y.; Sanders,R.; Dernovoy,D.; Kiryutin,B.; Lipman,D.J.; Tatusova,T.
EPI378004	NA	Netherlands	2010-Jan-01	EPI_ISL_122099	A/mallard/Netherlands/29/2010		Import from public-domain	Wentworth,D.E.; Dugan,V.; Halpin,R.; Lin,X.; Wester,E.; Ghedin,E.; Fedorova,N.; Tsitrin,T.; Stockwell,T.; Amedeo,P.; Bishop,B.; Edworthy,P.; Gupta,N.; Katzel,D.; Li,K.; Schobel,S.; Shrivastava,S.; Thovarai,V.; Wang,S.; Webster,R.; Webby,R.; Krauss,S.; Bao,Y.; Sanders,R.; Dernovoy,D.; Kiryutin,B.; Lipman,D.J.; Tatusova,T.
EPI377988	NA	Netherlands	2008-Jan-01	EPI_ISL_122097	A/mallard/Netherlands/58/2008		Import from public-domain	Wentworth,D.E.; Dugan,V.; Halpin,R.; Lin,X.; Wester,E.; Ghedin,E.; Fedorova,N.; Tsitrin,T.; Stockwell,T.; Amedeo,P.; Bishop,B.; Edworthy,P.; Gupta,N.; Katzel,D.; Li,K.; Schobel,S.; Shrivastava,S.; Thovarai,V.; Wang,S.; Webster,R.; Webby,R.; Krauss,S.; Bao,Y.; Sanders,R.; Dernovoy,D.; Kiryutin,B.; Lipman,D.J.; Tatusova,T.
EPI377980	NA	Netherlands	2008-Jan-01	EPI_ISL_122096	A/mallard/Netherlands/37/2008		Import from public-domain	Wentworth,D.E.; Dugan,V.; Halpin,R.; Lin,X.; Wester,E.; Ghedin,E.; Fedorova,N.; Tsitrin,T.; Stockwell,T.; Amedeo,P.; Bishop,B.; Edworthy,P.; Gupta,N.; Katzel,D.; Li,K.; Schobel,S.; Shrivastava,S.; Thovarai,V.; Wang,S.; Webster,R.; Webby,R.; Krauss,S.; Bao,Y.; Sanders,R.; Dernovoy,D.; Kiryutin,B.; Lipman,D.J.; Tatusova,T.
EPI1850616	NA	France	2021-Jan-18	EPI_ISL_1259399	A/red knot/France/21P003249/2021	Anses (Ploufragan-Plouzané)	ANSES Agence Nationale De Securite Sanitaire De L'alimentation	
EPI1850649	NA	France	2020-Dec-07	EPI_ISL_1263541	A/mallard/France/20P017917/2020	Anses (Ploufragan-Plouzané)	ANSES Agence Nationale De Securite Sanitaire De L'alimentation	
EPI1634505	NA	Ukraine	2011-Feb-15	EPI_ISL_399425	A/mallard/Ukraine/AN-23-15-02/2011		Import from public-domain	Muzyka,D.; Rula,O.; Tkachenko,S.; Muzyka,N.; Stegniy,B.; Pantin-Jackwood,M.; Koethe,S.; Pohlmann,A.; Beer,M.
EPI1850608	NA	France	2021-Jan-22	EPI_ISL_1259397	A/curlew/France/21P003648/2021	Anses (Ploufragan-Plouzané)	ANSES Agence Nationale De Securite Sanitaire De L'alimentation	
EPI1530560	NA	Netherlands	2014-Dec-24	EPI_ISL_373084	A/Black-headed Gull/Netherlands/22/2014		Import from public-domain	Poen,M.J.; Verhagen,J.H.; Vuong,O.; Scheuer,R.D.; Pas,S.D.; Fouchier,R.A.M.
EPI1530606	NA	Netherlands	2014-Dec-24	EPI_ISL_373084	A/Black-headed Gull/Netherlands/22/2014		Import from public-domain	Poen,M.J.; Verhagen,J.H.; Vuong,O.; Scheuer,R.D.; Pas,S.D.; Fouchier,R.A.M.
EPI1530607	NA	Netherlands	2014-Dec-24	EPI_ISL_373084	A/Black-headed Gull/Netherlands/22/2014		Import from public-domain	Poen,M.J.; Verhagen,J.H.; Vuong,O.; Scheuer,R.D.; Pas,S.D.; Fouchier,R.A.M.
EPI1817512	NA	Ukraine	2011-Nov-16	EPI_ISL_697588	A/White-fronted Goose/Ukraine/Kurganskoe-2-16- 11/2011		Import from public-domain	Muzyka,D.; Muzyka,N.; Rula,O.; Koethe,S.; Pohlmann,A.; Pantin-Jackwood,M.; Beer,M.
EPI573059	NA	Netherlands	2003-Mar-12	EPI_ISL_174321	A/Ostrich/Netherlands/03006814/2003	Wageningen Bioveterinary Research	Wageningen Bioveterinary Research	Desniwati,Desniwati; Heutink,Rene; Bouwstra, Ruth; Koch,Guus
EPI141374	NA	Germany	2006-Jan-01	EPI_ISL_16389	A/Perdix perdix/Germany/R44/06		Import from public-domain	
EPI1139754	NA	Japan	2006-Jan-01	EPI_ISL_291114	A/w-swan/Fukushima/364/06	National Institute of Animal Health	National Institute of Animal Health	Y,Uchida
EPI993146	NA	Japan	2007-Jan-01	EPI_ISL_262488	A/duck/Chiba/51/07	National Institute of Animal Health	National Institute of Animal Health	Junki, M
EPI540222	NA	Bangladesh	2007-Oct-31	EPI_ISL_165787	A/duck/Bangladesh/1052/2007	Institute of Epidemiology Disease Control and Research (IEDCR) & Bangladesh National Influenza Centre (NIC)	Centers for Disease Control and Prevention	Gerloff, Nancy; Simpson, Natosha; Davis, C. Todd
EPI540214	NA	Bangladesh	2007-Oct-31	EPI_ISL_165786	A/duck/Bangladesh/1051/2007	Institute of Epidemiology Disease Control and Research (IEDCR) & Bangladesh National Influenza Centre (NIC)	Centers for Disease Control and Prevention	Gerloff, Nancy; Simpson, Natosha; Davis, C. Todd
EPI1139821	NA	Japan	2006-Jan-01	EPI_ISL_291122	A/duck/Niigata/514/06	National Institute of Animal Health	National Institute of Animal Health	Y,Uchida
EPI1139796	NA	Japan	2006-Jan-01	EPI_ISL_291119	A/duck/Shimane/12/06	National Institute of Animal Health	National Institute of Animal Health	Y,Uchida
EPI1139763	NA	Japan	2006-Jan-01	EPI_ISL_291115	A/duck/Tsukuba/578/06	National Institute of Animal Health	National Institute of Animal Health	Y,Uchida
EPI1120635	NA	Japan	2007-Jan-01	EPI_ISL_288013	A/duck/Chiba/47/07	National Institute of Animal Health	National Institute of Animal Health	Y,Uchida
EPI1120579	NA	Japan	2006-Jan-01	EPI_ISL_288006	A/duck/Shimane/20/06	National Institute of Animal Health	National Institute of Animal Health	Y,Uchida
EPI855848	NA	Japan	2013-Nov-01	EPI_ISL_237168	A/duck/Nara/291118/2013	National Institute of Animal Health	National Institute of Animal Health	
EPI884264	NA	Russian Fede	2014-Sep-11	EPI_ISL_240681	A/mallard/Chany/126K-2/2014	Research Institute of Experimental and Clinical Medicine	Research Institute of Experimental and Clinical Medicine	Sharshov, K.A.; Sobolev, I.A.; Xinxin, Li; Alikina, T.Y.; Glushenko, A.V.; Kurskaya, O.G.; Kabilov, M.R.; Alekseev, A.Yu.; Shestopalov, A.M.
EPI1635083	NA	Cambodia	2018-Feb-24	EPI_ISL_399743	A/duck/Cambodia/C50W8M1/2018		Import from public-domain	Vijaykrishna,D.; Deng,Y.M.; Grau,M.L.; Kay,M.; Suttie,A.; Horwood,P.F.; Kalpravidh,W.; Claes,F.; Osbjer,K.; Dussart,P.; Barr,I.G.; Karlsson,E.A.; Dhanasekaran,V.; Deng,Y.-M.; L Grau,M.
EPI1175868	NA	Mongolia	2011-Sep-03	EPI_ISL_298540	A/duck/Mongolia/565/2011		Import from public-domain	Sakoda,Y.; Okamatsu,M.; Matsuno,K.; Jizou,M.

EPI1133986	NA	Mongolia	2011-Sep-03	EPI_ISL_290235	A/duck/Mongolia/258/2011	Import from public-domain	Sakoda,Y.; Okamatsu,M.; Matsuno,K.; Jizou,M.
EPI408361	NA	Korea, Repub	2007-Mar-01	EPI_ISL_70556	A/duck/Korea/BC10/2007	Import from public-domain	Kang,H.-M.; Park,H.-Y.; Lee,K.-J.; Choi,J.-G.; Lee,E.-K.; Song,B.-M.; Lee,H.-S.; Lee,Y.-J.; Kim,K.-I.
EPI227121	NA	France	2005-Jan-01	EPI_ISL_64861	A/duck/France/05054a/2005	Import from public-domain	Le Gall-Recule,G.
EPI160656	NA	Japan	2007-Jan-01	EPI_ISL_15042	A/duck/Hokkaido/167/2007	Import from public-domain	
EPI1189802	NA	Japan	2007-Jan-01	EPI_ISL_15042	A/duck/Hokkaido/167/2007	Import from public-domain	
EPI68166	NA	Italy	2004-Jan-01	EPI_ISL_7494	A/duck/Italy/775/2004	Import from public-domain	
EPI63244	NA	Italy	2005-Jan-01	EPI_ISL_7160	A/teal/Italy/3812/2005	Import from public-domain	
EPI377634	NA	Georgia	2010-Jan-01	EPI_ISL_122034	A/northern shoveler/Georgia/1/2010	Import from public-domain	Wentworth,D.E.; Dugan,V.; Halpin,R.; Lin,X.; Wester,E.; Ghedin,E.; Fedorova,N.; Tsitrin,T.; Stockwell,T.; Amedeo,P.; Bishop,B.; Edworthy,P.; Gupta,N.; Katzel,D.; Li,K.; Schobel,S.; Shrivastava,S.; Thovarai,V.; Wang,S.; Webster,R.; Webby,R.; Krauss,S.; Bao,Y.; Sanders,R.; Dernovoy,D.; Kiryutin,B.; Lipman,D.J.; Tatusova,T.
EPI534752	NA	Finland	2010-Jan-01	EPI_ISL_163883	A/teal/Finland/9201/2010	Import from public-domain	Lindh,E.A.; Ek-Kommonen,C.; Vaananen,V.-M.; Alasaari,J.; Vaheri,A.; Vapalahti,O.; Huovilainen,A.
EPI251613	NA	Sweden	2003-Aug-30	EPI_ISL_73363	A/common teal/Sweden/1/2003	Import from public-domain	Fouchier,R.
EPI296515	NA	Netherlands	2006-Jan-01	EPI_ISL_84557	A/mallard/Netherlands/65/2006	Import from public-domain	The NIAID Influenza Genome Sequencing Consortium
EPI618037	NA	Georgia	2011-Feb-11	EPI_ISL_189692	A/mallard/Republic of Georgia/12/2011	Import from public-domain	Wentworth,D.E.; Halpin,R.A.; Lin,X.; Simenauer,A.; Akopov,A.; Mohan,M.; Fedorova,N.; Tsitrin,T.; Puri,V.; Stockwell,T.; Amedeo,P.; Bishop,B.; Gupta,N.; Hoover,J.; Katzel,D.; Schobel,S.; Shrivastava,S.; Thovarai,V.; Bao,Y.; Sanders,R.; Zhdanov,S.; Kiryutin,B.; Lipman,D.J.; Tatusova,T.; Fouchier,R.A.M.
EPI619234	NA	Sweden	2005-Nov-23	EPI_ISL_189318	A/mallard/Sweden/5927/2005	Import from public-domain	Wentworth,D.E.; Halpin,R.A.; Lin,X.; Bera,J.; Ransier,A.; Fedorova,N.; Tsitrin,T.; McLellan,M.; Stockwell,T.; Amedeo,P.; Appalla,L.; Bishop,B.; Edworthy,P.; Gupta,N.; Hoover,J.; Katzel,D.; Li,K.; Schobel,S.; Shrivastava,S.; Thovarai,V.; Wang,S.; Fouchier,R.; Osterhaus,A.; Olsen,B.; Wille,M.; Latorre-Margalef,N.; Tolf,C.; Bao,Y.; Sanders,R.; Zhdanov,S.; Kiryutin,B.; Lipman,D.J.; Tatusova,T.; Waldenstrom,J.
EPI619235	NA	Sweden	2005-Nov-23	EPI_ISL_189318	A/mallard/Sweden/5927/2005	Import from public-domain	Wentworth,D.E.; Halpin,R.A.; Lin,X.; Bera,J.; Ransier,A.; Fedorova,N.; Tsitrin,T.; McLellan,M.; Stockwell,T.; Amedeo,P.; Appalla,L.; Bishop,B.; Edworthy,P.; Gupta,N.; Hoover,J.; Katzel,D.; Li,K.; Schobel,S.; Shrivastava,S.; Thovarai,V.; Wang,S.; Fouchier,R.; Osterhaus,A.; Olsen,B.; Wille,M.; Latorre-Margalef,N.; Tolf,C.; Bao,Y.; Sanders,R.; Zhdanov,S.; Kiryutin,B.; Lipman,D.J.; Tatusova,T.; Waldenstrom,J.
EPI514845	NA	Sweden	2003-Sep-07	EPI_ISL_158205	A/mallard/Sweden/1883/2003	Import from public-domain	Wentworth,D.E.; Halpin,R.A.; Lin,X.; Bera,J.; Ransier,A.; Fedorova,N.; Tsitrin,T.; McLellan,M.; Stockwell,T.; Amedeo,P.; Appalla,L.; Bishop,B.; Edworthy,P.; Gupta,N.; Hoover,J.; Katzel,D.; Li,K.; Schobel,S.; Shrivastava,S.; Thovarai,V.; Wang,S.; Fouchier,R.; Osterhaus,A.; Olsen,B.; Wille,M.; Latorre-Margalef,N.; Tolf,C.; Bao,Y.; Sanders,R.; Zhdanov,S.; Kiryutin,B.; Lipman,D.J.; Tatusova,T.; Waldenstrom,J.
EPI463398	NA	Sweden	2009-Dec-12	EPI_ISL_144353	A/mallard/Sweden/107892/2009	Import from public-domain	Wille,M.; Tolf,C.; Avril,A.; Latorre-Margalef,N.; Wallerstrom,S.; Olsen,B.; Waldenstrom,J.; Bengtsson,D.; Waldenstom,J.
EPI377928	NA	Sweden	2005-Jan-01	EPI_ISL_122091	A/mallard/Sweden/107/2005	Import from public-domain	Wentworth,D.E.; Dugan,V.; Halpin,R.; Lin,X.; Wester,E.; Ghedin,E.; Fedorova,N.; Tsitrin,T.; Stockwell,T.; Amedeo,P.; Bishop,B.; Edworthy,P.; Gupta,N.; Katzel,D.; Li,K.; Schobel,S.; Shrivastava,S.; Thovarai,V.; Wang,S.; Webster,R.; Webby,R.; Krauss,S.; Bao,Y.; Sanders,R.; Dernovoy,D.; Kiryutin,B.; Lipman,D.J.; Tatusova,T.
EPI377912	NA	Sweden	2005-Jan-01	EPI_ISL_122089	A/mallard/Sweden/93/2005	Import from public-domain	Wentworth,D.E.; Dugan,V.; Halpin,R.; Lin,X.; Wester,E.; Ghedin,E.; Fedorova,N.; Tsitrin,T.; Stockwell,T.; Amedeo,P.; Bishop,B.; Edworthy,P.; Gupta,N.; Katzel,D.; Li,K.; Schobel,S.; Shrivastava,S.; Thovarai,V.; Wang,S.; Webster,R.; Webby,R.; Krauss,S.; Bao,Y.; Sanders,R.; Dernovoy,D.; Kiryutin,B.; Lipman,D.J.; Tatusova,T.

EPI377879	NA	Sweden	2005-Jan-01	EPI_ISL_122085	A/mallard/Sweden/60/2005		Import from public-domain	Wentworth,D.E.; Dugan,V.; Halpin,R.; Lin,X.; Wester,E.; Ghedin,E.; Fedorova,N.; Tsitrin,T.; Stockwell,T.; Amedeo,P.; Bishop,B.; Edworthy,P.; Gupta,N.; Katzel,D.; Li,K.; Schobel,S.; Shrivastava,S.; Thovarai,V.; Wang,S.; Webster,R.; Webby,R.; Krauss,S.; Bao,Y.; Sanders,R.; Dernovoy,D.; Kiryutin,B.; Lipman,D.J.; Tatusova,T.
EPI377880	NA	Sweden	2005-Jan-01	EPI_ISL_122085	A/mallard/Sweden/60/2005		Import from public-domain	Wentworth,D.E.; Dugan,V.; Halpin,R.; Lin,X.; Wester,E.; Ghedin,E.; Fedorova,N.; Tsitrin,T.; Stockwell,T.; Amedeo,P.; Bishop,B.; Edworthy,P.; Gupta,N.; Katzel,D.; Li,K.; Schobel,S.; Shrivastava,S.; Thovarai,V.; Wang,S.; Webster,R.; Webby,R.; Krauss,S.; Bao,Y.; Sanders,R.; Dernovoy,D.; Kiryutin,B.; Lipman,D.J.; Tatusova,T.
EPI377871	NA	Sweden	2005-Jan-01	EPI_ISL_122084	A/mallard/Sweden/51/2005		Import from public-domain	Wentworth,D.E.; Dugan,V.; Halpin,R.; Lin,X.; Wester,E.; Ghedin,E.; Fedorova,N.; Tsitrin,T.; Stockwell,T.; Amedeo,P.; Bishop,B.; Edworthy,P.; Gupta,N.; Katzel,D.; Li,K.; Schobel,S.; Shrivastava,S.; Thovarai,V.; Wang,S.; Webster,R.; Webby,R.; Krauss,S.; Bao,Y.; Sanders,R.; Dernovoy,D.; Kiryutin,B.; Lipman,D.J.; Tatusova,T.
EPI377791	NA	Sweden	2002-Jan-01	EPI_ISL_122074	A/mallard/Sweden/57/2002		Import from public-domain	Wentworth,D.E.; Dugan,V.; Halpin,R.; Lin,X.; Wester,E.; Ghedin,E.; Fedorova,N.; Tsitrin,T.; Stockwell,T.; Amedeo,P.; Bishop,B.; Edworthy,P.; Gupta,N.; Katzel,D.; Li,K.; Schobel,S.; Shrivastava,S.; Thovarai,V.; Wang,S.; Webster,R.; Webby,R.; Krauss,S.; Bao,Y.; Sanders,R.; Dernovoy,D.; Kiryutin,B.; Lipman,D.J.; Tatusova,T.
EPI377674	NA	Sweden	2002-Jan-01	EPI_ISL_122039	A/mallard/Sweden/99/2002		Import from public-domain	Wentworth,D.E.; Dugan,V.; Halpin,R.; Lin,X.; Wester,E.; Ghedin,E.; Fedorova,N.; Tsitrin,T.; Stockwell,T.; Amedeo,P.; Bishop,B.; Edworthy,P.; Gupta,N.; Katzel,D.; Li,K.; Schobel,S.; Shrivastava,S.; Thovarai,V.; Wang,S.; Webster,R.; Webby,R.; Krauss,S.; Bao,Y.; Sanders,R.; Dernovoy,D.; Kiryutin,B.; Lipman,D.J.; Tatusova,T.
EPI377658	NA	Sweden	2002-Jan-01	EPI_ISL_122037	A/mallard/Sweden/6/2002		Import from public-domain	Wentworth,D.E.; Dugan,V.; Halpin,R.; Lin,X.; Wester,E.; Ghedin,E.; Fedorova,N.; Tsitrin,T.; Stockwell,T.; Amedeo,P.; Bishop,B.; Edworthy,P.; Gupta,N.; Katzel,D.; Li,K.; Schobel,S.; Shrivastava,S.; Thovarai,V.; Wang,S.; Webster,R.; Webby,R.; Krauss,S.; Bao,Y.; Sanders,R.; Dernovoy,D.; Kiryutin,B.; Lipman,D.J.; Tatusova,T.
EPI377569	NA	Sweden	2005-Jan-01	EPI_ISL_122026	A/mallard/Sweden/88/2005		Import from public-domain	Wentworth,D.E.; Dugan,V.; Halpin,R.; Lin,X.; Wester,E.; Ghedin,E.; Fedorova,N.; Tsitrin,T.; Stockwell,T.; Amedeo,P.; Bishop,B.; Edworthy,P.; Gupta,N.; Katzel,D.; Li,K.; Schobel,S.; Shrivastava,S.; Thovarai,V.; Wang,S.; Webster,R.; Webby,R.; Krauss,S.; Bao,Y.; Sanders,R.; Dernovoy,D.; Kiryutin,B.; Lipman,D.J.; Tatusova,T.
EPI377570	NA	Sweden	2005-Jan-01	EPI_ISL_122026	A/mallard/Sweden/88/2005		Import from public-domain	Wentworth,D.E.; Dugan,V.; Halpin,R.; Lin,X.; Wester,E.; Ghedin,E.; Fedorova,N.; Tsitrin,T.; Stockwell,T.; Amedeo,P.; Bishop,B.; Edworthy,P.; Gupta,N.; Katzel,D.; Li,K.; Schobel,S.; Shrivastava,S.; Thovarai,V.; Wang,S.; Webster,R.; Webby,R.; Krauss,S.; Bao,Y.; Sanders,R.; Dernovoy,D.; Kiryutin,B.; Lipman,D.J.; Tatusova,T.
EPI377560	NA	Sweden	2005-Jan-01	EPI_ISL_122025	A/mallard/Sweden/58/2005		Import from public-domain	Wentworth,D.E.; Dugan,V.; Halpin,R.; Lin,X.; Wester,E.; Ghedin,E.; Fedorova,N.; Tsitrin,T.; Stockwell,T.; Amedeo,P.; Bishop,B.; Edworthy,P.; Gupta,N.; Katzel,D.; Li,K.; Schobel,S.; Shrivastava,S.; Thovarai,V.; Wang,S.; Webster,R.; Webby,R.; Krauss,S.; Bao,Y.; Sanders,R.; Dernovoy,D.; Kiryutin,B.; Lipman,D.J.; Tatusova,T.
EPI377561	NA	Sweden	2005-Jan-01	EPI_ISL_122025	A/mallard/Sweden/58/2005		Import from public-domain	Wentworth,D.E.; Dugan,V.; Halpin,R.; Lin,X.; Wester,E.; Ghedin,E.; Fedorova,N.; Tsitrin,T.; Stockwell,T.; Amedeo,P.; Bishop,B.; Edworthy,P.; Gupta,N.; Katzel,D.; Li,K.; Schobel,S.; Shrivastava,S.; Thovarai,V.; Wang,S.; Webster,R.; Webby,R.; Krauss,S.; Bao,Y.; Sanders,R.; Dernovoy,D.; Kiryutin,B.; Lipman,D.J.; Tatusova,T.
EPI251771	NA	Sweden	2002-Oct-25	EPI_ISL_73386	A/mallard/Sweden/6/2002		Import from public-domain	Fouchier,R.
EPI182059	NA	Sweden	2005-Jan-01	EPI_ISL_30806	A/mallard/Sweden/65/2005		Import from public-domain	
EPI182035	NA	Sweden	2005-Jan-01	EPI_ISL_30803	A/mallard/Sweden/45/2005		Import from public-domain	
EPI21402	NA	Italy	2000-Jan-01	EPI_ISL_3915	A/mallard/Italy/208/2000		Import from public-domain	

EPI118090	NA	Italy	2000-Jan-01	EPI_ISL_3915	A/mallard/Italy/208/2000		Import from public-domain	
EPI21400	NA	Italy	1999-Jan-01	EPI_ISL_3914	A/mallard/Italy/36/1999		Import from public-domain	
EPI21398	NA	Italy	1999-Jan-01	EPI_ISL_3913	A/mallard/Italy/35/1999		Import from public-domain	
EPI1778060	NA	Bangladesh	2019-Feb-07	EPI_ISL_503509	A/garganey/Bangladesh/38920/2019		Import from public-domain	Barman,S.; Turner,J.C.; Hasan,M.; Akhtar,S.; Franks,J.; El-Shesheny,R.; Walker,D.; Seiler,P.; Mukherjee,N.; Kercher,L.; McKenzie,P.; Feeroz,M.; Webby,R.J.
EPI182396	NA	Russian Fede	1991-Aug-15	EPI_ISL_30877	A/duck/Altai/1285/1991		Import from public-domain	
EPI181914	NA	Japan	2006-Jan-01	EPI_ISL_30782	A/duck/Niigata/514/2006		Import from public-domain	Tsukamoto,K.; Shishido,M.
EPI1860046	NA	Switzerland	2021-Feb-01	EPI_ISL_2234809	A/yellow-legged_gull/Switzerland/15-0039_21VIR3035/2021	Istituto Zooprofilattico Sperimentale delle Venezie, EU/OIE/Reference Laboratory and FAO Reference Centre for AI and ND	Istituto Zooprofilattico Sperimentale Delle Venezie	Albini, S.; H?ssy, D.; Nenniger, C.; V?gtlin, A.; Zimmer, G.; Zecchin, B.; Fusaro, A.; Schivo, A.; Salviato, A.; Giussani, E.; Monne, I.; Terregino, C.
EPI1765867	NA	China	2018-Dec-06	EPI_ISL_499089	A/mallard/Shanghai/JDS120662/2018		Import from public-domain	Tang,L.
EPI1841779	NA	Netherlands	2018-Mar-07	EPI_ISL_814560	A/mallard/Netherlands/18003625-015/2018	Wageningen Bioveterinary Research	Wageningen Bioveterinary Research	Beerens, Nancy; Harders, Frank; Pritz-Verschuren, Sylvia; Roose, Marit; Germeraad, Evelien; Engelsma, Marc; Bossers, Alex; Heutink, Rene
EPI1230126	NA	Netherlands	2013-Jan-18	EPI_ISL_309837	A/Turkey/Netherlands/13001007/2013	Wageningen Bioveterinary Research	Wageningen Bioveterinary Research	Bergervoet, Saskia; Heutink, Rene; Harders, Frank; Beerens, Nancy
EPI1139761	NA	China	2018-Jan-06	EPI_ISL_291111	A/Jiangsu/1/2018(H7N4)	Jiangsu Provincial Center for Disease Control & Prevention	Jiangsu Provincial Center for Disease Control & Prevention	
EPI1536852	NA	China	2018-Jan-06	EPI_ISL_376123	A/Jiangsu/1/2018		Import from public-domain	Cui,L.; Zhao,K.; Ge,Y.; Chen,Y.; Zhu,X.; Wu,T.; Shi,Z.; Qi,X.; Zhu,F.
EPI1330280	NA	Bangladesh	2016-Jul-15	EPI_ISL_333097	A/duck/Bangladesh/18D659/2016	icddr,b International Centre for Diarrhoeal Disease Research, Bangladesh	Centers for Disease Control and Prevention	Yang, G.; Chowdury, S; Hodges, E; Rahman, M.Z.; Jang, Y.; Hossain, M.E.; Jones, J.; Stark, T.; Di, H.; Cook, P.W.; Ghosh, S.; Azziz-Baumgartner, E.; Barnes, J.; Wentworth, D.; Kennedy, E.; Davis, C.T.
EPI484579	NA	Bangladesh	2011-Apr-03	EPI_ISL_149697	A/duck/Bangladesh/727/2011	icddr,b International Centre for Diarrhoeal Disease Research, Bangladesh	Centers for Disease Control and Prevention	Gerloff, Nancy; Simpson, Natosha; Davis, C. Todd
EPI484576	NA	Bangladesh	2011-Apr-03	EPI_ISL_149695	A/duck/Bangladesh/814/2011	icddr,b International Centre for Diarrhoeal Disease Research, Bangladesh	Centers for Disease Control and Prevention	Gerloff, Nancy; Simpson, Natosha; Davis, C. Todd
EPI484574	NA	Bangladesh	2011-Apr-06	EPI_ISL_149694	A/duck/Bangladesh/983/2011	icddr,b International Centre for Diarrhoeal Disease Research, Bangladesh	Centers for Disease Control and Prevention	Gerloff, Nancy; Simpson, Natosha; Davis, C. Todd
EPI966044	NA	Netherlands	2011-Mar-10	EPI_ISL_257181	A/chicken/Netherlands/11004004/2011		Import from public-domain	Verhagen,J.H.; Lexmond,P.; Vuong,O.; Schutten,M.; Guldemeester,J.; Osterhaus,A.D.; Elbers,A.R.; Slaterus,R.; Hornman,M.; Koch,G.; Fouchier,R.A.; Osterhaus,A.D.M.E.; Elbers,A.R.W.; Fouchier,R.A.M.
EPI966045	NA	Netherlands	2010-Jun-04	EPI_ISL_257180	A/chicken/Netherlands/10009401/2010		Import from public-domain	Verhagen,J.H.; Lexmond,P.; Vuong,O.; Schutten,M.; Guldemeester,J.; Osterhaus,A.D.; Elbers,A.R.; Slaterus,R.; Hornman,M.; Koch,G.; Fouchier,R.A.; Osterhaus,A.D.M.E.; Elbers,A.R.W.; Fouchier,R.A.M.
EPI966043	NA	Netherlands	2010-May-16	EPI_ISL_257177	A/chicken/Netherlands/10007882/2010		Import from public-domain	Verhagen,J.H.; Lexmond,P.; Vuong,O.; Schutten,M.; Guldemeester,J.; Osterhaus,A.D.; Elbers,A.R.; Slaterus,R.; Hornman,M.; Koch,G.; Fouchier,R.A.; Osterhaus,A.D.M.E.; Elbers,A.R.W.; Fouchier,R.A.M.
EPI1229873	NA	Netherlands	2011-Mar-09	EPI_ISL_309804	A/Chicken/Netherlands/11004004/2011	Wageningen Bioveterinary Research	Wageningen Bioveterinary Research	Bergervoet, Saskia; Heutink, Rene; Harders, Frank; Beerens, Nancy
EPI1229841	NA	Netherlands	2010-Jun-04	EPI_ISL_309800	A/Chicken/Netherlands/10009401/2010	Wageningen Bioveterinary Research	Wageningen Bioveterinary Research	Bergervoet, Saskia; Heutink, Rene; Harders, Frank; Beerens, Nancy
EPI1229833	NA	Netherlands	2010-May-14	EPI_ISL_309799	A/Chicken/Netherlands/10007882/2010	Wageningen Bioveterinary Research	Wageningen Bioveterinary Research	Bergervoet, Saskia; Heutink, Rene; Harders, Frank; Beerens, Nancy
EPI1326747	NA	China	2018-Jan-25	EPI_ISL_332396	A/chicken/Jiangsu/3/2018		Harbin Veterinary Research Institute (CAAS)	Yulei,Li;Lu,Zhao; Minghui,Li; Song,Jin; Yanbing,Li
EPI1326739	NA	China	2018-Jan-25	EPI_ISL_332395	A/chicken/Jiangsu/2/2018		Harbin Veterinary Research Institute (CAAS)	Yulei,Li;Lu,Zhao;Minghui,Li;Song,Jin;Yanbing,Li
EPI1326422	NA	China	2018-Jan-25	EPI_ISL_332358	A/chicken/Jiangsu/1/2018		Harbin Veterinary Research Institute (CAAS)	Yulei,Li;Lu,Zhao;Minghui,Li;Song,Jin;Yanbing,Li
EPI1150084	NA	China	2018-Jan-06	EPI_ISL_293291	A/Duck/Jiangsu/16/2018(H7N4)	Jiangsu Provincial Center for Disease Control & Prevention	Jiangsu Provincial Center for Disease Control & Prevention	
EPI1150076	NA	China	2018-Jan-06	EPI_ISL_293290	A/Duck/Jiangsu/13/2018(H7N4)	Jiangsu Provincial Center for Disease Control & Prevention	Jiangsu Provincial Center for Disease Control & Prevention	
EPI1150068	NA		2018-Jan-06	EPI_ISL_293289	A/Duck/Jiangsu/12/2018(H7N4)	Jiangsu Provincial Center for Disease Control & Prevention	Jiangsu Provincial Center for Disease Control & Prevention	
EPI1150059	NA	China	2018-Jan-06	EPI_ISL_293288	A/Duck/Jiangsu/8/2018(H7N4)	Jiangsu Provincial Center for Disease Control & Prevention	Jiangsu Provincial Center for Disease Control & Prevention	

EPI1150051	NA	China	2018-Jan-06	EPI_ISL_293287	A/Duck/Jiangsu/4/2018(H7N4)	Jiangsu Provincial Center for Disease Control & Prevention	Jiangsu Provincial Center for Disease Control & Prevention	
EPI1635120	NA	Cambodia	2018-Apr-10	EPI_ISL_399748	A/duck/Cambodia/14T-24-1-D11/2018		Import from public-domain	Vijaykrishna,D.; Deng,Y.M.; Grau,M.L.; Kay,M.; Suttie,A.; Horwood,P.F.; Kalpravidh,W.; Claes,F.; Osbjer,K.; Dussart,P.; Barr,I.G.; Karlsson,E.A.; Dhanasekaran,V.; Deng,Y.-M.; L Grau,M.
EPI1635076	NA	Cambodia	2018-Mar-22	EPI_ISL_399742	A/duck/Cambodia/12T-24-1-D17/2018		Import from public-domain	Vijaykrishna,D.; Deng,Y.M.; Grau,M.L.; Kay,M.; Suttie,A.; Horwood,P.F.; Kalpravidh,W.; Claes,F.; Osbjer,K.; Dussart,P.; Barr,I.G.; Karlsson,E.A.; Dhanasekaran,V.; Deng,Y.-M.; L Grau,M.
EPI1635068	NA	Cambodia	2018-Mar-22	EPI_ISL_399741	A/duck/Cambodia/12T-24-1-D3/2018		Import from public-domain	Vijaykrishna,D.; Deng,Y.M.; Grau,M.L.; Kay,M.; Suttie,A.; Horwood,P.F.; Kalpravidh,W.; Claes,F.; Osbjer,K.; Dussart,P.; Barr,I.G.; Karlsson,E.A.; Dhanasekaran,V.; Deng,Y.-M.; L Grau,M.
EPI1635061	NA	Cambodia	2018-Mar-22	EPI_ISL_399740	A/duck/Cambodia/12T-24-1-D1/2018		Import from public-domain	Vijaykrishna,D.; Deng,Y.M.; Grau,M.L.; Kay,M.; Suttie,A.; Horwood,P.F.; Kalpravidh,W.; Claes,F.; Osbjer,K.; Dussart,P.; Barr,I.G.; Karlsson,E.A.; Dhanasekaran,V.; Deng,Y.-M.; L Grau,M.
EPI1635054	NA	Cambodia	2018-Mar-22	EPI_ISL_399738	A/duck/Cambodia/12T-24-1-D7/2018		Import from public-domain	Vijaykrishna,D.; Deng,Y.M.; Grau,M.L.; Kay,M.; Suttie,A.; Horwood,P.F.; Kalpravidh,W.; Claes,F.; Osbjer,K.; Dussart,P.; Barr,I.G.; Karlsson,E.A.; Dhanasekaran,V.; Deng,Y.-M.; L Grau,M.
EPI384005	NA	Thailand	2010-Dec-22	EPI_ISL_124283	A/duck/Thailand/CU-9744C /2010		Import from public-domain	Chaiyawong,S.; Nonthabenjawan,N.; Bunpapong,N.; Boonyapisitsopa,S.; Jairak,W.; Wongphatcharachai,M.; Thanawongnuwech,R.; Amornsin,A.
EPI383959	NA	Thailand	2010-Dec-22	EPI_ISL_124272	A/duck/Thailand/CU-9754C/2010		Import from public-domain	Chaiyawong,S.; Nonthabenjawan,N.; Bunpapong,N.; Boonyapisitsopa,S.; Jairak,W.; Wongphatcharachai,M.; Thanawongnuwech,R.; Amornsin,A.
EPI335969	NA	Russian Fede	2008-Aug-30	EPI_ISL_96113	A/teal/Chany/7119/2008		Import from public-domain	Sivay,M.V.; Sharshov,K.A.; Baranovich,T.; Govorkova,E.A.; Yurlov,A.K.; Shestopalov,A.M.; Webby,R.J.
EPI1140136	NA	Netherlands	2012-Feb-12	EPI_ISL_291186	A/Common Teal/Netherlands/12002960/2012	Wageningen Bioveterinary Research	Wageningen Bioveterinary Research	Bergervoet, Saskia; Heutink, Rene; Harders, Frank; Beerens, Nancy
EPI1777990	NA	Bangladesh	2019-Jan-15	EPI_ISL_503500	A/mallard/Bangladesh/37909/2019		Import from public-domain	Barman,S.; Turner,J.C.; Hasan,M.; Akhtar,S.; Franks,J.; El-Shesheny,R.; Walker,D.; Seiler,P.; Mukherjee,N.; Kercher,L.; McKenzie,P.; Feeroz,M.; Webby,R.J.
EPI890803	NA	Netherlands	2011-Oct-19	EPI_ISL_243398	A/mallard duck/Netherlands/30/2011		Import from public-domain	Poen,M.J.; Verhagen,J.H.; Vuong,O.; Scheuer,R.D.; Pas,S.D.; Fouchier,R.A.M.
EPI618099	NA	Sweden	2002-Nov-02	EPI_ISL_189468	A/mallard/Sweden/396/2002		Import from public-domain	Wentworth,D.E.; Halpin,R.A.; Lin,X.; Bera,J.; Ransier,A.; Fedorova,N.; Tsitrin,T.; McLellan,M.; Stockwell,T.; Amedeo,P.; Appalla,L.; Bishop,B.; Edworthy,P.; Gupta,N.; Hoover,J.; Katzel,D.; Li,K.; Schobel,S.; Shrivastava,S.; Thovarai,V.; Wang,S.; Fouchier,R.; Osterhaus,A.; Olsen,B.; Wille,M.; Latorre-Margalef,N.; Tolf,C.; Bao,Y.; Sanders,R.; Zhdanov,S.; Kiryutin,B.; Lipman,D.J.; Tatusova,T.; Waldenstrom,J.
EPI619113	NA	Sweden	2009-Nov-05	EPI_ISL_189340	A/mallard/Sweden/101165/2009		Import from public-domain	Wentworth,D.E.; Halpin,R.A.; Lin,X.; Bera,J.; Ransier,A.; Fedorova,N.; Tsitrin,T.; McLellan,M.; Stockwell,T.; Amedeo,P.; Appalla,L.; Bishop,B.; Edworthy,P.; Gupta,N.; Hoover,J.; Katzel,D.; Li,K.; Schobel,S.; Shrivastava,S.; Thovarai,V.; Wang,S.; Fouchier,R.; Osterhaus,A.; Olsen,B.; Wille,M.; Latorre-Margalef,N.; Tolf,C.; Bao,Y.; Sanders,R.; Zhdanov,S.; Kiryutin,B.; Lipman,D.J.; Tatusova,T.; Waldenstrom,J.
EPI619106	NA	Sweden	2009-Sep-03	EPI_ISL_189339	A/mallard/Sweden/99377/2009		Import from public-domain	Wentworth,D.E.; Halpin,R.A.; Lin,X.; Bera,J.; Ransier,A.; Fedorova,N.; Tsitrin,T.; McLellan,M.; Stockwell,T.; Amedeo,P.; Appalla,L.; Bishop,B.; Edworthy,P.; Gupta,N.; Hoover,J.; Katzel,D.; Li,K.; Schobel,S.; Shrivastava,S.; Thovarai,V.; Wang,S.; Fouchier,R.; Osterhaus,A.; Olsen,B.; Wille,M.; Latorre-Margalef,N.; Tolf,C.; Bao,Y.; Sanders,R.; Zhdanov,S.; Kiryutin,B.; Lipman,D.J.; Tatusova,T.; Waldenstrom,J.

EPI619127	NA	Sweden	2004-Nov-25	EPI_ISL_189330	A/mallard/Sweden/7242/2004		Import from public-domain	Wentworth,D.E.; Halpin,R.A.; Lin,X.; Bera,J.; Ransier,A.; Fedorova,N.; Tsitrin,T.; McLellan,M.; Stockwell,T.; Amedeo,P.; Appalla,L.; Bishop,B.; Edworthy,P.; Gupta,N.; Hoover,J.; Katzel,D.; Li,K.; Schobel,S.; Shrivastava,S.; Thovarai,V.; Wang,S.; Fouchier,R.; Osterhaus,A.; Olsen,B.; Wille,M.; Latorre-Margalef,N.; Tolf,C.; Bao,Y.; Sanders,R.; Zhdanov,S.; Kiryutin,B.; Lipman,D.J.; Tatusova,T.; Waldenstrom,J.
EPI619193	NA	Sweden	2003-Sep-02	EPI_ISL_189327	A/mallard/Sweden/3240/2003		Import from public-domain	Wentworth,D.E.; Halpin,R.A.; Lin,X.; Bera,J.; Ransier,A.; Fedorova,N.; Tsitrin,T.; McLellan,M.; Stockwell,T.; Amedeo,P.; Appalla,L.; Bishop,B.; Edworthy,P.; Gupta,N.; Hoover,J.; Katzel,D.; Li,K.; Schobel,S.; Shrivastava,S.; Thovarai,V.; Wang,S.; Fouchier,R.; Osterhaus,A.; Olsen,B.; Wille,M.; Latorre-Margalef,N.; Tolf,C.; Bao,Y.; Sanders,R.; Zhdanov,S.; Kiryutin,B.; Lipman,D.J.; Tatusova,T.; Waldenstrom,J.
EPI619280	NA	Sweden	2003-May-27	EPI_ISL_189315	A/mallard/Sweden/2834/2003		Import from public-domain	Wentworth,D.E.; Halpin,R.A.; Lin,X.; Bera,J.; Ransier,A.; Fedorova,N.; Tsitrin,T.; McLellan,M.; Stockwell,T.; Amedeo,P.; Appalla,L.; Bishop,B.; Edworthy,P.; Gupta,N.; Hoover,J.; Katzel,D.; Li,K.; Schobel,S.; Shrivastava,S.; Thovarai,V.; Wang,S.; Fouchier,R.; Osterhaus,A.; Olsen,B.; Wille,M.; Latorre-Margalef,N.; Tolf,C.; Bao,Y.; Sanders,R.; Zhdanov,S.; Kiryutin,B.; Lipman,D.J.; Tatusova,T.; Waldenstrom,J.
EPI463359	NA	Sweden	2009-Oct-22	EPI_ISL_144312	A/mallard/Sweden/100546/2009		Import from public-domain	Wille,M.; Tolf,C.; Avril,A.; Latorre-Margalef,N.; Wallerstrom,S.; Olsen,B.; Waldenstrom,J.; Bengtsson,D.; Waldenstom,J.
EPI439199	NA	Georgia	2011-Feb-11	EPI_ISL_138652	A/mallard/Republic of Georgia/12/2011		Import from public-domain	Lewis,N.S.; Javakhishvili,Z.; Russell,C.A.; Machablishvili,A.; Lexmond,P.; Verhagen,J.H.; Vuong,O.; Onashvili,T.; Donduashvili,M.; Smith,D.J.; Fouchier,R.A.; Fouchier,R.A.M.
EPI1903213	NA	Bangladesh	2020-Jan-27	EPI_ISL_4071639	A/duck/Bangladesh/42981/2020		Import from public-domain	Barman,S.; Turner,J.C.; Hasan,M.; Akhtar,S.; Franks,J.; El-Shesheny,R.; Walker,D.; Seiler,P.; Mukherjee,N.; Kercher,L.; McKenzie,P.; Feeroz,M.; Webby,R.J.
EPI1903126	NA	Bangladesh	2020-Jan-27	EPI_ISL_4071627	A/duck/Bangladesh/42987/2020		Import from public-domain	Barman,S.; Turner,J.C.; Hasan,M.; Akhtar,S.; Franks,J.; El-Shesheny,R.; Walker,D.; Seiler,P.; Mukherjee,N.; Kercher,L.; McKenzie,P.; Feeroz,M.; Webby,R.J.
EPI1778247	NA	Bangladesh	2019-Mar-29	EPI_ISL_503535	A/duck/Bangladesh/39729/2019		Import from public-domain	Barman,S.; Turner,J.C.; Hasan,M.; Akhtar,S.; Franks,J.; El-Shesheny,R.; Walker,D.; Seiler,P.; Mukherjee,N.; Kercher,L.; McKenzie,P.; Feeroz,M.; Webby,R.J.
EPI1777914	NA	Bangladesh	2019-Feb-08	EPI_ISL_503489	A/duck/Bangladesh/38557/2019		Import from public-domain	Barman,S.; Turner,J.C.; Hasan,M.; Akhtar,S.; Franks,J.; El-Shesheny,R.; Walker,D.; Seiler,P.; Mukherjee,N.; Kercher,L.; McKenzie,P.; Feeroz,M.; Webby,R.J.
EPI1777869	NA	Bangladesh	2019-Jan-17	EPI_ISL_503483	A/duck/Bangladesh/37626/2019		Import from public-domain	Barman,S.; Turner,J.C.; Hasan,M.; Akhtar,S.; Franks,J.; El-Shesheny,R.; Walker,D.; Seiler,P.; Mukherjee,N.; Kercher,L.; McKenzie,P.; Feeroz,M.; Webby,R.J.
EPI1777851	NA	Bangladesh	2019-Jan-17	EPI_ISL_503481	A/duck/Bangladesh/37630/2019		Import from public-domain	Barman,S.; Turner,J.C.; Hasan,M.; Akhtar,S.; Franks,J.; El-Shesheny,R.; Walker,D.; Seiler,P.; Mukherjee,N.; Kercher,L.; McKenzie,P.; Feeroz,M.; Webby,R.J.
EPI1326825	NA	China	2018-Jan-25	EPI_ISL_332401	A/duck/Jiangsu/2/2018		Harbin Veterinary Research Institute (CAAS)	Yulei,Li;Lu,Zhao; Minghui,Li; Song,Jin; Yanbing,Li
EPI1326809	NA	China	2018-Jan-25	EPI_ISL_332399	A/duck/Jiangsu/1/2018		Harbin Veterinary Research Institute (CAAS)	Yulei,Li;Lu,Zhao; Minghui,Li; Song,Jin; Yanbing,Li
EPI1150043	NA		2018-Jan-06	EPI_ISL_293286	A/Chicken/Jiangsu/103/2018(H7N4)	Jiangsu Provincial Center for Disease Control & Prevention	Jiangsu Provincial Center for Disease Control & Prevention	
EPI1139892	NA	China	2018-Jan-06	EPI_ISL_291131	A/Chicken/Jiangsu/1/2018(H7N4)	Jiangsu Provincial Center for Disease Control & Prevention	Jiangsu Provincial Center for Disease Control & Prevention	
EPI1813747	NA	Denmark	2020-Oct-30	EPI_ISL_644737	A/peregrine falcon/Denmark/13776-1/2020-10-30(H5N5)	Statens Serumm Institute	Statens Serum Institute	Charlotte,Hjulsager
EPI1811672	NA	Russian Fede	2020-Oct-02	EPI_ISL_626648	A/goose/Russian_Federation/Omsk/1680-6/2020	Federal Centre for Animal Health (ARRIAH)	Animal and Plant Health Agency (APHA)	
EPI296578	NA	Netherlands	2006-Jan-01	EPI_ISL_84565	A/herring gull/Netherlands/4/2006		Import from public-domain	The NIAID Influenza Genome Sequencing Consortium
EPI1858331	NA	Slovenia	2020-Dec-24	EPI_ISL_1665267	A/mute_swan/Slovenia/1914-20_21VIR959-5/2020	Istituto Zooprofilattico Sperimentale delle Venezie, EU/OIE/Reference Laboratory and FAO Reference Centre for AI and ND	Istituto Zooprofilattico Sperimentale Delle Venezie	Slavec, B.; Racnik, J.; Zorman Rojs, Olga.; Zecchin, B.; Fusaro, A.; Pastori, A.; Schivo, A.; Salviato, A.; Monne, I.; Terregino, C.
EPI1815185	NA	United Kingdo	2020-Nov-24	EPI_ISL_683999	A/mute swan/Wales/048068/2020	Animal and Plant Health Agency (APHA)	Animal and Plant Health Agency (APHA)	
EPI1815169	NA	United Kingdo	2020-Nov-24	EPI_ISL_683997	A/mute swan/Wales/048069/2020	Animal and Plant Health Agency (APHA)	Animal and Plant Health Agency (APHA)	
EPI1811662	NA	Russian Fede	2020-Oct-02	EPI_ISL_626647	A/chicken/Russian_Federation/Omsk/1680-10/2020	Federal Centre for Animal Health (ARRIAH)	Animal and Plant Health Agency (APHA)	

EPI1229889	NA	Netherlands	2011-May-10	EPI_ISL_309806	A/Chicken/Netherlands/11008325/2011	Wageningen Bioveterinary Research	Wageningen Bioveterinary Research	Bergervoet, Saskia; Heutink, Rene; Harders, Frank; Beerens, Nancy
EPI855678	NA	Japan	2014-Oct-01	EPI_ISL_237150	A/duck/Kyoto/261007/2014	National Institute of Animal Health	National Institute of Animal Health	
EPI1640125	NA	Russian Fede	2018-Sep-29	EPI_ISL_395083	A/mallard/Novosibirsk region/964k/2018	Research Institute of Experimental and Clinical Medicine	Research Institute of Experimental and Clinical Medicine	
EPI1640117	NA	Russian Fede	2018-Oct-01	EPI_ISL_395082	A/mallard/Novosibirsk region/999k/2018	Research Institute of Experimental and Clinical Medicine	Research Institute of Experimental and Clinical Medicine	
EPI1635050	NA	Cambodia	2018-Apr-05	EPI_ISL_399737	A/duck/Cambodia/C70W14M/2018		Import from public-domain	Vijaykrishna,D.; Deng,Y.M.; Grau,M.L.; Kay,M.; Suttie,A.; Horwood,P.F.; Kalpravidh,W.; Claes,F.; Osbjer,K.; Dussart,P.; Barr,I.G.; Karlsson,E.A.; Dhanasekaran,V.; Deng,Y.-M.; L Grau,M.
EPI296634	NA	Netherlands	2006-Jan-01	EPI_ISL_84572	A/northern shoveler/Netherlands/1/2006		Import from public-domain	The NIAID Influenza Genome Sequencing Consortium
EPI181971	NA	Netherlands	2005-Jan-01	EPI_ISL_30795	A/common teal/Netherlands/1/2005		Import from public-domain	
EPI889272	NA	Netherlands	2008-Dec-29	EPI_ISL_243403	A/Eurasian wigeon/Netherlands/2/2008		Import from public-domain	Poen,M.J.; Verhagen,J.H.; Vuong,O.; Scheuer,R.D.; Pas,S.D.; Fouchier,R.A.M.
EPI1014283	NA	Netherlands	2006-Jul-24	EPI_ISL_267765	A/mallard duck/Netherlands/11/2006		Import from public-domain	
EPI1013765	NA	Netherlands	2006-Jul-24	EPI_ISL_267263	A/mallard duck/Netherlands/13/2006		Import from public-domain	
EPI961545	NA	Sweden	2014-Oct-13	EPI_ISL_256315	A/Mallard/Sweden/689/2014		Import from public-domain	Wille,M.; Lindqvist,K.; Muradrasoli,S.; Olsen,B.; Jarhult,J.; Jarhult,J.D.
EPI961533	NA	Sweden	2014-Sep-15	EPI_ISL_256311	A/Mallard/Sweden/527/2014		Import from public-domain	Wille,M.; Lindqvist,K.; Muradrasoli,S.; Olsen,B.; Jarhult,J.; Jarhult,J.D.
EPI890701	NA	Netherlands	2006-Jul-24	EPI_ISL_243549	A/mallard duck/Netherlands/14/2006		Import from public-domain	Poen,M.J.; Verhagen,J.H.; Vuong,O.; Scheuer,R.D.; Pas,S.D.; Fouchier,R.A.M.
EPI296475	NA	Netherlands	2006-Jan-01	EPI_ISL_84552	A/mallard/Netherlands/30/2006		Import from public-domain	The NIAID Influenza Genome Sequencing Consortium
EPI618063	NA	Sweden	2005-Sep-11	EPI_ISL_189473	A/mallard/Sweden/8004/2005		Import from public-domain	Wentworth,D.E.; Halpin,R.A.; Lin,X.; Bera,J.; Ransier,A.; Fedorova,N.; Tsitrin,T.; McLellan,M.; Stockwell,T.; Amedeo,P.; Appalla,L.; Bishop,B.; Edworthy,P.; Gupta,N.; Hoover,J.; Katzel,D.; Li,K.; Schobel,S.; Shrivastava,S.; Thovarai,V.; Wang,S.; Fouchier,R.; Osterhaus,A.; Olsen,B.; Wille,M.; Latorre-Margalef,N.; Tolf,C.; Bao,Y.; Sanders,R.; Zhdanov,S.; Kiryutin,B.; Lipman,D.J.; Tatusova,T.; Waldenstrom,J.
EPI618132	NA	Sweden	2005-Oct-05	EPI_ISL_189471	A/mallard/Sweden/6039/2005		Import from public-domain	Wentworth,D.E.; Halpin,R.A.; Lin,X.; Bera,J.; Ransier,A.; Fedorova,N.; Tsitrin,T.; McLellan,M.; Stockwell,T.; Amedeo,P.; Appalla,L.; Bishop,B.; Edworthy,P.; Gupta,N.; Hoover,J.; Katzel,D.; Li,K.; Schobel,S.; Shrivastava,S.; Thovarai,V.; Wang,S.; Fouchier,R.; Osterhaus,A.; Olsen,B.; Wille,M.; Latorre-Margalef,N.; Tolf,C.; Bao,Y.; Sanders,R.; Zhdanov,S.; Kiryutin,B.; Lipman,D.J.; Tatusova,T.; Waldenstrom,J.
EPI618072	NA	Sweden	2006-Oct-20	EPI_ISL_189466	A/mallard/Sweden/51671/2006		Import from public-domain	Wentworth,D.E.; Halpin,R.A.; Lin,X.; Bera,J.; Ransier,A.; Fedorova,N.; Tsitrin,T.; McLellan,M.; Stockwell,T.; Amedeo,P.; Appalla,L.; Bishop,B.; Edworthy,P.; Gupta,N.; Hoover,J.; Katzel,D.; Li,K.; Schobel,S.; Shrivastava,S.; Thovarai,V.; Wang,S.; Fouchier,R.; Osterhaus,A.; Olsen,B.; Wille,M.; Latorre-Margalef,N.; Tolf,C.; Bao,Y.; Sanders,R.; Zhdanov,S.; Kiryutin,B.; Lipman,D.J.; Tatusova,T.; Waldenstrom,J.
EPI618796	NA	Sweden	2007-Jun-07	EPI_ISL_189368	A/mallard/Sweden/64476/2007		Import from public-domain	Wentworth,D.E.; Halpin,R.A.; Lin,X.; Bera,J.; Ransier,A.; Fedorova,N.; Tsitrin,T.; McLellan,M.; Stockwell,T.; Amedeo,P.; Appalla,L.; Bishop,B.; Edworthy,P.; Gupta,N.; Hoover,J.; Katzel,D.; Li,K.; Schobel,S.; Shrivastava,S.; Thovarai,V.; Wang,S.; Fouchier,R.; Osterhaus,A.; Olsen,B.; Wille,M.; Latorre-Margalef,N.; Tolf,C.; Bao,Y.; Sanders,R.; Zhdanov,S.; Kiryutin,B.; Lipman,D.J.; Tatusova,T.; Waldenstrom,J.
EPI618909	NA	Sweden	2006-Oct-21	EPI_ISL_189362	A/mallard/Sweden/51726/2006		Import from public-domain	Wentworth,D.E.; Halpin,R.A.; Lin,X.; Bera,J.; Ransier,A.; Fedorova,N.; Tsitrin,T.; McLellan,M.; Stockwell,T.; Amedeo,P.; Appalla,L.; Bishop,B.; Edworthy,P.; Gupta,N.; Hoover,J.; Katzel,D.; Li,K.; Schobel,S.; Shrivastava,S.; Thovarai,V.; Wang,S.; Fouchier,R.; Osterhaus,A.; Olsen,B.; Wille,M.; Latorre-Margalef,N.; Tolf,C.; Bao,Y.; Sanders,R.; Zhdanov,S.; Kiryutin,B.; Lipman,D.J.; Tatusova,T.; Waldenstrom,J.

EPI618894	NA	Sweden	2006-Oct-20	EPI_ISL_189361	A/mallard/Sweden/51645/2006	Import from public-domain	Wentworth,D.E.; Halpin,R.A.; Lin,X.; Bera,J.; Ransier,A.; Fedorova,N.; Tsitrin,T.; McLellan,M.; Stockwell,T.; Amedeo,P.; Appalla,L.; Bishop,B.; Edworthy,P.; Gupta,N.; Hoover,J.; Katzel,D.; Li,K.; Schobel,S.; Shrivastava,S.; Thovarai,V.; Wang,S.; Fouchier,R.; Osterhaus,A.; Olsen,B.; Wille,M.; Latorre-Margalef,N.; Tolf,C.; Bao,Y.; Sanders,R.; Zhdanov,S.; Kiryutin,B.; Lipman,D.J.; Tatusova,T.; Waldenstrom,J.
EPI618880	NA	Sweden	2006-Oct-19	EPI_ISL_189360	A/mallard/Sweden/51619/2006	Import from public-domain	Wentworth,D.E.; Halpin,R.A.; Lin,X.; Bera,J.; Ransier,A.; Fedorova,N.; Tsitrin,T.; McLellan,M.; Stockwell,T.; Amedeo,P.; Appalla,L.; Bishop,B.; Edworthy,P.; Gupta,N.; Hoover,J.; Katzel,D.; Li,K.; Schobel,S.; Shrivastava,S.; Thovarai,V.; Wang,S.; Fouchier,R.; Osterhaus,A.; Olsen,B.; Wille,M.; Latorre-Margalef,N.; Tolf,C.; Bao,Y.; Sanders,R.; Zhdanov,S.; Kiryutin,B.; Lipman,D.J.; Tatusova,T.; Waldenstrom,J.
EPI618866	NA	Sweden	2006-Oct-19	EPI_ISL_189359	A/mallard/Sweden/51582/2006	Import from public-domain	Wentworth,D.E.; Halpin,R.A.; Lin,X.; Bera,J.; Ransier,A.; Fedorova,N.; Tsitrin,T.; McLellan,M.; Stockwell,T.; Amedeo,P.; Appalla,L.; Bishop,B.; Edworthy,P.; Gupta,N.; Hoover,J.; Katzel,D.; Li,K.; Schobel,S.; Shrivastava,S.; Thovarai,V.; Wang,S.; Fouchier,R.; Osterhaus,A.; Olsen,B.; Wille,M.; Latorre-Margalef,N.; Tolf,C.; Bao,Y.; Sanders,R.; Zhdanov,S.; Kiryutin,B.; Lipman,D.J.; Tatusova,T.; Waldenstrom,J.
EPI618856	NA	Sweden	2006-Oct-18	EPI_ISL_189358	A/mallard/Sweden/51548/2006	Import from public-domain	Wentworth,D.E.; Halpin,R.A.; Lin,X.; Bera,J.; Ransier,A.; Fedorova,N.; Tsitrin,T.; McLellan,M.; Stockwell,T.; Amedeo,P.; Appalla,L.; Bishop,B.; Edworthy,P.; Gupta,N.; Hoover,J.; Katzel,D.; Li,K.; Schobel,S.; Shrivastava,S.; Thovarai,V.; Wang,S.; Fouchier,R.; Osterhaus,A.; Olsen,B.; Wille,M.; Latorre-Margalef,N.; Tolf,C.; Bao,Y.; Sanders,R.; Zhdanov,S.; Kiryutin,B.; Lipman,D.J.; Tatusova,T.; Waldenstrom,J.
EPI618845	NA	Sweden	2005-Sep-17	EPI_ISL_189357	A/mallard/Sweden/8023/2005	Import from public-domain	Wentworth,D.E.; Halpin,R.A.; Lin,X.; Bera,J.; Ransier,A.; Fedorova,N.; Tsitrin,T.; McLellan,M.; Stockwell,T.; Amedeo,P.; Appalla,L.; Bishop,B.; Edworthy,P.; Gupta,N.; Hoover,J.; Katzel,D.; Li,K.; Schobel,S.; Shrivastava,S.; Thovarai,V.; Wang,S.; Fouchier,R.; Osterhaus,A.; Olsen,B.; Wille,M.; Latorre-Margalef,N.; Tolf,C.; Bao,Y.; Sanders,R.; Zhdanov,S.; Kiryutin,B.; Lipman,D.J.; Tatusova,T.; Waldenstrom,J.
EPI619012	NA	Sweden	2004-Jun-18	EPI_ISL_189352	A/mallard/Sweden/4411/2004	Import from public-domain	Wentworth,D.E.; Halpin,R.A.; Lin,X.; Bera,J.; Ransier,A.; Fedorova,N.; Tsitrin,T.; McLellan,M.; Stockwell,T.; Amedeo,P.; Appalla,L.; Bishop,B.; Edworthy,P.; Gupta,N.; Hoover,J.; Katzel,D.; Li,K.; Schobel,S.; Shrivastava,S.; Thovarai,V.; Wang,S.; Fouchier,R.; Osterhaus,A.; Olsen,B.; Wille,M.; Latorre-Margalef,N.; Tolf,C.; Bao,Y.; Sanders,R.; Zhdanov,S.; Kiryutin,B.; Lipman,D.J.; Tatusova,T.; Waldenstrom,J.
EPI619085	NA	Sweden	2004-Jun-18	EPI_ISL_189351	A/mallard/Sweden/4401/2004	Import from public-domain	Wentworth,D.E.; Halpin,R.A.; Lin,X.; Bera,J.; Ransier,A.; Fedorova,N.; Tsitrin,T.; McLellan,M.; Stockwell,T.; Amedeo,P.; Appalla,L.; Bishop,B.; Edworthy,P.; Gupta,N.; Hoover,J.; Katzel,D.; Li,K.; Schobel,S.; Shrivastava,S.; Thovarai,V.; Wang,S.; Fouchier,R.; Osterhaus,A.; Olsen,B.; Wille,M.; Latorre-Margalef,N.; Tolf,C.; Bao,Y.; Sanders,R.; Zhdanov,S.; Kiryutin,B.; Lipman,D.J.; Tatusova,T.; Waldenstrom,J.
EPI618987	NA	Sweden	2004-May-31	EPI_ISL_189350	A/mallard/Sweden/4258/2004	Import from public-domain	Wentworth,D.E.; Halpin,R.A.; Lin,X.; Bera,J.; Ransier,A.; Fedorova,N.; Tsitrin,T.; McLellan,M.; Stockwell,T.; Amedeo,P.; Appalla,L.; Bishop,B.; Edworthy,P.; Gupta,N.; Hoover,J.; Katzel,D.; Li,K.; Schobel,S.; Shrivastava,S.; Thovarai,V.; Wang,S.; Fouchier,R.; Osterhaus,A.; Olsen,B.; Wille,M.; Latorre-Margalef,N.; Tolf,C.; Bao,Y.; Sanders,R.; Zhdanov,S.; Kiryutin,B.; Lipman,D.J.; Tatusova,T.; Waldenstrom,J.
EPI619091	NA	Sweden	2007-Sep-01	EPI_ISL_189337	A/mallard/Sweden/59517/2007	Import from public-domain	Wentworth,D.E.; Halpin,R.A.; Lin,X.; Bera,J.; Ransier,A.; Fedorova,N.; Tsitrin,T.; McLellan,M.; Stockwell,T.; Amedeo,P.; Appalla,L.; Bishop,B.; Edworthy,P.; Gupta,N.; Hoover,J.; Katzel,D.; Li,K.; Schobel,S.; Shrivastava,S.; Thovarai,V.; Wang,S.; Fouchier,R.; Osterhaus,A.; Olsen,B.; Wille,M.; Latorre-Margalef,N.; Tolf,C.; Bao,Y.; Sanders,R.; Zhdanov,S.; Kiryutin,B.; Lipman,D.J.; Tatusova,T.; Waldenstrom,J.

EPI619169	NA	Sweden	2007-Aug-29	EPI_ISL_189336	A/mallard/Sweden/59475/2007	Import from public-domain	Wentworth,D.E.; Halpin,R.A.; Lin,X.; Bera,J.; Ransier,A.; Fedorova,N.; Tsitrin,T.; McLellan,M.; Stockwell,T.; Amedeo,P.; Appalla,L.; Bishop,B.; Edworthy,P.; Gupta,N.; Hoover,J.; Katzel,D.; Li,K.; Schobel,S.; Shrivastava,S.; Thovarai,V.; Wang,S.; Fouchier,R.; Osterhaus,A.; Olsen,B.; Wille,M.; Latorre-Margalef,N.; Tolf,C.; Bao,Y.; Sanders,R.; Zhdanov,S.; Kiryutin,B.; Lipman,D.J.; Tatusova,T.; Waldenstrom,J.
EPI619162	NA	Sweden	2006-Nov-03	EPI_ISL_189335	A/mallard/Sweden/58256/2006	Import from public-domain	Wentworth,D.E.; Halpin,R.A.; Lin,X.; Bera,J.; Ransier,A.; Fedorova,N.; Tsitrin,T.; McLellan,M.; Stockwell,T.; Amedeo,P.; Appalla,L.; Bishop,B.; Edworthy,P.; Gupta,N.; Hoover,J.; Katzel,D.; Li,K.; Schobel,S.; Shrivastava,S.; Thovarai,V.; Wang,S.; Fouchier,R.; Osterhaus,A.; Olsen,B.; Wille,M.; Latorre-Margalef,N.; Tolf,C.; Bao,Y.; Sanders,R.; Zhdanov,S.; Kiryutin,B.; Lipman,D.J.; Tatusova,T.; Waldenstrom,J.
EPI619155	NA	Sweden	2006-Oct-12	EPI_ISL_189334	A/mallard/Sweden/51156/2006	Import from public-domain	Wentworth,D.E.; Halpin,R.A.; Lin,X.; Bera,J.; Ransier,A.; Fedorova,N.; Tsitrin,T.; McLellan,M.; Stockwell,T.; Amedeo,P.; Appalla,L.; Bishop,B.; Edworthy,P.; Gupta,N.; Hoover,J.; Katzel,D.; Li,K.; Schobel,S.; Shrivastava,S.; Thovarai,V.; Wang,S.; Fouchier,R.; Osterhaus,A.; Olsen,B.; Wille,M.; Latorre-Margalef,N.; Tolf,C.; Bao,Y.; Sanders,R.; Zhdanov,S.; Kiryutin,B.; Lipman,D.J.; Tatusova,T.; Waldenstrom,J.
EPI619148	NA	Sweden	2006-Jun-07	EPI_ISL_189333	A/mallard/Sweden/50055/2006	Import from public-domain	Wentworth,D.E.; Halpin,R.A.; Lin,X.; Bera,J.; Ransier,A.; Fedorova,N.; Tsitrin,T.; McLellan,M.; Stockwell,T.; Amedeo,P.; Appalla,L.; Bishop,B.; Edworthy,P.; Gupta,N.; Hoover,J.; Katzel,D.; Li,K.; Schobel,S.; Shrivastava,S.; Thovarai,V.; Wang,S.; Fouchier,R.; Osterhaus,A.; Olsen,B.; Wille,M.; Latorre-Margalef,N.; Tolf,C.; Bao,Y.; Sanders,R.; Zhdanov,S.; Kiryutin,B.; Lipman,D.J.; Tatusova,T.; Waldenstrom,J.
EPI619141	NA	Sweden	2005-Sep-11	EPI_ISL_189332	A/mallard/Sweden/8005/2005	Import from public-domain	Wentworth,D.E.; Halpin,R.A.; Lin,X.; Bera,J.; Ransier,A.; Fedorova,N.; Tsitrin,T.; McLellan,M.; Stockwell,T.; Amedeo,P.; Appalla,L.; Bishop,B.; Edworthy,P.; Gupta,N.; Hoover,J.; Katzel,D.; Li,K.; Schobel,S.; Shrivastava,S.; Thovarai,V.; Wang,S.; Fouchier,R.; Osterhaus,A.; Olsen,B.; Wille,M.; Latorre-Margalef,N.; Tolf,C.; Bao,Y.; Sanders,R.; Zhdanov,S.; Kiryutin,B.; Lipman,D.J.; Tatusova,T.; Waldenstrom,J.
EPI619134	NA	Sweden	2005-Sep-08	EPI_ISL_189331	A/mallard/Sweden/7996/2005	Import from public-domain	Wentworth,D.E.; Halpin,R.A.; Lin,X.; Bera,J.; Ransier,A.; Fedorova,N.; Tsitrin,T.; McLellan,M.; Stockwell,T.; Amedeo,P.; Appalla,L.; Bishop,B.; Edworthy,P.; Gupta,N.; Hoover,J.; Katzel,D.; Li,K.; Schobel,S.; Shrivastava,S.; Thovarai,V.; Wang,S.; Fouchier,R.; Osterhaus,A.; Olsen,B.; Wille,M.; Latorre-Margalef,N.; Tolf,C.; Bao,Y.; Sanders,R.; Zhdanov,S.; Kiryutin,B.; Lipman,D.J.; Tatusova,T.; Waldenstrom,J.
EPI619120	NA	Sweden	2005-Sep-20	EPI_ISL_189329	A/mallard/Sweden/5389/2005	Import from public-domain	Wentworth,D.E.; Halpin,R.A.; Lin,X.; Bera,J.; Ransier,A.; Fedorova,N.; Tsitrin,T.; McLellan,M.; Stockwell,T.; Amedeo,P.; Appalla,L.; Bishop,B.; Edworthy,P.; Gupta,N.; Hoover,J.; Katzel,D.; Li,K.; Schobel,S.; Shrivastava,S.; Thovarai,V.; Wang,S.; Fouchier,R.; Osterhaus,A.; Olsen,B.; Wille,M.; Latorre-Margalef,N.; Tolf,C.; Bao,Y.; Sanders,R.; Zhdanov,S.; Kiryutin,B.; Lipman,D.J.; Tatusova,T.; Waldenstrom,J.
EPI619186	NA	Sweden	2003-Jun-12	EPI_ISL_189326	A/mallard/Sweden/2990/2003	Import from public-domain	Wentworth,D.E.; Halpin,R.A.; Lin,X.; Bera,J.; Ransier,A.; Fedorova,N.; Tsitrin,T.; McLellan,M.; Stockwell,T.; Amedeo,P.; Appalla,L.; Bishop,B.; Edworthy,P.; Gupta,N.; Hoover,J.; Katzel,D.; Li,K.; Schobel,S.; Shrivastava,S.; Thovarai,V.; Wang,S.; Fouchier,R.; Osterhaus,A.; Olsen,B.; Wille,M.; Latorre-Margalef,N.; Tolf,C.; Bao,Y.; Sanders,R.; Zhdanov,S.; Kiryutin,B.; Lipman,D.J.; Tatusova,T.; Waldenstrom,J.
EPI251803	NA	Sweden	2003-May-27	EPI_ISL_73390	A/mallard/Sweden/8/2003	Import from public-domain	Fouchier,R.

EPI190366	NA	Sweden	2005-Jan-01	EPI_ISL_33857	A/mallard/Sweden/4/2005		Import from public-domain	Spiro,D.; Halpin,R.; Bera,J.; Ghedin,E.; Dugan,V.; Hostetler,J.; Fedorova,N.; Hine,E.; Overton,L.; Kim,M.; Szczypinski,B.; Stockwell,T.; Sitz,J.; Katzel,D.; Li,K.; Axelrod,N.; Safford,T.; Amedeo,P.; Schobel,S.; Shrivastava,S.; Wang,S.; Resnick,A.; Thovarai,V.; Bestebroer,T.M.; Munster,V.J.; Lexmond,P.; Rimmelzwaan,G.F.; Osterhaus,A.D.M.E.; Fouchier,R.A.M.; Bao,Y.; Sanders,R.; Dernovoy,D.; Kiryutin,B.; Lipman,D.J.; Tatusova,T.
EPI1811566	NA	Germany	2020-Oct-29	EPI_ISL_614399	A/buzzard/Germany-MV/AI02166/2020	Landesamt für Landwirtschaft, Lebensmittelsicherheit und Fischerei (LALLF)	Friedrich-Loeffler-Institut	
EPI1814629	NA	Belgium	2020-Nov-18	EPI_ISL_660264	A/Gallus_gallus/Belgium/12168_002/2020	Sciensano - Animal Infectious Diseases	Sciensano, Department of Animal Infectious Diseases	Van Borm,Steven; Mathijs,Elisabeth; Vandenbussche,Frank; van den Berg,Thierry; Lambrecht,Bénédicte; Steensels,Mieke
EPI1307455	NA	Netherlands	2008-Sep-28	EPI_ISL_329022	A/ruddy turnstone/Netherlands/1/2008		Import from public-domain	Poen,M.J.; Verhagen,J.H.; Vuong,O.; Scheuer,R.D.; Pas,S.D.; Fouchier,R.A.M.
EPI1307152	NA	Netherlands	2009-Oct-22	EPI_ISL_328985	A/ruddy turnstone/Netherlands/1/2009		Import from public-domain	Poen,M.J.; Verhagen,J.H.; Vuong,O.; Scheuer,R.D.; Pas,S.D.; Fouchier,R.A.M.
EPI1843625	NA	Italy	2020-Nov-28	EPI_ISL_956409	A/common_teal/Italy/20VIR7439-190/2020	Istituto Zooprofilattico Sperimentale delle Venezie, EU/OIE/Reference Laboratory and FAO Reference Centre for AI and ND	Istituto Zooprofilattico Sperimentale Delle Venezie	Zecchin, B.; Fusaro, A.; Milani, A.; Schivo, A.; Salviato, A.; Pastori, A.; Zamperin, G.; Monne, I.; Terregino, C.
EPI990777	NA	Germany	2016-Dec-27	EPI_ISL_262056	A/greylag goose/Germany-NI/AR11353-L02142/2016		Friedrich-Loeffler-Institut	
EPI1123252	NA	Netherlands	2017-May-22	EPI_ISL_288411	A/Go/NL-Utrecht/17006881-001/2017	Wageningen Bioveterinary Research	Wageningen Bioveterinary Research	Beerens, Nancy; Heutink, Rene; Harders, Frank; Verschuren-Pritz, Sylvia; Bossers, Alex; Koch, Guus; Bergervoet, Saskia
EPI712641	NA	China	2014-Dec-02	EPI_ISL_212404	A/Bean Goose/Hubei/chenhu I77/2014_H6N5	State Key Laboratory of Virology and Wuhan Institute of Virology, Chinese Academy of Sciences	Wuhan Institute of Virology	
EPI712631	NA	China	2014-Dec-02	EPI_ISL_212401	A/Bean Goose/Hubei/chenhu 2466(I75)/2014_H6N5	State Key Laboratory of Virology and Wuhan Institute of Virology, Chinese Academy of Sciences	Wuhan Institute of Virology	
EPI712549	NA	China	2014-Dec-02	EPI_ISL_212380	A/Bean Goose/Hubei/chenhu I34/2014_H6N5	State Key Laboratory of Virology and Wuhan Institute of Virology, Chinese Academy of Sciences	Wuhan Institute of Virology	
EPI961480	NA	Russian Fede	2016-Oct-01	EPI_ISL_256301	A/environment/Kamchatka/18/2016		State Research Center of Virology and Biotechnology (VECTOR)	Ivan,Susloparov; Natalya,Goncharova; Natalya,Kolosova; Vasiliy,Marchenko; Alexander,Ryzhikov
EPI874786	NA	Germany	2016-Dec-13	EPI_ISL_240893	A/swan/Germany-SN/R10645/2016		Friedrich-Loeffler-Institut	
EPI1860166	NA	Romania	2021-Jan-22	EPI_ISL_2234824	A/whooper_swan/Romania/10362_21VIR2593-20/2021	Istituto Zooprofilattico Sperimentale delle Venezie, EU/OIE/Reference Laboratory and FAO Reference Centre for AI and ND	Istituto Zooprofilattico Sperimentale Delle Venezie	Onita, I.; Neicut, A.; Raluca, B.; Razvan, M.; Florica, B.; Zecchin, B.; Fusaro, A.; Giussani, E.; Schivo, A.; Salviato, A.; Monne, I.; Terregino, C.
EPI1858339	NA	Romania	2021-Jan-08	EPI_ISL_1665268	A/whooper_swan/Romania/10123_21VIR849-1/2021	Istituto Zooprofilattico Sperimentale delle Venezie, EU/OIE/Reference Laboratory and FAO Reference Centre for AI and ND	Istituto Zooprofilattico Sperimentale Delle Venezie	Onita, I.; Neicut, A.; Raluca, B.; Razvan, M.; Florica, B.; Zecchin, B.; Fusaro, A.; Pastori, A.; Schivo, A.; Salviato, A.; Monne, I.; Terregino, C.
EPI891675	NA	Croatia	2017-Jan-20	EPI_ISL_243698	A/mute swan/Croatia/42/2017		Croatian Veterinary Institute	Savić, Vladimir
EPI873621	NA	Croatia	2016-Dec-27	EPI_ISL_240101	A/mute swan/Croatia/102/2016		Croatian Veterinary Institute	Savić, Vladimir
EPI962073	NA	Hungary	2017-Feb-14	EPI_ISL_256462	A/Mute_swan/Hungary/5879/2017	National Food Chain Safety Office Veterinary Diagnostic Directorate Laboratory for Molecular Biology	Danam.Vet.Molbiol	Adam, Dan
EPI940483	NA	Czech Republ	2017-Feb-09	EPI_ISL_250920	A/mute swan/Czech Republic/2031-17/2017 (H5N5)		State Veterinary Institute Prague	Nagy,Alexander
EPI959443	NA	Poland	2017-Jan-31	EPI_ISL_255917	A/mute swan/Poland/64/2017		National Veterinary Research Institut Poland, PIWet-PIB	Swieton E., Smietanka K.
EPI1117263	NA	Netherlands	2016-Dec-13	EPI_ISL_287565	A/M_Swan/NL-Groningen/16015826-001/2016	Wageningen Bioveterinary Research	Wageningen Bioveterinary Research	Beerens, Nancy; Heutink, Rene; Harders, Frank; Verschuren-Pritz, Sylvia; Bossers, Alex; Koch, Guus; Bergervoet, Saskia
EPI888420	NA	Germany	2017-Jan-22	EPI_ISL_243049	A/turkey/Germany-SH/R425/2017		Friedrich-Loeffler-Institut	
EPI680489	NA	Singapore	2015-Sep-09	EPI_ISL_203617	A/common redshank/Singapore/F83-2/2015(H9N5)		Agri-Food & Veterinary Authority of Singapore	Xinyu, Toh; Ee Leng, Tan; Nurshilla, Harith; Yifan, Wang; Taoqi, Huangfu
EPI680487	NA	Singapore	2015-Sep-09	EPI_ISL_203616	A/common redshank/Singapore/F83-1/2015(H9N5)		Agri-Food & Veterinary Authority of Singapore	Xinyu, Toh; Ee Leng, Tan; Nurshilla, Harith; Yifan, Wang; Taoqi, Huangfu
EPI954622	NA	Italy	2017-Jan-10	EPI_ISL_255189	A/gadwall/Italy/17VIR133-2/2017	Istituto Zooprofilattico Sperimentale Delle Venezie	Istituto Zooprofilattico Sperimentale Delle Venezie	Zecchin, B.; Fusaro, A.; Milani, A.; Schivo, A.; Salviato, A.; Zamperin, G.; Marciano, S.; Ormelli, S.; Terregino, C.; Monne, I.
EPI888601	NA	Italy	2016-Dec-29	EPI_ISL_243085	A/wigeon/Italy/16VIR9616-3/2016	Istituto Zooprofilattico Sperimentale Delle Venezie	Istituto Zooprofilattico Sperimentale Delle Venezie	Silvia,Ormelli; Sabrina,Marciano; Alessia,Schivo; Annalisa,Salviato; Adelaide,Milani; Gianpiero,Zamperini; Bianca,Zecchin; Alice,Fusaro; Calogero,Terregino; Isabella,Monne

EPI1319423	NA	Russian Fede	2018-Jan-23	EPI_ISL_331307	A/teal/Dagestan/1017/2018	Research Institute of Experimental and Clinical Medicine	Research Institute of Experimental and Clinical Medicine	Sharshov, K.; Sobolev, I.; Kurskaya, O.; Murashkina, T.; Alekseev, A.; Gadzhiev, A.; Magomedova, M.; Alikina, T.; Kabilov, M.; Shestopalov, A.
EPI1319415	NA	Russian Fede	2017-Sep-02	EPI_ISL_331306	A/shoveler/Ubinskoe_Lake/43/2017	Research Institute of Experimental and Clinical Medicine	Research Institute of Experimental and Clinical Medicine	Sharshov, K.; Sobolev, I.; Kurskaya, O.; Alekseev, A.; Alikina, T.; Kabilov, M.; Shestopalov, A.
EPI1319358	NA	Russian Fede	2017-Sep-03	EPI_ISL_331295	A/teal/Chany/324/2017		Research Institute of Experimental and Clinical Medicine	Sharshov, K.; Sobolev, I.; Kurskaya, O.; Li, X.; Alekseev, A.; Alikina, T.; Kabilov, M.; Shestopalov, A.
EPI399897	NA	Korea, Repub	2009-Nov-01	EPI_ISL_130355	A/aquatic bird/Korea/CN5/2009		Import from public-domain	Park,S.J.; Park,B.K.; Song,D.S.; Poo,H.; Park,S.-J.; Song,D.-S.
EPI314739	NA	Korea, Repub	2009-Nov-01	EPI_ISL_89350	A/aquatic bird/Korea/CN5/2009		Import from public-domain	Kim,J.-K.; Nam,J.-H.; Kim,E.-H.; Poo,H.
EPI981846	NA	Germany	2017-Jan-30	EPI_ISL_260059	A/cormorant/Germany-SH/R896/2017		Friedrich-Loeffler-Institut	
EPI1330317	NA	Bangladesh	2016-Dec-18	EPI_ISL_333102	A/duck/Bangladesh/17D747/2016	icddr,b International Centre for Diarrhoeal Disease Research, Bangladesh	Centers for Disease Control and Prevention	Yang, G.; Chowdury, S; Hodges, E; Rahman, M.Z.; Jang, Y.; Hossain, M.E.; Jones, J.; Stark, T.; Di, H.; Cook, P.W.; Ghosh, S.; Azziz-Baumgartner, E.; Barnes, J.; Wentworth, D.; Kennedy, E.; Davis, C.T.
EPI986364	NA	Croatia	2017-Mar-07	EPI_ISL_261332	A/chicken/Croatia/104/2017		Croatian Veterinary Institute	Savić, Vladimir
EPI855606	NA	Japan	2014-Nov-01	EPI_ISL_237141	A/duck/Shimane/321112/2014	National Institute of Animal Health	National Institute of Animal Health	
EPI866943	NA	Japan	2015-Oct-30	EPI_ISL_239415	A/duck/Fukui/181015/2015		National Institute of Animal Health	
EPI866833	NA	Japan	2016-Oct-18	EPI_ISL_239393	A/duck/Aichi/231002/2016		National Institute of Animal Health	
EPI866951	NA	Japan	2015-Oct-30	EPI_ISL_239416	A/duck/Fukui/181006/2015		National Institute of Animal Health	
EPI1834681	NA	China	2016-Oct-27	EPI_ISL_707499	A/duck/Hunan/10.27_YYGK57B2-O/2016		Import from public-domain	Bi,Y.; Li,J.; Li,S.; Fu,G.; Jin,T.; Zhang,C.; Yang,Y.; Ma,Z.; Tian,W.; Xiao,S.; Li,L.; Yin,R.; Zhang,Y.; Wang,L.; Qin,Y.; Yao,Z.; Meng,F.; Hu,D.; Li,D.; Wong,G.; Liu,F.; Lv,N.; Fu,L.; Peng,Y.; Ma,J.; Sharshov,K.; Shestopalov,A.; Gulyaeva,M.; Gao,G.F.; Chen,J.; Shi,Y.; Liu,W.J.; Chu,D.; Huang,Y.; Liu,Y.; Liu,L.; Chen,Q.; Shi,W.
EPI1582413	NA	China	2014-Mar-01	EPI_ISL_388614	A/DUCK/China/S1254/2014		Import from public-domain	Yuancheng,Z.
EPI1510642	NA	Mongolia	2018-Sep-03	EPI_ISL_368643	A/duck/Mongolia/850/2018		Import from public-domain	Sakoda,Y.; Okamatsu,M.; Matsuno,K.
EPI1510600	NA	Mongolia	2018-Sep-03	EPI_ISL_368638	A/duck/Mongolia/703/2018		Import from public-domain	Sakoda,Y.; Okamatsu,M.; Matsuno,K.
EPI1510592	NA	Mongolia	2018-Sep-03	EPI_ISL_368637	A/duck/Mongolia/656/2018		Import from public-domain	Sakoda,Y.; Okamatsu,M.; Matsuno,K.
EPI1035769	NA	China	2010-Feb-04	EPI_ISL_273049	A/duck/Guangxi/052/2010		Import from public-domain	Li,M.; Xie,Z.X.; Xie,L.J.; Deng,X.W.; Xie,Q.Z.; Liu,J.B.; Fan,Q.; Pang,Y.S.; Luo,S.S.
EPI461090	NA	China	2009-Nov-27	EPI_ISL_143816	A/duck/Guangxi/GXd-1/2009		Import from public-domain	Xie,Z.; Zhou,C.; Peng,Y.; Liu,J.; Pang,Y.; Deng,X.; Xie,L.; Fan,Q.
EPI600154	NA	China	2013-Dec-08	EPI_ISL_181611	A/migratory duck/Jiangxi/31577/2013		Import from public-domain	Ma,C.; Lam,T.T.Y.; Chai,Y.; Wang,J.; Fan,X.; Hong,W.; Zhang,Y.; Li,L.; Liu,Y.; Smith,D.K.; Webby,R.J.; Peiris,J.S.M.; Zhu,H.; Guan,Y.
EPI1778762	NA	Belgium	2018-Aug-29	EPI_ISL_502613	A/Anas platyrhynchos/Belgium/7828/2018		Import from public-domain	Lambrecht,B.; Steensels,M.; Fusaro,A.; Milani,A.; Pastori,A.; Schivo,A.; Salviato,A.; Zamperin,G.; Monne,I.; Terregino,C.
EPI1774506	NA	Belgium	2018-Aug-29	EPI_ISL_502613	A/Anas platyrhynchos/Belgium/7828/2018		Import from public-domain	Lambrecht,B.; Steensels,M.; Fusaro,A.; Milani,A.; Pastori,A.; Schivo,A.; Salviato,A.; Zamperin,G.; Monne,I.; Terregino,C.
EPI978868	NA	Germany	2017-Feb-06	EPI_ISL_259074	A/common buzzard/Germany-SN/R1117/2017		Friedrich-Loeffler-Institut	
EPI965472	NA	Bangladesh	2015-Feb-22	EPI_ISL_257087	A/black-tailed godwit/Bangladesh/24734/2015		Import from public-domain	Barman,S.; Marinova-Petkova,A.; Hasan,M.K.; Akhtar,S.; Turner,J.C.; Franks,J.; Walker,D.; Seiler,P.; Friedman,K.; Kercher,L.; Kayali,G.; Jones-Engel,L.; McKenzie,P.; Krauss,S.; Webby,R.J.; Feeroz,M.M.; Webster,R.G.
EPI984998	NA	Germany	2017-Jan-22	EPI_ISL_260058	A/grey heron/Germany-SN/R572/2017		Friedrich-Loeffler-Institut	
EPI1785773	NA	China	2014-Oct-17	EPI_ISL_505078	A/wild birds/Hubei/102/2014		Import from public-domain	Ge,Y.; Deng,G.; Chen,H.
EPI1785765	NA	China	2014-Oct-16	EPI_ISL_505077	A/wild birds/Hubei/100/2014		Import from public-domain	Ge,Y.; Deng,G.; Chen,H.
EPI1785757	NA	China	2014-Oct-16	EPI_ISL_505076	A/wild birds/Hubei/99/2014		Import from public-domain	Ge,Y.; Deng,G.; Chen,H.
EPI678380	NA	Singapore	2015-Sep-09	EPI_ISL_203129	A/common redshank/Singapore/F83-2/2015		Import from public-domain	Toh,X.; Tan,E.L.; Harith,N.; Wang,Y.; Huangfu,T.
EPI678379	NA	Singapore	2015-Sep-09	EPI_ISL_203128	A/common redshank/Singapore/F83-1/2015		Import from public-domain	Toh,X.; Tan,E.L.; Harith,N.; Wang,Y.; Huangfu,T.
EPI888421	NA	Germany	2016-Dec-22	EPI_ISL_243050	A/barnacle goose/Germany-SH/R11505/2016		Friedrich-Loeffler-Institut	
EPI890453	NA	Netherlands	2007-Dec-18	EPI_ISL_243505	A/greater white-fronted goose/Netherlands/3/2007		Import from public-domain	Poen,M.J.; Verhagen,J.H.; Vuong,O.; Scheuer,R.D.; Pas,S.D.; Fouchier,R.A.M.
EPI740387	NA	China	2016-Feb-26	EPI_ISL_217877	A/Bean Goose/Hubei/chenhu XVI270-1/2016_H11N5	State Key Laboratory of Virology and Wuhan Institute of Virology, Chinese Academy of Sciences	Wuhan Institute of Virology	
EPI296546	NA	Netherlands	2007-Jan-01	EPI_ISL_84561	A/Bewicks swan/Netherlands/1/2007		Import from public-domain	The NIAID Influenza Genome Sequencing Consortium
EPI1185047	NA	Poland	2017-Feb-02	EPI_ISL_300746	A/swan/Poland/81/2017		National Veterinary Research Institut Poland, PIWet-PIB	Swieton E., Smietanka K.
EPI1860158	NA	Romania	2021-Jan-14	EPI_ISL_2234823	A/whooper_swan/Romania/10213_21VIR2593-27/2021	Istituto Zooprofilattico Sperimentale delle Venezie, EU/OIE/Reference Laboratory and FAO Reference Centre for AI and ND	Istituto Zooprofilattico Sperimentale Delle Venezie	Onita, I.; Neicut, A.; Raluca, B.; Razvan, M.; Florica, B.; Zecchin, B.; Fusaro, A.; Giussani, E.; Schivo, A.; Salviato, A.; Monne, I.; Terregino, C.

EPI1860150	NA	Romania	2021-Jan-12	EPI_ISL_2234822	A/whooper_swan/Romania/10171_21VIR2593-15/2021	Istituto Zooprofilattico Sperimentale delle Venezie, EU/OIE/Reference Laboratory and FAO Reference Centre for AI and ND	Istituto Zooprofilattico Sperimentale Delle Venezie	Onita, I.; Neicut, A.; Raluca, B.; Razvan, M.; Florica, B.; Zecchin, B.; Fusaro, A.; Giussani, E.; Schivo, A.; Salviato, A.; Monne, I.; Terregino, C.
EPI1860142	NA	Romania	2021-Jan-08	EPI_ISL_2234821	A/whooper_swan/Romania/10122_21VIR2593-23/2021	Istituto Zooprofilattico Sperimentale delle Venezie, EU/OIE/Reference Laboratory and FAO Reference Centre for AI and ND	Istituto Zooprofilattico Sperimentale Delle Venezie	Onita, I.; Neicut, A.; Raluca, B.; Razvan, M.; Florica, B.; Zecchin, B.; Fusaro, A.; Giussani, E.; Schivo, A.; Salviato, A.; Monne, I.; Terregino, C.
EPI1858347	NA	Romania	2021-Jan-20	EPI_ISL_1665269	A/whooper_swan/Romania/10311_21VIR849-2/2021	Istituto Zooprofilattico Sperimentale delle Venezie, EU/OIE/Reference Laboratory and FAO Reference Centre for AI and ND	Istituto Zooprofilattico Sperimentale Delle Venezie	Onita, I.; Neicut, A.; Raluca, B.; Razvan, M.; Florica, B.; Zecchin, B.; Fusaro, A.; Pastori, A.; Schivo, A.; Salviato, A.; Monne, I.; Terregino, C.
EPI1858291	NA	Slovakia	2021-Jan-15	EPI_ISL_1665262	A/mute_swan/Slovakia/Pah6_21VIR1086-2/2021	Istituto Zooprofilattico Sperimentale delle Venezie, EU/OIE/Reference Laboratory and FAO Reference Centre for AI and ND	Istituto Zooprofilattico Sperimentale Delle Venezie	Dirb?kov?, Z.; Tin?k, M.; Zecchin, B.; Fusaro, A.; Pastori, A.; Schivo, A.; Salviato, A.; Monne, I.; Terregino, C.
EPI1858283	NA	Slovakia	2021-Jan-29	EPI_ISL_1665261	A/mute_swan/Slovakia/Pah15_21VIR1086-3/2021	Istituto Zooprofilattico Sperimentale delle Venezie, EU/OIE/Reference Laboratory and FAO Reference Centre for AI and ND	Istituto Zooprofilattico Sperimentale Delle Venezie	Dirb?kov?, Z.; Tin?k, M.; Zecchin, B.; Fusaro, A.; Pastori, A.; Schivo, A.; Salviato, A.; Monne, I.; Terregino, C.
EPI1858267	NA	Austria	2021-Feb-01	EPI_ISL_1665259	A/mute_swan/Austria/21014124_21VIR1085-8/2021	Istituto Zooprofilattico Sperimentale delle Venezie, EU/OIE/Reference Laboratory and FAO Reference Centre for AI and ND	Istituto Zooprofilattico Sperimentale Delle Venezie	Wodak, E.; Revilla Fern?ndez, S.; Schmoll, F.; Zecchin, B.; Fusaro, A.; Pastori, A.; Schivo, A.; Salviato, A.; Monne, I.; Terregino, C.
EPI1858259	NA	Austria	2021-Feb-10	EPI_ISL_1665258	A/mute_swan/Austria/21013162_21VIR1085-5/2021	Istituto Zooprofilattico Sperimentale delle Venezie, EU/OIE/Reference Laboratory and FAO Reference Centre for AI and ND	Istituto Zooprofilattico Sperimentale Delle Venezie	Wodak, E.; Revilla Fern?ndez, S.; Schmoll, F.; Zecchin, B.; Fusaro, A.; Pastori, A.; Schivo, A.; Salviato, A.; Monne, I.; Terregino, C.
EPI1858251	NA	Austria	2021-Feb-04	EPI_ISL_1665257	A/mute_swan/Austria/21011165_21VIR1085-2/2021	Istituto Zooprofilattico Sperimentale delle Venezie, EU/OIE/Reference Laboratory and FAO Reference Centre for AI and ND	Istituto Zooprofilattico Sperimentale Delle Venezie	Wodak, E.; Revilla Fern?ndez, S.; Schmoll, F.; Zecchin, B.; Fusaro, A.; Pastori, A.; Schivo, A.; Salviato, A.; Monne, I.; Terregino, C.
EPI1850130	NA	Czech Republ	2021-Jan-24	EPI_ISL_1180234	A/mute swan/Czech Republic/1656-1/2021	State Veterinary Institute Prague	State Veterinary Institute Prague	Nagy,A; Cernikova,L; Stara,M
EPI1846882	NA	Czech Republ	2021-Feb-04	EPI_ISL_1033124	A/mute swan/Czech Republic/2600/2021	State Veterinary Institute Prague	State Veterinary Institute Prague	Nagy,A; Cernikova,L; Stara,M
EPI1229993	NA	Netherlands	2006-Jan-24	EPI_ISL_309819	A/Turkey/Netherlands/06001571/2006	Wageningen Bioveterinary Research	Wageningen Bioveterinary Research	Bergervoet, Saskia; Heutink, Rene; Harders, Frank; Beerens, Nancy
EPI1883516	NA	Poland	2021-Apr-28	EPI_ISL_3102068	A/chicken/Poland/H984_21RS1385-9/2021	Istituto Zooprofilattico Sperimentale delle Venezie, EU/OIE/Reference Laboratory and FAO Reference Centre for AI and ND	Istituto Zooprofilattico Sperimentale Delle Venezie	Smietanka, K.; Swieton, E.; Zecchin, B.; Fusaro, A.; Milani, A.; Schivo, A.; Salviato, A.; Giussani, E.; Monne, I.; Terregino, C.
EPI1883468	NA	Poland	2021-May-11	EPI_ISL_3102062	A/chicken/Poland/H1124_21RS1385-3/2021	Istituto Zooprofilattico Sperimentale delle Venezie, EU/OIE/Reference Laboratory and FAO Reference Centre for AI and ND	Istituto Zooprofilattico Sperimentale Delle Venezie	Smietanka, K.; Swieton, E.; Zecchin, B.; Fusaro, A.; Milani, A.; Schivo, A.; Salviato, A.; Giussani, E.; Monne, I.; Terregino, C.
EPI1858605	NA	Bulgaria	2021-Feb-01	EPI_ISL_1719913	A/chicken/Bulgaria/39_21VIR1454-3/2021	Istituto Zooprofilattico Sperimentale delle Venezie, EU/OIE/Reference Laboratory and FAO Reference Centre for AI and ND	Istituto Zooprofilattico Sperimentale Delle Venezie	Goujgoulova, G.; Slavcheva, I.; Zecchin, B.; Fusaro, A.; Milani, A.; Schivo, A.; Salviato, A.; Giussani, E.; Monne, I.; Terregino, C.
EPI1858203	NA	Slovakia	2021-Jan-22	EPI_ISL_1665250	A/chicken/Slovakia/Pah10_21VIR1086-5/2021	Istituto Zooprofilattico Sperimentale delle Venezie, EU/OIE/Reference Laboratory and FAO Reference Centre for AI and ND	Istituto Zooprofilattico Sperimentale Delle Venezie	Dirb?kov?, Z.; Tin?k, M.; Zecchin, B.; Fusaro, A.; Pastori, A.; Schivo, A.; Salviato, A.; Monne, I.; Terregino, C.
EPI1858195	NA	Romania	2021-Feb-23	EPI_ISL_1665249	A/chicken/Romania/10101_21VIR2044-1/2021	Istituto Zooprofilattico Sperimentale delle Venezie, EU/OIE/Reference Laboratory and FAO Reference Centre for AI and ND	Istituto Zooprofilattico Sperimentale Delle Venezie	Onita, I.; Neicut, A.; Raluca, B.; Razvan, M.; Florica, B.; Zecchin, B.; Fusaro, A.; Pastori, A.; Schivo, A.; Salviato, A.; Monne, I.; Terregino, C.
EPI1858179	NA	Italy	2021-Feb-17	EPI_ISL_1665247	A/chicken/Italy/21VIR1151-2/2021	Istituto Zooprofilattico Sperimentale delle Venezie, EU/OIE/Reference Laboratory and FAO Reference Centre for AI and ND	Istituto Zooprofilattico Sperimentale Delle Venezie	Zecchin, B.; Fusaro, A.; Pastori, A.; Schivo, A.; Salviato, A.; Monne, I.; Terregino, C.
EPI1846307	NA	United Kingdo	2021-Jan-05	EPI_ISL_996003	A/chicken/Northern_Ireland/2021-000067_21VIR114-19/2021	AFBI - Agri-Food & Bioscience Institute	Istituto Zooprofilattico Sperimentale Delle Venezie	McMenamy, MJ.; Harkin, V.; Lemon, K.; Zecchin, B.; Fusaro, A.; Schivo, A.; Salviato, A.; Pastori, A.; Monne, I.; Terregino, C.
EPI1854322	NA	Czech Republ	2021-Mar-06	EPI_ISL_1399241	A/chicken/Czech Republic/4526/2021	State Veterinary Institute Prague	State Veterinary Institute Prague	Nagy,A;Cernikova,L;Stara,M
EPI1854266	NA	Czech Republ	2021-Feb-27	EPI_ISL_1399234	A/chicken/Czech Republic/4092-2/2021	State Veterinary Institute Prague	State Veterinary Institute Prague	Nagy,A;Cernikova,L;Stara,M
EPI1847758	NA	Czech Republ	2021-Feb-12	EPI_ISL_1058021	A/chicken/Czech Republic/3099-1/2021	State Veterinary Institute Prague	State Veterinary Institute Prague	Nagy,A; Cernikova,L; Stara,M
EPI866959	NA	Japan	2015-Oct-27	EPI_ISL_239417	A/duck/Aichi/231009/2015		National Institute of Animal Health	
EPI295953	NA	China	2008-Dec-15	EPI_ISL_84335	A/duck/Yangzhou/013/2008		Import from public-domain	Gu,M.; Zhao,G.; Song,Q.; Li,Y.; Liu,W.; Liu,X.
EPI600798	NA	China	2003-Dec-04	EPI_ISL_181415	A/migratory duck/Jiangxi/7231/2003		Import from public-domain	Ma,C.; Lam,T.T.Y.; Chai,Y.; Wang,J.; Fan,X.; Hong,W.; Zhang,Y.; Li,L.; Liu,Y.; Smith,D.K.; Webby,R.J.; Peiris,J.S.M.; Zhu,H.; Guan,Y.
EPI600792	NA	China	2003-Nov-25	EPI_ISL_181414	A/migratory duck/Jiangxi/6847/2003		Import from public-domain	Ma,C.; Lam,T.T.Y.; Chai,Y.; Wang,J.; Fan,X.; Hong,W.; Zhang,Y.; Li,L.; Liu,Y.; Smith,D.K.; Webby,R.J.; Peiris,J.S.M.; Zhu,H.; Guan,Y.
EPI268287	NA	China	2003-Jan-01	EPI_ISL_76429	A/duck/Hunan/5613/2003		Import from public-domain	Huang,K.; Bahl,J.; Fan,X.; Vijaykrishna,D.; Cheung,C.; Webby,R.J.; Webster,R.G.; Chen,H.; Smith,G.J.D.; Peiris,M.J.S.; Guan,Y.

EPI1883452	NA	Poland	2021-Mar-05	EPI_ISL_3102060	A/duck/Poland/H160_21RS1385-1/2021	Istituto Zooprofilattico Sperimentale delle Venezie, EU/OIE/Reference Laboratory and FAO Reference Centre for AI and ND	Istituto Zooprofilattico Sperimentale Delle Venezie	Smietanka, K.; Swieton, E.; Zecchin, B.; Fusaro, A.; Milani, A.; Schivo, A.; Salviato, A.; Giussani, E.; Monne, I.; Terregino, C.
EPI618645	NA	Sweden	2007-Nov-12	EPI_ISL_189401	A/mallard/Sweden/69794/2007		Import from public-domain	Wentworth,D.E.; Halpin,R.A.; Lin,X.; Bera,J.; Ransier,A.; Fedorova,N.; Tsitrin,T.; McLellan,M.; Stockwell,T.; Amedeo,P.; Appalla,L.; Bishop,B.; Edworthy,P.; Gupta,N.; Hoover,J.; Katzel,D.; Li,K.; Schobel,S.; Shrivastava,S.; Thovarai,V.; Wang,S.; Fouchier,R.; Osterhaus,A.; Olsen,B.; Wille,M.; Latorre-Margalef,N.; Tolf,C.; Bao,Y.; Sanders,R.; Zhdanov,S.; Kiryutin,B.; Lipman,D.J.; Tatusova,T.; Waldenstrom,J.
EPI618630	NA	Sweden	2007-Oct-16	EPI_ISL_189400	A/mallard/Sweden/68529/2007		Import from public-domain	Wentworth,D.E.; Halpin,R.A.; Lin,X.; Bera,J.; Ransier,A.; Fedorova,N.; Tsitrin,T.; McLellan,M.; Stockwell,T.; Amedeo,P.; Appalla,L.; Bishop,B.; Edworthy,P.; Gupta,N.; Hoover,J.; Katzel,D.; Li,K.; Schobel,S.; Shrivastava,S.; Thovarai,V.; Wang,S.; Fouchier,R.; Osterhaus,A.; Olsen,B.; Wille,M.; Latorre-Margalef,N.; Tolf,C.; Bao,Y.; Sanders,R.; Zhdanov,S.; Kiryutin,B.; Lipman,D.J.; Tatusova,T.; Waldenstrom,J.
EPI618616	NA	Sweden	2007-Oct-15	EPI_ISL_189399	A/mallard/Sweden/60069/2007		Import from public-domain	Wentworth,D.E.; Halpin,R.A.; Lin,X.; Bera,J.; Ransier,A.; Fedorova,N.; Tsitrin,T.; McLellan,M.; Stockwell,T.; Amedeo,P.; Appalla,L.; Bishop,B.; Edworthy,P.; Gupta,N.; Hoover,J.; Katzel,D.; Li,K.; Schobel,S.; Shrivastava,S.; Thovarai,V.; Wang,S.; Fouchier,R.; Osterhaus,A.; Olsen,B.; Wille,M.; Latorre-Margalef,N.; Tolf,C.; Bao,Y.; Sanders,R.; Zhdanov,S.; Kiryutin,B.; Lipman,D.J.; Tatusova,T.; Waldenstrom,J.
EPI618602	NA	Sweden	2006-Oct-08	EPI_ISL_189398	A/mallard/Sweden/50968/2006		Import from public-domain	Wentworth,D.E.; Halpin,R.A.; Lin,X.; Bera,J.; Ransier,A.; Fedorova,N.; Tsitrin,T.; McLellan,M.; Stockwell,T.; Amedeo,P.; Appalla,L.; Bishop,B.; Edworthy,P.; Gupta,N.; Hoover,J.; Katzel,D.; Li,K.; Schobel,S.; Shrivastava,S.; Thovarai,V.; Wang,S.; Fouchier,R.; Osterhaus,A.; Olsen,B.; Wille,M.; Latorre-Margalef,N.; Tolf,C.; Bao,Y.; Sanders,R.; Zhdanov,S.; Kiryutin,B.; Lipman,D.J.; Tatusova,T.; Waldenstrom,J.
EPI618665	NA	Sweden	2003-Oct-12	EPI_ISL_189395	A/mallard/Sweden/2213/2003		Import from public-domain	Wentworth,D.E.; Halpin,R.A.; Lin,X.; Bera,J.; Ransier,A.; Fedorova,N.; Tsitrin,T.; McLellan,M.; Stockwell,T.; Amedeo,P.; Appalla,L.; Bishop,B.; Edworthy,P.; Gupta,N.; Hoover,J.; Katzel,D.; Li,K.; Schobel,S.; Shrivastava,S.; Thovarai,V.; Wang,S.; Fouchier,R.; Osterhaus,A.; Olsen,B.; Wille,M.; Latorre-Margalef,N.; Tolf,C.; Bao,Y.; Sanders,R.; Zhdanov,S.; Kiryutin,B.; Lipman,D.J.; Tatusova,T.; Waldenstrom,J.
EPI618625	NA	Sweden	2002-Oct-31	EPI_ISL_189394	A/mallard/Sweden/343/2002		Import from public-domain	Wentworth,D.E.; Halpin,R.A.; Lin,X.; Bera,J.; Ransier,A.; Fedorova,N.; Tsitrin,T.; McLellan,M.; Stockwell,T.; Amedeo,P.; Appalla,L.; Bishop,B.; Edworthy,P.; Gupta,N.; Hoover,J.; Katzel,D.; Li,K.; Schobel,S.; Shrivastava,S.; Thovarai,V.; Wang,S.; Fouchier,R.; Osterhaus,A.; Olsen,B.; Wille,M.; Latorre-Margalef,N.; Tolf,C.; Bao,Y.; Sanders,R.; Zhdanov,S.; Kiryutin,B.; Lipman,D.J.; Tatusova,T.; Waldenstrom,J.
EPI514607	NA	Sweden	2006-Oct-05	EPI_ISL_158234	A/mallard/Sweden/50709/2006		Import from public-domain	Wentworth,D.E.; Halpin,R.A.; Lin,X.; Bera,J.; Ransier,A.; Fedorova,N.; Tsitrin,T.; McLellan,M.; Stockwell,T.; Amedeo,P.; Appalla,L.; Bishop,B.; Edworthy,P.; Gupta,N.; Hoover,J.; Katzel,D.; Li,K.; Schobel,S.; Shrivastava,S.; Thovarai,V.; Wang,S.; Fouchier,R.; Osterhaus,A.; Olsen,B.; Wille,M.; Latorre-Margalef,N.; Tolf,C.; Bao,Y.; Sanders,R.; Zhdanov,S.; Kiryutin,B.; Lipman,D.J.; Tatusova,T.; Waldenstrom,J.
EPI377768	NA	Sweden	2002-Jan-01	EPI_ISL_122071	A/mallard/Sweden/30/2002		Import from public-domain	Wentworth,D.E.; Dugan,V.; Halpin,R.; Lin,X.; Wester,E.; Ghedin,E.; Fedorova,N.; Tsitrin,T.; Stockwell,T.; Amedeo,P.; Bishop,B.; Edworthy,P.; Gupta,N.; Katzel,D.; Li,K.; Schobel,S.; Shrivastava,S.; Thovarai,V.; Wang,S.; Webster,R.; Webby,R.; Krauss,S.; Bao,Y.; Sanders,R.; Dernovoy,D.; Kiryutin,B.; Lipman,D.J.; Tatusova,T.
EPI251818	NA	Sweden	2003-Oct-12	EPI_ISL_73392	A/mallard/Sweden/86/2003		Import from public-domain	Fouchier,R.
EPI251663	NA	Netherlands	1999-Oct-07	EPI_ISL_73370	A/mallard/Netherlands/2/1999		Import from public-domain	Fouchier,R.
EPI190186	NA	Switzerland	2006-Dec-15	EPI_ISL_33832	A/mallard/Switzerland/WV4060167/2006		Import from public-domain	
EPI1883986	NA	Hungary	2021-Mar-08	EPI_ISL_3135926	A/eagle/Hungary/8569/2021 (H5N5)	National Food Chain Safety Office Veterinary Diagnostic Directorate Laboratory for Molecular Biology	National Food Chain Safety Office, Hungary	Katalin,Szentpáli-Gavallér;Ádám,Bálint;Krisztina,Ursu;Péter,Malik

EPI1884048	NA	Austria	2021-Apr-27	EPI_ISL_3142391	A/mute_swan/Austria/21051907_21VIR3291-7/2021	Istituto Zooprofilattico Sperimentale delle Venezie, EU/OIE/Reference Laboratory and FAO Reference Centre for AI and ND	Istituto Zooprofilattico Sperimentale Delle Venezie	Wodak, E.; Revilla Fernandez, S.; Schmoll, F.; Zecchin, B.; Fusaro, A.; Milani, A.; Schivo, A.; Salviato, A.; Giussani, E.; Monne, I.; Terregino, C.
EPI1883620	NA	Romania	2021-Apr-08	EPI_ISL_3102081	A/mute_swan/Romania/11981-1_21VIR3163-5/2021	Istituto Zooprofilattico Sperimentale delle Venezie, EU/OIE/Reference Laboratory and FAO Reference Centre for AI and ND	Istituto Zooprofilattico Sperimentale Delle Venezie	Onita, I.; Neicut, A.; Raluca, B.; Razvan, M.; Florica, B.; Zecchin, B.; Fusaro, A.; Giussani, E.; Schivo, A.; Salviato, A.; Monne, I.; Terregino, C.
EPI1883412	NA	Bulgaria	2021-Mar-30	EPI_ISL_3102055	A/grey_heron/Bulgaria/223_21VIR4270-2/2021	Istituto Zooprofilattico Sperimentale delle Venezie, EU/OIE/Reference Laboratory and FAO Reference Centre for AI and ND	Istituto Zooprofilattico Sperimentale Delle Venezie	Goujgoulova, G.; Slavcheva, I.; Zecchin, B.; Fusaro, A.; Milani, A.; Schivo, A.; Salviato, A.; Giussani, E.; Monne, I.; Terregino, C.
EPI1883404	NA	Bulgaria	2021-Mar-30	EPI_ISL_3102054	A/European_herring_gull/Bulgaria/222_21VIR4270-1/2021	Istituto Zooprofilattico Sperimentale delle Venezie, EU/OIE/Reference Laboratory and FAO Reference Centre for AI and ND	Istituto Zooprofilattico Sperimentale Delle Venezie	Goujgoulova, G.; Slavcheva, I.; Zecchin, B.; Fusaro, A.; Milani, A.; Schivo, A.; Salviato, A.; Giussani, E.; Monne, I.; Terregino, C.
EPI1847766	NA	Czech Republ	2021-Feb-12	EPI_ISL_1058022	A/chicken/Czech Republic/3099-2/2021	State Veterinary Institute Prague	State Veterinary Institute Prague	Nagy,A; Cernikova,L; Stara,M
EPI1848757	NA	Russian Fede	2021-Jan-29	EPI_ISL_1114757	A/turkey/Rostov-on-Don/332-09/2021	State Research Center of Virology and Biotechnology (VECTOR)	State Research Center of Virology and Biotechnology (VECTOR)	Natalia,Goncharova; Ivan,Susloparov; Natalia,Kolosova; Alexey,Danilenko; Juliya,Bulanovich; Vasiliy,Marchenko; Alexander,Ryzhikov
EPI1839528	NA	United Kingdo	2020-Dec-14	EPI_ISL_766056	A/falcon/England/041976/2020	Animal and Plant Health Agency (APHA)	Animal and Plant Health Agency (APHA)	
EPI1883476	NA	Poland	2021-May-05	EPI_ISL_3102063	A/goose/Poland/H1044_21RS1385-4/2021	Istituto Zooprofilattico Sperimentale delle Venezie, EU/OIE/Reference Laboratory and FAO Reference Centre for AI and ND	Istituto Zooprofilattico Sperimentale Delle Venezie	Smietanka, K.; Swieton, E.; Zecchin, B.; Fusaro, A.; Milani, A.; Schivo, A.; Salviato, A.; Giussani, E.; Monne, I.; Terregino, C.
EPI1811621	NA	Kazakhstan	2020-Sep-19	EPI_ISL_615073	A/domestic_goose/Kazakhstan/1-242_2-20-B/2020	National Veterinary Reference Center	Animal and Plant Health Agency (APHA)	
EPI1839252	NA	Russian Fede	2020-Aug-29	EPI_ISL_739685	A/goose/Russia_Omsk region/55-1/2020	Research Institute of Experimental and Clinical Medicine	WHO National Influenza Centre Russian Federation	Sobolev, I.; Sharshov, K.; Dubovitskiy, N.; Alekseev, A.; Leonov, S.; Irza, V.; Fadeev, A.; Danilenko, D.; Komissarov, A.; Shestopalov, A.
EPI1839244	NA	Russian Fede	2020-Sep-15	EPI_ISL_739684	A/goose/Russia_Novosibirsk region/1-12/2020	Research Institute of Experimental and Clinical Medicine	WHO National Influenza Centre Russian Federation	Sobolev, I.; Sharshov, K.; Dubovitskiy, N.; Alekseev, A.; Leonov, S.; Irza, V.; Fadeev, A.; Danilenko, D.; Komissarov, A.; Shestopalov, A.
EPI1858275	NA	Norway	2021-Jan-05	EPI_ISL_1665260	A/mute_swan/Norway/FU5_21VIR850-3/2021	Istituto Zooprofilattico Sperimentale delle Venezie, EU/OIE/Reference Laboratory and FAO Reference Centre for AI and ND	Istituto Zooprofilattico Sperimentale Delle Venezie	Madslien, K.; Moldal, T.; Gjerset, B.; Gudmundsson, S.; Follestad, A.; Tronerud, OH.; Dean, KR.; Akerstedt, J.; Jorgensen,HJ.; das Neves, CG.; Romo, G.; Zecchin, B.; Fusaro, A.; Pastori, A.; Schivo, A.; Salviato, A.; Monne, I.; Terregino, C.
EPI1859649	NA	Poland	2021-Feb-15	EPI_ISL_2111625	A/mute_swan/Poland/MB131/2021	National Veterinary Research Institut Poland, PIWet-PIB	National Veterinary Research Institute	Dziadek, K.; Swieton, E. ; Smietanka, K.
EPI1883994	NA	Hungary	2021-Mar-08	EPI_ISL_3135931	A/swan/Hungary/9638/2021 (H5N8)	National Food Chain Safety Office Veterinary Diagnostic Directorate Laboratory for Molecular Biology	National Food Chain Safety Office, Hungary	Katalin,Szentpáli-Gavallér;Ádám,Bálint;Krisztina,Ursu;Péter,Malik
EPI1848111	NA	Czech Republ	2021-Feb-14	EPI_ISL_1080480	A/mute swan/Czech Republic/3160-1/2021	State Veterinary Institute Prague	State Veterinary Institute Prague	Nagy,A; Cernikova,L; Stara,M
EPI1883556	NA	Poland	2021-May-14	EPI_ISL_3102073	A/turkey/Poland/H1184_21RS1385-14/2021	Istituto Zooprofilattico Sperimentale delle Venezie, EU/OIE/Reference Laboratory and FAO Reference Centre for AI and ND	Istituto Zooprofilattico Sperimentale Delle Venezie	Smietanka, K.; Swieton, E.; Zecchin, B.; Fusaro, A.; Milani, A.; Schivo, A.; Salviato, A.; Giussani, E.; Monne, I.; Terregino, C.
EPI1851834	NA	Norway	2020-Nov-30	EPI_ISL_1295639	A/turkey/Norway/FU496/2020	Norwegian Veterinary Institute	Animal and Plant Health Agency (APHA)	Britt Gjerset, Torfinn Moldal
EPI1858523	NA	Czech Republ	2021-Mar-25	EPI_ISL_1697197	A/australian brushturkey/Czech Republic/5904/2021	State Veterinary Institute Prague	State Veterinary Institute Prague	Nagy,A;Cernikova,L;Stara,M
EPI1857470	NA	Netherlands	2020-Nov-06	EPI_ISL_1575134	A/Mute Swan/Netherlands/3/2020	Erasmus Medical Center	Erasmus Medical Center	
EPI1857454	NA	Netherlands	2020-Nov-06	EPI_ISL_1575132	A/Herring Gull/Netherlands/2/2020	Erasmus Medical Center	Erasmus Medical Center	
EPI1883072	NA	Denmark	2020-Nov-15	EPI_ISL_984695	A/chicken/Denmark/14819-6/2020	Statens Serumm Institute	Statens Serum Institute	Yuan Liang, Charlotte Hjulsager
EPI1842056	NA	Poland	2020-Dec-04	EPI_ISL_846603	A/chicken/Poland/476/2020(H5N8)	National Veterinary Research Institut Poland, PIWet-PIB	National Veterinary Research Institut Poland, PIWet-PIB	Swieton, E.; Smietanka, K.
EPI1883923	NA	Kosovo	2021-May-25	EPI_ISL_3128531	A/chicken/Kosovo/90_21VIR5162-4/2021	Istituto Zooprofilattico Sperimentale delle Venezie, EU/OIE/Reference Laboratory and FAO Reference Centre for AI and ND	Istituto Zooprofilattico Sperimentale Delle Venezie	Uka, K.; Cana, A.; Merovci, X.; Krstevski, K.; Zecchin, B.; Fusaro, A.; Giussani, E.; Schivo, A.; Salviato, A.; Palumbo, E.; Monne, I.; Terregino, C.
EPI1883907	NA	Kosovo	2021-May-19	EPI_ISL_3128529	A/chicken/Kosovo/82_21VIR5162-1/2021	Istituto Zooprofilattico Sperimentale delle Venezie, EU/OIE/Reference Laboratory and FAO Reference Centre for AI and ND	Istituto Zooprofilattico Sperimentale Delle Venezie	Uka, K.; Cana, A.; Merovci, X.; Krstevski, K.; Zecchin, B.; Fusaro, A.; Giussani, E.; Schivo, A.; Salviato, A.; Palumbo, E.; Monne, I.; Terregino, C.
EPI1883428	NA	Bulgaria	2021-Apr-26	EPI_ISL_3102057	A/chicken/Bulgaria/275-4_21VIR4270-6/2021	Istituto Zooprofilattico Sperimentale delle Venezie, EU/OIE/Reference Laboratory and FAO Reference Centre for AI and ND	Istituto Zooprofilattico Sperimentale Delle Venezie	Goujgoulova, G.; Slavcheva, I.; Zecchin, B.; Fusaro, A.; Milani, A.; Schivo, A.; Salviato, A.; Giussani, E.; Monne, I.; Terregino, C.

EPI1883420	NA	Bulgaria	2021-Apr-23	EPI_ISL_3102056	A/chicken/Bulgaria/274-5_21VIR4270-4/2021	Istituto Zooprofilattico Sperimentale delle Venezie, EU/OIE/Reference Laboratory and FAO Reference Centre for AI and ND	Istituto Zooprofilattico Sperimentale Delle Venezie	Goujgoulova, G.; Slavcheva, I.; Zecchin, B.; Fusaro, A.; Milani, A.; Schivo, A.; Salviato, A.; Giussani, E.; Monne, I.; Terregino, C.
EPI1839260	NA	Kazakhstan	2020-Sep-18	EPI_ISL_739686	A/chicken/Kazakhstan/Kn-3/2020	Research Institute of Experimental and Clinical Medicine	WHO National Influenza Centre Russian Federation	Sobolev, I.; Sharshov, K.; Dubovitskiy, N.; Alekseev, A.; Leonov, S.; Irza, V.; Fadeev, A.; Danilenko, D.; Komissarov, A.; Shestopalov, A.
EPI1859544	NA	Czech Republ	2021-Apr-18	EPI_ISL_1941480	A/chicken/Czech Republic/7681-5/2021	State Veterinary Institute Prague	State Veterinary Institute Prague	Nagy,A;Cernikova,L;Stara,M
EPI1859512	NA	Czech Republ	2021-Apr-18	EPI_ISL_1941365	A/chicken/Czech Republic/7682-5/2021	State Veterinary Institute Prague	State Veterinary Institute Prague	Nagy,A;Cernikova,L;Stara,M
EPI1859504	NA	Czech Republ	2021-Apr-18	EPI_ISL_1941351	A/chicken/Czech Republic/7682-9/2021	State Veterinary Institute Prague	State Veterinary Institute Prague	Nagy,A;Cernikova,L;Stara,M
EPI1858475	NA	Czech Republ	2021-Apr-01	EPI_ISL_1697191	A/chicken/Czech Republic/6527/2021	State Veterinary Institute Prague	State Veterinary Institute Prague	Nagy,A;Cernikova,L;Stara,M
EPI1854250	NA	Czech Republ	2021-Feb-25	EPI_ISL_1399232	A/chicken/Czech Republic/3893/2021	State Veterinary Institute Prague	State Veterinary Institute Prague	Nagy,A;Cernikova,L;Stara,M
EPI1883484	NA	Poland	2021-May-01	EPI_ISL_3102064	A/duck/Poland/H1029_21RS1385-5/2021	Istituto Zooprofilattico Sperimentale delle Venezie, EU/OIE/Reference Laboratory and FAO Reference Centre for AI and ND	Istituto Zooprofilattico Sperimentale Delle Venezie	Smietanka, K.; Swieton, E.; Zecchin, B.; Fusaro, A.; Milani, A.; Schivo, A.; Salviato, A.; Giussani, E.; Monne, I.; Terregino, C.
EPI1811679	NA	Russian Fede	2020-Sep-18	EPI_ISL_626649	A/duck/Russian_Federation/Saratov/1578-2/2020	Federal Centre for Animal Health (ARRIAH)	Animal and Plant Health Agency (APHA)	
EPI1859576	NA	Czech Republ	2021-Apr-06	EPI_ISL_1941580	A/duck/Czech Republic/6653-15/2021	State Veterinary Institute Prague	State Veterinary Institute Prague	Nagy,A;Cernikova,L;Stara,M
EPI1859568	NA	Czech Republ	2021-Apr-06	EPI_ISL_1941579	A/duck/Czech Republic/6653-5/2021	State Veterinary Institute Prague	State Veterinary Institute Prague	Nagy,A;Cernikova,L;Stara,M
EPI1883859	NA	Greece	2021-Mar-22	EPI_ISL_3128523	A/dalmatian pelican/Greece/47t_21VIR3735-3/2021	Istituto Zooprofilattico Sperimentale delle Venezie, EU/OIE/Reference Laboratory and FAO Reference Centre for AI and ND	Istituto Zooprofilattico Sperimentale Delle Venezie	Georgiades, G.; Ragias, B.; Gkolia, A.; Anthopoulou, E.; Zecchin, B.; Fusaro, A.; Giussani, E.; Schivo, A.; Salviato, A.; Palumbo, E.; Monne, I.; Terregino, C.
EPI1883596	NA	Poland	2021-Apr-22	EPI_ISL_3102078	A/mute_swan/Poland/MB396_21RS1385-19/2021	Istituto Zooprofilattico Sperimentale delle Venezie, EU/OIE/Reference Laboratory and FAO Reference Centre for AI and ND	Istituto Zooprofilattico Sperimentale Delle Venezie	Smietanka, K.; Swieton, E.; Zecchin, B.; Fusaro, A.; Milani, A.; Schivo, A.; Salviato, A.; Giussani, E.; Monne, I.; Terregino, C.
EPI1860054	NA	Italy	2021-Jan-28	EPI_ISL_2234810	A/seagull/Italy/21VIR2479/2021	Istituto Zooprofilattico Sperimentale delle Venezie, EU/OIE/Reference Laboratory and FAO Reference Centre for AI and ND	Istituto Zooprofilattico Sperimentale Delle Venezie	Zecchin, B.; Fusaro, A.; Schivo, A.; Salviato, A.; Giussani, E.; Monne, I.; Terregino, C.
EPI1815152	NA	Italy	2020-Nov-14	EPI_ISL_683593	A/Eurasian_wigeon/Italy/20VIR7139-121/2020	Istituto Zooprofilattico Sperimentale delle Venezie, EU/OIE/Reference Laboratory and FAO Reference Centre for AI and ND	Istituto Zooprofilattico Sperimentale Delle Venezie	Zecchin, B.; Fusaro, A.; Pastori, A.; Milani, A.; Salviato, A.; Schivo, A.; Monne, I.; Terregino, C.
EPI1814608	NA	Italy	2020-Nov-14	EPI_ISL_654958	A/mallard/Italy/20VIR7139-73/2020	Istituto Zooprofilattico Sperimentale delle Venezie, EU/OIE/Reference Laboratory and FAO Reference Centre for AI and ND	Istituto Zooprofilattico Sperimentale Delle Venezie	Zecchin, B.; Fusaro, A.; Pastori, A.; Milani, A.; Salviato, A.; Schivo, A.;Monne, I.; Terregino, C.
EPI1858235	NA	Italy	2021-Feb-23	EPI_ISL_1665255	A/guinea_fowl/Italy/21VIR1293-20/2021	Istituto Zooprofilattico Sperimentale delle Venezie, EU/OIE/Reference Laboratory and FAO Reference Centre for AI and ND	Istituto Zooprofilattico Sperimentale Delle Venezie	Zecchin, B.; Fusaro, A.; Pastori, A.; Schivo, A.; Salviato, A.; Monne, I.; Terregino, C.
EPI1883056	NA	Denmark	2020-Nov-07	EPI_ISL_984693	A/barnacle goose/Denmark/14599-1/2020	Statens Serumm Institute	Statens Serum Institute	Yuan Liang, Charlotte Hjulsager
EPI1814737	NA	Sweden	2020-Nov-12	EPI_ISL_668457	A/barnacle goose/Sweden/SVA201117SZ0468/KN003355/2020	National Veterinary Institute, SVA	National Veterinary Institute	
EPI1858403	NA	Czech Republ	2021-Mar-25	EPI_ISL_1697182	A/chicken/Czech Republic/5903/2021	State Veterinary Institute Prague	State Veterinary Institute Prague	Nagy,A;Cernikova,L;Stara,M
EPI1850138	NA	Czech Republ	2021-Jan-22	EPI_ISL_1191587	A/chicken/Czech Republic/1566-2/2021	State Veterinary Institute Prague	State Veterinary Institute Prague	Nagy,A;Cernikova,L;Stara,M
EPI1848805	NA	Russian Fede	2021-Jan-05	EPI_ISL_1114763	A/chicken/Krasnodar/334-03/2021	State Research Center of Virology and Biotechnology (VECTOR)	State Research Center of Virology and Biotechnology (VECTOR)	Natalia,Goncharova; Ivan,Susloparov; Natalia,Kolosova; Alexey,Danilenko; Juliya,Bulanovich; Vasiliy,Marchenko; Alexander,Ryzhikov
EPI1848789	NA	Russian Fede	2021-Jan-05	EPI_ISL_1114761	A/chicken/Krasnodar/334-02/2021	State Research Center of Virology and Biotechnology (VECTOR)	State Research Center of Virology and Biotechnology (VECTOR)	Natalia,Goncharova; Ivan,Susloparov; Natalia,Kolosova; Alexey,Danilenko; Juliya,Bulanovich; Vasiliy,Marchenko; Alexander,Ryzhikov
EPI1846995	NA	Russian Fede	2020-Dec-12	EPI_ISL_1039235	A/chicken/Astrakhan/321-05/2020	State Research Center of Virology and Biotechnology (VECTOR)	State Research Center of Virology and Biotechnology (VECTOR)	Natalia,Goncharova; Ivan,Susloparov; Natalia,Kolosova; Alexey,Danilenko; Juliya,Bulanovich; Vasiliy,Marchenko; Alexander,Ryzhikov
EPI1846979	NA	Russian Fede	2020-Dec-12	EPI_ISL_1039232	A/chicken/Astrakhan/321-01/2020	State Research Center of Virology and Biotechnology (VECTOR)	State Research Center of Virology and Biotechnology (VECTOR)	
EPI1813224	NA	Russian Fede	2020-Aug-17	EPI_ISL_644135	A/chicken/Omsk/0119/2020	State Research Center of Virology and Biotechnology (VECTOR)	State Research Center of Virology and Biotechnology (VECTOR)	Natalia,Goncharova; Ivan,Susloparov; Natalia,Kolosova; Alexey,Danilenko; Juliya,Bulanovich; Vasiliy,Marchenko; Alexander,Ryzhikov

EPI1813184	NA	Russian Fede	2020-Aug-17	EPI_ISL_644130	A/goose/Omsk/01161/2020	State Research Center of Virology and Biotechnology (VECTOR)	State Research Center of Virology and Biotechnology (VECTOR)	Natalia,Goncharova; Ivan,Susloparov; Natalia,Kolosova; Alexey,Danilenko; Juliya,Bulanovich; Vasiliy,Marchenko; Alexander,Ryzhikov
EPI1813136	NA	Russian Fede	2020-Aug-13	EPI_ISL_644124	A/duck/Omsk/0004/2020	State Research Center of Virology and Biotechnology (VECTOR)	State Research Center of Virology and Biotechnology (VECTOR)	Natalia,Goncharova; Ivan,Susloparov; Natalia,Kolosova; Alexey,Danilenko; Juliya,Bulanovich; Vasiliy,Marchenko; Alexander,Ryzhikov
EPI1814713	NA	Croatia	2020-Nov-17	EPI_ISL_666890	A/turkey/Croatia/104/2020	Croatian Veterinary Institute, Poultry Centre	Croatian Veterinary Institute	Savić, Vladimir
EPI1848765	NA	Russian Fede	2021-Jan-29	EPI_ISL_1114758	A/turkey/Rostov-on-Don/332-10/2021	State Research Center of Virology and Biotechnology (VECTOR)	State Research Center of Virology and Biotechnology (VECTOR)	Natalia,Goncharova; Ivan,Susloparov; Natalia,Kolosova; Alexey,Danilenko; Juliya,Bulanovich; Vasiliy,Marchenko; Alexander,Ryzhikov
EPI1813832	NA	Sweden	2020-Nov-13	EPI_ISL_647969	A/Turkey/Sweden/SVA201114SZ0001/20KN303106/2020	National Veterinary Institute, SVA	National Veterinary Institute	
EPI954790	NA	Hungary	2017-Jan-24	EPI_ISL_255210	A/Harris_hawk/Hungary/2762a/2017	Danam.Vet.Molbiol	Danam.Vet.Molbiol	Adam, Dan
EPI869925	NA	Poland	2016-Dec-02	EPI_ISL_240102	A/domestic goose/Poland/33/2016		National Veterinary Research Institut Poland, PIWet-PIB	E. Swieton, K. Smietanka
EPI1811687	NA	Russian Fede	2020-Aug-20	EPI_ISL_626651	A/goose/Russian_Federation/Kurgan/1345-25/2020	Federal Centre for Animal Health (ARRIAH)	Animal and Plant Health Agency (APHA)	
EPI1811594	NA	Kazakhstan	2020-Sep-22	EPI_ISL_615065	A/domestic_goose/Kazakhstan/1-261_2-20-B/2020	National Veterinary Reference Center	Animal and Plant Health Agency (APHA)	
EPI1019709	NA	Netherlands	2016-Nov-16	EPI_ISL_268661	A/L-bl-ba-gull/NL-Sovon/16014324-014/2016	Wageningen Bioveterinary Research	Wageningen Bioveterinary Research	Beerens, Nancy; Heutink, Rene; Harders, Frank; Verschuren-Pritz, Sylvia; Bossers, Alex; Koch, Guus; Bergervoet, Saskia
EPI1261939	NA	Italy	2017-Oct-11	EPI_ISL_316587	A/swan/Italy/17VIR9038-2/2017	Istituto Zooprofilattico Sperimentale Delle Venezie	Istituto Zooprofilattico Sperimentale Delle Venezie	Mulatti, P.; Fusaro, A.; Scolamacchia, F.; Zecchin, B.; Azzolini, A.; Zamperin, G.; Milani, A.; Salviato, A.; Schivo, A.; Terregino, C.; Bonfanti, L.; Monne, I.; Marangon, S.
EPI1126369	NA	Czech Republ	2017-Jan-20	EPI_ISL_268939	A/mute swan/Czech Republic/987-17_2/2017 (H5N8)	State Veterinary Institute Prague	State Veterinary Institute Prague	Nagy,A
EPI1498952	NA	United Kingdo	2016-Dec-31	EPI_ISL_366240	A/Cygnus olor/England/WVZP/2016		Import from public-domain	Hill,S.C.; Hansen,R.; Watson,S.; Coward,V.; Russell,C.; Cooper,J.; Essen,S.; Everest,H.; Parag,K.V.; Fiddaman,S.; Reid,S.; Lewis,N.; Brookes,S.M.; Smith,A.L.; Sheldon,B.; Perrins,C.M.; Brown,I.H.; Pybus,O.G.
EPI1848733	NA	Russian Fede	2020-Dec-31	EPI_ISL_1114754	A/mute swan/North Ossetia-Alania/325-03/2020	State Research Center of Virology and Biotechnology (VECTOR)	State Research Center of Virology and Biotechnology (VECTOR)	Natalia,Goncharova; Ivan,Susloparov; Natalia,Kolosova; Alexey,Danilenko; Juliya,Bulanovich; Vasiliy,Marchenko; Alexander,Ryzhikov
EPI1185021	NA	Poland	2017-Mar-08	EPI_ISL_300705	A/turkey/Poland/285/2017	National Veterinary Research Institut Poland, PIWet-PIB	National Veterinary Research Institut Poland, PIWet-PIB	Swieton E., Smietanka K.
EPI544759	NA	Germany	2014-Nov-04	EPI_ISL_167140	A/turkey/Germany-MV/R2472/2014		Friedrich-Loeffler-Institut	
EPI555068	NA	Italy	2014-Dec-15	EPI_ISL_169350	A/turkey/Italy/14VIR7898-10/2014	Istituto Zooprofilattico Sperimentale Delle Venezie	Istituto Zooprofilattico Sperimentale Delle Venezie	Luca,Tassoni; Silvia,Ormelli; Alessia,Schivo; Alice,Fusaro; Isabella,Monne; Giovanni,Cattoli
EPI1261985	NA	Italy	2017-Oct-23	EPI_ISL_316593	A/pigeon/Italy/17VIR9842/2017	Istituto Zooprofilattico Sperimentale Delle Venezie	Istituto Zooprofilattico Sperimentale Delle Venezie	Mulatti, P.; Fusaro, A.; Scolamacchia, F.; Zecchin, B.; Azzolini, A.; Zamperin, G.; Milani, A.; Salviato, A.; Schivo, A.; Terregino, C.; Bonfanti, L.; Monne, I.; Marangon, S.
EPI1019725	NA	Netherlands	2016-Nov-16	EPI_ISL_268663	A/Magpie/NL-Volendam/16014331-002/2016	Wageningen Bioveterinary Research	Wageningen Bioveterinary Research	Beerens, Nancy; Heutink, Rene; Harders, Frank; Verschuren-Pritz, Sylvia; Bossers, Alex; Koch, Guus; Bergervoet, Saskia
EPI954646	NA	Hungary	2017-Jan-11	EPI_ISL_255192	A/Goose/Hungary/982/2017	Danam.Vet.Molbiol	Danam.Vet.Molbiol	Adam, Dan
EPI954630	NA	Hungary	2017-Jan-02	EPI_ISL_255190	A/Mute swan/Hungary/119/2017	Danam.Vet.Molbiol	Danam.Vet.Molbiol	Adam, Dan
EPI1857486	NA	Netherlands	2020-Nov-06	EPI_ISL_1575136	A/Mute Swan/Netherlands/5/2020	Erasmus Medical Center	Erasmus Medical Center	
EPI1857462	NA	Netherlands	2020-Nov-06	EPI_ISL_1575133	A/Herring Gull/Netherlands/3/2020	Erasmus Medical Center	Erasmus Medical Center	
EPI1721785	NA	Bulgaria	2018-Mar-02	EPI_ISL_419344	A/chicken/Bulgaria/Dobrich/12-2/2018	NDRVMI (National Diagnostic and Research Veterinary Medical Institute)	University of Cambridge	
EPI1261724	NA	Italy	2017-Oct-28	EPI_ISL_316560	A/chicken/Italy/17VIR9680/2017	Istituto Zooprofilattico Sperimentale Delle Venezie	Istituto Zooprofilattico Sperimentale Delle Venezie	Mulatti, P.; Fusaro, A.; Scolamacchia, F.; Zecchin, B.; Azzolini, A.; Zamperin, G.; Milani, A.; Salviato, A.; Schivo, A.; Terregino, C.; Bonfanti, L.; Monne, I.; Marangon, S.
EPI1811630	NA	Iraq	2020-May-12	EPI_ISL_623074	A/chicken/Iraq/1/2020	Central Veterinary Labs	Animal and Plant Health Agency (APHA)	
EPI931195	NA	Czech Republ	2017-Feb-07	EPI_ISL_250918	A/chicken/Czech Republic/1863-17_1/2017 (H5N8)		State Veterinary Institute Prague	Nagy, Alexander

EPI552778	NA	Netherlands	2014-Nov-21	EPI_ISL_169282	A/chicken/Netherlands/emc-3/2014	Erasmus Medical Center	Erasmus Medical Center	Fouchier, Ron A.M.; Bestebroer, Theo; Van den Brand, Judith M.A.; Van der Vliet, Stefan; Verhagen, Josanne H.
EPI959434	NA	Poland	2016-Dec-28	EPI_ISL_255915	A/chicken/Poland/114/2016		National Veterinary Research Institut Poland, PIWet-PIB	Swieton E., Smietanka K.
EPI869933	NA	Poland	2016-Dec-17	EPI_ISL_240104	A/chicken/Poland/79A/2016		National Veterinary Research Institut Poland, PIWet-PIB	E. Swieton, K. Smietanka
EPI573167	NA	Netherlands	2014-Nov-19	EPI_ISL_174349	A/chicken/Netherlands/14015766/2014	Wageningen Bioveterinary Research	Wageningen Bioveterinary Research	Heutink, Rene; Harders, Frank; Verschuren-Pritz, Sylvia; Bossers, Alex; Koch, Guus; Bouwstra, Ruth
EPI1721889	NA	Bulgaria	2018-Apr-17	EPI_ISL_419357	A/duck/Bulgaria/Plovdiv/76-1/2018	NDRVMI (National Diagnostic and Research Veterinary Medical Institute)	University of Cambridge	
EPI1721857	NA	Bulgaria	2018-Apr-02	EPI_ISL_419353	A/duck/Bulgaria/Yambol/35-1/2018	NDRVMI (National Diagnostic and Research Veterinary Medical Institute)	University of Cambridge	
EPI926615	NA	Russian Fede	2016-Oct-04	EPI_ISL_240677	A/domestic duck/Siberia/103/2016	Research Institute of Experimental and Clinical Medicine	Research Institute of Experimental and Clinical Medicine	Sharshov, K.A.; Kurskaya, O.G.; Alexeev, A.Y.; Sobolev, I.A.; Alikina, T.Y.; Kabilov, M.R.; Shestopalov, A.M.
EPI1019469	NA	Netherlands	2016-Dec-01	EPI_ISL_268631	A/Dk/NL-Biddinghuizen/16015145-021-025/2016	Wageningen Bioveterinary Research	Wageningen Bioveterinary Research	Beerens, Nancy; Heutink, Rene; Harders, Frank; Verschuren-Pritz, Sylvia; Bossers, Alex; Koch, Guus; Bergervoet, Saskia
EPI596297	NA	Netherlands	2014-Nov-24	EPI_ISL_181094	A/eurasian wigeon/Netherlands/2/2014		Import from public-domain	Verhagen,J.H.; Van der Jeugd,H.P.; Nolet,B.A.; Vuong,O.; Majoor,F.; De Vries,P.P.; Kharitonov,S.; Kuiken,T.; Fouchier,R.A.M.
EPI1023607	NA	Netherlands	2016-Dec-05	EPI_ISL_269696	A/Eurasian_Wigeon/Netherlands/23/2016	Erasmus Medical Center	Erasmus Medical Center	Poen,M.J.;Van Der Jeugd,H.P.;Vuong,O.;Scheuer,R.D.;Kleyheeg,E.; Bestebroer,T.M.;Begeman,L;van den Brand,J.M.A.;Kuiken,T.;Fouchier,R.A.M.
EPI585113	NA	Netherlands	2015-Feb-25	EPI_ISL_177649	A/eurasian wigeon/Netherlands/1/2015	Erasmus Medical Center	Erasmus Medical Center	Verhagen, Josanne; Vuong, Oanh; Van der Jeugd, Henk; Nolet, Bart; Fouchier, Ron
EPI551150	NA	Netherlands	2014-Nov-24	EPI_ISL_168747	A/eurasian wigeon/Netherlands/emc-2/2014	Erasmus Medical Center	Erasmus Medical Center	Fouchier, Ron A.M.; Verhagen, Josanne H.; Vuong, Oanh; Bestebroer, Theo; Van Vliet, Stefan; Van der Jeugd, Henk
EPI1019517	NA	Netherlands	2016-Nov-27	EPI_ISL_268637	A/Eur_Wig/NL-De Waal (Texel)/16014891-004/2016	Wageningen Bioveterinary Research	Wageningen Bioveterinary Research	Beerens, Nancy; Heutink, Rene; Harders, Frank; Verschuren-Pritz, Sylvia; Bossers, Alex; Koch, Guus; Bergervoet, Saskia
EPI860515	NA	Hungary	2016-Nov-08	EPI_ISL_237964	A/Mulard_duck/Hungary/54494/2016	National Food Chain Safety Office Veterinary Diagnostic Directorate Laboratory for Molecular Biology	Danam.Vet.Molbiol	Zsuzsanna, Ronai; Krisztina, Ursu; Adam, Balint; Dora, Szalay; Akos, Thuma; Eva, Gyuris; Karoly, Erdelyi; Adam, Dan
EPI1019757	NA	Netherlands	2016-Dec-05	EPI_ISL_268667	A/Sea_eagle/NL-Assen/16015398-002/2016	Wageningen Bioveterinary Research	Wageningen Bioveterinary Research	Beerens, Nancy; Heutink, Rene; Harders, Frank; Verschuren-Pritz, Sylvia; Bossers, Alex; Koch, Guus; Bergervoet, Saskia
EPI1019381	NA	Netherlands	2016-Nov-30	EPI_ISL_268620	A/Buzzard/NL-Durgerdam/16015100-004/2016	Wageningen Bioveterinary Research	Wageningen Bioveterinary Research	Beerens, Nancy; Heutink, Rene; Harders, Frank; Verschuren-Pritz, Sylvia; Bossers, Alex; Koch, Guus; Bergervoet, Saskia
EPI1019805	NA	Netherlands	2016-Nov-14	EPI_ISL_268673	A/T_Dk/NL-Werkendam/16014159-003/2016	Wageningen Bioveterinary Research	Wageningen Bioveterinary Research	Beerens, Nancy; Heutink, Rene; Harders, Frank; Verschuren-Pritz, Sylvia; Bossers, Alex; Koch, Guus; Bergervoet, Saskia
EPI1848653	NA	Russian Fede	2020-Oct-17	EPI_ISL_1114742	A/chicken/Kostroma/304-08/2020	State Research Center of Virology and Biotechnology (VECTOR)	State Research Center of Virology and Biotechnology (VECTOR)	Natalia,Goncharova; Ivan,Susloparov; Natalia,Kolosova; Alexey,Danilenko; Juliya,Bulanovich; Vasiliy,Marchenko; Alexander,Ryzhikov
EPI1847019	NA	Russian Fede	2020-Dec-12	EPI_ISL_1039239	A/chicken/Astrakhan/321-10/2020	State Research Center of Virology and Biotechnology (VECTOR)	State Research Center of Virology and Biotechnology (VECTOR)	Natalia,Goncharova; Ivan,Susloparov; Natalia,Kolosova; Alexey,Danilenko; Juliya,Bulanovich; Vasiliy,Marchenko; Alexander,Ryzhikov
EPI1272571	NA	Russian Fede	2018-Aug-01	EPI_ISL_320958	A/chicken/Tatarstan/7/2018		State Research Center of Virology and Biotechnology (VECTOR)	Alexey,Danilenko; Vasiliy,Marchenko; Ivan,Susloparov; Natalya,Goncharova; Natalya,Kolosova; Juliya, Bulanovich; Alexander,Ryzhikov
EPI1272563	NA	Russian Fede	2018-Jul-31	EPI_ISL_320957	A/chicken/Cheboksary/854/2018		State Research Center of Virology and Biotechnology (VECTOR)	Alexey,Danilenko; Vasiliy,Marchenko; Ivan,Susloparov; Natalya,Goncharova; Natalya,Kolosova; Juliya, Bulanovich; Alexander,Ryzhikov
EPI1272539	NA	Russian Fede	2018-Jul-20	EPI_ISL_320954	A/chicken/Cheboksary/850/2018		State Research Center of Virology and Biotechnology (VECTOR)	Alexey,Danilenko; Vasiliy,Marchenko; Ivan,Susloparov; Natalya,Goncharova; Natalya,Kolosova; Juliya, Bulanovich; Alexander,Ryzhikov
EPI1271020	NA	Russian Fede	2018-Jul-04	EPI_ISL_320685	A/chicken/Samara/679/2018		State Research Center of Virology and Biotechnology (VECTOR)	Alexey,Danilenko; Vasiliy,Marchenko; Ivan,Susloparov; Natalya,Goncharova; Natalya,Kolosova; Juliya, Bulanovich; Alexander,Ryzhikov
EPI1270988	NA	Russian Fede	2018-Jun-26	EPI_ISL_320681	A/chicken/Kursk/760/2018		State Research Center of Virology and Biotechnology (VECTOR)	Alexey,Danilenko; Vasiliy,Marchenko; Ivan,Susloparov; Natalya,Goncharova; Natalya,Kolosova; Juliya, Bulanovich; Alexander,Ryzhikov

EPI1270956	NA	Russian Fede	2018-Jun-19	EPI_ISL_320677	A/chicken/Penza/607/2018		State Research Center of Virology and Biotechnology (VECTOR)	Alexey,Danilenko; Vasiliy,Marchenko; Ivan,Susloparov; Natalya,Goncharova; Natalya,Kolosova; Juliya, Bulanovich; Alexander,Ryzhikov
EPI1270932	NA	Russian Fede	2018-Jul-09	EPI_ISL_320674	A/chicken/Rostov-on-Don/766/2018		State Research Center of Virology and Biotechnology (VECTOR)	Alexey,Danilenko; Vasiliy,Marchenko; Ivan,Susloparov; Natalya,Goncharova; Natalya,Kolosova; Juliya, Bulanovich; Alexander,Ryzhikov
EPI1270891	NA	Russian Fede	2018-Jun-15	EPI_ISL_320663	A/chicken/Penza/301/2018		State Research Center of Virology and Biotechnology (VECTOR)	Alexey,Danilenko; Vasiliy,Marchenko; Ivan,Susloparov; Natalya,Goncharova; Natalya,Kolosova; Juliya, Bulanovich; Alexander,Ryzhikov
EPI1813392	NA	Russian Fede	2020-Aug-17	EPI_ISL_644156	A/goose/Omsk/011101/2020	State Research Center of Virology and Biotechnology (VECTOR)	State Research Center of Virology and Biotechnology (VECTOR)	Natalia,Goncharova; Ivan,Susloparov; Natalia,Kolosova; Alexey,Danilenko; Juliya,Bulanovich; Vasiliy,Marchenko; Alexander,Ryzhikov
EPI555122	NA	Germany	2014-Dec-30	EPI_ISL_169633	A/mallard/Germany-ST/R23/2015		Friedrich-Loeffler-Institut	
EPI576393	NA	Sweden	2015-Mar-05	EPI_ISL_175535	A/MuteSwan/Sweden/SVA150313KU0141/SZ543/2015	National Veterinary Institute	National Veterinary Institute	'Zohari,Siamak';'Karin,Ullman';Olofsson,Ann-Sophie*
EPI869935	NA	Poland	2016-Dec-21	EPI_ISL_240106	A/mute swan/Poland/108/2016		National Veterinary Research Institut Poland, PIWet-PIB	E. Swieton, K. Smietanka
EPI954606	NA	Italy	2017-Feb-16	EPI_ISL_255187	A/turkey/Italy/17VIR1452-22/2017	Istituto Zooprofilattico Sperimentale Delle Venezie	Istituto Zooprofilattico Sperimentale Delle Venezie	Zecchin, B.; Fusaro, A.; Milani, A.; Schivo, A.; Salviato, A.; Zamperin, G.; Marciano, S.; Ormelli, S.; Terregino, C.; Monne, I.
EPI1261366	NA	Italy	2017-Mar-28	EPI_ISL_316515	A/turkey/Italy/17VIR2722-29/2017	Istituto Zooprofilattico Sperimentale Delle Venezie	Istituto Zooprofilattico Sperimentale Delle Venezie	Mulatti, P.; Fusaro, A.; Scolamacchia, F.; Zecchin, B.; Azzolini, A.; Zamperin, G.; Milani, A.; Salviato, A.; Schivo, A.; Terregino, C.; Bonfanti, L.; Monne, I.; Marangon, S.
EPI1261630	NA	Italy	2017-Oct-09	EPI_ISL_316548	A/turkey/Italy/17VIR8819-3/2017	Istituto Zooprofilattico Sperimentale Delle Venezie	Istituto Zooprofilattico Sperimentale Delle Venezie	Mulatti, P.; Fusaro, A.; Scolamacchia, F.; Zecchin, B.; Azzolini, A.; Zamperin, G.; Milani, A.; Salviato, A.; Schivo, A.; Terregino, C.; Bonfanti, L.; Monne, I.; Marangon, S.
EPI1270851	NA	Russian Fede	2018-Jun-22	EPI_ISL_320658	A/chicken/Samara/447/2018		State Research Center of Virology and Biotechnology (VECTOR)	Alexey,Danilenko; Vasiliy,Marchenko; Ivan,Susloparov; Natalya,Goncharova; Natalya,Kolosova; Juliya, Bulanovich; Alexander,Ryzhikov
EPI1270875	NA	Russian Fede	2018-Jun-22	EPI_ISL_320661	A/goose/Samara/459/2018		State Research Center of Virology and Biotechnology (VECTOR)	Alexey,Danilenko; Vasiliy,Marchenko; Ivan,Susloparov; Natalya,Goncharova; Natalya,Kolosova; Juliya, Bulanovich; Alexander,Ryzhikov
EPI1351445	NA	Russian Fede	2018-Nov-02	EPI_ISL_336933	A/chicken/Voronezh/1513/2018		State Research Center of Virology and Biotechnology (VECTOR)	Natalya,Goncharova; Alexey,Danilenko; Vasiliy,Marchenko; Ivan,Susloparov; Natalya,Kolosova; Juliya, Bulanovich; Alexander,Ryzhikov
EPI1775500	NA	Belgium	2017-Nov-19	EPI_ISL_502736	A/Anas platyrhynchos/Belgium/10402_H195386/2017		Import from public-domain	Lambrecht,B.; Steensels,M.; Fusaro,A.; Milani,A.; Pastori,A.; Schivo,A.; Salviato,A.; Zamperin,G.; Monne,I.; Terregino,C.
EPI1581363	NS	Bangladesh	2018-Dec-09	EPI_ISL_387986	A/duck/Bangladesh/36395/2018		Import from public-domain	Barman,S.; Turner,J.C.; Hasan,M.; Akhtar,S.; Franks,J.; El-Shesheny,R.; Walker,D.; Seiler,P.; Friedman,K.; Kercher,L.; McKenzie,P.; Webby,R.J.; Feeroz,M.; Webster,R.G.
EPI1882958	NS	Denmark	2020-Oct-26	EPI_ISL_3031133	A_mallard_Denmark_12946-11_2020-10-26	Statens Serumm Institute	Statens Serum Institute	Hjulsager, Charlotte

We gratefully acknowledge the authors, originating and submitting laboratories of the sequences from GISAID's EpiFlu™ Database on which this research is based. The list is detailed below.
All submitters of data may be contacted directly via www.gisaid.org
